# Mapping 123 million neonatal, infant and child deaths between 2000 and 2017

**DOI:** 10.1038/s41586-019-1545-0

**Published:** 2019-10-16

**Authors:** Roy Burstein, Nathaniel J. Henry, Michael L. Collison, Laurie B. Marczak, Amber Sligar, Stefanie Watson, Neal Marquez, Mahdieh Abbasalizad-Farhangi, Masoumeh Abbasi, Foad Abd-Allah, Amir Abdoli, Mohammad Abdollahi, Ibrahim Abdollahpour, Rizwan Suliankatchi Abdulkader, Michael R. M. Abrigo, Dilaram Acharya, Oladimeji M. Adebayo, Victor Adekanmbi, Davoud Adham, Mahdi Afshari, Mohammad Aghaali, Keivan Ahmadi, Mehdi Ahmadi, Ehsan Ahmadpour, Rushdia Ahmed, Chalachew Genet Akal, Joshua O. Akinyemi, Fares Alahdab, Noore Alam, Genet Melak Alamene, Kefyalew Addis Alene, Mehran Alijanzadeh, Cyrus Alinia, Vahid Alipour, Syed Mohamed Aljunid, Mohammed J. Almalki, Hesham M. Al-Mekhlafi, Khalid Altirkawi, Nelson Alvis-Guzman, Adeladza Kofi Amegah, Saeed Amini, Arianna Maever Loreche Amit, Zohreh Anbari, Sofia Androudi, Mina Anjomshoa, Fereshteh Ansari, Carl Abelardo T. Antonio, Jalal Arabloo, Zohreh Arefi, Olatunde Aremu, Bahram Armoon, Amit Arora, Al Artaman, Anvar Asadi, Mehran Asadi-Aliabadi, Amir Ashraf-Ganjouei, Reza Assadi, Bahar Ataeinia, Sachin R. Atre, Beatriz Paulina Ayala Quintanilla, Martin Amogre Ayanore, Samad Azari, Ebrahim Babaee, Arefeh Babazadeh, Alaa Badawi, Soghra Bagheri, Mojtaba Bagherzadeh, Nafiseh Baheiraei, Abbas Balouchi, Aleksandra Barac, Quique Bassat, Bernhard T. Baune, Mohsen Bayati, Neeraj Bedi, Ettore Beghi, Masoud Behzadifar, Meysam Behzadifar, Yared Belete Belay, Brent Bell, Michelle L. Bell, Dessalegn Ajema Berbada, Robert S. Bernstein, Natalia V. Bhattacharjee, Suraj Bhattarai, Zulfiqar A. Bhutta, Ali Bijani, Somayeh Bohlouli, Nicholas J. K. Breitborde, Gabrielle Britton, Annie J. Browne, Sharath Burugina Nagaraja, Reinhard Busse, Zahid A. Butt, Josip Car, Rosario Cárdenas, Carlos A. Castañeda-Orjuela, Ester Cerin, Wagaye Fentahun Chanie, Pranab Chatterjee, Dinh-Toi Chu, Cyrus Cooper, Vera M. Costa, Koustuv Dalal, Lalit Dandona, Rakhi Dandona, Farah Daoud, Ahmad Daryani, Rajat Das Gupta, Ian Davis, Nicole Davis Weaver, Dragos Virgil Davitoiu, Jan-Walter De Neve, Feleke Mekonnen Demeke, Gebre Teklemariam Demoz, Kebede Deribe, Rupak Desai, Aniruddha Deshpande, Hanna Demelash Desyibelew, Sagnik Dey, Samath Dhamminda Dharmaratne, Meghnath Dhimal, Daniel Diaz, Leila Doshmangir, Andre R. Duraes, Laura Dwyer-Lindgren, Lucas Earl, Roya Ebrahimi, Soheil Ebrahimpour, Andem Effiong, Aziz Eftekhari, Elham Ehsani-Chimeh, Iman El Sayed, Maysaa El Sayed Zaki, Maha El Tantawi, Ziad El-Khatib, Mohammad Hassan Emamian, Shymaa Enany, Sharareh Eskandarieh, Oghenowede Eyawo, Maha Ezalarab, Mahbobeh Faramarzi, Mohammad Fareed, Roghiyeh Faridnia, Andre Faro, Ali Akbar Fazaeli, Mehdi Fazlzadeh, Netsanet Fentahun, Seyed-Mohammad Fereshtehnejad, João C. Fernandes, Irina Filip, Florian Fischer, Nataliya A. Foigt, Masoud Foroutan, Joel Msafiri Francis, Takeshi Fukumoto, Nancy Fullman, Silvano Gallus, Destallem Gebremedhin Gebre, Tsegaye Tewelde Gebrehiwot, Gebreamlak Gebremedhn Gebremeskel, Bradford D. Gessner, Birhanu Geta, Peter W. Gething, Reza Ghadimi, Keyghobad Ghadiri, Mahsa Ghajarzadeh, Ahmad Ghashghaee, Paramjit Singh Gill, Tiffany K. Gill, Nick Golding, Nelson G. M. Gomes, Philimon N. Gona, Sameer Vali Gopalani, Giuseppe Gorini, Bárbara Niegia Garcia Goulart, Nicholas Graetz, Felix Greaves, Manfred S. Green, Yuming Guo, Arvin Haj-Mirzaian, Arya Haj-Mirzaian, Brian James Hall, Samer Hamidi, Hamidreza Haririan, Josep Maria Haro, Milad Hasankhani, Edris Hasanpoor, Amir Hasanzadeh, Hadi Hassankhani, Hamid Yimam Hassen, Mohamed I. Hegazy, Delia Hendrie, Fatemeh Heydarpour, Thomas R. Hird, Chi Linh Hoang, Gillian Hollerich, Enayatollah Homaie Rad, Mojtaba Hoseini-Ghahfarokhi, Naznin Hossain, Mostafa Hosseini, Mehdi Hosseinzadeh, Mihaela Hostiuc, Sorin Hostiuc, Mowafa Househ, Mohamed Hsairi, Olayinka Stephen Ilesanmi, Mohammad Hasan Imani-Nasab, Usman Iqbal, Seyed Sina Naghibi Irvani, Nazrul Islam, Sheikh Mohammed Shariful Islam, Mikk Jürisson, Nader Jafari Balalami, Amir Jalali, Javad Javidnia, Achala Upendra Jayatilleke, Ensiyeh Jenabi, John S. Ji, Yash B. Jobanputra, Kimberly Johnson, Jost B. Jonas, Zahra Jorjoran Shushtari, Jacek Jerzy Jozwiak, Ali Kabir, Amaha Kahsay, Hamed Kalani, Rohollah Kalhor, Manoochehr Karami, Surendra Karki, Amir Kasaeian, Nicholas J. Kassebaum, Peter Njenga Keiyoro, Grant Rodgers Kemp, Roghayeh Khabiri, Yousef Saleh Khader, Morteza Abdullatif Khafaie, Ejaz Ahmad Khan, Junaid Khan, Muhammad Shahzeb Khan, Young-Ho Khang, Khaled Khatab, Amir Khater, Mona M. Khater, Alireza Khatony, Mohammad Khazaei, Salman Khazaei, Maryam Khazaei-Pool, Jagdish Khubchandani, Neda Kianipour, Yun Jin Kim, Ruth W. Kimokoti, Damaris K. Kinyoki, Adnan Kisa, Sezer Kisa, Tufa Kolola, Soewarta Kosen, Parvaiz A. Koul, Ai Koyanagi, Moritz U. G. Kraemer, Kewal Krishan, Kris J. Krohn, Nuworza Kugbey, G. Anil Kumar, Manasi Kumar, Pushpendra Kumar, Desmond Kuupiel, Ben Lacey, Sheetal D. Lad, Faris Hasan Lami, Anders O. Larsson, Paul H. Lee, Mostafa Leili, Aubrey J. Levine, Shanshan Li, Lee-Ling Lim, Stefan Listl, Joshua Longbottom, Jaifred Christian F. Lopez, Stefan Lorkowski, Sameh Magdeldin, Hassan Magdy Abd El Razek, Muhammed Magdy Abd El Razek, Azeem Majeed, Afshin Maleki, Reza Malekzadeh, Deborah Carvalho Malta, Abdullah A. Mamun, Navid Manafi, Ana-Laura Manda, Morteza Mansourian, Francisco Rogerlândio Martins-Melo, Anthony Masaka, Benjamin Ballard Massenburg, Pallab K. Maulik, Benjamin K. Mayala, Mohsen Mazidi, Martin McKee, Ravi Mehrotra, Kala M. Mehta, Gebrekiros Gebremichael Meles, Walter Mendoza, Ritesh G. Menezes, Atte Meretoja, Tuomo J. Meretoja, Tomislav Mestrovic, Ted R. Miller, Molly K. Miller-Petrie, Edward J. Mills, George J. Milne, G. K. Mini, Seyed Mostafa Mir, Hamed Mirjalali, Erkin M. Mirrakhimov, Efat Mohamadi, Dara K. Mohammad, Aso Mohammad Darwesh, Naser Mohammad Gholi Mezerji, Ammas Siraj Mohammed, Shafiu Mohammed, Ali H. Mokdad, Mariam Molokhia, Lorenzo Monasta, Yoshan Moodley, Mahmood Moosazadeh, Ghobad Moradi, Masoud Moradi, Yousef Moradi, Maziar Moradi-Lakeh, Mehdi Moradinazar, Paula Moraga, Lidia Morawska, Abbas Mosapour, Seyyed Meysam Mousavi, Ulrich Otto Mueller, Atalay Goshu Muluneh, Ghulam Mustafa, Behnam Nabavizadeh, Mehdi Naderi, Ahamarshan Jayaraman Nagarajan, Azin Nahvijou, Farid Najafi, Vinay Nangia, Duduzile Edith Ndwandwe, Nahid Neamati, Ionut Negoi, Ruxandra Irina Negoi, Josephine W. Ngunjiri, Huong Lan Thi Nguyen, Long Hoang Nguyen, Son Hoang Nguyen, Katie R. Nielsen, Dina Nur Anggraini Ningrum, Yirga Legesse Nirayo, Molly R. Nixon, Chukwudi A. Nnaji, Marzieh Nojomi, Mehdi Noroozi, Shirin Nosratnejad, Jean Jacques Noubiap, Soraya Nouraei Motlagh, Richard Ofori-Asenso, Felix Akpojene Ogbo, Kelechi E. Oladimeji, Andrew T. Olagunju, Meysam Olfatifar, Solomon Olum, Bolajoko Olubukunola Olusanya, Mojisola Morenike Oluwasanu, Obinna E. Onwujekwe, Eyal Oren, Doris D. V. Ortega-Altamirano, Alberto Ortiz, Osayomwanbo Osarenotor, Frank B. Osei, Aaron E. Osgood-Zimmerman, Stanislav S. Otstavnov, Mayowa Ojo Owolabi, Mahesh P. A., Abdol Sattar Pagheh, Smita Pakhale, Songhomitra Panda-Jonas, Animika Pandey, Eun-Kee Park, Hadi Parsian, Tahereh Pashaei, Sangram Kishor Patel, Veincent Christian Filipino Pepito, Alexandre Pereira, Samantha Perkins, Brandon V. Pickering, Thomas Pilgrim, Majid Pirestani, Bakhtiar Piroozi, Meghdad Pirsaheb, Oleguer Plana-Ripoll, Hadi Pourjafar, Parul Puri, Mostafa Qorbani, Hedley Quintana, Mohammad Rabiee, Navid Rabiee, Amir Radfar, Alireza Rafiei, Fakher Rahim, Zohreh Rahimi, Vafa Rahimi-Movaghar, Shadi Rahimzadeh, Fatemeh Rajati, Sree Bhushan Raju, Azra Ramezankhani, Chhabi Lal Ranabhat, Davide Rasella, Vahid Rashedi, Lal Rawal, Robert C. Reiner Jr, Andre M. N. Renzaho, Satar Rezaei, Aziz Rezapour, Seyed Mohammad Riahi, Ana Isabel Ribeiro, Leonardo Roever, Elias Merdassa Roro, Max Roser, Gholamreza Roshandel, Daem Roshani, Ali Rostami, Enrico Rubagotti, Salvatore Rubino, Siamak Sabour, Nafis Sadat, Ehsan Sadeghi, Reza Saeedi, Yahya Safari, Roya Safari-Faramani, Mahdi Safdarian, Amirhossein Sahebkar, Mohammad Reza Salahshoor, Nasir Salam, Payman Salamati, Farkhonde Salehi, Saleh Salehi Zahabi, Yahya Salimi, Hamideh Salimzadeh, Joshua A. Salomon, Evanson Zondani Sambala, Abdallah M. Samy, Milena M. Santric Milicevic, Bruno Piassi Sao Jose, Sivan Yegnanarayana Iyer Saraswathy, Rodrigo Sarmiento-Suárez, Benn Sartorius, Brijesh Sathian, Sonia Saxena, Alyssa N. Sbarra, Lauren E. Schaeffer, David C. Schwebel, Sadaf G. Sepanlou, Seyedmojtaba Seyedmousavi, Faramarz Shaahmadi, Masood Ali Shaikh, Mehran Shams-Beyranvand, Amir Shamshirian, Morteza Shamsizadeh, Kiomars Sharafi, Mehdi Sharif, Mahdi Sharif-Alhoseini, Hamid Sharifi, Jayendra Sharma, Rajesh Sharma, Aziz Sheikh, Chloe Shields, Mika Shigematsu, Rahman Shiri, Ivy Shiue, Kerem Shuval, Tariq J. Siddiqi, João Pedro Silva, Jasvinder A. Singh, Dhirendra Narain Sinha, Malede Mequanent Sisay, Solomon Sisay, Karen Sliwa, David L. Smith, Ranjani Somayaji, Moslem Soofi, Joan B. Soriano, Chandrashekhar T. Sreeramareddy, Agus Sudaryanto, Mu’awiyyah Babale Sufiyan, Bryan L. Sykes, P. N. Sylaja, Rafael Tabarés-Seisdedos, Karen M. Tabb, Takahiro Tabuchi, Nuno Taveira, Mohamad-Hani Temsah, Abdullah Sulieman Terkawi, Zemenu Tadesse Tessema, Kavumpurathu Raman Thankappan, Sathish Thirunavukkarasu, Quyen G. To, Marcos Roberto Tovani-Palone, Bach Xuan Tran, Khanh Bao Tran, Irfan Ullah, Muhammad Shariq Usman, Olalekan A. Uthman, Amir Vahedian-Azimi, Pascual R. Valdez, Job F. M. van Boven, Tommi Juhani Vasankari, Yasser Vasseghian, Yousef Veisani, Narayanaswamy Venketasubramanian, Francesco S. Violante, Sergey Konstantinovitch Vladimirov, Vasily Vlassov, Theo Vos, Giang Thu Vu, Isidora S. Vujcic, Yasir Waheed, Jon Wakefield, Haidong Wang, Yafeng Wang, Yuan-Pang Wang, Joseph L. Ward, Robert G. Weintraub, Kidu Gidey Weldegwergs, Girmay Teklay Weldesamuel, Ronny Westerman, Charles Shey Wiysonge, Dawit Zewdu Wondafrash, Lauren Woyczynski, Ai-Min Wu, Gelin Xu, Abbas Yadegar, Tomohide Yamada, Vahid Yazdi-Feyzabadi, Christopher Sabo Yilgwan, Paul Yip, Naohiro Yonemoto, Javad Yoosefi Lebni, Mustafa Z. Younis, Mahmoud Yousefifard, Hebat-Allah Salah A. Yousof, Chuanhua Yu, Hasan Yusefzadeh, Erfan Zabeh, Telma Zahirian Moghadam, Sojib Bin Zaman, Mohammad Zamani, Hamed Zandian, Alireza Zangeneh, Taddese Alemu Zerfu, Yunquan Zhang, Arash Ziapour, Sanjay Zodpey, Christopher J. L. Murray, Simon I. Hay

**Affiliations:** 1 Institute for Health Metrics and Evaluation, University of Washington, Seattle, WA USA; 2 Department of Nutrition, Tabriz University of Medical Sciences, Tabriz, Iran; 3 Kermanshah University of Medical Sciences, Kermanshah, Iran; 4 Department of Neurology, Cairo University, Cairo, Egypt; 5 Department of Parasitology and Mycology, Jahrom University of Medical Sciences, Jahrom, Iran; 6 The Institute of Pharmaceutical Sciences, Tehran University of Medical Sciences, Tehran, Iran; 7 Multiple Sclerosis Research Center, Tehran University of Medical Sciences, Tehran, Iran; 8 Department of Epidemiology, Arak University of Medical Sciences, Arak, Iran; 9 Department of Public Health, Ministry of Health, Riyadh, Saudi Arabia; 10 Research Department, Philippine Institute for Development Studies, Quezon City, The Philippines; 11 Department of Preventive Medicine, Dongguk University, Gyeongju, South Korea; 12 Department of Community Medicine, Kathmandu University, Devdaha, Nepal; 13 College of Medicine, University College Hospital, Ibadan, Nigeria; 14 School of Medicine, Cardiff University, Cardiff, UK; 15 School of Health, Ardabil University of Medical Science, Ardabil, Iran; 16 Department of Community Medicine, Zabol University of Medical Sciences, Zabol, Iran; 17 Department of Epidemiology and Biostatistics, Qom University of Medical Sciences, Qom, Iran; 18 School of Pharmacy, University of Lincoln, Lincoln, UK; 19 Environmental Technologies Research Center, Ahvaz Jundishapur University of Medical Sciences, Ahvaz, Iran; 20 Department of Parasitology and Mycology, Tabriz University of Medical Sciences, Tabriz, Iran; 21 James P. Grant School of Public Health, Brac University, Dhaka, Bangladesh; 22 Health Systems and Population Studies Division, International Centre for Diarrhoeal Disease Research Bangladesh, Dhaka, Bangladesh; 23 Department of Medical Laboratory Science, Bahir Dar University, Bahir Dar, Ethiopia; 24 Epidemiology and Medical Statistics, University of Ibadan, Ibadan, Nigeria; 25 Evidence Based Practice Center, Mayo Clinic Foundation for Medical Education and Research, Rochester, MN USA; 26 Prevention Division, Queensland Health, Herston, Queensland Australia; 27 School of Health Sciences, Madda Walabu University, Bale Goba, Ethiopia; 28 Institute of Public Health, University of Gondar, Gondar, Ethiopia; 29 Research School of Population Health, Australian National University, Canberra, Australian Capital Territory Australia; 30 Qazvin University of Medical Sciences, Qazvin, Iran; 31 Department of Health Care Management and Economics, Urmia University of Medical Science, Urmia, Iran; 32 Health Economics Department, Iran University of Medical Sciences, Tehran, Iran; 33 Health Management and Economics Research Center, Iran University of Medical Sciences, Tehran, Iran; 34 Department of Health Policy and Management, Kuwait University, Safat, Kuwait; 35 International Centre for Casemix and Clinical Coding, National University of Malaysia, Bandar Tun Razak, Malaysia; 36 Faculty of Public Health and Tropical Medicine, Jazan University, Jazan, Saudi Arabia; 37 Jazan University, Jazan, Saudi Arabia; 38 Medical Research Center, Jazan University, Jazan, Saudi Arabia; 39 Department of Medical Parasitology, Sana’a University, Sana’a, Yemen; 40 King Saud University, Riyadh, Saudi Arabia; 41 Research Group in Health Economics, Universidad de Cartagena, Cartagena, Colombia; 42 Research Group in Hospital Management and Health Policies, Universidad de la Costa, Barranquilla, Colombia; 43 Biomedical Science, University of Cape Coast, Cape Coast, Ghana; 44 Health Services Management Department, Arak University of Medical Sciences, Arak, Iran; 45 Department of Epidemiology and Biostatistics, University of the Philippines Manila, Manila, The Philippines; 46 Online Programs for Applied Learning, Johns Hopkins University, Baltimore, MD USA; 47 Department of Medicine, University of Thessaly, Volos, Greece; 48 Social Determinants of Health Research Center, Rafsanjan University of Medical Sciences, Rafsanjan, Iran; 49 Research Center for Evidence Based Medicine-Health Management and Safety Promotion Research Institute, Tabriz University of Medical Sciences, Tabriz, Iran; 50 Department of Health Policy and Administration, University of the Philippines Manila, Manila, The Philippines; 51 Department of Applied Social Sciences, Hong Kong Polytechnic University, Hong Kong, China; 52 Department of Health Promotion and Education, Tehran University of Medical Sciences, Tehran, Iran; 53 School of Health Sciences, Birmingham City University, Birmingham, UK; 54 School of Nursing and Midwifery, Saveh University of Medical Sciences, Saveh, Iran; 55 Social Determinants of Health Research Center, Saveh University of Medical Sciences, Saveh, Iran; 56 School of Science and Health, Western Sydney University, Penrith, New South Wales Australia; 57 Oral Health Services, Sydney Local Health District, Sydney, New South Wales Australia; 58 Department of Community Health Sciences, University of Manitoba, Winnipeg, Manitoba Canada; 59 Research Center for Environmental Determinants of Health, Kermanshah University of Medical Sciences, Kermanshah, Iran; 60 Preventive Medicine and Public Health Research Center, Iran University of Medical Sciences, Tehran, Iran; 61 Faculty of Medicine, Tehran University of Medical Sciences, Tehran, Iran; 62 Education Development Center, Mashhad University of Medical Sciences, Mashhad, Iran; 63 Non-communicable Diseases Research Center, Tehran University of Medical Sciences, Tehran, Iran; 64 Center for Clinical Global Health Education, Johns Hopkins University, Baltimore, MD USA; 65 Dr D. Y. Patil Medical College, Pune, India; 66 The Judith Lumley Centre, La Trobe University, Melbourne, Victoria Australia; 67 General Office for Research and Technological Transfer, Peruvian National Institute of Health, Lima, Peru; 68 Department of Family and Community Health, University of Health and Allied Sciences, Ho, Ghana; 69 Center for Infectious Diseases Research, Babol, Iran; 70 Public Health Risk Sciences Division, Public Health Agency of Canada, Toronto, Ontario Canada; 71 Department of Nutritional Sciences, University of Toronto, Toronto, Ontario Canada; 72 Department of Chemistry, Sharif University of Technology, Tehran, Iran; 73 Tissue Engineering and Applied Cell Sciences Division, Tarbiat Modares University, Tehran, Iran; 74 Division of Diseases, Advanced Technologies Research Group, Tehran, Iran; 75 School of Nursing and Midwifery, Iran University of Medical Sciences, Tehran, Iran; 76 Clinic for Infectious and Tropical Diseases, Clinical Center of Serbia, Belgrade, Serbia; 77 Faculty of Medicine, University of Belgrade, Belgrade, Serbia; 78 Barcelona Institute for Global Health, University of Barcelona, Barcelona, Spain; 79 Catalan Institution for Research and Advanced Studies (ICREA), Barcelona, Spain; 80 Department of Psychiatry, Melbourne Medical School, Melbourne, Victoria Australia; 81 Health Human Resources Research Center, Shiraz University of Medical Sciences, Shiraz, Iran; 82 Department of Community Medicine, Gandhi Medical College Bhopal, Bhopal, India; 83 Department of Neuroscience, Mario Negri Institute for Pharmacological Research, Milan, Italy; 84 Social Determinants of Health Research Center, Lorestan University of Medical Sciences, Khorramabad, Iran; 85 Hepatitis Research Center, Lorestan University of Medical Sciences, Khorramabad, Iran; 86 Pharmacoepidemiology and Social Pharmacy, Mekelle University, Mekelle, Ethiopia; 87 School of Forestry and Environmental Studies, Yale University, New Haven, CT USA; 88 Department of Public Health, Arba Minch University, Arba Minch, Ethiopia; 89 Hubert Department of Global Health, Emory University, Atlanta, GA USA; 90 Department of Global Health, University of South Florida, Tampa, FL USA; 91 London School of Hygiene & Tropical Medicine, London, UK; 92 Nepal Academy of Science & Technology, Patan, Nepal; 93 The Centre for Global Child Health, Hospital for Sick Children, University of Toronto, Toronto, Ontario Canada; 94 Center of Excellence in Women and Child Health, Aga Khan University, Karachi, Pakistan; 95 Social Determinants of Health Research Center, Babol University of Medical Sciences, Babol, Iran; 96 Department of Veterinary Medicine, Karaj Islamic Azad University, Kermanshah, Iran; 97 Department of Psychology, Ohio State University, Columbus, OH USA; 98 Psychiatry and Behavioral Health Department, Ohio State University, Columbus, OH USA; 99 Neuroscience Department, Institute for Scientific Research and High Technology Services, City of Knowledge, Panama; 100 Gorgas Memorial Institute for Health Studies, Panama, Panama; 101 Big Data Institute, Li Ka Shing Centre for Health Information and Discovery, University of Oxford, Oxford, UK; 102 Department of Community Medicine, Employees’ State Insurance Model Hospital, Bangalore, India; 103 Department for Health Care Management, Technical University of Berlin, Berlin, Germany; 104 School of Population and Public Health, University of British Columbia, Vancouver, British Columbia Canada; 105 Al Shifa School of Public Health, Al Shifa Trust Eye Hospital, Rawalpindi, Pakistan; 106 Centre for Population Health Sciences, Nanyang Technological University, Singapore, Singapore; 107 Global Ehealth Unit, Imperial College London, London, UK; 108 Department of Population and Health, Metropolitan Autonomous University, Mexico City, Mexico; 109 Colombian National Health Observatory, National Institute of Health, Bogota, Colombia; 110 Epidemiology and Public Health Evaluation Group, National University of Colombia, Bogota, Colombia; 111 Mary Mackillop Institute for Health Research, Australian Catholic University, Melbourne, Victoria Australia; 112 School of Public Health, University of Hong Kong, Hong Kong, China; 113 Department of Obstetrics and Gynaecology, University of Gondar, Gondar, Ethiopia; 114 Division of Epidemiology, National Institute of Cholera and Enteric Diseases, Kolkata, India; 115 Faculty of Biology, Hanoi National University of Education, Hanoi, Vietnam; 116 Department of Rheumatology, University of Oxford, Oxford, UK; 117 Medical Research Council Lifecourse Epidemiology Unit, University of Southampton, Southampton, UK; 118 Applied Molecular Biosciences Unit (UCIBIO), University of Porto, Porto, Portugal; 119 Institute of Public Health Kalyani, Kalyani, India; 120 School of Health Science, Orebro University, Orebro, Sweden; 121 Public Health Foundation of India, Gurugram, India; 122 Toxoplasmosis Research Center, Mazandaran University of Medical Sciences, Sari, Iran; 123 Department of General Surgery, Carol Davila University of Medicine and Pharmacy, Bucharest, Romania; 124 Department of Surgery, Clinical Emergency Hospital St Pantelimon, Bucharest, Romania; 125 Heidelberg Institute of Global Health (HIGH), Heidelberg University, Heidelberg, Germany; 126 Bahir Dar University, Bahir Dar, Ethiopia; 127 School of Pharmacy, Aksum University, Aksum, Ethiopia; 128 Addis Ababa University, Addis Ababa, Ethiopia; 129 School of Public Health, Addis Ababa University, Addis Ababa, Ethiopia; 130 Department of Global Health and Infection, Brighton and Sussex Medical School, Brighton, UK; 131 Division of Cardiology, Atlanta Veterans Affairs Medical Center, Decatur, GA USA; 132 Public Health Nutrition, Bahir Dar University, Bahir Dar, Ethiopia; 133 Centre for Atmospheric Sciences, Indian Institute of Technology Delhi, New Delhi, India; 134 Department of Community Medicine, University of Peradeniya, Peradeniya, Sri Lanka; 135 Health Research Section, Nepal Health Research Council, Kathmandu, Nepal; 136 Center of Complexity Sciences, National Autonomous University of Mexico, Mexico City, Mexico; 137 Facultad de Medicina Veterinaria y Zootecnia, Autonomous University of Sinaloa, Culiacan Rosales, Mexico; 138 Department of Health Policy and Economy, Tabriz University of Medical Sciences, Tabriz, Iran; 139 School of Medicine, Federal University of Bahia, Salvador, Brazil; 140 Diretoria Médica, Roberto Santos General Hospital, Salvador, Brazil; 141 Department of Health Metrics Sciences, School of Medicine, University of Washington, Seattle, WA USA; 142 Environmental Health Research Center, Kurdistan University of Medical Sciences, Sanandaj, Iran; 143 Clinical Epidemiology and Biostatistics, University of Newcastle, Newcastle, New South Wales Australia; 144 Department of Toxicology and Pharmacology, Tabriz University of Medical Sciences, Tabriz, Iran; 145 Department of Basic Sciences, Maragheh University of Medical Sciences, Maragheh, Iran; 146 National Institute for Health Researchers, Tehran University of Medical Sciences, Tehran, Iran; 147 Medical Research Institute, Alexandria University, Alexandria, Egypt; 148 Department of Clinical Pathology, Mansoura University, Mansoura, Egypt; 149 Pediatric Dentistry and Dental Public Health, Alexandria University, Alexandria, Egypt; 150 Preventive Dental Sciences, Imam Abdulrahman Bin Faisal University, Dammam, Saudi Arabia; 151 Department of Public Health Sciences, Karolinska Institutet, Stockholm, Sweden; 152 Ophthalmic Epidemiology Research Center, Shahroud University of Medical Sciences, Shahroud, Iran; 153 Department of Microbiology and Immunology, Suez Canal University, Ismailia, Egypt; 154 Epidemiology and Population Health, British Columbia Centre for Excellence in HIV/AIDS, Vancouver, British Columbia Canada; 155 Faculty of Health Sciences, Simon Fraser University, Burnaby, British Columbia Canada; 156 Babol University of Medical Sciences, Babol, Iran; 157 College of Medicine, Imam Muhammad Ibn Saud Islamic University, Riyadh, Saudi Arabia; 158 Department of Parasitology, Mazandaran University of Medical Sciences, Sari, Iran; 159 Department of Psychology, Federal University of Sergipe, Sao Cristovao, Brazil; 160 Social Determinants of Health Research Center, Hamadan University of Medical Sciences, Hamadan, Iran; 161 Environmental Health Engineering, Tehran University of Medical Sciences, Tehran, Iran; 162 Department of Environmental Health Engineering, Ardabil University of Medical Science, Ardabil, Iran; 163 Department of Public Health Nutrition, Bahir Dar University, Bahir Dar, Ethiopia; 164 Department of Neurobiology, Karolinska Institutet, Stockholm, Sweden; 165 Division of Neurology, University of Ottawa, Ottawa, Ontario Canada; 166 Center for Biotechnology and Fine Chemistry, Catholic University of Portugal, Porto, Portugal; 167 Psychiatry Department, Kaiser Permanente, Fontana, CA USA; 168 Department of Health Sciences, A.T. Still University, Mesa, AZ USA; 169 Department of Public Health Medicine, Bielefeld University, Bielefeld, Germany; 170 Institute of Gerontology, National Academy of Medical Sciences of Ukraine, Kyiv, Ukraine; 171 Abadan School of Medical Sciences, Abadan, Iran; 172 Clinical Medicine and Wits Reproductive Health and HIV Institute, University of the Witwatersrand, Johannesburg, South Africa; 173 Gene Expression & Regulation Program, Cancer Institute (W.I.A.), Philadelphia, PA USA; 174 Department of Dermatology, Kobe University, Kobe, Japan; 175 Department of Environmental Health Science, Mario Negri Institute for Pharmacological Research, Milan, Italy; 176 Mekelle University, Mekelle, Ethiopia; 177 Dr Tewelde Legesse Health Sciences College, Mekelle, Ethiopia; 178 Department of Epidemiology, Jimma University, Jimma, Ethiopia; 179 School of Nursing, Mekelle University, Mekelle, Ethiopia; 180 Nursing Department, Aksum University, Aksum, Ethiopia; 181 Vaccines Department, Pfizer, Collegeville, PA USA; 182 Agency of Preventive Medicine, Paris, France; 183 Department of Pharmacy, Wollo University, Dessie, Ethiopia; 184 Health Research Institute, Babol University of Medical Sciences, Babol, Iran; 185 Department of Neurology, Tehran University of Medical Sciences, Tehran, Iran; 186 Department of Health Services Management, Iran University of Medical Sciences, Tehran, Iran; 187 Unit of Academic Primary Care, University of Warwick, Coventry, UK; 188 Adelaide Medical School, University of Adelaide, Adelaide, South Australia Australia; 189 Department of Chemistry, University of Porto, Porto, Portugal; 190 REQUIMTE/LAQV, Porto, Portugal; 191 Nursing and Health Sciences Department, University of Massachusetts Boston, Boston, MA USA; 192 Department of Biostatistics and Epidemiology, University of Oklahoma, Oklahoma City, OK USA; 193 Department of Health and Social Affairs, Government of the Federated States of Micronesia, Palikir, Federated States of Micronesia; 194 Occupational and Environmental Epidemiology Section, Cancer Prevention and Research Institute, Florence, Italy; 195 Postgraduate Program in Epidemiology, Federal University of Rio Grande do Sul, Porto Alegre, Brazil; 196 Department of Primary Care and Public Health, Imperial College London, London, UK; 197 Health Improvement Directorate, Public Health England, London, UK; 198 School of Public Health, University of Haifa, Haifa, Israel; 199 School of Public Health and Preventive Medicine, Monash University, Melbourne, Victoria Australia; 200 Department of Epidemiology and Biostatistics, Zhengzhou University, Zhengzhou, China; 201 Department of Pharmacology, Tehran University of Medical Sciences, Tehran, Iran; 202 Obesity Research Center, Research Institute for Endocrine Sciences, Shahid Beheshti University of Medical Sciences, Tehran, Iran; 203 Department of Radiology, Johns Hopkins University, Baltimore, MD USA; 204 Global and Community Mental Health Research Group, University of Macau, Macao, China; 205 School of Health and Environmental Studies, Hamdan Bin Mohammed Smart University, Dubai, United Arab Emirates; 206 Tabriz University of Medical Sciences, Tabriz, Iran; 207 Biomedical Research Networking Center for Mental Health Network (CIBERSAM), Madrid, Spain; 208 Research and Development Unit, San Juan de Dios Sanitary Park, Sant Boi De Llobregat, Spain; 209 School of Nutrition and Food Sciences, Tabriz University of Medical Sciences, Tabriz, Iran; 210 Healthcare Management, Maragheh University of Medical Sciences, Maragheh, Iran; 211 Department of Microbiology, Tehran University of Medical Sciences, Tehran, Iran; 212 Department of Microbiology, Maragheh University of Medical Sciences, Maragheh, Iran; 213 School of Nursing and Midwifery Tabriz University of Medical Sciences, Tabriz, Iran; 214 Independent Consultant, Tabriz, Iran; 215 Public Health Department, Mizan-Tepi University, Teppi, Ethiopia; 216 Unit of Epidemiology and Social Medicine, University Hospital Antwerp, Antwerp, Belgium; 217 School of Public Health, Curtin University, Bentley, Western Australia Australia; 218 Medical Biology Research Center, Kermanshah University of Medical Sciences, Kermanshah, Iran; 219 Population Health, Baker Heart and Diabetes Institute, Melbourne, Victoria Australia; 220 Center of Excellence in Behavioral Medicine, Nguyen Tat Thanh University, Ho Chi Minh, Vietnam; 221 Social Determinants of Health Research Center, Guilan Road Trauma Research Center, Guilan University of Medical Sciences, Rasht, Iran; 222 Guilan Road Trauma Research Center, Guilan University of Medical Sciences, Rasht, Iran; 223 Radiology and Nuclear Medicine Department, Kermanshah University of Medical Sciences, Kermanshah, Iran; 224 Department of Pharmacology and Therapeutics, University of Dhaka, Dhaka, Bangladesh; 225 Department of Epidemiology and Biostatistics, Tehran University of Medical Sciences, Tehran, Iran; 226 Computer Science Department, University of Human Development, Sulaimaniyah, Iraq; 227 Department of Internal Medicine, Bucharest Emergency Hospital, Bucharest, Romania; 228 Faculty of Dentistry, Carol Davila University of Medicine and Pharmacy, Bucharest, Romania; 229 Clinical Legal Medicine, National Institute of Legal Medicine Mina Minovici, Bucharest, Romania; 230 Division of Information and Computing Technology, Hamad Bin Khalifa University, Doha, Qatar; 231 Qatar Foundation for Education, Science and Community Development, Doha, Qatar; 232 Faculty of Medicine Tunis, Medicine School of Tunis, Baab Saadoun, Tunisia; 233 Department of Community Medicine, University of Ibadan, Ibadan, Nigeria; 234 Department of Public Health, Lorestan University of Medical Sciences, Khorramabad, Iran; 235 Global Health and Development Department, Taipei Medical University, Taipei City, Taiwan; 236 Research Institute for Endocrine Sciences, Shahid Beheshti University of Medical Sciences, Tehran, Iran; 237 MRC Epidemiology Unit, University of Cambridge, Cambridge, UK; 238 Harvard University, Boston, MA USA; 239 Institute for Physical Activity and Nutrition, Deakin University, Burwood, Victoria Australia; 240 Sydney Medical School, University of Sydney, Sydney, New South Wales Australia; 241 Institute of Family Medicine and Public Health, University of Tartu, Tartu, Estonia; 242 Psychosis Department, Babol Nushirvani University of Technology, Babol, Iran; 243 Psychiatric Department, Kermanshah University of Medical Sciences, Kermanshah, Iran; 244 Department of Medical Mycology, Mazandaran University of Medical Sciences, Sari, Iran; 245 Faculty of Graduate Studies, University of Colombo, Colombo, Sri Lanka; 246 Institute of Medicine, University of Colombo, Colombo, Sri Lanka; 247 School of Midwifery, A.T. Still University, Mesa, AZ USA; 248 Environmental Research Center, Duke Kunshan University, Kunshan, China; 249 Department of Medicine, University of Miami, Atlantis, FL USA; 250 Department of Ophthalmology, Heidelberg University, Heidelberg, Germany; 251 Beijing Institute of Ophthalmology, Beijing Tongren Hospital, Beijing, China; 252 Social Determinants of Health Research Center, University of Social Welfare and Rehabilitation Sciences, Tehran, Iran; 253 Faculty of Medicine and Health Sciences, University of Opole, Opole, Poland; 254 Department of Family Medicine and Public Health, University of Opole, Opole, Poland; 255 Minimally Invasive Surgery Research Center, Iran University of Medical Sciences, Tehran, Iran; 256 Department of Nutrition and Dietetics, Mekelle University, Mekelle, Ethiopia; 257 Mazandaran University of Medical Sciences, Sari, Iran; 258 Isfahan University of Medical Sciences, Isfahan, Iran; 259 Social Determinants of Health Research Center, Qazvin University of Medical Sciences, Qazvin, Iran; 260 Department of Epidemiology, Hamadan University of Medical Sciences, Hamadan, Iran; 261 Research and Development, Australian Red Cross Blood Service, Sydney, New South Wales Australia; 262 School of Public Health and Community Medicine, University of New South Wales, Sydney, New South Wales Australia; 263 Hematologic Malignancies Research Center, Tehran University of Medical Sciences, Tehran, Iran; 264 Hematology-Oncology and Stem Cell Transplantation Research Center, Tehran University of Medical Sciences, Tehran, Iran; 265 Department of Anesthesiology & Pain Medicine, University of Washington, Seattle, WA USA; 266 Odel Campus, University of Nairobi, Nairobi, Kenya; 267 Michigan State University, East Lansing, MI USA; 268 Tabriz Health Management Research Center, Tabriz University of Medical Sciences, Tabriz, Iran; 269 National Institute for Health Research (NIHR), Tehran University of Medical Sciences, Tehran, Iran; 270 Department of Public Health and Community Medicine, Jordan University of Science and Technology, Ramtha, Jordan; 271 Social Determinants of Health Research Center, Ahvaz Jundishapur University of Medical Sciences, Ahvaz, Iran; 272 Epidemiology and Biostatistics Department, Health Services Academy, Islamabad, Pakistan; 273 Population Studies, International Institute for Population Sciences, Mumbai, India; 274 Department of Internal Medicine, John H. Stroger Jr Hospital of Cook County, Chicago, IL USA; 275 Department of Internal Medicine, Dow University of Health Sciences, Karachi, Pakistan; 276 Institute of Health Policy and Management, Seoul National University, Seoul, South Korea; 277 Department of Health Policy and Management, Seoul National University, Seoul, South Korea; 278 Faculty of Health and Wellbeing, Sheffield Hallam University, Sheffield, UK; 279 Department of Arts and Sciences, Ohio University, Zanesville, OH USA; 280 Internal Medicine and Gastroenterology Department, National Hepatology and Tropical Research Institute, Cairo, Egypt; 281 Department of Medical Parasitology, Cairo University, Cairo, Egypt; 282 Department of Environmental Health Engineering, Hamadan University of Medical Sciences, Hamadan, Iran; 283 Department of Public Health, Mazandaran University of Medical Sciences, Sari, Iran; 284 Department of Nutrition and Health Science, Ball State University, Muncie, IN USA; 285 School of Public Health, Kermanshah University of Medical Sciences, Kermanshah, Iran; 286 School of Medicine, Xiamen University Malaysia, Sepang, Malaysia; 287 Department of Nutrition, Simmons College, Boston, MA USA; 288 Department of Health Management and Health Economics, Kristiania University College, Oslo, Norway; 289 Department of Health Services Policy and Management, University of South Carolina, Columbia, SC USA; 290 Nursing and Health Promotion, Oslo Metropolitan University, Oslo, Norway; 291 Department of Public Health, Debre Berhan University, Debre Berhan, Ethiopia; 292 Independent Consultant, Jakarta, Indonesia; 293 Department of Internal and Pulmonary Medicine, Sheri Kashmir Institute of Medical Sciences, Srinagar, India; 294 CIBERSAM, San Juan de Dios Sanitary Park, Sant Boi De Llobregat, Spain; 295 Department of Zoology, University of Oxford, Oxford, UK; 296 Medical School, Harvard University, Boston, MA USA; 297 Department of Anthropology, Panjab University, Chandigarh, India; 298 Family and Community Health, University of Health and Allied Sciences, Ho, Ghana; 299 Psychology and Health Promotion, University of Kwazulu-Natal, Durban, South Africa; 300 Department of Psychiatry, University of Nairobi, Nairobi, Kenya; 301 Department of Psychology, University College London, London, UK; 302 International Institute for Population Sciences, Mumbai, India; 303 Department of Public Health Medicine, University of Kwazulu-Natal, Durban, South Africa; 304 Nursing, St John of God Hospital, Duayaw Nkwanta, Ghana; 305 Nuffield Department of Population Health, University of Oxford, Oxford, UK; 306 Oxford Biomedical Research Centre, National Institute for Health Research (NIHR), Oxford, UK; 307 Department of Pediatrics, Post Graduate Institute of Medical Education and Research, Chandigarh, India; 308 Department of Community and Family Medicine, Academy of Medical Science, Baghdad, Iraq; 309 Department of Medical Sciences, Uppsala University, Uppsala, Sweden; 310 Department of Clinical Chemistry and Pharmacology, Uppsala University Hospital, Uppsala, Sweden; 311 School of Nursing, Hong Kong Polytechnic University, Hong Kong, China; 312 Department of Medicine, University of Malaya, Kuala Lumpur, Malaysia; 313 Department of Medicine and Therapeutics, The Chinese University of Hong Kong, Shatin, China; 314 Department of Dentistry, Radboud University, Nijmegen, The Netherlands; 315 Section for Translational Health Economics, Heidelberg University Hospital, Heidelberg, Germany; 316 Department of Vector Biology, Liverpool School of Tropical Medicine, Liverpool, UK; 317 Alliance for Improving Health Outcomes, Quezon City, The Philippines; 318 Institute of Nutrition, Friedrich Schiller University Jena, Jena, Germany; 319 Competence Cluster for Nutrition and Cardiovascular Health (NUTRICARD), Jena, Germany; 320 Physiology Department, Suez Canal University, Ismailia, Egypt; 321 Proteomics and Metabolomics Unit, Suez Canal University, Ismailia, Egypt; 322 Department of Cardiology, Damietta University, Damietta, Egypt; 323 Ophthalmology Department, Aswan Faculty of Medicine, Aswan, Egypt; 324 Digestive Diseases Research Institute, Tehran University of Medical Sciences, Tehran, Iran; 325 Non-communicable Diseases Research Center, Shiraz University of Medical Sciences, Shiraz, Iran; 326 Department of Maternal and Child Nursing and Public Health, Federal University of Minas Gerais, Belo Horizonte, Brazil; 327 Institute for Social Science Research, The University of Queensland, Brisbane, Queensland Australia; 328 Ophthalmology Department, Iran University of Medical Sciences, Tehran, Iran; 329 Department Ophthalmology, University of Manitoba, Winnipeg, Manitoba Canada; 330 Surgery Department, Emergency University Hospital Bucharest, Bucharest, Romania; 331 Department of Health Education and Health Promotion, Iran University of Medical Sciences, Tehran, Iran; 332 Campus Caucaia, Federal Institute of Education, Science and Technology of Ceará, Caucaia, Brazil; 333 Faculty of Health and Education, Botho University-Botswana, Gaborone, Botswana; 334 Division of Plastic Surgery, University of Washington, Seattle, WA USA; 335 School of Medicine, University of New South Wales, Sydney, New South Wales Australia; 336 Research Department, The George Institute for Global Health, New Delhi, India; 337 Department of Biology and Biological Engineering, Chalmers University of Technology, Gothenburg, Sweden; 338 Department of Health Services Research and Policy, London School of Hygiene & Tropical Medicine, London, UK; 339 Preventive Oncology Department, National Institute of Cancer Prevention and Research, Noida, India; 340 Department of Epidemiology and Biostatistics, University of California San Francisco, San Francisco, CA USA; 341 Peru Country Office, United Nations Population Fund (UNFPA), Lima, Peru; 342 Forensic Medicine Division, Imam Abdulrahman Bin Faisal University, Dammam, Saudi Arabia; 343 Neurocenter, Helsinki University Hospital, Helsinki, Finland; 344 School of Health Sciences, University of Melbourne, Parkville, Victoria Australia; 345 Breast Surgery Unit, Helsinki University Hospital, Helsinki, Finland; 346 University of Helsinki, Helsinki, Finland; 347 Clinical Microbiology and Parasitology Unit, Dr Zora Profozic Polyclinic, Zagreb, Croatia; 348 University Centre Varazdin, University North, Varazdin, Croatia; 349 Pacific Institute for Research & Evaluation, Calverton, MD USA; 350 Health, Evidence and Impact, McMaster University, Hamilton, Ontario Canada; 351 Department of Computer Science and Software Engineering, University of Western Australia, Perth, Western Australia Australia; 352 Department of Public Health, Amrita Institute of Medical Sciences, Kochi, India; 353 Department of Clinical Biochemistry, Babol University of Medical Sciences, Babol, Iran; 354 Golestan University of Medical Sciences, Gorgan, Iran; 355 Foodborne and Waterborne Diseases Research Center, Shahid Beheshti University of Medical Sciences, Tehran, Iran; 356 Faculty of General Medicine, Kyrgyz State Medical Academy, Bishkek, Kyrgyzstan; 357 Department of Atherosclerosis and Coronary Heart Disease, National Center of Cardiology and Internal Disease, Bishkek, Kyrgyzstan; 358 Health Equity Research Center, Tehran University of Medical Sciences, Tehran, Iran; 359 Department of Medicine Huddinge, Karolinska Institutet, Stockholm, Sweden; 360 Department of Food Technology, College of Agriculture, Salahaddin University-Erbil, Erbil, Iraq; 361 Information Technology Department, University of Human Development, Sulaimaniyah, Iraq; 362 Department of Biostatistics, Hamadan University of Medical Sciences, Hamadan, Iran; 363 School of Pharmacy, Haramaya University, Harar, Ethiopia; 364 Institute of Public Health, Heidelberg University, Heidelberg, Germany; 365 Health Systems and Policy Research Unit, Ahmadu Bello University, Zaria, Nigeria; 366 Faculty of Life Sciences and Medicine, King’s College London, London, UK; 367 Clinical Epidemiology and Public Health Research Unit, Burlo Garofolo Institute for Maternal and Child Health, Trieste, Italy; 368 Health Sciences Research Center, Mazandaran University of Medical Sciences, Sari, Iran; 369 Department of Epidemiology and Biostatistics, Kurdistan University of Medical Sciences, Sanandaj, Iran; 370 Social Determinants of Health Research Center, Kurdistan University of Medical Sciences, Sanandaj, Iran; 371 Department of Epidemiology, Iran University of Medical Sciences, Tehran, Iran; 372 Department of Mathematical Sciences, University of Bath, Bath, UK; 373 International Laboratory for Air Quality and Health, Queensland University of Technology, Brisbane, Queensland Australia; 374 Department of Clinical Biochemistry, Tarbiat Modares University, Tehran, Iran; 375 Department of Health Management and Economics, Tehran University of Medical Sciences, Tehran, Iran; 376 Federal Institute for Population Research, Wiesbaden, Germany; 377 Center for Population and Health, Wiesbaden, Germany; 378 Department of Epidemiology and Biostatistics, University of Gondar, Gondar, Ethiopia; 379 Department of Pediatric Medicine, Nishtar Medical University, Multan, Pakistan; 380 Department of Pediatrics & Pediatric Pulmonology, Institute of Mother & Child Care, Multan, Pakistan; 381 Department of Urology, Tehran University of Medical Sciences, Tehran, Iran; 382 Operating Room Department, Kermanshah University of Medical Sciences, Kermanshah, Iran; 383 Research and Analytics, Initiative for Financing Health and Human Development, Chennai, India; 384 Research and Analytics, Bioinsilico Technologies, Chennai, India; 385 Cancer Research Center of Cancer Institute, Tehran University of Medical Sciences, Tehran, Iran; 386 Department of Epidemiology & Biostatistics, Kermanshah University of Medical Sciences, Kermanshah, Iran; 387 Suraj Eye Institute, Nagpur, India; 388 Cochrane South Africa, South African Medical Research Council, Cape Town, South Africa; 389 Emergency Hospital of Bucharest, Carol Davila University of Medicine and Pharmacy, Bucharest, Romania; 390 General Surgery Department, Carol Davila University of Medicine and Pharmacy, Bucharest, Romania; 391 Anatomy and Embryology Department, Carol Davila University of Medicine and Pharmacy, Bucharest, Romania; 392 Department of Cardiology, Cardio-aid, Bucharest, Romania; 393 Department of Biological Sciences, University of Embu, Embu, Kenya; 394 Institute for Global Health Innovations, Duy Tan University, Hanoi, Vietnam; 395 Center for Excellence in Behavioral Health, Nguyen Tat Thanh University, Ho Chi Minh, Vietnam; 396 Global Health Department, University of Washington, Seattle, WA USA; 397 Department of Pediatrics, University of Washington, Seattle, WA USA; 398 Clinical Pharmacy Unit, Mekelle University, Mekelle, Ethiopia; 399 Public Health Science Department, School of Public Health and Family Medicine, University of Cape Town, Cape Town, South Africa; 400 Department of Community and Family Medicine, Iran University of Medical Sciences, Tehran, Iran; 401 University of Social Welfare and Rehabilitation Sciences, Tehran, Iran; 402 Department of Health Economics, Tabriz University of Medical Sciences, Tabriz, Iran; 403 Department of Medicine, University of Cape Town, Cape Town, South Africa; 404 Centre of Cardiovascular Research and Education in Therapeutics, Monash University, Melbourne, Victoria Australia; 405 Independent Consultant, Accra, Ghana; 406 Translational Health Research Institute, Western Sydney University, Penrith, New South Wales Australia; 407 Center for the Aid Program of Research in South Africa (CAPRISA) TB and HIV Pathogenesis Unit, United Nations Programme on HIV/AIDS (UNAIDS), Durban, South Africa; 408 Department of Psychiatry and Behavioural Neurosciences, McMaster University, Hamilton, Ontario Canada; 409 Department of Psychiatry, University of Lagos, Lagos, Nigeria; 410 Gastroenterology and Liver Disease Research Center, A.C.S. Medical College and Hospital, Tehran, Iran; 411 Department of Food Science and Postharvest Technology, Gulu University, Gulu, Uganda; 412 Ghent University, Ghent, Belgium; 413 Centre for Healthy Start Initiative, Lagos, Nigeria; 414 Department of Health Promotion and Education, University of Ibadan, Ibadan, Nigeria; 415 Department of Pharmacology and Therapeutics, University of Nigeria Nsukka, Enugu, Nigeria; 416 University of Washington, Seattle, WA USA; 417 Graduate School of Public Health, San Diego State University, San Diego, CA USA; 418 Center for Health Systems Research, National Institute of Public Health, Cuernavaca, Mexico; 419 School of Medicine, Autonomous University of Madrid, Madrid, Spain; 420 Department of Nephrology and Hypertension, The Institute for Health Research Foundation Jiménez Díaz University Hospital, Madrid, Spain; 421 Environmental Management and Toxicology, University of Benin, Benin City, Nigeria; 422 Faculty of Geoinformation Science and Earth Observation, University of Twente, Enschede, The Netherlands; 423 Department of Mathematics and Statistics, University of Energy and Natural Resources, Sunyani, Ghana; 424 Analytical Center, Moscow Institute of Physics and Technology, Dolgoprudny, Russia; 425 Committee for the Comprehensive Assessment of Medical Devices and Information Technology, Health Technology Assessment Association, Moscow, Russia; 426 Institute for Advanced Medical Research and Training, University of Ibadan, Ibadan, Nigeria; 427 Department of Tb & Respiratory Medicine, Jagadguru Sri Shivarathreeswara University, Mysore, India; 428 Department of Medicine, Ottawa Hospital Research Institute, Ottawa, Ontario Canada; 429 Heidelberg University, Heidelberg, Germany; 430 Department of Medical Humanities and Social Medicine, Kosin University, Busan, South Korea; 431 Research and Evaluation, Population Council, New Delhi, India; 432 Indian Institute of Health Management Research University, Jaipur, India; 433 Center for Research and Innovation, Ateneo De Manila University, Pasig City, The Philippines; 434 Department of Genetics, Harvard University, Boston, MA USA; 435 Laboratory of Genetics and Molecular Cardiology, University of São Paulo, Sao Paulo, Brazil; 436 Department of Cardiology, University of Bern, Bern, Switzerland; 437 Parasitology and Entomology Department, Tarbiat Modares University, Tehran, Iran; 438 National Centre for Register-Based Research, Aarhus University, Aarhus, Denmark; 439 Department of Nutrition and Food Sciences, Maragheh University of Medical Sciences, Maragheh, Iran; 440 Department of Public Health, Maragheh University of Medical Sciences, Maragheh, Iran; 441 Non-communicable Diseases Research Center, Alborz University of Medical Sciences, Karaj, Iran; 442 Biomedical Engineering, Amirkabir University of Technology, Tehran, Iran; 443 College of Graduate Health Sciences, A.T. Still University, Mesa, AZ USA; 444 Medichem, Barcelona, Spain; 445 Molecular and Cell Biology Research Center, Mazandaran University of Medical Sciences, Sari, Iran; 446 Department of Immunology, Mazandaran University of Medical Sciences, Sari, Iran; 447 Endocrinology and Metabolism Molecular-Cellular Sciences Institute, Tehran University of Medical Sciences, Tehran, Iran; 448 Thalassemia and Hemoglobinopathy Research Center, Ahvaz Jundishapur University of Medical Sciences, Ahvaz, Iran; 449 Department of Clinical Biochemistry, Kermanshah University of Medical Sciences, Kermanshah, Iran; 450 Sina Trauma and Surgery Research Center, Tehran University of Medical Sciences, Tehran, Iran; 451 Department of Health Education & Promotion, Kermanshah University of Medical Sciences, Kermanshah, Iran; 452 Department of Nephrology, Nizam’s Institute of Medical Sciences, Hyderabad, India; 453 Prevention of Metabolic Disorders Research Center, Shahid Beheshti University of Medical Sciences, Tehran, Iran; 454 Critical Care Quality Improvement Research Center, Shahid Beheshti University of Medical Sciences, Tehran, Iran; 455 Policy Research Institute, Kathmandu, Nepal; 456 Institute for Poverty Alleviation and International Development, Yonsei University, Wonju, South Korea; 457 Institute of Public Health, Federal University of Bahia, Salvador, Brazil; 458 Gonçalo Moniz Institute, Oswaldo Cruz Foundation, Salvador, Brazil; 459 School of Behavioral Sciences and Mental Health, Iran University of Medical Sciences, Tehran, Iran; 460 Social Science and Psychology, Western Sydney University, Penrith, New South Wales Australia; 461 School of Social Sciences and Psychology, Western Sydney University, Penrith, New South Wales Australia; 462 Research Center for Environmental Determinants of Health (RCEDH), Kermanshah University of Medical Sciences, Kermanshah, Iran; 463 Department of Epidemiology, Shahid Beheshti University of Medical Sciences, Tehran, Iran; 464 Department of Epidemiology, Birjand University of Medical Sciences, Birjand, Iran; 465 EPIUnit, University of Porto, Porto, Portugal; 466 Department of Clinical Research, Federal University of Uberlândia, Uberlândia, Brazil; 467 Public Health, Addis Ababa University, Addis Ababa, Ethiopia; 468 Department of Public Health, Wollega University, Nekemte, Ethiopia; 469 Martin School, University of Oxford, Oxford, UK; 470 Golestan Research Center of Gastroenterology and Hepatology, Golestan University of Medical Sciences, Gorgan, Iran; 471 Epidemiology and Biostatistics, Kurdistan University of Medical Sciences, Sanandaj, Iran; 472 Infectious Diseases and Tropical Medicine Research Center, Babol University of Medical Sciences, Babol, Iran; 473 School of Biotechnology, Ikiam Amazon Regional University, Tena, Ecuador; 474 Department of Ocean Science and Engineering, Southern University of Science and Technology, Shenzhen, China; 475 Department of Biomedical Sciences, University of Sassari, Sassari, Italy; 476 Department of Health, Safety and Environment (HSE), Shahid Beheshti University of Medical Sciences, Tehran, Iran; 477 Faculty of Public Health, Kermanshah University of Medical Sciences, Kermanshah, Iran; 478 Department of Neuroscience, Iran University of Medical Sciences, Tehran, Iran; 479 Neurogenic Inflammation Research Center, Mashhad University of Medical Sciences, Mashhad, Iran; 480 Biotechnology Research Center, Mashhad University of Medical Sciences, Mashhad, Iran; 481 Department of Anatomical Sciences, Kermanshah University of Medical Sciences, Kermanshah, Iran; 482 Department of Pathology, Al-Imam Mohammad Ibn Saud Islamic University, Riyadh, Saudi Arabia; 483 School of Health and Policy Management, Faculty of Health, York University, Toronto, Ontario Canada; 484 Taleghani Hospital, Kermanshah University of Medical Sciences, Kermanshah, Iran; 485 Department of Radiology and Nuclear Medicine, Kermanshah University of Medical Sciences, Kermanshah, Iran; 486 Taleghani Hospital, Kermanshah, Iran; 487 Center for Health Policy & Center for Primary Care and Outcomes Research, Stanford University, Stanford, CA USA; 488 Department of Entomology, Ain Shams University, Cairo, Egypt; 489 Centre School of Public Health and Health Management, University of Belgrade, Belgrade, Serbia; 490 Post-graduate Program in Infectious Diseases and Tropical Medicine, Federal University of Minas Gerais, Belo Horizonte, Brazil; 491 Department of Community Medicine, PSG Institute of Medical Sciences and Research, Coimbatore, India; 492 PSG-FAIMER South Asia Regional Institute, Coimbatore, India; 493 Department of Health and Society, Faculty of Medicine, University of Applied and Environmental Sciences, Bogotá, Colombia; 494 Faculty of Infectious and Tropical Diseases, London School of Hygiene & Tropical Medicine, London, UK; 495 Surgery Department, Hamad Medical Corporation, Doha, Qatar; 496 Faculty of Health & Social Sciences, Bournemouth University, Bournemouth, UK; 497 School of Public Health, Imperial College London, London, UK; 498 Department of Psychology, University of Alabama at Birmingham, Birmingham, AL USA; 499 Center of Expertise in Microbiology, Tehran University of Medical Sciences, Tehran, Iran; 500 Invasive Fungi Research Center, Mazandaran University of Medical Sciences, Sari, Iran; 501 Department of Health Promotion and Education, Alborz University of Medical Sciences, Karaj, Iran; 502 Independent Consultant, Karachi, Pakistan; 503 School of Medicine, Dezful University of Medical Sciences, Dezful, Iran; 504 Medical Laboratory Sciences, Mazandaran University of Medical Sciences, Sari, Iran; 505 Chronic Diseases (Home Care) Research Center, Hamadan University of Medical Sciences, Hamadan, Iran; 506 Department of Laboratory Sciences, Karaj Islamic Azad University, Kermanshah, Iran; 507 Department of Basic Sciences, Karaj Islamic Azad University, Kermanshah, Iran; 508 HIV/STI Surveillance Research Center, Kerman University of Medical Sciences, Kerman, Iran; 509 Policy and Planning Division, Ministry of Health, Riyadh, Saudi Arabia; 510 University School of Management and Entrepreneurship, Delhi Technological University, New Delhi, India; 511 Division of General Internal Medicine and Primary Care, Harvard University, Boston, MA USA; 512 Usher Institute of Population Health Sciences and Informatics, University of Edinburgh, Edinburgh, UK; 513 National Institute of Infectious Diseases, Tokyo, Japan; 514 Finnish Institute of Occupational Health, Helsinki, Finland; 515 Institute of Medical Epidemiology, Martin Luther University Halle-Wittenberg, Halle, Germany; 516 Department of Epidemiology, University of Alabama at Birmingham, Birmingham, AL USA; 517 Department of Medicine, University of Alabama at Birmingham, Birmingham, AL USA; 518 Department of Epidemiology, School of Preventive Oncology, Patna, India; 519 Department of Epidemiology, Healis Sekhsaria Institute for Public Health, Mumbai, India; 520 Department of Physiotherapy and Occupational Therapy, Næstved-Slagelse-Ringsted Hospitals, Slagelse, Denmark; 521 Medical Division, German Leprosy and TB Relief Association Ethiopia, Addis Ababa, Ethiopia; 522 Department of Medicine, University of Washington, Seattle, WA USA; 523 Department of Medicine, University of Calgary, Calgary, Alberta Canada; 524 Social Development and Health Promotion Research Center, Kermanshah University of Medical Sciences, Kermanshah, Iran; 525 Hospital Universitario de la Princesa, Autonomous University of Madrid, Madrid, Spain; 526 Centro de Investigación en Red de Enfermedades Respiratorias (CIBERES), Institute of Health Carlos III, Madrid, Spain; 527 Division of Community Medicine, International Medical University, Kuala Lumpur, Malaysia; 528 Department of Nursing, Muhammadiyah University of Surakarta, Kartasura, Indonesia; 529 Department of Community Medicine, Ahmadu Bello University, Zaria, Nigeria; 530 Department of Criminology, Law and Society, University of California Irvine, Irvine, CA USA; 531 Neurology Department, Sree Chitra Tirunal Institute for Medical Sciences and Technology, Trivandrum, India; 532 Carlos III Health Institute, Biomedical Research Networking Center for Mental Health Network (CIBERSAM), Madrid, Spain; 533 Department of Medicine, University of Valencia, Valencia, Spain; 534 School of Social Work, University of Illinois, Urbana, IL USA; 535 Cancer Control Center, Osaka International Cancer Institute, Osaka, Japan; 536 University Institute ‘Egas Moniz’, Monte Da Caparica, Portugal; 537 Research Institute for Medicines, Faculty of Pharmacy of Lisbon, University of Lisbon, Lisbon, Portugal; 538 Department of Pediatrics, King Saud University, Riyadh, Saudi Arabia; 539 College of Medicine, Alfaisal University, Riyadh, Saudi Arabia; 540 Anesthesiology Department, University of Virginia, Charlottesville, VA USA; 541 Syrian Expatriate Medical Association (SEMA), Charlottesville, VA USA; 542 Department of Public Health and Community Medicine, Central University Kerala, Kasaragod, India; 543 Nanyang Technological University, Singapore, Singapore; 544 School of Exercise and Nutrition Sciences, Queensland University of Technology, Brisbane, Queensland Australia; 545 Department of Pathology and Legal Medicine, University of São Paulo, Sao Paulo, Brazil; 546 Department of Health Economics, Hanoi Medical University, Hanoi, Vietnam; 547 Department of Molecular Medicine and Pathology, University of Auckland, Auckland, New Zealand; 548 Clinical Hematology and Toxicology, Military Medical University, Hanoi, Vietnam; 549 Gomal Center of Biochemistry and Biotechnology, Gomal University, Dera Ismail Khan, Pakistan; 550 TB Culture Laboratory, Mufti Mehmood Memorial Teaching Hospital, Dera Ismail Khan, Pakistan; 551 Division of Health Sciences, University of Warwick, Coventry, UK; 552 Department of Education and Health, Trauma Research Center, Tehran, Iran; 553 Critical and Intensive Care Department, Trauma Research Center, Tehran, Iran; 554 Argentine Society of Medicine, Buenos Aires, Argentina; 555 Velez Sarsfield Hospital, Buenos Aires, Argentina; 556 University Medical Center Groningen, University of Groningen, Groningen, The Netherlands; 557 Department of General Practice, University Medical Center Groningen, Groningen, The Netherlands; 558 Ukk Institute, Tampere, Finland; 559 Psychosocial Injuries Research Center, Ilam University of Medical Sciences, Ilam, Iran; 560 Raffles Neuroscience Centre, Raffles Hospital, Singapore, Singapore; 561 Yong Loo Lin School of Medicine, National University of Singapore, Singapore, Singapore; 562 Department of Medical and Surgical Sciences, University of Bologna, Bologna, Italy; 563 Occupational Health Unit, Sant’orsola Malpighi Hospital, Bologna, Italy; 564 Department of Information Technologies and Management, Moscow Institute of Physics and Technology, Dolgoprudny, Russia; 565 Department of Information and Internet Technologies, I. M. Sechenov First Moscow State Medical University, Moscow, Russia; 566 Department of Health Care Administration and Economy, National Research University Higher School of Economics, Moscow, Russia; 567 Foundation University Medical College, Foundation University, Rawalpindi, Pakistan; 568 Department of Statistics, University of Washington, Seattle, WA USA; 569 Department of Epidemiology and Biostatistics, Wuhan University, Wuhan, China; 570 Department of Psychiatry, University of São Paulo, Sao Paulo, Brazil; 571 Institute of Child Health, University College London, London, UK; 572 Cardiology Department, Royal Children’s Hospital, Melbourne, Victoria Australia; 573 Murdoch Childrens Research Institute, Melbourne, Victoria Australia; 574 School of Nursing, Aksum University, Aksum, Ethiopia; 575 Competence Center of Mortality-Follow-Up, Federal Institute for Population Research, Wiesbaden, Germany; 576 Cochrane South Africa, Medical Research Council South Africa, Cape Town, South Africa; 577 Department of Global Health, Stellenbosch University, Cape Town, South Africa; 578 Department of Pharmacology and Toxicology, Mekelle University, Mekelle, Ethiopia; 579 Department of Pharmacology, Addis Ababa University, Addis Ababa, Ethiopia; 580 Zhejiang Spine Research Center, Wenzhou Medical University, Wenzhou, China; 581 School of Medicine, Nanjing University, Nanjing, China; 582 Department of Diabetes and Metabolic Diseases, University of Tokyo, Tokyo, Japan; 583 Department of Health Management, Policy and Economics, Kerman University of Medical Sciences, Kerman, Iran; 584 Health Services Management Research Center, Kerman University of Medical Sciences, Kerman, Iran; 585 Department of Pediatrics, University of Jos, Jos, Nigeria; 586 Department of Pediatrics, Jos University Teaching Hospital, Jos, Nigeria; 587 Centre for Suicide Research and Prevention, University of Hong Kong, Hong Kong, China; 588 Department of Social Work and Social Administration, University of Hong Kong, Hong Kong, China; 589 Department of Psychopharmacology, National Center of Neurology and Psychiatry, Tokyo, Japan; 590 Health Economics & Finance, Global Health, Jackson State University, Jackson, MS USA; 591 Research Center for Public Health, Tsinghua University, Peking, China; 592 Prevention of Cardiovascular Disease Research Center, Shahid Beheshti University of Medical Sciences, Tehran, Iran; 593 Medical Parasitology Department, Cairo University, Cairo, Egypt; 594 Global Health Institute, Wuhan University, Wuhan, China; 595 Department of Health Management and Economics, A.C.S. Medical College and Hospital, Tehran, Iran; 596 Department of Electrical Engineering, Sharif University of Technology, Tehran, Iran; 597 Electrical Engineering, Institute for Research in Fundamental Sciences, Tehran, Iran; 598 Social Determinants of Health Research Center, Ardabil University of Medical Science, Ardabil, Iran; 599 Maternal and Child Health Division, International Centre for Diarrhoeal Disease Research Bangladesh, Dhaka, Bangladesh; 600 Department of Medicine, Monash University, Melbourne, Victoria Australia; 601 Student Research Committee, Babol University of Medical Sciences, Babol, Iran; 602 Department of Community Medicine, Ardabil University of Medical Science, Ardabil, Iran; 603 Social Development and Health Promotion Research Center, Kermanshah University of Medical Sciences, Kermanshah, Iran; 604 Maternal and Child Wellbeing Unit, African Population Health Research Centre, Nairobi, Kenya; 605 Public Health Department, Dilla University, Dilla, Ethiopia; 606 Department of Preventative Medicine, Wuhan University, Wuhan, China; 607 School of Public Health, Wuhan University of Science and Technology, Wuhan, China; 608 Indian Institute of Public Health, Public Health Foundation of India, Gurugram, India; 609 School of BioSciences, University of Melbourne, Parkville, Victoria, Australia; 610 State University of Semarang, Public Health Science Department, Kota Semarang, Indonesia; 611 Graduate Institute of Biomedical Informatics, Taipei Medical University, Taipei City, Taiwan

**Keywords:** Paediatrics, Public health, Developing world

## Abstract

Since 2000, many countries have achieved considerable success in improving child survival, but localized progress remains unclear. To inform efforts towards United Nations Sustainable Development Goal 3.2—to end preventable child deaths by 2030—we need consistently estimated data at the subnational level regarding child mortality rates and trends. Here we quantified, for the period 2000–2017, the subnational variation in mortality rates and number of deaths of neonates, infants and children under 5 years of age within 99 low- and middle-income countries using a geostatistical survival model. We estimated that 32% of children under 5 in these countries lived in districts that had attained rates of 25 or fewer child deaths per 1,000 live births by 2017, and that 58% of child deaths between 2000 and 2017 in these countries could have been averted in the absence of geographical inequality. This study enables the identification of high-mortality clusters, patterns of progress and geographical inequalities to inform appropriate investments and implementations that will help to improve the health of all populations.

## Abstract (web summary)

A high-resolution, global atlas of mortality of children under five years of age between 2000 and 2017 highlights subnational geographical inequalities in the distribution, rates and absolute counts of child deaths by age.

## Main

Gains in child survival have long served as an important proxy measure for improvements in overall population health and development^1,2^. Global progress in reducing child deaths has been heralded as one of the greatest success stories of global health^3^. The annual global number of deaths of children under 5 years of age (under 5)^4^ has declined from 19.6 million in 1950 to 5.4 million in 2017. Nevertheless, these advances in child survival have been far from universally achieved, particularly in low- and middle-income countries (LMICs)^4^. Previous subnational child mortality assessments at the first (that is, states or provinces) or second (that is, districts or counties) administrative level indicate that extensive geographical inequalities persist^5–7^.

Progress in child survival also diverges across age groups^4^. Global reductions in mortality rates of children under 5—that is, the under-5 mortality rate (U5MR)—among post-neonatal age groups are greater than those for mortality of neonates (0–28 days)^4,8^. It is relatively unclear how these age patterns are shifting at a more local scale, posing challenges to ensuring child survival. To pursue the ambitious Sustainable Development Goal (SDG) of the United Nations^9^ to “end preventable deaths of newborns and children under 5” by 2030, it is vital for decision-makers at all levels to better understand where, and at what ages, child survival remains most tenuous.

## Precision public health and child mortality

Country-level estimates facilitate international comparisons but mask important geographical heterogeneity. Previous assessments of mortality of children under 5 have noted significant within-country heterogeneity, particularly in sub-Saharan Africa^5,7,10–14^, as well as in Brazil^15^, Iran^16^ and China^17^. Understanding public health risks at more granular subpopulation levels is central to the emerging concept of precision public health^18^, which uses “the best available data to target more effectively and efficiently interventions…to those most in need”^18^. Efforts to produce high-resolution estimates of mortality of children under 5, determinants at scales that cover the multiple countries are emerging, including for vaccine coverage^19,20^, malaria^21^, diarrhoea^22^ and child growth failure^23,24^. In a previous study, we produced comprehensive estimates of African child mortality rates at a 5 × 5-km scale for 5-year intervals^5^. For areas outside of Africa, in which 72% of the world’s children live and 46% of global child deaths occurred in 2017^4^, subnational heterogeneity remains mostly undescribed^25^.

Here we produce estimates of death counts and mortality rates of children under 5, infants (under 1 years of age) and neonates (0–28 days) in 99 countries at policy-relevant subnational scales (first and second administrative levels) for each year from 2000 to 2017. We fit a geostatistical discrete hazards model to a large dataset that is composed of 467 geo-referenced household surveys and censuses, representing approximately 15.9 million births and 1.1 million deaths of children from 2000 to 2017. Our model includes socioeconomic, environmental and health-related spatial covariates with known associations to child mortality and uses a Gaussian process random effect to exploit the correlation between data points near each other across dimensions of space, time and age group, which helps to mitigate the limitations associated with data sparsity in our estimations. For this study, we report U5MR as the expected number of deaths per 1,000 live births, reflecting the probability of dying before the age of 5 for a given location and year.

## Unequal rates of child mortality

**Fig. 1:**
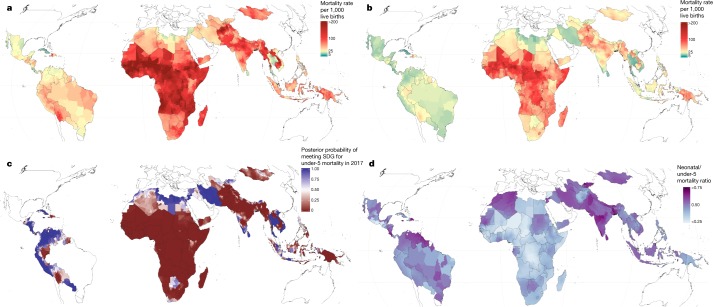
U5MR estimates in 99 LMICs. **a**, U5MR at the second administrative level in 2000. **b**, U5MR at the second administrative level in 2017. **c**, Modelled posterior exceedance probability that a given second administrative unit had achieved the SDG 3.2 target of 25 deaths per 1,000 live births for children under 5 in 2017. **d**, Proportion of mortality of children under 5 occurring in the neonatal (0–28 days) group at the second administrative level in 2017.

**Fig. 2:**
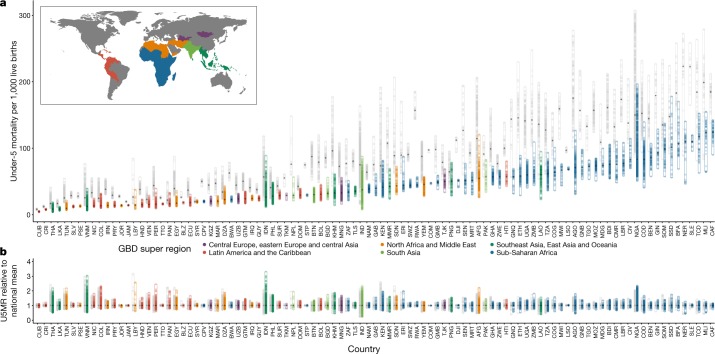
Geographical inequality in U5MR across 99 countries for 2000 and 2017. **a**, Absolute inequalities. Range of U5MR estimates in second administrative-level units across 99 LMICs. **b**, Relative inequalities. Range of ratios of U5MR estimates in second administrative-level units relative to country means. Each dot represents a second administrative-level unit. The lower bound of each bar represents the second administrative-level unit with the lowest U5MR in each country. The upper end of each bar represents the second administrative-level unit with the highest U5MR in each country. Thus, each bar represents the extent of geographical inequality in U5MRs estimated for each country. Bars indicating the range in 2017 are coloured according to their Global Burden of Disease super-region. Grey bars indicate the range in U5MR in 2000. The diamond in each bar represents the median U5MR estimated across second administrative-level units in each country and year. A coloured bar that is shorter than its grey counterpart indicates that geographical inequality has narrowed.

The risk of a newborn dying before their fifth birthday varies tremendously based on where in the world, and within their country, they are born. Across the 99 countries in this study, we estimate that U5MR varied as much as 24-fold at the national level in 2017, with the highest rate in the Central African Republic of 123.9 deaths (95% uncertainty interval, 104.9–148.2) per 1,000 live births, and the lowest rate in Cuba of 5.1 deaths (4.4–6.0)^4^. We observed large subnational variation within countries in which overall U5MR was either high or comparatively low. For example, in Vietnam, rates across second administrative units (henceforth referred to as ‘units’) varied 5.7-fold, from 6.9 (4.6–9.8) in the Tenth District in Hồ Chí Minh City to 39.7 (28.1–55.6) in Mường Tè District in the Northwest region (Figs. 1b, 2).

**Extended Data Fig. 1:**
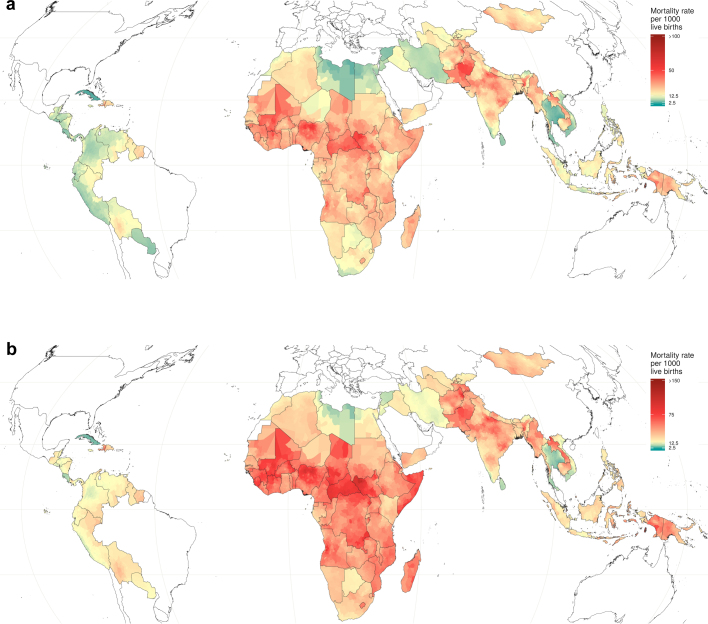
Neonatal and infant mortality rates in 2017. **a**, **b**, Maps showing the mortality rates of neonates (**a**; birth to 28 days of age) and infants (**b**; under 1 year of age) across second administrative-level units in 2017. Note that the ranges in the keys are different for the two maps.

Decreases in U5MR between 2000 and 2017 were evident to some extent throughout all units (Figs. 1a, b, 2). No unit showed a significant increase in U5MR in this period, and in most units U5MR decreased greatly, even in units in which the mortality risk was the highest. Out of 17,554 units, 60.3% (10,585 units) showed a significant (defined as 95% uncertainty intervals that did not overlap) decrease in U5MR between 2000 and 2017. Across units in 2000, U5MR ranged from 7.5 (5.0–10.6) in Santa Clara district, Villa Clara province, Cuba, to 308.4 (274.9–348.4) in the Sabon Birni Local Government Area of Sokoto State, Nigeria. By 2017, the unit with the highest estimated U5MR across all 99 countries was Garki Local Government Area, Jigawa state, Nigeria, at 195.1 (158.6–230.9). Overall, the total percentage of units with a U5MR higher than 80 deaths per 1,000 live births decreased from 28.9% (5,070) of units in 2000 to 7.0% (1,236) in 2017. Furthermore, 32% of units, representing 11.9% of the under-5 population in the 99 countries, had already met SDG 3.2 for U5MR with a 90% certainty threshold (Fig. 1c). For neonatal mortality, 34% of units met the target of ≤12 deaths per 1,000 live births (Extended Data Fig. 1). Within countries, successes were mixed in some cases. For example, Colombia, Guatemala, Libya, Panama, Peru and Vietnam had all achieved SDG 3.2 for U5MR at the national level by 2017, but each country had units that did not achieve the goal with 90% certainty (Fig. 1c).

Successful reductions in child mortality were also observed throughout entire countries. For example, in 43 LMICs across several world regions, the worst-performing unit in 2017 had a U5MR that was lower than the best-performing unit in 2000 (Fig. 2). Nearly half of these countries were in sub-Saharan Africa. Rwanda showed notable progress during the study period, reducing mortality from 144.0 (130.0–161.6) in its best-achieving district in 2000 (Rubavu) to 57.2 (47.4–72.1) in its worst-achieving district in 2017 (Kayonza). These broad reductions in U5MR have also led to a convergence of absolute subnational geographical inequalities, although relative subnational inequalities appear to be mostly unchanged between 2000 and 2017 (Fig. 2 and Supplementary Fig. 6.12). Despite this success, the highest U5MRs in 2017 were still largely concentrated in areas in which rates were highest in 2000 (Fig. 1a, b). We observed estimated U5MR ≥ 80 across large geographical areas in Western and Central sub-Saharan Africa, and within Afghanistan, Cambodia, Haiti, Laos and Myanmar (Fig. 1b).

Deaths of neonates (0–28 days of age) and post-neonates (28–364 days of age) have come to encompass a larger fraction of overall mortality of children under 5 in recent years. By 2017 (Fig. 1d), neonatal mortality increased as a proportion of total deaths of children under 5 in 91% (90) of countries and for 83% (14,656) of units compared to 2000. In almost all places where U5MR decreased, the share of the mortality burden increased in the groups of children with younger ages. Similarly, the mortality of infants (<1 year) has increased relative to the mortality for children who are 1–4 years of age in many areas. For example, in the Diourbel Region, Senegal, infant mortality constituted 54.4% (52.4–56.6) of total mortality of children under 5 in 2000; by 2017, the relative contribution of infant mortality was 73.2% (70.3–75.8). This shift towards mortality predominantly affecting neonates and infants was not as evident in all locations; mortality for children aged 1–4 years was responsible for more than 30% of overall under-5 deaths in 13% (2,226) of units, mostly within high-mortality areas in sub-Saharan Africa.

## Distribution of under-5 deaths may not follow rates

**Fig. 3:**
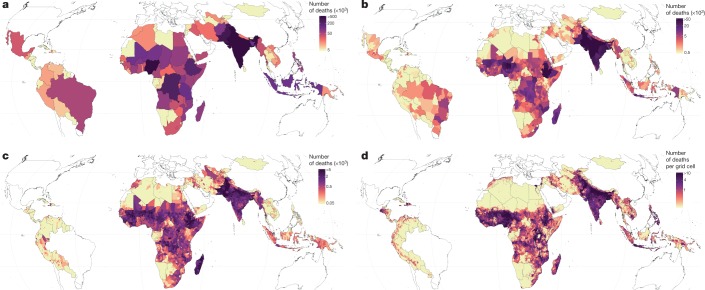
Estimated number of children under 5 who died within 99 countries in 2017. **a**, Number of deaths of children under 5 in each country. **b**, Number of deaths in each first administrative-level unit. **c**, Number of deaths in each second administrative-level unit. **d**, Number of deaths of children under 5 in each 5 × 5-km grid cell. Note that scales vary for each aggregation unit.

The goal of mortality-reduction efforts is ultimately to prevent premature deaths, and not just to reduce mortality rates. Across the countries studied here, there were 3.5 million (41%) fewer deaths of children under 5 in 2017 than in 2000 (5.0 million compared to 8.5 million). At the national level, the largest number of child deaths in 2017 occurred in India (1.04 (0.98–1.10) million), Nigeria (0.79 (0.65–0.96) million), Pakistan (0.34 (0.27–0.41) million) and the Democratic Republic of the Congo (0.25 (0.21–0.31) million) (Fig. 3a). Within these countries, the geographical concentration of the deaths of the children varied. In Pakistan, over 50% of child deaths in 2017 occurred in Punjab province, which had a U5MR of 63.3 (54.1–76.0) deaths per 1,000 live births (Fig. 3b). By contrast, 50% of child deaths in the Democratic Republic of the Congo in 2017 occurred across 9 out of 26 provinces. Such findings are in a large part artefacts of how borders are drawn around various at-risk populations (the provinces above account for 53% and 63%, respectively, of the under-5 population that is at risk in these two countries), but can have a real impact at the level at which planning occurs. Some concentrated areas with apparent high absolute numbers of deaths highlighted by local-level estimates become less noticeable when reporting at aggregated administrative levels; for example, areas across Guatemala, Honduras and El Salvador are visually striking hotspots in Fig. 3d, but less so in Fig. 3b, c.

**Fig. 4:**
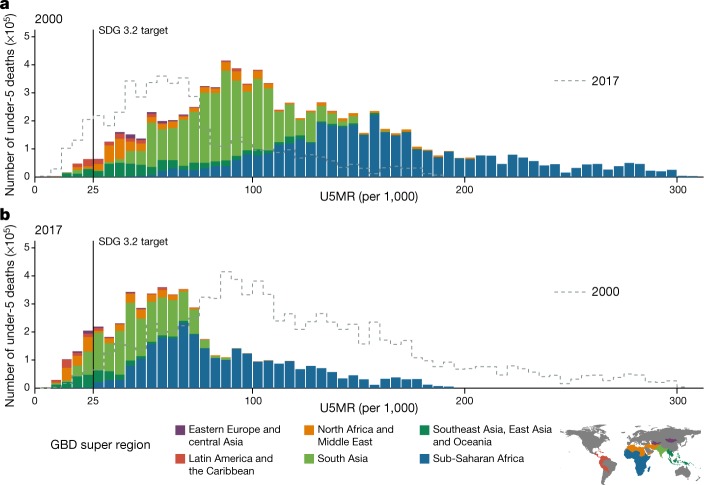
Number of deaths of children under 5, distributed across level of U5MR, in 2000 and in 2017, across 99 countries. Bar heights represent the total number of deaths of children under 5 within all second administrative-level units with corresponding U5MR. Bins are a width of 5 deaths per 1,000 live births. The colour of each bar represents the global region as defined by the subset legend map. As such, the sum of heights of all bars represents the total number of deaths across the 99 countries. **a**, Deaths of children under 5 in 2000. **b**, Deaths of children under 5 in 2017. The dotted line in the 2000 plot is the shape of the distribution in 2017, and the dotted line in the 2017 plot represents the distribution in 2000.

Our estimates indicate that targeting areas with a ‘high’ U5MR of 80 will have a lower overall effect than in previous years owing to the reductions in mortality rates. In 2000, 23.7% of child deaths—representing 2.0 (1.7–2.4) million deaths—occurred in regions in which U5MR was less than 80 that year (Fig. 4). By comparison, in 2017, 69.5% of child deaths occurred in areas in which U5MR was below 80. A growing proportion of deaths of children under 5 are occurring in ‘low’-mortality areas; 7.3% (5.1–10.2) of all deaths of children under 5 in 2017 occurred in locations in which the U5MR was below the SDG 3.2 target rate of 25, compared to 1.2% (0.9–1.6) in 2000. For instance, Lima, Peru, has a U5MR in the 8th percentile of units in this study, yet it ranks in the 96th percentile of highest number of deaths of children under 5.

Despite population growth, child deaths have declined due to the outpaced decline in U5MR. For example, there were a total of 8.5 (7.2–10.0) million deaths of children under 5 in the countries in this study in 2000; had the 2017 under-5 population been exposed to the same U5MRs that were observed in 2000, there would have been 10.6 (9.0–12.5) million deaths in 2017. Instead, we observed 5.0 (3.8–6.6) million deaths in 2017 (Extended Data Fig. 5).

**Extended Data Fig. 2:**
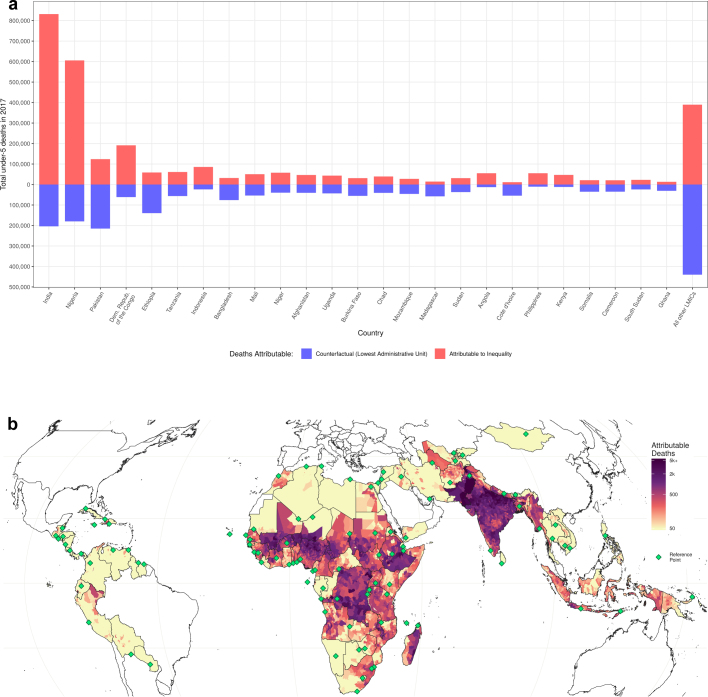
Impact of inequality on U5MR. **a**, Potential reduction in the number of deaths that would have occurred if all second administrative-level units in the 20 countries with the greatest number of deaths of children under 5 in 2017 realized a homogenous U5MR that was equal to that of the lowest observed mortality rate in that country. In total, 66% of under-5 deaths could have been averted if all countries maintained mortality rates equal to the second administrative-level unit with lowest mortality. If this reference rate is set to the lowest observed rate across all of the 99 countries that were included in this study, 95% of under-5 deaths could have been averted. The size of each bar represents the total number of under-5 deaths in each country. The red portion of each bar indicates the number of deaths ‘attributed’ to geographical inequality in mortality rates, whereas the blue portion represents the number of deaths that would remain in the scenario in which all second administrative-level units within countries had the same mortality rate as the best-performing unit. **b**, Locations of under-5 deaths ‘attributable’ to geographical inequality, across all second administrative-level units in each country. Each country has one unit highlighted with a green diamond, which is the reference unit, or the location with the lowest mortality rate in the country in 2017.

Finally, we combine estimates of subnational variation in mortality rates and populations to gain a better understanding of the impact of geographical inequality. Overall, 2.7 (2.5–2.9) million deaths, or 54% of the total number of deaths of children under 5, would have been averted in 2017 had all units had a U5MR that matched the best-performing unit in each respective country (Extended Data Fig. 2). Over the 2000–2017 period, this number is 71.8 (68.5–74.9) million deaths, or 58% (55–61) of the total number of deaths of children under 5. Total deaths attributable to inequality in this scenario ranged from 13 (6–24) deaths in Belize to 0.84 (0.72–0.99) million deaths in India. Furthermore, had all units met the SDG 3.2 target of 25 deaths per 1,000, an estimated 2.6 (2.3–2.8) million deaths of children under 5 would have been averted in 2017.

## Discussion

This study offers a comprehensive, geospatially resolved resource for national and subnational estimates of child deaths and mortality rates for 99 LMICs, where 93% of the world’s child deaths^4^ occurred in 2017. Gains in child survival varied substantially within the vast majority of countries from 2000 to 2017. Countries such as Vietnam, for example, showed more than fivefold variation in mortality rates across second administrative-level units. The inconsistency of successes, even at subnational levels, indicates how differences in health policy, financial resources, access to and use of health services, infrastructure, and economic development ultimately contribute to millions of lives cut short^25–27^. By providing detailed maps that show precisely where these deaths are estimated to have occurred, we provide an important evidence base for looking both to the past, for examples of success, and towards the future, in order to identify where precision public-health initiatives could save the most lives.

The epidemiological toll of child mortality should be considered both in terms of total deaths and as rates of mortality. Focusing only on mortality rates can effectively mask areas in which rates are comparatively low but child deaths are high owing to large population sizes. The number of deaths that occur in high-risk areas has declined, and most under-5 deaths in recent years have occurred in lower-risk areas. This ‘prevention paradox’^28^ could indicate that whole-population interventions could have a larger overall impact than targeting high-risk areas^29^. At the same time, strategies that target resources to those locations that have the highest number of child deaths risk leaving behind some of the world’s most marginalized communities: remote, more-sparsely populated places in which, relative to the number of children born each year, a large number of children die before their fifth birthday. Instead, by considering subnational measures of both counts and rates of deaths of children under 5, decision-makers can better tailor child health programs to align with local contexts, norms and needs. Rural communities with high rates but low counts may benefit from ‘last-mile’ initiatives to provide effective health services to populations who lack adequate access to care. By contrast, locations with low rates but high counts may require programs that focus on alleviating the cost of care, unsafe environmental exposures or health risks that are uniquely associated with urban slums^30^. The SDGs have pointed the global development agenda towards progress in child survival. Our analysis indicates that reaching the SDG 3.2 targets of 25 child deaths per 1,000 live births and 12 neonatal deaths per 1,000 live births will require only modest improvements or have already been achieved by some units; however, these targets are ambitious for other units in which child mortality remains high. It is worth noting that many countries contain areas that fit both of these profiles. For example, 11 countries had at least 1 unit that had already met SDG 3.2 with high certainty, and at least 1 unit that had not. Subnational estimates can empower countries to benchmark gains in child survival against their own subnational exemplars as well as advances that have been achieved by their peers. Through our counterfactual analysis we showed that even if all units had met the SDG 3.2 goal in 2017, there would still have been 2.4 million deaths of children under 5, indicating that ‘ending preventable child deaths’ is more complex than simply meeting a target threshold. Future research efforts must address the causes of child mortality in local areas and more precisely identify causes of child deaths that are amenable to intervention. To that end, new and innovative data-collection efforts, such as the ongoing Child Health and Mortality Prevention Surveillance network, offer promising prospects by applying high-validity, pathology-based methods alongside verbal autopsies to determine the cause of death^31^.

This study offers a unique platform to support the identification of local success stories that could be replicated elsewhere. In Rwanda, for example, the highest U5MR at the district level in 2017 was 60.2% (52.0–67.8%) lower than the lowest U5MR at the district level in 2000. Such gains have been partially credited to focused investments in the country’s poorest populations, expanding the Mutuelles de santé insurance program, and developing a strong workforce of community health workers who provide evidence-based treatment and health promotion^32,33^. Nepal and Cambodia are among the exemplars for considerably decreasing subnational inequalities in child survival since 2000. In an era when narrowing disparities within countries is as important as reducing national-level gaps, these results provide the evidence base to inform best practices and stimulate national conversations about related social determinants.

Neonatal mortality rates have also declined but failed to keep pace with reductions in mortality rates of older children, leading to a higher proportion of deaths of children under 5 occurring within the first four weeks of life: from 37.4% (37.1–37.7) in 2000 to 43.7% (43.1–44.3%) in 2017. This trend is probably related to the increase in scale of routine programs and improved infrastructure (for example, vaccination^34^, and water and sanitation^35^) and the introduction of effective interventions to target communicable diseases (for example, malaria control^36^ and prevention of mother-to-child transmission of HIV^37^). These interventions have tended to target amenable causes of mortality that are more common in older children under 5 rather than dominant causes of neonatal mortality, such as prematurity and congenital anomalies^38^. Notably, irrespective of income level or location, some causes of neonatal death (for example, chromosomal anomalies and severe preterm birth complications) remain difficult to prevent completely with current medical technologies. Ultimately, large gains in neonatal mortality will require serious investment in health system strengthening^39^. Affordable approaches to preventing the majority of neonatal deaths in LMICs exist and there are success stories with lessons learned to apply^40–44^, but decisions about which approaches to take must be based on the local epidemiological and health system context. In the absence of spatially detailed cause of death data, subnational neonatal mortality estimates can indicate dominant causes and thus serve as a useful proxy to guide prioritization of interventions^45^.

**Extended Data Fig. 3:**
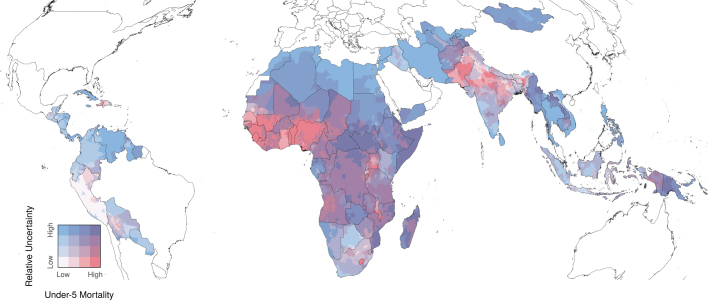
Relative uncertainty in U5MR estimates for 2017. Relative uncertainty in second administrative-level estimates compared with mean estimated U5MRs in each second administrative-level unit for 2017. Mean rates and relative uncertainty are split into population-weighted quartiles. These cut-off points indicate the relative uncertainty minimum, 25th, 50th and 75th percentiles, and maximum, which are 0.29, 0.51, 0.63 and 0.86, and 3.12, respectively. The under-5 mortality minimum, 25th, 50th and 75th percentiles, and maximum are 1.4, 13.0, 22.9 and 44.8, and 190.6 deaths per 1,000 live births. Areas in which our estimates are more uncertain are coloured with a scale of increasing blue hue, whereas areas in which the mean estimates of U5MR are high are coloured with a scale of increasing red hue. Purple areas have high, but uncertain, estimates of U5MRs. White areas have low relative mortality, with fairly certain estimates. Relative uncertainty is defined as the ratio of the width of the 95% uncertainty interval to the mean estimate.

**Extended Data Fig. 4:**
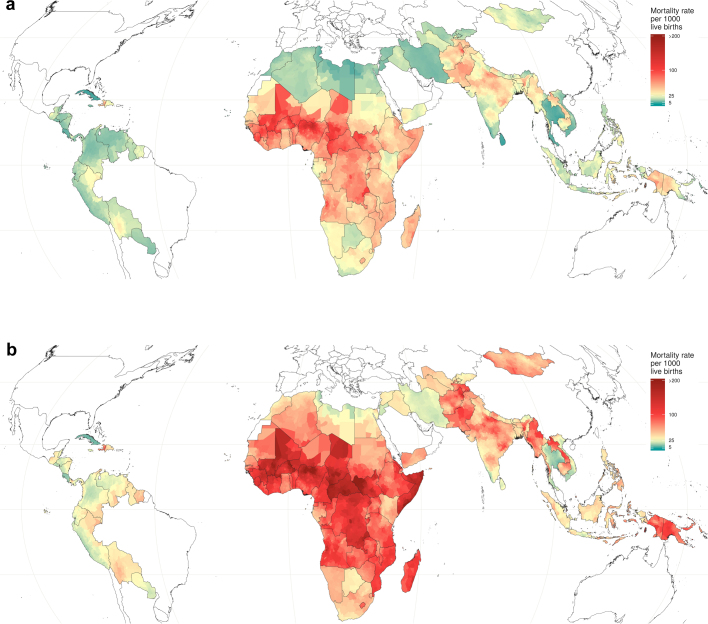
Lower and upper uncertainty interval boundaries for U5MR mortality estimates in 2017. **a**, **b**, Lower (**a**) and upper (**b**) 95% uncertainty intervals for U5MR estimates across the second administrative-level units in 99 countries.

**Extended Data Fig. 5:**
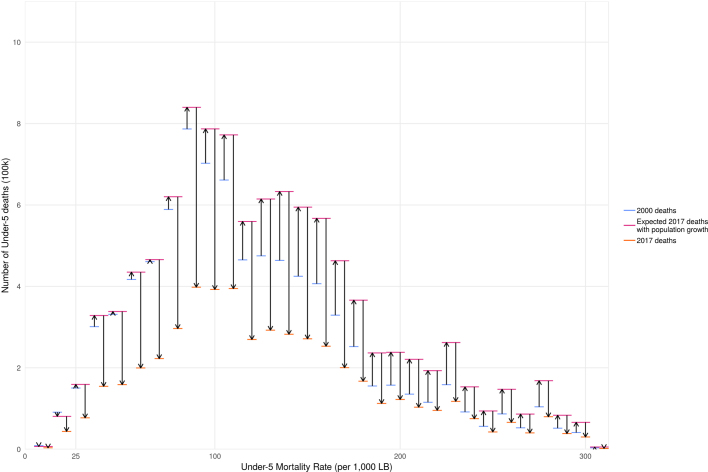
The counteracting forces of population change and mortality rate decline on total number of under-5 deaths. Arrow plots show the mortality rate strata (bins of 10 per 1,000 livebirths) in 2000.

The accuracy and precision of our estimates were primarily determined by the timeliness, quantity and quality of available data. In Sri Lanka, for example, there were no available surveys, and the wide uncertainty intervals surrounding estimates reflect the dearth of available evidence in that country (Extended Data Figs. 3, 4). In certain areas, this decreased the confidence that we had in claiming that a specific subnational area met the SDG 3.2 target (Fig. 1c). This issue is most concerning in cases in which estimated mortality rates are high, thus helping to identify locations in which it would be most useful to focus future data-collection efforts. High mortality rates with large uncertainty intervals were estimated across much of Eastern and Central sub-Saharan Africa, and in Cambodia, Laos, Myanmar and Papua New Guinea (Extended Data Figs. 3, 4). Furthermore, ongoing conflict in countries such as Syria, Yemen and Iraq pose substantial challenges to collecting more contemporaneous data, and our estimates may not fully capture the effects of prolonged civil unrest or war^46,47^. Further methodological and data limitations are discussed in the Methods.

The accurate estimation of mortality is also a matter of equity; highly refined health surveillance is common in high-income countries, whereas in LMICs, in which rates of child mortality are the highest, surveillance that helps to guide investments in health towards the areas with the greatest need is less routine^48^. Ideally, all countries would have high-quality, continuous, and complete civil and vital registration systems that capture all of the births, deaths and causes of death at the appropriate geographical resolution^49^. In the meantime, analyses such as this serve to bridge the information gap that exists between low-mortality countries with strong information systems and countries that face a dual challenge of weaker information systems and higher disease burden.

By harnessing the unprecedented availability of geo-referenced data and developing robust statistical methods, we provide a high-resolution atlas of child death counts and rates since 2000, covering countries that account for 93% of child deaths. We bring attention to subnational geographical inequalities in the distribution, rates and absolute counts of child deaths by age. These high-resolution estimates can help decision-makers to structure policy and program implementation and facilitate pathways to end preventable child deaths^50^ by 2030.

## Methods

### Overview

**Extended Data Fig. 6:**
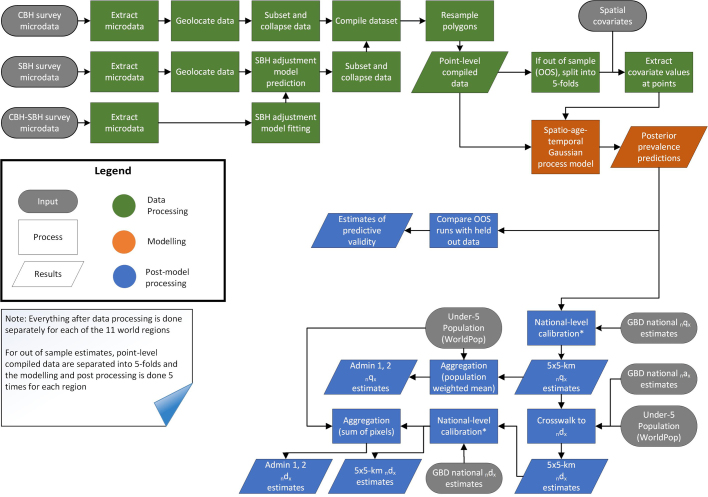
Flowchart summarizing analytical process. Standard demographic notation were used. *n*, length of age bin; *x*, starting age of age bin; *d*, number of deaths; *q*, probability of death; *a*, average time lived in age bin by those who died in the age bin.

We fitted a discrete hazards geostatistical model^51,52^ with correlated space–time–age errors and made predictions to generate joint estimates—with uncertainty—of the probability of death (the number of deaths per live births) and the number of deaths for children aged 0–28 days (neonates), children under 1 year old (infants) and children under 5 years old at the subnational level for 99 LMICs for each year from 2000 to 2017. The analytical process is summarized in the flowchart in Extended Data Fig. 6. We made estimates at a grid-cell resolution of approximately 5 × 5-km and then produced spatially aggregated estimates at the first (that is, states or provinces) and second (that is, districts or counties) administrative levels, as well as the country level.

Countries were selected for inclusion in this study based on their socio-demographic index (SDI) published in the Global Burden of Disease study (GBD)^53^. The SDI is a measure of development based on income per capita, educational attainment and fertility rates among women under 25 years old. We primarily aimed to include all countries in the middle, low–middle or low SDI quintiles, with several exceptions. Brazil and Mexico were excluded despite middle SDI status owing to the availability of high-quality vital registration data in these countries, which have served as the basis for existing subnational estimates of child mortality. Because this study did not incorporate vital registration data sources (see ‘Limitations’), Brazil and Mexico were not estimated directly; instead, state-level estimates from the GBD 2017 study were directly substituted in figures where appropriate^4^. Albania and Moldova were excluded despite middle SDI status owing to geographical discontinuity with other included countries and lack of available survey data. North Korea was excluded despite low–middle SDI status owing to geographical discontinuity and insufficient data. As countries with high–middle SDI status in 2017, China and Malaysia were excluded from this analysis. Libya was included despite high–middle SDI status to create better geographical continuity. Island nations with populations under 1 million were excluded because they typically lacked sufficient survey data or geographical continuity for a geospatial analytic approach to be advantageous over a national approach. Supplementary Figure 3.1 shows a map of the countries included in this study and Supplementary Table 3.1 lists the countries.

### Data

We extracted individual records from 555 household sample survey and census sources. Records came in the form of either summary birth histories (SBHs) or complete birth histories (CBHs). All input data were subject to quality checks, which resulted in the exclusion of 82 surveys and censuses owing to quality concerns (see Supplementary Information section 3.2 for more details). Data on life and mortality experiences from CBH sources can be tabulated directly into discrete period and age bins, thus allowing for period-specific mortality estimations, known as the synthetic cohort method^54–56^. For SBH data, we used indirect estimation^57^ to estimate age-specific mortality probabilities and sample sizes and assign them to specific time periods. Complete details are available in Supplementary Information section 3.3.2.

In all cases, after pre-processing, each data point provided a number of deaths and a sample size for an age bin in a specific year and location. We referenced all data points to GPS coordinates (latitude and longitude) wherever possible. In cases in which GPS data were unavailable, we matched data points to the smallest possible areal unit (also referred to as ‘polygons’). All polygon data were spatially resampled into multiple GPS coordinates and weighted based on the population distribution following a previously described procedure^5,22,23,58^ and described in Supplementary Information section 3.4. Our combined global dataset contained approximately 15.9 million births and 1.1 million child deaths. A complete list of data sources is provided in Supplementary Table 8.1.

In addition to data on child mortality, we used a number of spatial data sources for this analysis. These included a suite of geospatial covariates, population estimates and administrative boundaries^68^. These sources and processing procedures are described in Supplementary Information section 4.

### Spatial covariates

We extracted values from each of 10 geospatial covariates at each data point location. Geospatial covariates are spatial data represented at the 5 × 5-km grid-cell resolution. The covariates were travel time to the nearest city, educational attainment of maternal-aged women, the ratio of population of children under 5 to women of reproductive age (ages 15–49 years old), the mass per cubic meter of air of particles with a diameter less than 2.5 μm, total population, a binary indicator of urbanicity, intensity of lights at night, the proportion of children aged 12–23 months who had received the third dose of diphtheria–pertussis–tetanus vaccine, incidence rate of *Plasmodium falciparum*-associated malaria in children under 5 and prevalence of stunting in children under 5 (see Supplementary Information). All covariate values were centred on their means and scaled by their standard deviations. Covariates typically had global spatial coverage and values that vary by year. More details of the spatial covariates can be found in Supplementary Information section 4.

### Analysis

#### Geostatistical model

To synthesize information across various sources, and to make consistent estimates across space and time, we fitted discrete hazards^51,52^ geostatistical models^59^ to our data. The models were discrete in the sense that ages were represented in seven mutually exclusive bins (0, 1–5, 6–11, 12–23, 24–35, 36–47 and 48–59 months), each with its own assumed constant mortality probability. The model explicitly accounted for variation across age bin, year and space through inclusion of both fixed and random effects. Indicator variables for each age bin were included to form a discrete baseline mortality hazard function, representing the risk of mortality in discrete bins from birth to 59 months of age with covariates set at their means. Baseline hazard functions were allowed to vary in space and time in response to changing covariate values, as well as in response to linear effect on year. To model this relationship, we estimated the effect of each covariate value on the risk of mortality. These estimated effects were then applied to the gridded surface of covariate values to make predictions across the entire study geography. We also included a Gaussian random effect across countries to account for larger-scale variations due to political or institutional effects, as well as a Gaussian random effect for each data source to account for source-specific biases. Finally, we included a Gaussian process random effect with a covariance matrix structured to account for remaining correlation across age, time and physical space. As such, estimates at a specific age, time or place benefitted from drawing predictive strength from data points nearby in all of these dimensions.

For each modelling region, we fitted one such discrete hazards model with a binomial data likelihood. All data were prepared such that we counted or estimated the number of children entering into (*n*) and dying within (*Y*) each period–age bin from each GPS-point location (*s*) in each survey (*k*) within each country (*c*).

The number of deaths for children in age band (*a*) in year (*t*) at location (*s*) was assumed to follow a binomial distribution:

$${Y}_{a,s,t} \sim {\rm{binomial}}\left({n}_{a,s,t},\;{P}_{a,s,t}\right)$$

where *P*_*a*,*s*,*t*_ is the probability of death in age bin *a*, conditional on survival to that age bin for a particular space–time location. Using a generalized linear regression modelling framework, a logit link function is used to relate *P* to a linear combination of effects:

$${\rm{logit}}\left({P}_{a,s,t}\right)={\beta }^{0}+\mathop{\sum }\limits_{a=2}^{7}{I}_{a}{\beta }_{a}^{1}+{\beta }^{2}{X}_{s,t}+{\beta }^{3}t+{\nu }_{c\left[s\right]}+{\nu }_{k\left[s\right]}+{Z}_{a,s,t}$$

The first term, *β*^0^, is an intercept, representing the mean for the first age band when all covariates are equal to zero, whereas ${\beta }_{a}^{1}$ are fixed effects for each age band, representing the mean overall hazard deviation for each age band from the intercept, when all other covariates are equal to zero. *β*^2^ are the effects of geospatial covariates (*X*_*s*,*t*_), which we describe in detail in Supplementary Information section 4. *β*^3^ is an overall linear temporal effect to account for overall temporal trends within the region. All geospatial covariates were centred and scaled by subtracting their mean and dividing by their standard deviations. Each *v* term represents uncorrelated Gaussian random effects: ${\nu }_{c\left[s\right]} \sim {\rm{normal}}\left(0,{\sigma }_{c}^{2}\right)$ is a country-level random effect applied to all location*s* (*s*) within a country (*c*); ${\nu }_{k\left[s\right]} \sim {\rm{normal}}\left(0,{\sigma }_{k}^{2}\right)$ is a data source-level random effect for the survey (*k*) from which the data at location *s* were observed. Data source-level random effects were used to account for systematic variation or biases across data sources and were included in model fitting but not in prediction from fitted models. The term *Z*_*a*,*s*,*t*_ ~ Gaussian process(0, *K*) is a correlated random effect across age, space and time, and is modelled as a four-dimensional mean zero Gaussian process with covariance matrix *K*. This term accounts for structured residual correlation across these spatial–age–temporal dimensions that are not accounted for by any of the model’s other fixed or random effects. This structure was chosen, because the hazard probability for each age group is expected to vary in space and time, and such spatiotemporal correlations are likely to be similar across ages. *K* is constructed as a separable process across age, space and time $\left(K={\Sigma }_{a}\otimes {\Sigma }_{t}\otimes {\Sigma }_{s}\right)$. The continuous spatial component is modelled with a stationary isotropic Matérn covariance function, and the age and temporal effects were each assumed to be discrete auto-regressive order 1. We provide further details on model fitting and specification in Supplementary Information section 5.1.

We assigned priors to all model parameters and performed maximum a posteriori inference using Template Model Builder^60^ software in R version 3.4. We fitted the model separately for each of 11 world regions (see Supplementary Fig. 3.1), owing to memory constraints and to allow model parameters to vary across epidemiologically distinct regions.

#### Post-estimation

Using the joint precision matrix and point estimates, we generated 1,000 draws from all model parameters using a multivariate-normal approximation. These model parameter draws were used to predict corresponding draws of mortality probabilities across all age groups for each grid cell in each year. In other words, for each age bin in each year we estimated 1,000 gridded surfaces of mortality probability estimates, each surface corresponding to one draw from the posterior parameter estimates^61^. All subsequent post-estimation procedures were carried out across draws to propagate model uncertainty. We used these estimated spatiotemporal gridded surfaces of age-specific mortality probabilities to produce various final resulting data products.

From the fitted model parameters, we produced posterior mortality probability estimates for each age group for each 5 × 5-km grid cell for each year from 2000 to 2017. We combined gridded age group estimates to obtain infant (under 1) and child (under 5) mortality estimates at each gridded location. Using a conversion from mortality probability to mortality rates, and using a gridded surface of population, we also estimated the number of deaths that occurred in each age group at each location in each year. For both mortality probabilities and counts, we multiplied out corresponding gridded estimates by a constant to ensure that at the national—and in two countries, the first administrative-level unit—aggregated estimates for each age group and year were calibrated such that they equalled estimates in the GBD study^4^. This calibration allowed us to take advantage of national data sources, such as vital registration, that could not be used in this study. We also aggregated grid-cell-level estimates to first and second administrative-level units using gridded population surface to weight estimates. These steps are described in Supplementary Information section 5.2.

#### Model validation

We used fivefold cross-validation to assess and compare model performance with respect to estimating local trends of age-specific mortality. Each fold was created by combining complete surveys into subsets of approximately 20% of data sources from the input data. Holding out entire surveys at a time served as a comparable approximation to the type of missingness in our data, essentially helping us check how well our model estimates of mortality probabilities compared to empirical estimates of mortality probability from an unobserved data source that did not inform the model.

For each posterior draw, we aggregated to administrative units. Using data aggregated to the administrative unit and aggregated estimate pairs, we calculated the difference between out-of-sample empirical data estimates and modelled estimates, and we report the following summary metrics: mean error, which serves as a measure of bias, the square root of mean errors, which serves as a measure of the total variation in the errors, the correlation and 95% coverage. At the second administrative-level unit for under-5 mortality, our out-of-sample 95% coverage was 93%, correlation was 0.78, mean absolute error was 0.015 and mean error was −0.0011. These results indicate a good overall fit, with minimal bias. This procedure and the full validation results are discussed in Supplementary Information section 5.3.

### Limitations

This work should be assessed in full acknowledgement of several data and methodological limitations. We exclusively used CBH and SBH data from household survey and census data sources. Ideally, estimates of child mortality should incorporate all available data, including data from administrative vital registration systems. Vital registration systems are commonly present in many middle-income and all high-income countries. There are known data-quality issues with vital registration sources in many middle-income countries^48,62^ that add complications to their inclusion in our modelling procedure. For example, systems may not capture all deaths, and this level of ‘underreporting’ probably varies in space, time and age. In addition, underreporting is probably negatively correlated with mortality, and could contribute substantial bias to estimates. Statistical methods must be developed to jointly estimate—and adjust for—underreporting in vital registration data before such data can be used in geospatial models of child mortality. Promising work has begun in this domain in specific countries^63^, but further advancement will be necessary to improve estimates across a time series and across many countries at once.

We assume that SBH and CBH data were retrospectively representative in the locations in which they were collected. As such, we assume that survey respondents did not migrate. High-spatial-resolution migration estimates with which to adjust estimates do not yet exist, and many of the data sources that we use do not collect information on migration. We conducted a focused sensitivity analysis (Supplementary Information section 5.4.4) for migration in six countries, and found that although our results were generally robust, there was variation by country. Furthermore, despite providing high-quality retrospective data from representative samples of households, birth history data can suffer from certain non-sampling issues, such as survival/selection biases^64^ and misplacement of births^65^. We did not attempt to make corrections to data, and they were used as-is. Furthermore, retrospective birth history data will—by design—have a changing composition of maternal ages depending on the time since the survey. This was minimized by limiting retrospective trends to up to 17 years.

Although we collated a large geo-referenced database of survey data on child mortality, these data represented about 1% (1.1 million) of total deaths of children under 5 in study areas over the period. Where data do not exist or are not available in certain locations, mean estimates are informed from smoothing to nearby estimates and covariates. As such, there could be additional small-scale heterogeneity that is not picked up by our model. Wider uncertainty intervals in areas with no data account for these potential unknowns, and our 95% coverage estimates in out-of-sample predictive tests appear to be well-calibrated at the second administrative unit level. Furthermore, discrete localized mortality shock events could be missing in our analysis due to the lack of data and selection biases in surveys and censuses, and spatiotemporal smoothing. Fatal discontinuities are explicitly accounted for at the national or province level by calibration to GBD estimates. In all, 0.35% (0.4 million) of the 123 million deaths over this period were attributed to fatal discontinuities.

On the modelling side, we integrated point and areal data into a continuous model by constructing pseudo-points from areal data. Modelling approaches that integrate point and areal data as part of a joint model likelihood function are in development^66^ but are currently computationally infeasible at the large geographical scales at which we currently model. Furthermore, we divided our models into 11 regional fits (see Supplementary Fig. 3.1), as a full model that encompasses all 99 countries would be computationally infeasible due to memory constraints. Splitting up modelling in this way had the benefit of enabling parameters to vary across epidemiologically distinct world regions. A preferred model, however, would be fitted to all data simultaneously with parameters that are spatially variable.

The separable model used for age–space–time correlations is a common parsimonious assumption afforded in applying spatiotemporal geostatistical models due to efficient computation and inference; however, it yields the assumption of fully symmetric covariance. The symmetry implicit in the separable model dictates, for example, that (holding age constant for simplicity) the covariance between the observations at (location 1, time 1) and (location 2, time 2) is the same as the covariance between (location 1, time 2) and (location 2, time 1). Given our available data density in space–age–time, we believe that attempting to parameterize a more complex non-separable model would be challenging both computationally and inferentially, and it is not clear whether there would be much to benefit from the extra complications.

There are several limitations to address with respect to the use of covariates in the model. Most of the geospatial covariates that we used in the geostatistical model were themselves estimates produced from various geospatial models. Some of those estimated surfaces used covariates that were also included in our model in their estimation process. As such, we emphasize that our model is meant to be predictive, and that drawing inference from fitted coefficients across these highly correlated covariates is problematic and not recommended. Furthermore, we assumed no measurement error in the covariate values and assumed that the functional form between mortality and all covariates was linear in logit space. In certain locations, we used covariate values for prediction that were outside the observed range of the training data. As we explore in Supplementary Information section 5.4.2, however, these areas represent a relatively small proportion of the population.

Finally, we used a method for indirect estimation of SBHs that was recently developed and validated^57^. As such, indirect estimation was carried out as a pre-processing step before fitting the geostatistical model. We attempted to propagate various forms of uncertainty that could be introduced in this step, which resulted in halving the total effective sample size across all SBH data. In future, we aim to fully integrate such processing into the statistical model; such methods are in development^67^, but are not yet computationally feasible at scale.

### Reporting summary

Further information on research design is available in the Nature Research Reporting Summary linked to this paper.

## Online content

Any methods, additional references, Nature Research reporting summaries, source data, extended data, supplementary information, acknowledgements, peer review information; details of author contributions and competing interests; and statements of data and code availability are available at 10.1038/s41586-019-1545-0.

## Supplementary information

Supplementary Information Supplementary Text on data and methods, Supplementary Model descriptions, Supplementary Discussion, Supplementary References, Supplementary Figures 3.1 – 6.12, and Supplementary Tables 1.1 – 8.2.

Reporting Summary

Supplementary Information A full list of LBD Under-5 Mortality Collaborators and their affiliations.

## Data Availability

The findings of this study are supported by data that are available from public online repositories, data that are publicly available upon request of the data provider and data that are not publicly available due to restrictions by the data provider. Non-publicly available data were used under a license for the current study, but may be available from the authors upon reasonable request and with permission of the data provider. A detailed table of data sources and availability can be found in Supplementary Table 8.1. The full output of the analyses is publicly available in the Global Health Data Exchange (GHDx; http://ghdx.healthdata.org/record/ihme-data/lmic-under5-mortality-rate-geospatial-estimates-2000-2017) and can be explored using custom data visualization tools (https://vizhub.healthdata.org/lbd/under5).
