# Supplementary material for: Mapping 123 million neonatal, infant and child deaths between 2000 and 2017
Source: Nature. 2019 Oct 16;574(7778):353–8. doi: 10.1038/s41586-019-1545-0 (PMC6800389; doi:10.1038/s41586-019-1545-0)
Supplement: Supplementary file 1 — Supplementary Text on data and methods, Supplementary Model descriptions, Supplementary Discussion, Supplementary References, Supplementary Figures 3.1 – 6.12, and Supplementary Tables 1.1 – 8.2. [file 41586_2019_1545_MOESM1_ESM.pdf]

nature
SUPPLEMENTARY INFORMATION
https://doi.org/10.1038/s41586-019-1545-0

In the format provided by the authors and unedited.

Mapping 123 million neonatal, infant and child deaths between 2000 and 2017

A list of authors and their affiliations appears in the online version of the paper

Supplementary Information

Table of Contents

Supplementary Figures 3
Supplementary Tables 5
1 Guidelines for Accurate and Transparent Health Estimates Reporting (GATHER) 6
2 Methods overview 8
3 Data 10
3.1 Geographic inclusion 10
3.2 Sources and inclusion 13
3.3 Data preparation 14
3.3.1 Complete birth histories 14
3.3.2 Summary birth histories 14
3.4 Polygon resampling 22
4 Covariates and auxiliary data 22
4.1 Description of layers and justification 22
4.2 Spatial and temporal standardization 24
4.3 Administrative boundaries 26
4.4 Gridded population data 26
5 Statistical model 26
5.1 Geostatistical model 26
5.1.1 Model results 28
5.2 Post-estimation 31
5.2.1 Combining age groups 31
5.2.2 Calculating numbers of deaths 32
5.2.3 Masking grid-cell-level estimates 32
5.2.4 Summarizing results and aggregating to administrative subdivisions 32
5.2.5 Calibration with Global Burden of Disease 2017 33
5.3 Validation 40
5.3.1 Validation results 40
5.4 Other diagnostics 66
5.4.1 Comparison with previous analysis 66

5.4.2 Covariates out of range 73
5.4.3 National-level time series plots 75
5.4.4 Migration sensitivity analysis 81
5.4.5 Sources of reductions in deaths 92
5.4.6 Incorporating uncertainty into estimates of progress 95
6 Additional results 96
7 References 110
8 Data sources 113

Supplementary Figures

Supplementary Figure 3.1: Countries included in this analysis and modelling regions 10
Supplementary Figure 3.2: Survey data coverage for Africa (with Yemen), 2000–2017........................... 17
Supplementary Figure 3.3: Survey data coverage for Latin America, 2000–2017 18
Supplementary Figure 3.4: Survey data coverage for Western and Central Asia, 2000–2017 19
Supplementary Figure 3.5: Survey data coverage for Southeast Asia and Oceania, 2000–2017............... 20
Supplementary Figure 3.6: Survey data coverage for South Asia, 2000–2017... 21
Supplementary Figure 4.1: Maps of the covariates used 23
Supplementary Figure 5.1: Fitted estimates of covariate coefficients across the 12 model regions......... 30
Supplementary Figure 5.2: Posterior hyper-parameter fits across the 12 model regions 31
Supplementary Figure 5.3: Pre- and post-calibration comparisons, national level GBD, under-5............ 34
Supplementary Figure 5.4: Pre- and post-calibration comparisons, national-level GBD, infant............... 35
Supplementary Figure 5.5: Pre- and post-calibration comparisons, national level GBD, neonatal 36
Supplementary Figure 5.6: Pre- and post-calibration comparisons, subnational level GBD, under-5 37
Supplementary Figure 5.7: Pre- and post-calibration comparisons, subnational-level GBD, infant 38
Supplementary Figure 5.8: Pre- and post-calibration comparisons, subnational-level GBD, neonatal 39
Supplementary Figure 5.9: In-sample predictions versus aggregated data at the country level 42
Supplementary Figure 5.10: Out-of-sample predictions vs. aggregated data at the country level 46
Supplementary Figure 5.11: In-sample predictions vs. aggregated data at the first administrative level. 50
Supplementary Figure 5.12: Out-of-sample predictions vs. aggregated data at the first administrative level 54
Supplementary Figure 5.13: In-sample predictions vs. aggregated data at the second administrative level 58
Supplementary Figure 5.14: Out-of-sample predictions vs. aggregated data at the second administrative level 62
Supplementary Figure 5.15: Comparison between mean second administrative level estimates of under-5 mortality probability between current and previous analysis... 67
Supplementary Figure 5.16: Comparison between mean second administrative level estimates of neonatal mortality probability between current and previous analysis 68
Supplementary Figure 5.17: Relative difference between current and previous estimates of under-5 mortality probability, 2015 69
Supplementary Figure 5.18: Difference between current and previous estimates of under-5 mortality probability, 2015 70
Supplementary Figure 5.19: Second administration units with significant difference between current and previous estimates of under-5 mortality probability, 2015.. 71
Supplementary Figure 5.20: Percentage of the population living outside of the training data range of at least one covariate 74
Supplementary Figure 5.21: Grid cells with covariate values outside the range of values observed in the model training data... 75
Supplementary Figure 5.22: Raw data aggregates and estimated trends before calibration to GBD, under-5 mortality probability, 2000 to 2017 76

Supplementary Figure 5.23: Raw data aggregates and estimated trends after calibration to GBD, under-5 mortality probability, 2000 to 2017 77
Supplementary Figure 5.24: Raw data aggregates and estimated trends before calibration to GBD, infant mortality probability, 2000 to 2017 78
Supplementary Figure 5.25: Raw data aggregates and estimated trends after calibration to GBD, infant mortality probability, 2000 to 2017 79
Supplementary Figure 5.26: Raw data aggregates and estimated trends before calibration to GBD, neonatal mortality probability, 2000 to 2017 80
Supplementary Figure 5.27: Raw data aggregates and estimated trends after calibration to GBD, neonatal mortality probability, 2000 to 2017 81
Supplementary Figure 5.28: National-level trends, comparing estimates made using the full survey of respondents, versus keeping only those children born in current residence 82
Supplementary Figure 5.29: Map and scatterplot comparisons between full data and a subset of those who did not move, Bangladesh, 2007. Second administrative area R-squared for years 2000, 2010, and 2017 = 0.89, 0.96, 0.90. 83
Supplementary Figure 6.1: Second administrative subdivisions with a significant decline in neonatal mortality probability between 2000 and 2017 97
Supplementary Figure 6.2: Second administrative subdivisions with a significant decline in infant mortality probability between 2000 and 2017 98
Supplementary Figure 6.3: Second administrative subdivisions with a significant decline in under-5 mortality probability between 2000 and 2017 99
Supplementary Figure 6.4: Percentage of under-5 deaths in each bin of under-5 mortality probability that occur in the indicated world regions 100
Supplementary Figure 6.5: Posterior probability of having met the SDG 3.2 target of 12 deaths per 1,000 live births for neonatal mortality probability in 2017 101
Supplementary Figure 6.6: Under-5 mortality probability by second administrative subdivision projected to 2030 102
Supplementary Figure 6.7: Under-5 mortality probability by second administrative subdivision in 2000, normalized to the mean under-5 mortality probability within each country 103
Supplementary Figure 6.8: Under-5 mortality probability by second administrative subdivision in 2017, normalized to the mean under-5 mortality probability within each country 104
Supplementary Figure 6.9: Number of under-5 deaths, distributed across level of under-5 mortality rate in 2017 across 100 countries 106
Supplementary Figure 6.10: Point data availability per pixel by modeling region, 2000–2017 107
Supplementary Figure 6.11: Point data availability per pixel by modeling region, 2000–2017, after dropping observations 108
Supplementary Figure 6.12: Change in absolute and relative inequalities in under-5 mortality rate across second administrative level units between 2000 and 2017. 109

Supplementary Tables

Supplementary Table 1.1: GATHER Compliance 7
Supplementary Table 3.1: Countries included in the analysis (99) grouped by modelling regions. 11
Supplementary Table 4.1: Geospatial covariate citations 24
Supplementary Table 5.1: High-level summary of validation metrics by administrative level 41
Supplementary Table 5.2: High-level summary of validation metrics by age bin 41
Supplementary Table 5.3: Data sources added in SSA countries since the previous analysis 72
Supplementary Table 8.1: Data sources included in analysis 113
Supplementary Table 8.2: Data sources excluded from analysis, with rationale 187

1 Guidelines for Accurate and Transparent Health Estimates Reporting (GATHER)

Item #	Checklist item	Location
**Objectives and funding**		
**1**	Define the indicator(s), populations (including age, sex, and geographic entities), and time period(s) for which estimates were made.	Introduction
**2**	List the funding sources for the work.	Main Manuscript
**3**	Describe how the data were identified and how the data were accessed.	SI Section 3
**4**	Specify the inclusion and exclusion criteria. Identify all ad-hoc exclusions.	SI Section 6.1<br>Suppl Tables 8.1, 8.2
**5**	Provide information on all included data sources and their main characteristics. For each data source used, report reference information or contact name/institution, population represented, data collection method, year(s) of data collection, sex and age range, diagnostic criteria or measurement method, and sample size, as relevant.	Suppl Table 8.1
**6**	Identify and describe any categories of input data that have potentially important biases (e.g., based on characteristics listed in item 5).	Suppl Section 6.2
**7**	Describe and give sources for any other data inputs.	Suppl Section 4
**8**	Provide all data inputs in a file format from which data can be efficiently extracted (e.g., a spreadsheet rather than a PDF), including all relevant meta-data listed in item 5. For any data inputs that cannot be shared because of ethical or legal reasons, such as third-party ownership, provide a contact name or the name of the institution that retains the right to the data.	Suppl Table 8.1 and with IDs to links of full source metadata and download links available at www.ghdx.org
**9**	Provide a conceptual overview of the data analysis method. A diagram may be helpful.	Extended Data Figure 5
**10**	Provide a detailed description of all steps of the analysis, including mathematical formulae. This description should cover, as relevant, data cleaning, data pre-processing, data adjustments and weighting of data sources, and mathematical or statistical model(s).	Supple Sections 3, 4, 5
**11**	Describe how candidate models were evaluated and how the final model(s) were selected.	Suppl Section 5
**12**	Provide the results of an evaluation of model performance, if done, as well as the results of any relevant sensitivity analysis.	Suppl Section 5

**13**	Describe methods for calculating uncertainty of the estimates. State which sources of uncertainty were, and were not, accounted for in the uncertainty analysis.	Suppl Section 5
**14**	State how analytic or statistical source code used to generate estimates can be accessed.	Source code available here: https://github.com/ihmeuw/lbd/tree/u5m-lmic-2019
**Results and Discussion**		
**15**	Provide published estimates in a file format from which data can be efficiently extracted.	Raster and csv files to be made available on www.ghdx.org
**16**	Report a quantitative measure of the uncertainty of the estimates (e.g. uncertainty intervals).	Throughout Main Manuscript
**17**	Interpret results in light of existing evidence. If updating a previous set of estimates, describe the reasons for changes in estimates.	Introduction in main manuscript
**18**	Discuss limitations of the estimates. Include a discussion of any modelling assumptions or data limitations that affect interpretation of the estimates.	Main discussion, as well as limitations section in Methods

Supplementary Table 1.1: GATHER Compliance

2 Methods overview

Our analytical process generally followed that of our previous work to map under-5 mortality probabilities[1], diarrhea prevalence[2], child growth failure[3], and educational attainment[4] with several key exceptions and methodological advancements. Extended Data Figure 5 is a flow diagram which summarizes the analytical process we used. In this introductory section we give a broad overview of the analytical process.

The aim of this analysis was to produce joint estimates, with uncertainty, of the probability of death for children aged 0-28 days (neonates), children under 1 (infants), and children under 5, as well as estimates of the numbers of deaths for these age groups, at the subnational level for 99 low- and middle-income countries for each year from 2000 to 2017. Estimates were made using a statistical model that was continuous in space, and prediction was done at a grid-cell resolution of approximately 5 x 5 kilometers, and reported at the first and second administrative level, as well at the country level.

We use the term mortality *probability* to describe the number of deaths per live births (typically in terms of 1,000 live births). This is the quantity we model and discuss in the paper. In standard demographic notation, mortality probability for under-5s is referred to as *5q0*. Often, it is colloquially acceptable to use the term mortality *rate* for this measure, as we did in the main manuscript. These have technically different definitions; mortality rate refers to deaths per person-years lived. Since we make use of both mortality probabilities and rates in our analytical processing, for the purpose of clarity we will not use these terms interchangeably in describing our methodology.

We extracted individual records from 549 household and census sources. Records were gathered either in the form of summary birth histories (SBHs) or complete birth histories (CBHs). SBH data are at the woman level, while CBH data are at the child level. In one country we also used surveillance data (see Supplementary Section 3.3). Data preparation differed for these two types: sample sizes and number of death events over time and age could be tabulated directly for CBH data, while SBH data were prepared in accordance with indirect methods developed and validated previously by Burstein and colleagues[5]. All data observations were geo-referenced to either GPS locations (points) or areal units. Areal data were converted to pseudo-points and weighted based on spatial population distributions. Our combined global dataset contained 15.9 million records, each representing a mortality estimate (number of deaths and sample size) for a location, age, and time period. See Supplementary Section 3 for more details on data preparation.

We extracted values from each of 10 geospatial covariates at each data point. Geospatial covariates are spatial data which are represented at the 5 x 5-km grid-cell resolution. Covariates typically have global spatial coverage and values that vary each year. See Supplementary Section 4 for more information about the covariates used in this analysis.

In order to synthesize information across various sources, and to make consistent estimates across space and time, we fit discrete hazards geostatistical models to binomial data. The model explicitly accounted for variation across age bin, time, and space through inclusion of both fixed and random effects. Indicator variables for each age bin were included to form a discrete baseline mortality hazard function. Baseline hazard functions are allowed to vary in space and time in response to changing covariate values, as well a linear effect on a secular time trend. We included a Gaussian random effect across countries to account for larger-scale variations due to political or institutional effects. We also

included Gaussian random effects for each data source to account for source-specific biases. Finally, we included a Gaussian process random effect with a covariance matrix structured to account for correlation across age, time, and physical space. As such, estimates at a specific age, time, or place benefit from drawing predictive strength from data points which are nearby in any of these dimensions. We assigned priors to all model parameters and performed maximum a posteriori (MAP) inference using Template Model Builder[6] (TMB) software in R version 3.4. See Supplementary Section 5 for more details on the statistical model.

To assess the predictive performance of our model performance, we implemented a cross-validation procedure. We reran the model five times, holding out 20% of data sources each time, and produced estimates for the held-out data. Using this out of sample data as a basis for comparison, we estimated a number of predictive validity metrics including root mean squared error, correlation, mean error, and 95% coverage. See Supplementary Section 5 for more details on model performance.

From the fitted model parameters, we produce predictive posterior mortality estimates for each age group at each 5 x 5 kilometer grid cell for each year 2000 through 2017. We also supplemented these estimates with grid-cell-level population data in order to estimate the number of deaths occurring in each age group at each location in time. We ensured that at the national level, aggregated estimates for each age group and year are calibrated such that they equal estimates in the Global Burden of Disease (GBD) study. See Supplementary Section 5 for more details on post-estimation procedures.

In the subsequent sections of this document, we provide details on each step of this analytical process.

3 Data

3.1 Geographic inclusion

Countries were selected for inclusion in this analysis using the Socio-Demographic Index (SDI) published in the GBD[7]. The SDI is a measure of development that combines education, fertility, and income. We primarily aimed to include all countries in the middle, low-middle, or low SDI quintiles, with several exceptions. Brazil and Mexico were excluded despite middle SDI status due to the availability of high-quality vital registration (VR) data in these countries which have served as the basis for existing subnational estimates of child mortality. Because this study did not incorporate vital registration data sources (see “Limitations”), Brazil and Mexico were not estimated directly; instead, state-level estimates from the Global Burden of Disease 2017 study were directly substituted where appropriate. As countries with high-middle SDI status in 2017, China and Malaysia were excluded from this analysis. Albania and Moldova were excluded despite middle SDI status due to geographic discontinuity with other included countries and lack of available survey data. Libya was included despite high-middle SDI status to create better geographic continuity. Island nations with populations under 1 million were excluded because they typically lacked sufficient survey data or geographic continuity for a geospatial analytic approach to be advantageous over a national one. North Korea was excluded due to insufficient data. In all, 99 countries were included in this analysis. Supplementary Figure 3.1 shows a map of the countries included in this study, and Supplementary Table 3.2 lists the countries.

Supplementary Figure 3.1: Countries included in this analysis and modelling regions

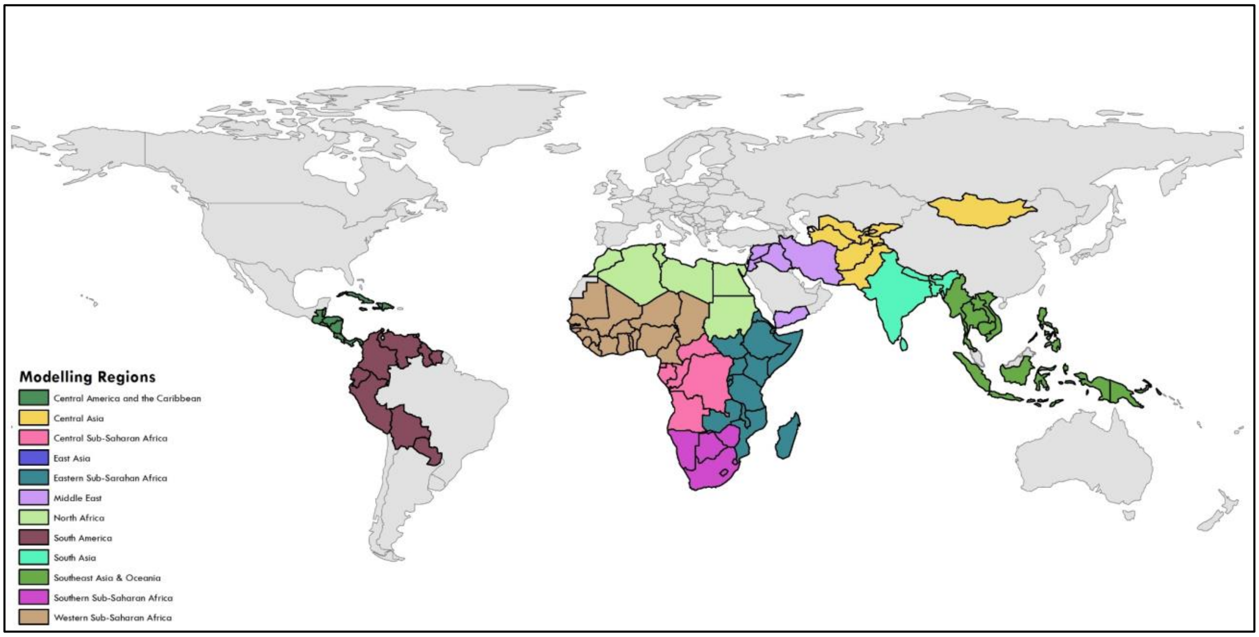

Supplementary Table 3.1: Countries included in the analysis (99) grouped by modelling regions.

Region Name	Countries	ISO3 Code
**Central America and the Caribbean**	Belize	BLZ
	Costa Rica	CRI
	Cuba	CUB
	Dominican Republic	DOM
	El Salvador	SLV
	Guatemala	GTM
	Haiti	HTI
	Honduras	HND
	Jamaica	JAM
	Nicaragua	NIC
	Panama	PAN
**Central Asia**	Afghanistan	AFG
	Kyrgyzstan	KGZ
	Pakistan	PAK
	Tajikistan	TJK
	Turkmenistan	TKM
	Uzbekistan	UZB
	Mongolia	MNG
**Central sub-Saharan Africa**	Angola	AGO
	Central African Republic	CAF
	Democratic Republic of the Congo	COD
	Equatorial Guinea	GNQ
	Gabon	GAB
	Republic of the Congo	COG
**Eastern sub-Saharan Africa**	Burundi	BDI
	Comoros	COM
	Djibouti	DJI
	Eritrea	ERI
	Ethiopia	ETH
	Kenya	KEN
	Madagascar	MDG
	Malawi	MWI
	Mozambique	MOZ
	Rwanda	RWA
	Somalia	SOM
	South Sudan	SSD
	Tanzania	TZA
	Uganda	UGA
	Zambia	ZMB
**Middle East**	Iran	IRN

	Iraq<br>Jordan<br>Palestine<br>Syria<br>Yemen	IRQ<br>JOR<br>PSE<br>SYR<br>YEM
**North Africa**	Algeria<br>Egypt<br>Libya<br>Morocco<br>Sudan<br>Tunisia	DZA<br>EGY<br>LBY<br>MAR<br>SDN<br>TUN
**South America**	Bolivia<br>Colombia<br>Ecuador<br>Peru<br>Trinidad and Tobago<br>Venezuela<br>Guyana<br>Paraguay<br>Suriname	BOL<br>COL<br>ECU<br>PER<br>TTO<br>VEN<br>GUY<br>PRY<br>SUR
**South Asia**	Bangladesh<br>Bhutan<br>India<br>Sri Lanka<br>Nepal	BGD<br>BTN<br>IND<br>LKA<br>NEP
**Southeast Asia and Oceania**	Indonesia<br>Papua New Guinea<br>Philippines<br>Timor-Leste<br>Cambodia<br>Laos<br>Myanmar<br>Thailand<br>Vietnam	IDN<br>PNG<br>PHL<br>TLS<br>KHM<br>LAO<br>MMR<br>THA<br>VNM
**Southern sub-Saharan Africa**	Botswana<br>Lesotho<br>Namibia<br>South Africa<br>Swaziland<br>Zimbabwe	BWA<br>LSO<br>NAM<br>ZAF<br>SWZ<br>ZWE
**Western sub-Saharan Africa**	Benin<br>Burkina Faso<br>Cameroon<br>Cape Verde	BEN<br>BFA<br>CMR<br>CPV

	Chad	TCD
	Côte d'Ivoire	CIV
	Ghana	GHA
	Guinea	GIN
	Guinea-Bissau	GNB
	Liberia	LBR
	Mali	MLI
	Mauritania	MRT
	Niger	NER
	Nigeria	NGA
	Sao Tome and Principe	STP
	Senegal	SEN
	Sierra Leone	SLE
	The Gambia	GMB
	Togo	TGO

3.2 Sources and inclusion

Data sources were identified with a search of the global health data exchange (GHDx: http://ghdx.healthdata.org/) with the following keywords: "complete birth history", "summary birth history", "child mortality", and "infant mortality". This search was repeated periodically to capture newly released data sources. GHDx search was accompanied by bespoke searches on country statistical websites for potential sources not listed or tagged in the GHDx. We used complete or summary birth history data contained in household surveys or censuses that were available as microdata (individual-level data) and collected from 2000 or later. We use complete birth histories only in surveys that have both complete and summary birth histories since complete birth histories are more detailed and to avoid using the same data twice. For each data source we produced a data quality report. Data sources were excluded based on screening for missingness and unexpected trends in several variables. Sources were excluded due to missingness greater than 10% in date of birth or death and children ever born or died, unrealistic geographic trends compared to other surveys in nearby country-years, inability to match the microdata to geographic locations, or non-standard methodology.

The sources included for analysis are listed in Supplementary Table 8.1. In total, we identified 209 summary birth history and 258 complete birth history data sources which were included in the analytical process. In addition, we identified 82 data sources which we ultimately chose not to include in the analysis. A list of these sources with justification notes for exclusion is available in Supplementary Table 8.2. Supplementary Figures 3.3 through 3.7 show the spatial data coverage of data by country.

3.3 Data preparation

3.3.1 Complete birth histories

Complete birth histories (CBH) capture detailed vital event histories of children, as reported retrospectively by their mothers. CBH surveys include information about the month and year of birth, as well as the age of death if applicable for each child born to responding mothers. Data on life and mortality experiences from CBH sources can be tabulated directly into discrete period and age bins, thus allowing for age- and period-specific mortality estimation, known as the synthetic cohort method[8–10]. We refer to data tabulated for a specific period and age as period-age-binned.

For this study, we used 18 annual period bins from 2000 to 2017, and seven age bins. Age bins were defined as follows: Neonatal (NN; [live birth – 1 month)), Post-neonatal 1 (PNN1; [1 month – 6 months)), Post-neonatal 2 (PNN2; [6 months – 1 year)), 1 year olds (1yr; [1 year – 2 years)), 2 year olds (2yr; [2 years – 3 years)), 3 year olds (3yr; [3 years – 4 years)), 4 year olds [4 years – 5 years)). Thus, one child can supply information toward up to seven age bins. Each child entering a period-age bin counts toward the sample size of that age bin (unless the child entered the age bin more recently than the length of the age bin, in which case they were censored). If a child died within a period-age bin, that death is recorded as occurring in that bin. The ratio of deaths within to number entering can thus be considered an estimate of the probability of death in that age bin, conditional on entering the age bin. This is the same as the lifetable quantity q_a for age bin a.[11]

For survey clusters, we tabulated deaths and number entering directly. For areal units, we used survey weights, if provided, to estimate a mortality probability for each component period-age bin, which was then multiplied by the sample size of the period-bin within the areal unit to get an estimate of numbers of deaths.

3.3.2 Summary birth histories

Summary birth histories (SBHs) are widely collected in censuses and household surveys. In an SBH, a respondent (typically the mother), is asked about how many live births she has had (children ever born, CEB), and how many of her children have died (CD). There is no additional information given about the timing of births or deaths, and as such, an additional inferential step is needed in order to estimate mortality probabilities and assign them to specific time period. This step is broadly referred to as indirect estimation.[11]

We prepared summary birth histories in accordance with the methods detailed in the paper by Burstein and colleagues.[5] We describe the method briefly here but refer readers to that paper for details.

A discrete time generalized additive hazard model was trained using individual-level data from 243 Demographic and Health Surveys (DHSs). The model was fit on CBH data but using covariates also available from SBH data (mother's age, CEB, CD/CEB, and national-level covariates). SBH data were then applied to the fitted model to make predictions of discrete hazard curves for all hypothetical children

potentially ever born to mothers reporting in SBH. Discrete hazard curves were set up to have breaks at the same seven age bins used for tabulation of CBH data. Each of these hypothetical children is given a weight based on their probability of birth (given their mother's age, total fertility, and region of residence). Hazard curves were turned into age-period-specific estimates of mortality by taking weighted means across all hypothetical children living in a survey cluster or administrative area. In addition, weights were summed over these area-age-bins to approximate sample sizes.

3.3.2.1 Calculating effective sample sizes for summary birth history observations

To account for the fact that summary birth history data arose from modelled indirect estimates, we used a three-step simulation procedure to calculate an effective sample size for each SBH data point based on uncertainty from predictions and out-of-sample uncertainty as calculated by Burstein and colleagues[5].

We applied the age-specific indirect estimation method described by Burstein and colleagues to all 209 SBH datasets used in the paper, producing an estimate of mortality probability ($\hat{p}$) and sample size ($\hat{N}$) for each area-age-period bin from each survey (notated as s, a, t, and k, respectively). We refer to areas as the smallest available geography from each survey, typically a cluster or a second administrative or first administrative unit. Sample sizes represented estimates of the number of children expected to enter each area-age-period bin, estimates of $\hat{N}$ were deterministic based on Burstein and colleagues' method, but $\hat{p}$ arose from a statistical model, and thus uncertainty in fitted model parameters was propagated into estimates of $\hat{p}$. For each $\hat{p}_{k,a,s,t}$ we extracted 1000 predictive draws from the model by simulating from a multivariate Gaussian distribution using the mean vector and variance-covariance matrix of all estimated model parameters. Furthermore, modelled estimates could deviate from the truth for various reasons not captured in model uncertainty. This added variance can be estimated by comparing model estimates to out of sample empirical validation data. This procedure was done as part of the subnational validation by Burstein and colleagues where they performed leave-one (survey)-out validation for each of the 243 DHSs used to train the complete model. To account for this additional error within each age bin, we first estimated the error with the relative residual error terms (cv) for each of the seven age bins. The value cv_a for each of the seven age-bins was calculated across all surveys as $SD\left(\text{logit}(\hat{p}_{k,a,t}) - \text{logit}(p^{oos}_{k,a,t})\right)/\text{mean}(\text{logit}(\hat{p}_{k,a,t}))$, where $\hat{p}_{k,a,t}$ is the model estimate for age bin a from survey k and $p^{oos}_{k,at}$ is the empirical estimate for the same survey held out of sample (i.e., the validation data).

In the first step, we incorporated this out of sample residual error into each estimate of $\hat{p}_{k,a,s,t}$ via simulation. This was done for each draw of each area-age-bin estimate, in logit space to constrain estimates from 0 to 1, by simulating: $\text{logit}(\hat{p}^{\text{inflated}}_{k,a,s,t}) \sim \text{Normal}(\text{logit}(\hat{p}_{k,a,s,t}), \text{logit}(\hat{p}_{k,a,s,t}) * cv_a)$.

In the second step we simulated numbers of deaths for each area-age-period bin for each of the 1000 draws: $\hat{Y}^{\text{inflated}}_{k,a,s,t} \sim \text{Binomial}(\hat{p}^{\text{inflated}}_{k,a,s,t}, \hat{N}^{\text{inflated}}_{k,a,s,t})$.

In the third step, we calculated the effective sample size for each area-age-period bin by assuming that $\hat{Y}^{\text{inflated}}_{k,a,s,t}$ arose from a beta-binomial distribution. The beta-binomial distribution was chosen because it assumes a fixed sample size ($\hat{N}$), but a variable probability, accounting for model and residual uncertainty we had incorporated into $\hat{p}^{\text{inflated}}_{k,a,s,t}$ (and thus $\hat{Y}^{\text{inflated}}_{k,a,s,t}$) via simulation in the first two steps. In our geostatistical model (see Supplementary Section 5), we use a binomial data likelihood, so our

interest was in finding an effective sample size for a binomial distribution with the same variance as a beta-binomial distribution for each area-age-period observation. Across the draws for each $\hat{Y}_{k,a,s,t}^{inflated}$ we first calculated the beta-binomial parameters $\hat{\alpha}_{k,a,s,t}$ and $\hat{\beta}_{k,a,s,t}$ using method of moments. We then used the variance equation for a beta-binomial to calculate the variance as $\mathrm{Var}(\hat{Y}_{k,a,s,t}^{inflated}/\hat{N}_{k,a,s,t}) = \frac{\hat{\alpha}_{k,a,s,t}\hat{\beta}_{k,a,s,t}(\hat{\alpha}_{k,a,s,t}+\hat{\beta}_{k,a,s,t}+\hat{N}_{k,a,s,t})}{(\hat{\alpha}_{k,a,s,t}+\hat{\beta}_{k,a,s,t})^2(\hat{\alpha}_{k,a,s,t}+\hat{\beta}_{k,a,s,t}+1)\hat{N}_{k,a,s,t}}$. We then asked the question: if this data had been observed from a true binomial distribution, what would the effective sample size, $N_{bin_{k,a,s,t}}$, have been to see as much variation in $\hat{p}_{k,a,s,t} = \hat{Y}_{k,a,s,t}^{inflated}/\hat{N}_{k,a,s,t}$ as was seen under the beta-binomial setting? To answer this question and to solve for $N_{bin_{k,a,s,t}}$, we set the variance of an estimator for the probability of success of a binomial distribution, $\frac{\hat{p}_{k,a,s,t}(1-\hat{p}_{k,a,s,t})}{N_{bin_{k,a,s,t}}}$, equal to the beta-binomial variance shown above. Setting $\frac{\hat{\alpha}_{k,a,s,t}\hat{\beta}_{k,a,s,t}(\hat{\alpha}_{k,a,s,t}+\hat{\beta}_{k,a,s,t}+\hat{N}_{k,a,s,t})}{(\hat{\alpha}_{k,a,s,t}+\hat{\beta}_{k,a,s,t})^2(\hat{\alpha}_{k,a,s,t}+\hat{\beta}_{k,a,s,t}+1)\hat{N}_{k,a,s,t}} = \frac{\hat{p}_{k,a,s,t}(1-\hat{p}_{k,a,s,t})}{N_{bin_{k,a,s,t}}}$ and rearranging the equations, we arrive at the equation for the effective sample size for each binomial SBH observation (suppressing k, a, s, t subscripts for simplicity): $N_{bin} = N_{eff} = \frac{\hat{p}(1-\hat{p})(\hat{\alpha}+\hat{\beta})^2(\hat{\alpha}+\hat{\beta}+1)\hat{N}}{\hat{\alpha}\beta(\hat{\alpha}+\hat{\beta}+\hat{N})}$.

Via this procedure, 31% of SBH observations had an N_{eff} that was larger than $\hat{N}$. This was due to practical constraints of having small mortality probabilities and small sample sizes, which can lead to unstable simulation estimates. For these observations, we set $N_{eff} = \hat{N}$. Since this was predominantly a problem of small sample sizes, this only affected 1.7% of the data weighted by sample size. The sum of all $\hat{N}$ across all SBH data was 210 million, and the sum of all N_{eff} was 123 million. Thus, accounting for model variance and out of sample residuals reduced total SBH sample size by 41%.

Supplementary Figure 3.2: Survey data coverage for Africa (with Yemen), 2000–2017

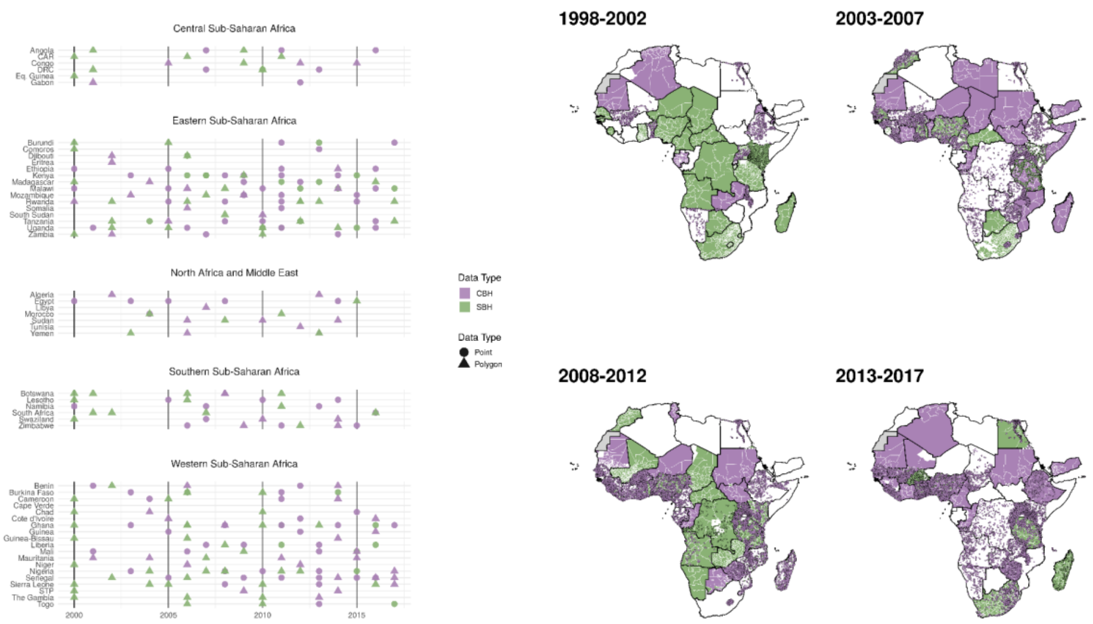

Supplementary Figure 3.3: Survey data coverage for Latin America, 2000–2017

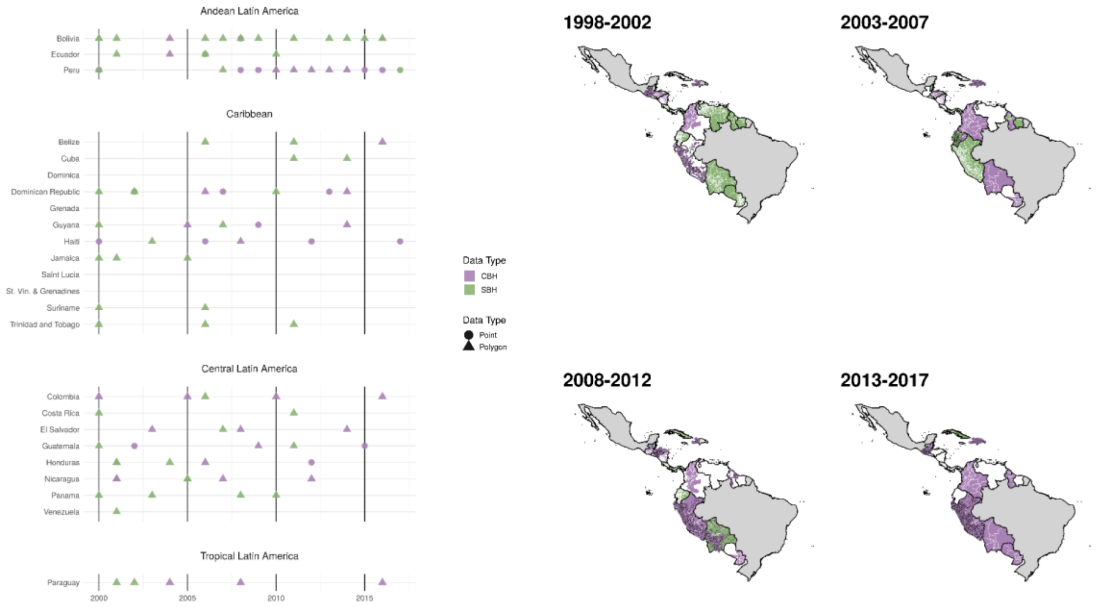

Supplementary Figure 3.4: Survey data coverage for Western and Central Asia, 2000–2017

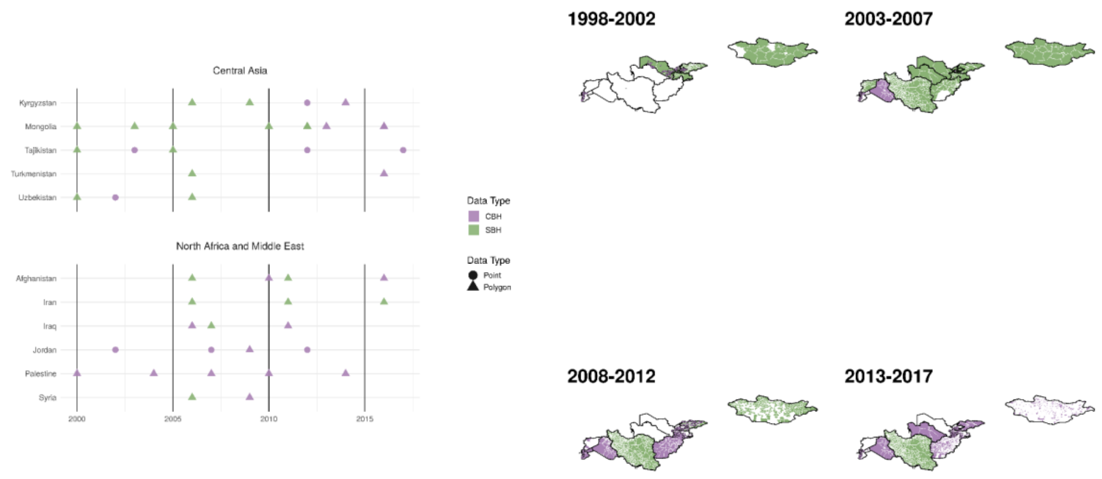

Supplementary Figure 3.5: Survey data coverage for Southeast Asia and Oceania, 2000–2017

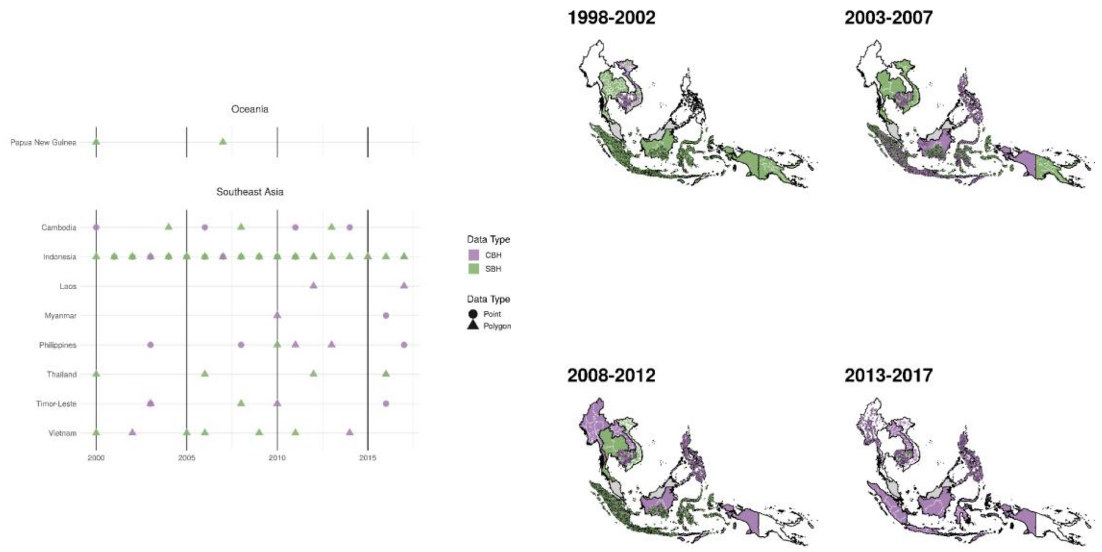

Supplementary Figure 3.6: Survey data coverage for South Asia, 2000–2017

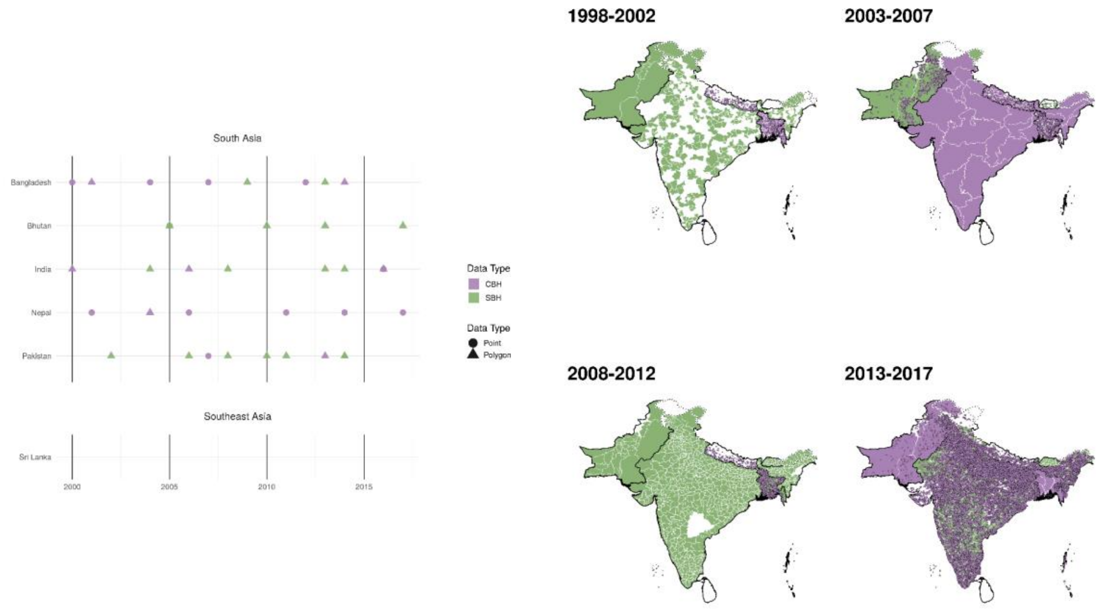

3.4 Polygon resampling

We matched all data to GPS coordinates (latitude and longitude) wherever possible. We refer to these precisely located data as 'point data'. In cases where point data were not available, we matched data points to the smallest possible areal unit (also referred to as polygons). In most cases these polygons represent administrative sub-divisions. Since the geostatistical model we fit (see Supplementary Section 5) requires point data, we re-sampled polygon data into pseudo-point data. This approach has been used before in mapping studies of child mortality[1], child growth failure[3], education[4], and diarrhea burden[2].

The approach to producing pseudo-points proceeded as follows for each polygon-level observation: we sampled 10,000 locations with weights proportional to the underlying population (as measured by WorldPop, see Supplementary Section 4.4) at 1 x 1-kilometer spatial resolution. We then used k-means clustering to derive a reduced set of points, with k set to 1 per 1000 grid cells. Each of these resulting clusters then served as pseudo-point data. Each pseudo-point was assigned a weight proportional to the number of sampled locations contained in it via k-means clustering. The observed mortality probability for the polygon as a whole was assigned to each point, and the sample size for each point was taken as the sample size for the polygon multiplied by the weight. The sample sizes for all pseudo-points derived from a polygon thus sum to equal the sample size of the polygon as a whole.

4 Covariates and auxiliary data

4.1 Description of layers and justification

We used geospatial covariates to help improve predictions in places without observed data. Images of each covariate at one time-point are available in Supplementary Figure 4.1. We chose covariates that had known associations with child mortality. These included socio-demographic and environmental measures. We also included covariates representing health outcomes which directly contribute to mortality.

We included the following socio-demographic and environmental covariates: Travel time to nearest inhabited area of 50,000 or more, which serves as a proxy for remoteness from services[12]; intensity of lights at night, which serves as a measure of electricity consumption and economic development[13,14]; mean years of educational attainment by women of reproductive age[15,16]; the mass per cubic meter of air of particles with a diameter less than 2.5 micrometers ($PM_{2.5}$)[17]; ratio of children under 5 to number of women of reproductive age (fertility proxy)[18,19]; the total population; and urbanicity[20].

We also included the following health-related covariates: proportion of children aged 12-23 months who have received the third dose of diphtheria-pertussis-tetanus vaccine[21], which also serves as a proxy for routine health service utilization in children[22,23]; incidence rate of *Plasmodium falciparum* malaria in children under 5[24,25]; and prevalence of stunting in children under 5[26–28].

Supplementary Figure 4.1: Maps of the covariates used

1: Travel time to nearest inhabited place with population > 50,000. 2: Light intensity at night. 3: Coverage of DPT3 vaccine in children under 2. 4: Average years of education for women of reproductive age. 5: Fertility ratio. 6: Urban areas. 7: $PM_{2.5}$. 8: Pf Malaria Incidence rate. 9: Stunting prevalence in children under 5. 10: Log of total population. For time-varying covariates, the most recent year of available data is mapped here.

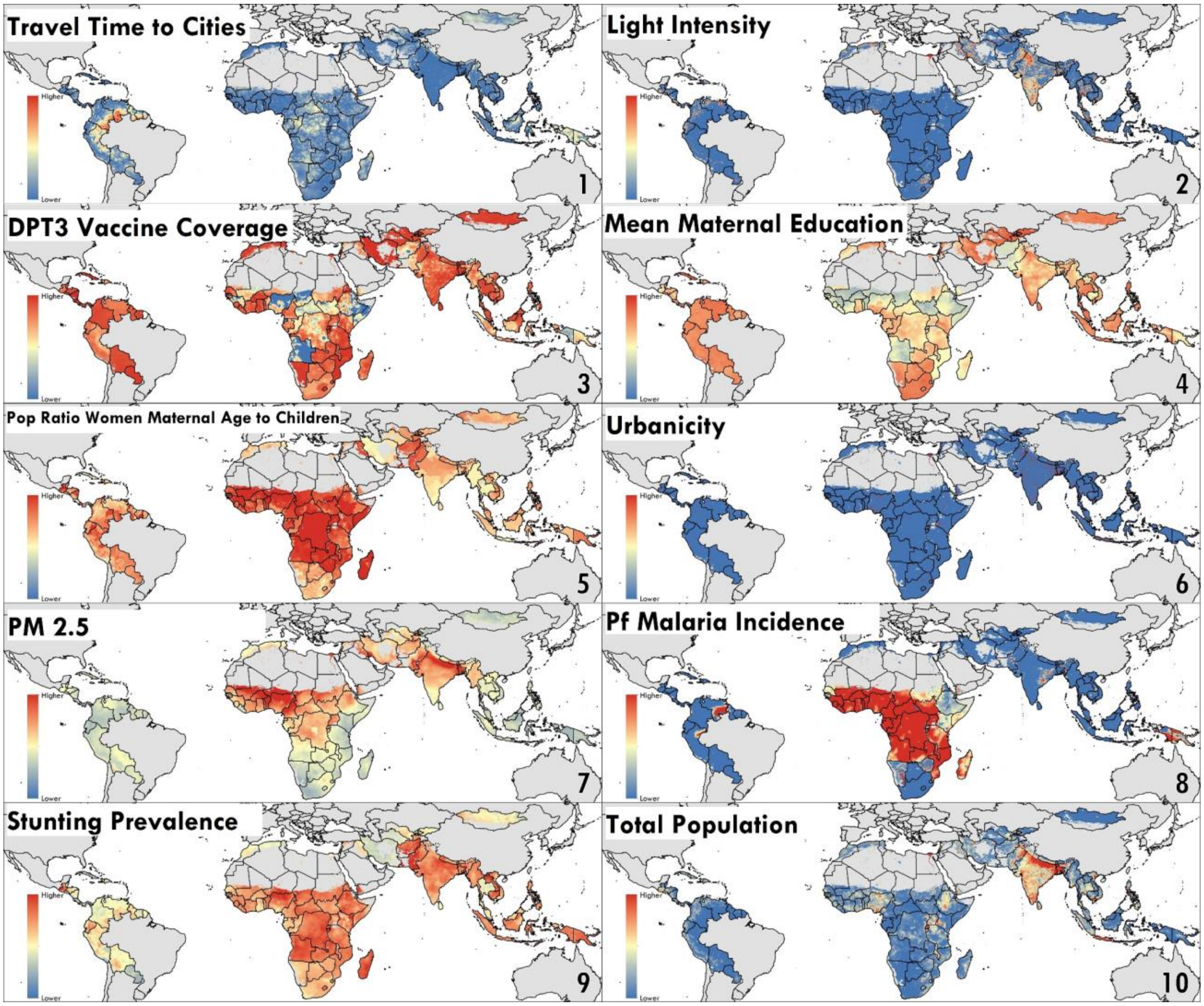

4.2 Spatial and temporal standardization

Raw data from spatial covariates came in varying spatial and temporal resolutions. All covariates were prepared to a standardized format and aligned to a 5 x 5-km global annual grid. If raw input resolution was finer than 5 x 5-km we resampled the raster by taking either a neighborhood average or sum (in the case of count covariates, such as population).

If covariates were reported in time intervals coarser than year we interpolated between years. Several covariates were not available out to 2017, and for these we completed the time series by carrying forward data available for the most recent year. Travel time to cities was synoptic and thus did not vary over time. For this covariate no temporal adjustments were made and it was used as non-temporally changing in the model.

Some covariates did not have full global coverage. For each of the 11 model regions used in this analysis, we first checked if each covariate had full geographic coverage in the region. If the covariate did not cover the region, it was not used.

Supplementary Table 4.1: Geospatial covariate citations

Citations for externally sourced covariates. Covariates prevalence of diarrhea, coverage of DPT3 vaccine in children under 5, average years of education for women of reproductive age, and prevalence of stunting in children under 5 are all modeled by the Institute for Health Metrics and Evaluation.

Covariate	Source	Paper Citation	Dataset Citation	Processing
access2 (Travel time to nearest settlement)	Oxford	Weiss, D. J. *et al.* A global map of travel time to cities to assess inequalities in accessibility in 2015. *Nature* **533**, 333-336 (2018).	Available at: https://map.ox.ac.uk/research-project/accessibility_to_cities/	Raster resampled using bilinear interpolation to standard 5 km spatial resolution.
dmspntl (Nighttime lights)	NOAA DMSP satellite program (derived)		Available at: https://www.ngdc.noaa.gov/eog/dmsp/downloadV4composites.html	Rasters resampled using bilinear interpolation to standard 5 km spatial resolution.

fertility	WorldPop (derived)	Lloyd, C. T., Sorichetta, A. & Tatem, A. J. High resolution global gridded data for use in population studies. *Sci. Data* **4**, sdata20171 (2017).	Available at: http://www.worldpop.org.uk/data/get_data/. (Accessed: 25th July 2017)	fertility = (M04 + F04) / WOCBA where:<br>M04 = males, 0-4 years old<br>F04 = females, 0-4 years old<br>WOCBA = women of child bearing age (females 15-49 years old) - from WorldPop<br>5-year rasters were interpolated using an exponential growth rate to produce annual rasters. 1 km raw data were aggregated to 5 km spatial resolution, preserving total population sums. Once converted to fertility, those areas with a population of zero were filled with the nearest neighboring fertility value where the original population was > 0.
ghslurbanicity (Urbanicity)	European Commission/ GHS	Pesaresi, M. et al. Operating procedure for the production of the Global Human Settlement Layer from Landsat data of the epochs 1975, 1990, 2000, and 2014. (Publications Office of the European Union, 2016).	Available at: http://ghsl.jrc.ec.europa.eu/data.php	Rasters resampled using bilinear interpolation to standard 5 km spatial resolution. For intervening data years, the closest available data year was used.
ihmepm25 (ambient air pollution, particulate matter 2.5)	IHME GBD		Data integration model available at: https://arxiv.org/abs/1609.00141	Rasters resampled using bilinear interpolation to standard 5 km spatial resolution.
map_pf_incidence (Malaria incidence)	The Malaria Atlas Project	Gething, P. W. *et al.* Mapping Plasmodium falciparum Mortality in Africa between 1990 and 2015. *N. Engl. J. Med.* **375**, 2435–2445 (2016).	Available at: http://www.map.ox.ac.uk/	Rasters resampled using bilinear interpolation to standard 5 km spatial resolution.
worldpop (Population)	WorldPop	Lloyd, C. T., Sorichetta, A. & Tatem, A. J. High resolution global gridded data for use in population studies. *Sci. Data* **4**, sdata20171 (2017).	World Pop. Get data. Available at: http://www.worldpop.org.uk/data/get_data/. (Accessed: 25th July 2017)	5-year rasters were interpolated using an exponential growth rate to produce annual rasters. 1 km raw data were aggregated to 5 km spatial resolution, preserving total population sums.

4.3 Administrative boundaries

All country-level and first and second administrative-level boundaries used in this analysis came from the Database of Global Administrative Areas (GADM) version 3.6. GADM shapefiles are available to download from https://gadm.org/.

First administrative-level boundaries were further edited to match boundaries used for subnational estimation in the Global Burden of Disease (GBD) in cases where GADM did not match the GBD geographies. This affected India and Indonesia. Six separate first administrative units were merged into one unit called "The Six Minor Territories" in India. In Indonesia, Kalimatantan Timur was split into two provinces: Kalimantan Timur (South) and Kalimantan Utara (North).

Finally, in India, district (second administrative subdivision) boundaries were edited to include the latest district divisions up to December 2018 using a shapefile from ML_INFOMAP (www.mlinfomap.com).

4.4 Gridded population data

We used WorldPop (http://www.worldpop.org.uk/) as a source for all gridded population data used throughout this analysis, including as a covariate in modelling and for taking population weighted averages at areal units. Except for use as a model covariate and for polygon resampling, where we used gridded total population, we used gridded population for the under-5 age band (see for example our discussion of post estimation in Supplementary Section 5.2). WorldPop provides gridded population estimates at the 1 x 1-km spatial resolution and at five-year intervals, so we resampled the raster by taking a zonal sum to reach the 5 x 5-km resolution we used for analysis and results. We interpolated between years using an exponential growth rate.

5 Statistical model

5.1 Geostatistical model

For each modeling region, we assume a discrete hazards model, with a baseline hazard function varying across the seven age bins, as described in Supplementary Section 3.3. Age bins are (in months): NN: [0-1), PNN1: [1-6), PNN2: [6-12), 1yr: [12-24), 2yr: [24-36), 3yr: [36-48), 4yr: [48-60).

Each child recorded in CBH data is counted as entering a period-age bin (an age band within a calendar year), and a death event for a given child is assigned if they died within a period-age bin. We counted the number of children entering into, N, and dying within, Y, for each period-age bin from each point location in each survey k within each country c in the data. Likewise, these same variables are estimated indirectly for each point in SBH data as described in Supplementary Section 3.3.

The number of deaths for children in age band a in year t at point location s was assumed to follow a binomial distribution:

$$Y_{a,s,t} \sim Binomal(N_{a,s,t}, p_{a,s,t})$$

Where $p_{a,s,t}$ can be interpreted as the probability of death in the age bin, conditional on survival to that age bin for a particular space-time location. Using a generalized linear regression modelling framework, a logit link function is used to relate p to a linear combination of effects:

$$Logit(p_{a,s,t}) = \beta^0 + \sum_{a=2}^{7} I_a\beta_a^1 + \beta^2 X_{s,t} + \beta^3 t + \nu_{c[s]} + \nu_{k[s]} + Z_{a,s,t}$$

The first term β^0 is an intercept, representing the mean for the first age band when all covariates equal zero, while $I_a\beta_a^1$ are fixed effects for each age band, representing the mean overall hazard deviation for each age band from the intercept. $\boldsymbol{\beta^2 X_{s,t}}$ are the effects of geospatial covariates, which we described in detail in Supplementary Section 4. All geospatial covariates were centered and scaled by subtracting their mean and dividing by their standard deviations. $\beta^3 t$ is an overall linear temporal effect to account for broad secular trends. Each ν term represent uncorrelated random effects: $\nu_{c[s]} \sim Normal(0, \sigma_c^2)$ is a country-level random effect applied to all locations, s, within a country; $\nu_{k[s]} \sim Normal(0, \sigma_k^2)$ is a data source-level random effect for the survey k from which the data at location s was observed. Survey-level random effects were used to account for systematic variation or biases across sources and were included in model fitting but not in prediction from fitted models.

The term $Z_{a,s,t} \sim Gaussian\ Process(0, \boldsymbol{K})$ is a correlated random effect across age, space, and time and is modeled as a four-dimensional mean zero Gaussian process with covariance matrix $\boldsymbol{K}$. This term accounts for structured residual correlation across these spatial-age-temporal dimensions that are not accounted for by any of the other model's fixed or random effects. This structure was chosen because the hazard for each age group is expected to vary in space and time, and such spatiotemporal correlation are likely to be similar across ages. $\boldsymbol{K}$ is constructed as a separable process across age, space, and time: $\boldsymbol{K} = \boldsymbol{\Sigma_a} \otimes \boldsymbol{\Sigma_t} \otimes \boldsymbol{\Sigma_s}$. The continuous spatial component is modeled with a stationary isotropic Matérn covariance function: $\text{cov}_s(d_s) = \frac{2^{1-\nu}}{\tau\Gamma(\nu)}\left(\sqrt{2\nu}\frac{d_s}{\kappa}\right)^{\nu} K_\nu\left(\sqrt{2\nu}\frac{d_s}{\kappa}\right)$, where K_ν is the modified Bessel function of the second kind. The Matérn function has three hyperparameters: κ, τ, and ν; the parameter ν is fixed at 1 and κ and τ are fitted in the model. The overall amplitude of the process is determined by the marginal variance, $1/\tau > 0$ while the distance required between two spatial locations, $d_s = |s_i - s_j| > 0$, before their correlation drops below any specific threshold is governed by the scaling parameter, $\kappa > 0$. The age and temporal effects were each assumed to be discrete auto-regressive order 1 (AR1) processes where the discrete steps are taken annually in time and across the seven age groups. The AR1 covariance functions are each defined by single correlation parameters: ρ_a and ρ_t for age and time, respectively.

We specified the following priors for model parameters:

$$\log(\kappa) \sim Normal(0,1)$$

$$\log(\tau) \sim Normal(0,1)$$

$$\log\left(\frac{1+\rho_a}{1-\rho_a}\right) \sim Normal(1,1)$$

$$\log\left(\frac{1+\rho_t}{1-\rho_t}\right) \sim Normal(1,1)$$

$$\beta^{0,1,2,3} \sim Normal(0,9)$$

$$\log(\sigma_c) \sim Normal(-4, 4)$$

$$\log(\sigma_k) \sim Normal(-4, 4)$$

We used transformations on priors in order to perform optimization on a likelihood and maintain constrained parameters. Log-transforms were used to constrain certain parameters ($\kappa, \tau, \sigma_c, \sigma_k$) positive. Transformations on the ρ parameters constrained values between -1 and 1 and centered at zero. Priors on all fixed effects were weakly informative, since covariates were centered and scaled. Priors for the standard deviation of Gaussian random effects (σ_c, σ_k) were structured such that they had a small mean and a long tail because we thought these effects would be minimal considering other model components, but did not want to completely constrain their size. The models were fit using Template Model Builder[6] (TMB) package in R version 3.4. We used maximum a posteriori (MAP) inference, using a maximum likelihood estimation with an augmented optimization objective (log-likelihood function) which incorporated prior distributions for all model parameters. TMB uses automatic differentiation to find the Laplace approximation to the marginal log-likelihood with respect to the hyperparameters of any random effects specified in the model. A non-linear optimization routine is then used to maximize the marginal log-likelihood to derive point estimates for all model parameters. The $Z_{a,s,t}$ random effects were fitted using the stochastic partial differential equations[29] approximation to Gaussian Process residuals. We constructed a finite elements mesh for the SPDE approximation based on a polygon boundary defining the spatial limits of the modeling region. The mesh had a minimum edge length of 100 kilometers over land. Finally, a generalized delta-method is used to approximate the joint precision matrix of all model parameters.

Using the joint precision matrix and point estimates, we generated 1000 draws from all model parameters using a multivariate-normal approximation. These model parameter draws were used to predict corresponding draws of mortality probabilities across all age groups for each grid cell in each year. In other words, for each age bin in each year we estimated 1000 surfaces of mortality probability estimates, each surface corresponding to one draw from the posterior parameter estimates. Within each surface, or "candidate map", the correlation structure across space-age-time is maintained (for a detailed discussion, see Patil and colleagues[30]).

Separate models were fitted for each of 11 global regions (see map in Supplementary Figure 3.1 and country list in Supplementary Figure 3.1). Splitting up modeling in this way was done for two reasons. First, it was not computationally feasible to fit a single global model. Second, fitting by regions allows for variation in fitted parameters across epidemiologically distinct regions.

5.1.1 Model results

Coefficient values for each model region are shown in Supplementary Figure 5.1. These are exponentiated coefficients from a model with a logit link and as such should be interpreted as odds ratios. Geospatial covariates enter the model as centered and scaled by the mean and standard deviations, as such coefficients can be interpreted as the odds ratio for a one standard deviation

increase in covariate value. The horizontal black line on the figure indicates an odds ratio of 1, or no effect on mortality. Any effect size below the line can be interpreted as protective, and effects above the line are associated with increased mortality. We emphasize that, due to high correlation and issues of circularity in production of covariate surfaces, drawing inference on effect sizes or directions is not recommended.

Posterior values for all model hyper-parameters are shown in Supplementary Figure 5.2. Variation in country random effects was substantial in most regions, indicating that there were significant country-level intercept shifts that were not accounted for by spatial covariates alone. Variation in country random effects was consistently larger than variation in the data source-specific random effects, except for the North Africa region. Data-source-specific random effects account for systematic bias across sources and were not included in predictions. We also show posterior estimates for the four parameters guiding the Gaussian Process random effect. The ρ parameter for age was always above 0.75 and the ρ parameter for year was always above 0.98, indicating high correlation across age bins, and very high correlation across annual time steps. This was expected given the way that data were prepped, where a single data source contributes a time trend of information for all the different age bins.

Supplementary Figure 5.1: Fitted estimates of covariate coefficients across the 12 model regions

All fixed effect parameter estimates are exponentiated. Geospatial covariates enter the model as centered and scaled by their standard deviations. Additional fixed effects not included in this plot are the overall intercept and age-bin-specific intercepts.

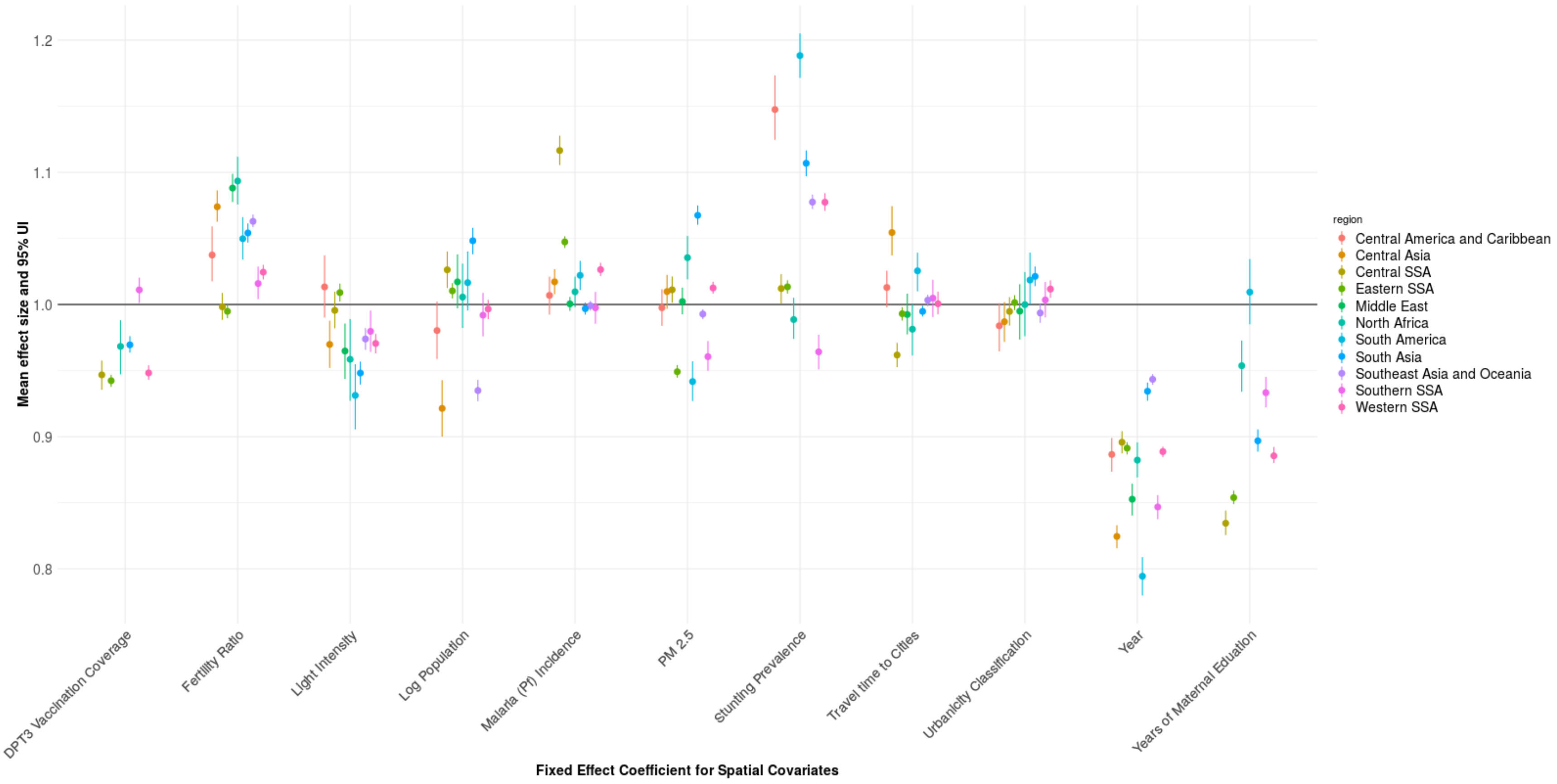

Supplementary Figure 5.2: Posterior hyper-parameter fits across the 12 model regions

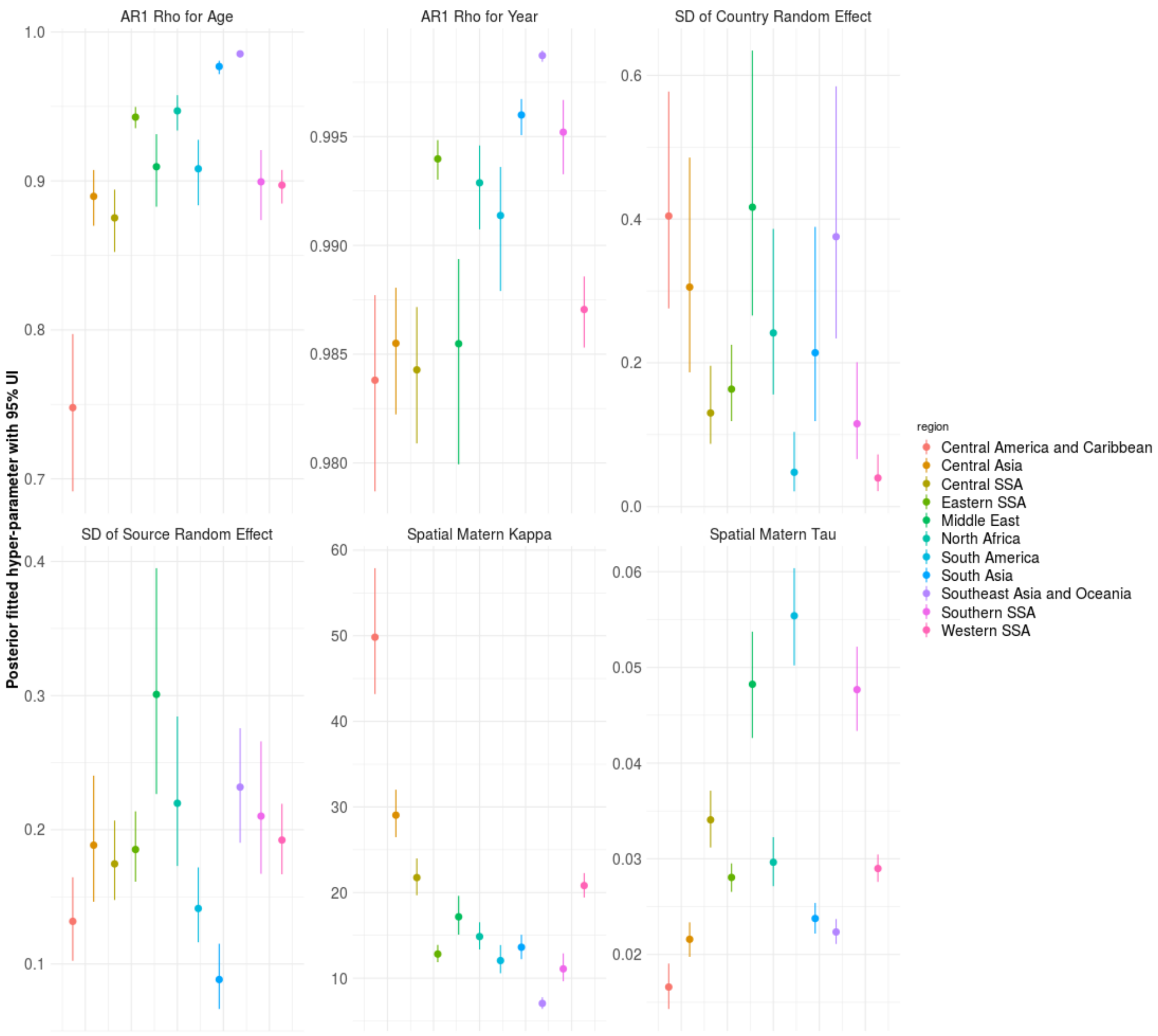

5.2 Post-estimation

5.2.1 Combining age groups

For reporting in this paper, we focus on presenting results for three age bands of mortality: neonatal, infant, and under-5. Neonatal is taken as the first age band predicted from the model, and infant and under-5 are derived from a combination of age bands using the synthetic cohort approach to estimating period-specific mortality probabilities. All aggregation across ages was done at the draw level for each

space-time grid cell. For each space-time grid cell (gc) and posterior predictive draw (m) of estimated mortality probability $\hat{p}^{m,gc}$, infant mortality ($1\hat{q}0^{m,gc}$) is calculated as $1-\prod_{a=1}^{3}(1-\hat{p}_a^{m,gc})$, and under-5 mortality ($5\hat{q}0^{m,gc}$) is calculated as $1-\prod_{a=1}^{7}(1-\hat{p}_a^{m,gc})$. Since these calculations are done at the grid-cell-draw level, correlation structure within each draw is maintained by this processing.

5.2.2 Calculating numbers of deaths

In order to produce estimates of death counts, we first converted each grid-cell-draw estimate of mortality probability, q, predicted directly from the model (and aggregated into the three age bins we report) into estimates of yearly mortality rates, m. For infants and under-5s we used the following formula[11]: $m = 1/(-n + a + n/q)$, where n is the width of the age band (1 and 5 for infant and under-5, respectively), and a is the average number of years lived by those who died within the age band. For a we used the country-year-specific estimate produced as part of the GBD study[7]. We calculated m in the neonatal band as $-12 * log(1-q)$.[11] For provisional estimates of deaths, we multiplied m at each age-space-time-draw by high-resolution population estimates for under-5s available from the WorldPop project. WorldPop does not publish estimates of neonatal or infant population, so we assumed within-country relative spatial distributions of populations in those age bins equivalent to those seen across the broader under-5 populations. Furthermore, WorldPop does not publish uncertainty around their grid-cell-level population estimates, so we were unable to propagate population count uncertainty into our estimates of death counts. Finally, we scaled grid-cell-level death counts by national death count estimates available from the GBD (see Supplementary Section 5.2.5 for more details on calibration to GBD).

5.2.3 Masking grid-cell-level estimates

Although the model can predict at all locations covered by available raster covariates, all final model outputs for which land cover was classified as “barren or sparsely vegetated” or “snow and ice” on the basis of MODIS satellite data (2013) and where the total population density was less than 10 individuals per 1 × 1 km grid cell in 2015 were masked from improved understanding when communicating with data specialists and policymakers.

5.2.4 Summarizing results and aggregating to administrative subdivisions

Summary maps for probabilities of death and death counts were produced by taking summary statistics across draws at either the grid cell or aggregated levels. For example, mean grid-cell-level maps (such as Figure 1c) were derived by taking the mean across all draws in each age-time grid cell, while uncertainty intervals were derived from the 2.5th and 97.5th percentiles at each age-time grid cell.

Spatially aggregated estimates, such as those at the country and first- and second-administrative areal unit were made by taking population weighted averages (using WorldPop gridded population data) of the value of interest for each draw across each areal unit, such that we produced draw-level estimates for each areal unit. Grid cells were assigned to an area based on the location of their respective centroids. These were subsequently summarized across draws. In places where borders intersected grid cells, death counts in each grid cell were split based on the share of land area in each adjacent area.

5.2.5 Calibration with Global Burden of Disease 2017

We performed a calibration of our estimates to national and subnational estimates from the GBD 2017 study[7]. This allowed us to take advantage of the national-level information such as vital registration data which is used as part of the GBD estimation process but which we were unable to currently use in our geostatistical model. To do so, we first assigned each grid cell to a country based on the location of the grid cell centroid. We then generated mean estimates for each country-age-year based on the aggregation approach described above in Supplementary Section 5.2.4. For each country-age-year estimate, we generated a scaling factor defined as the ratio between the GBD estimate and the aggregated mean national estimate from our model. These scaling factors were applied to each grid-cell draw, ensuring that aggregated mean estimates from our model were identical to the mean estimate from the GBD 2017 study.

The relationship between our aggregated national-level predictions (i.e., pre-calibration predictions) and those from the GBD 2017 are compared in Supplementary Figures 5.3-5.5. Overall, the median scaling factor was 0.99 for neonates, 1.02 for infants, and 1.03 for under-5s, and the inter-quartile ranges were 0.87–1.09, 0.91–1.11, and 0.92–1.12 for the neonates, infants, and under-5s, respectively, indicating close agreement with between the aggregated geospatial estimates and national-level estimates produced for the GBD 2017 study. Disagreement was evident in countries such as Costa Rica and Thailand, where GBD estimates are largely informed by vital registration data which disagree with concurrent survey data sources utilized in our model. We also saw disagreement in countries such as Haiti in 2010, where the mortality shock from earthquake was incorporated into the GBD estimate via the fatal discontinuity analysis[7].

For India and Indonesia, GBD produced subnational estimates of neonatal, infant, and child mortality at the first administrative level. For these countries, we calibrated our estimates to these subnational estimates. Overall, the median scaling factor across subnational units in these countries were: for neonates 1.00, for infants 1.06, and for under-5s 1.03, and the inter-quartile ranges were 0.91–1.13, 0.95–1.16, and 0.93–1.13 for the neonates, infants, and under-5s, respectively. The relationship between our aggregated predictions (i.e. pre-calibration predictions) and those from the subnational areas in these countries from GBD 2017 are compared in Supplementary Figures 5.6-5.8.

Supplementary Figure 5.3: Pre- and post-calibration comparisons, national level GBD, under-5

Comparison of under-5 mortality probability estimates for 2000, 2005, 2010, and 2017 in GBD-reported national geographies derived by population weighting 5 x 5-km grid cell estimates before (x-axis) and after (y-axis) calibration to GBD 2017 by year.

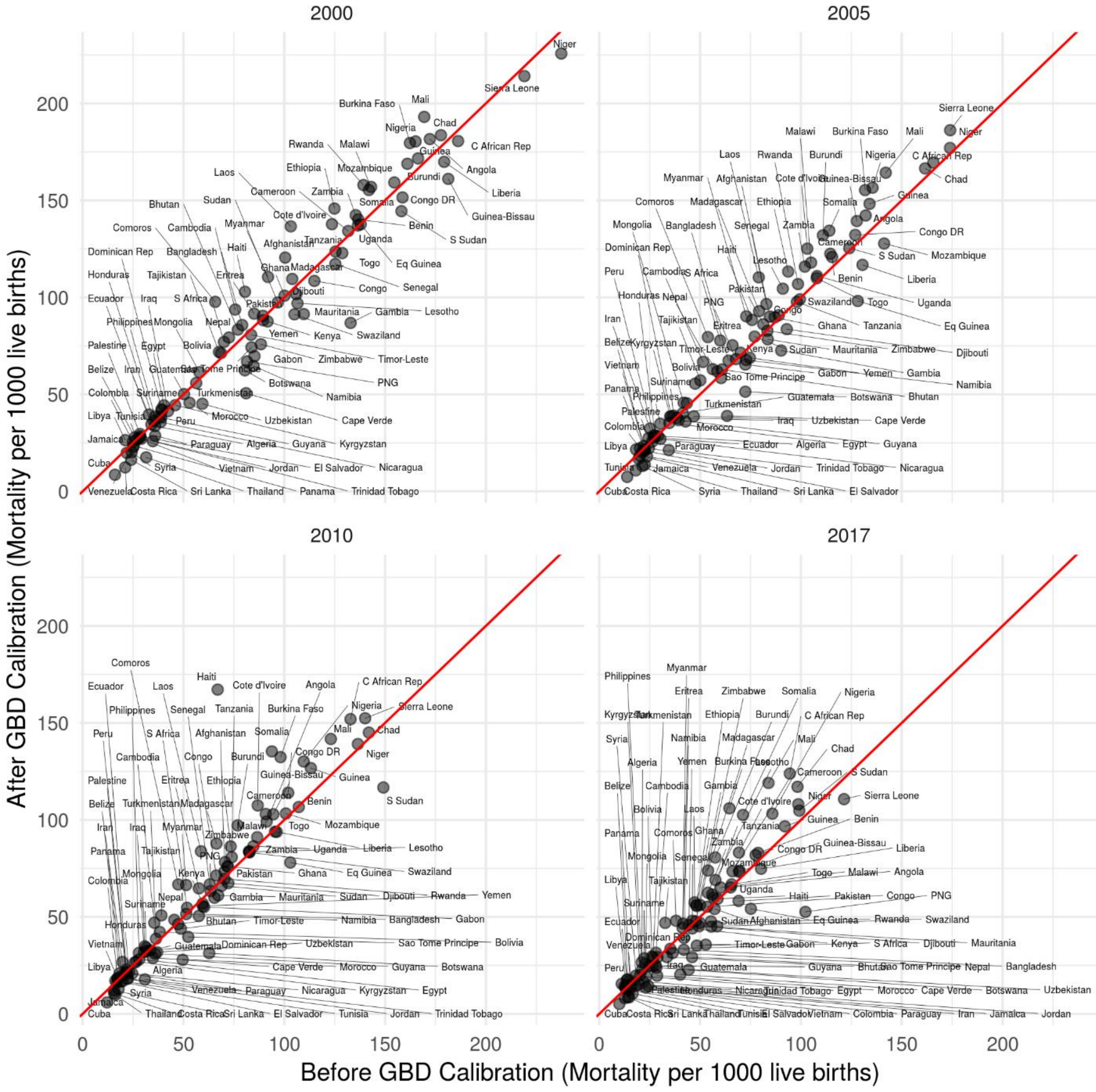

Supplementary Figure 5.4: Pre- and post-calibration comparisons, national-level GBD, infant

Comparison of infant mortality probability estimates for 2000, 2005, 2010, and 2017 in GBD-reported national geographies derived by population weighting 5 x 5-km grid cell estimates before (x-axis) and after (y-axis) calibration to GBD 2017 by year.

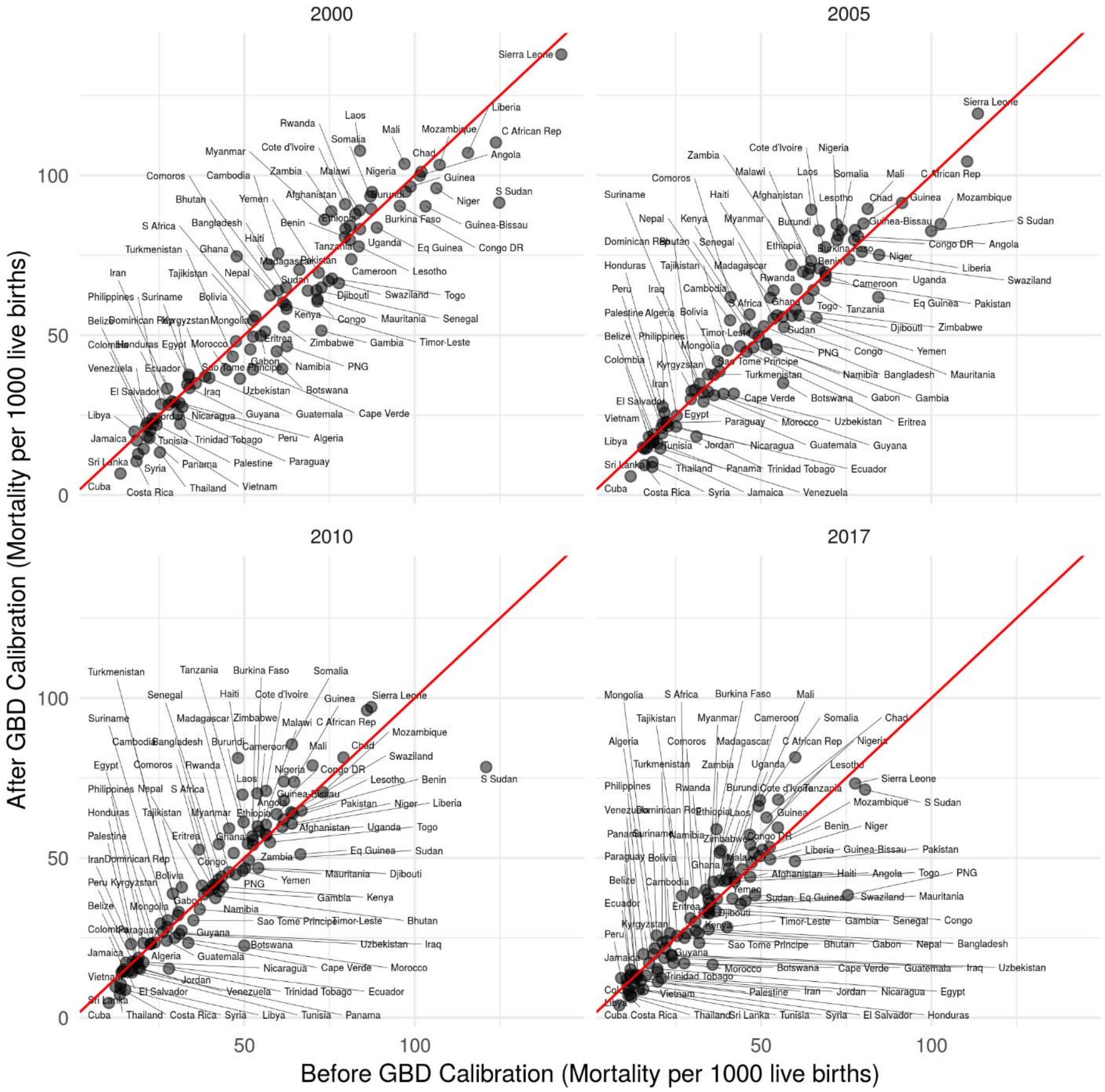

Supplementary Figure 5.5: Pre- and post-calibration comparisons, national level GBD, neonatal

Comparison of neonatal mortality probability estimates for 2000, 2005, 2010, and 2017in GBD reported national geographies derived by population weighting 5 x 5-km grid cell estimates before (x-axis) and after (y-axis) calibration to GBD 2017 by year.

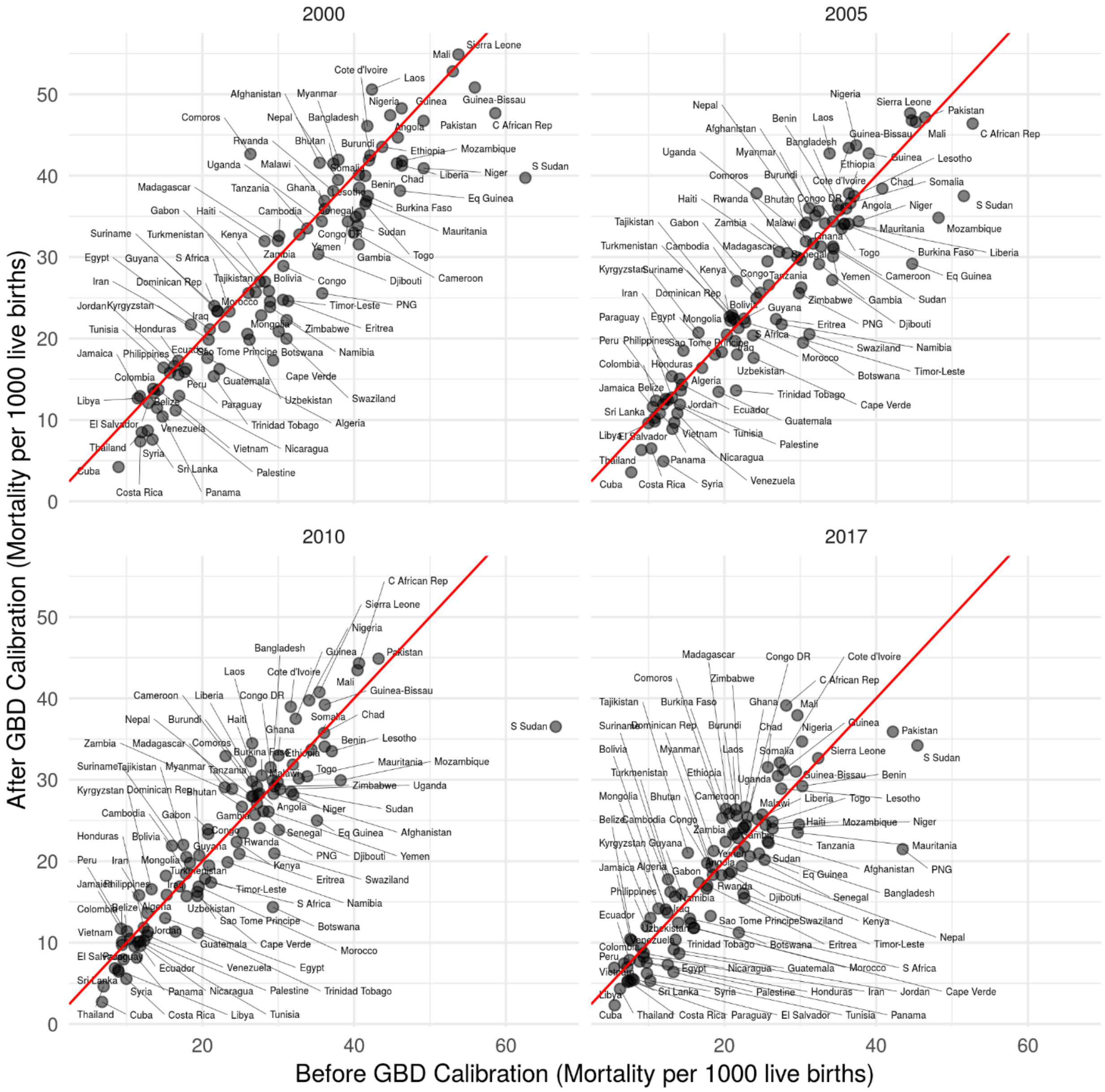

Supplementary Figure 5.6: Pre- and post-calibration comparisons, subnational level GBD, under-5

Comparison of under-5 mortality probability estimates for 2000, 2005, 2010, and 2017 in GBD reported subnational geographies derived by population weighting 5 x 5-km grid cell estimates before (x-axis) and after (y-axis) calibration to GBD 2017 by year.

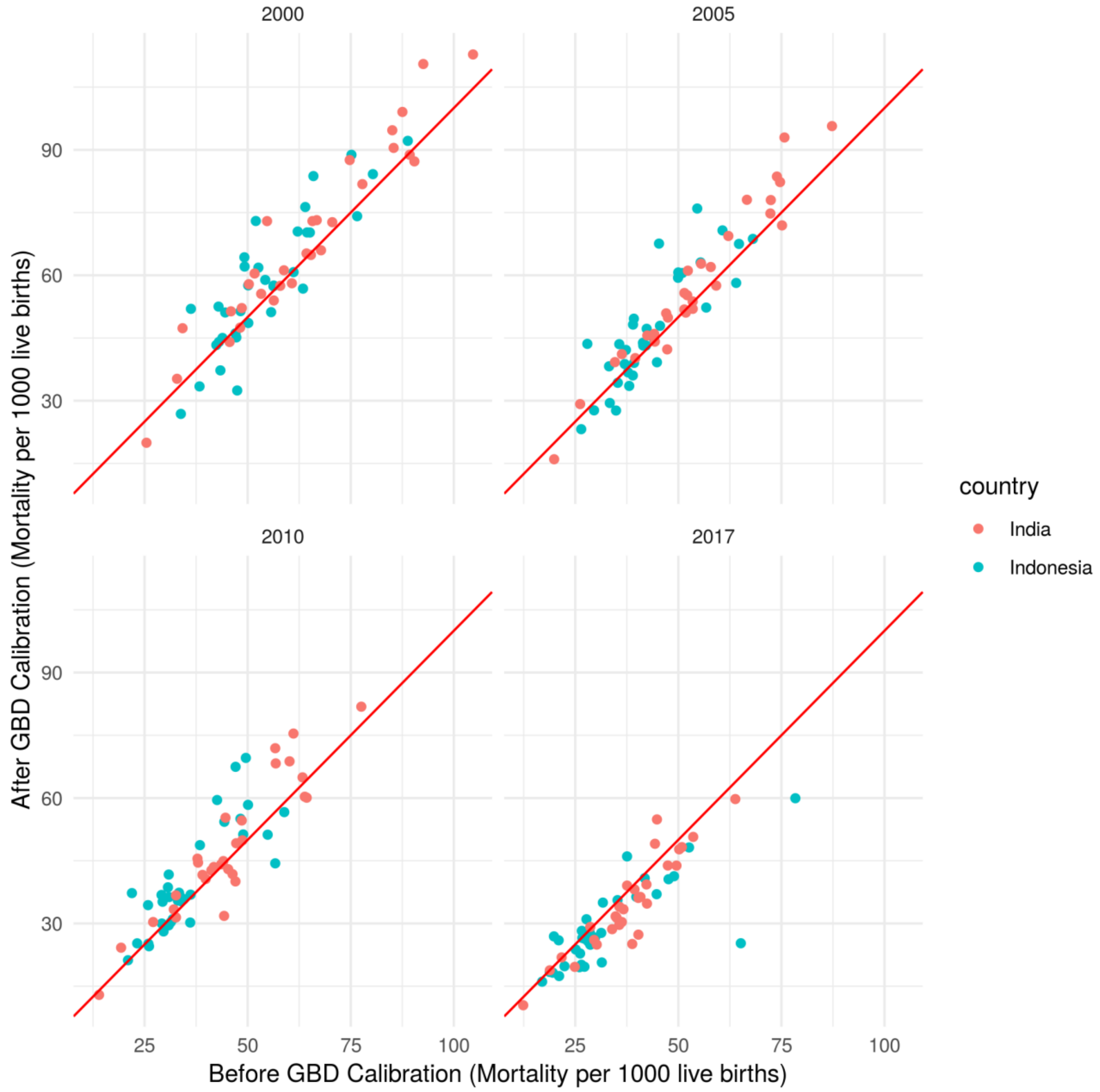

Supplementary Figure 5.7: Pre- and post-calibration comparisons, subnational-level GBD, infant

Comparison of infant mortality probability estimates for 2000, 2005, 2010, and 2017 in GBD reported subnational geographies derived by population weighting 5 x 5-km grid cell estimates before (x-axis) and after (y-axis) calibration to GBD 2017 by year.

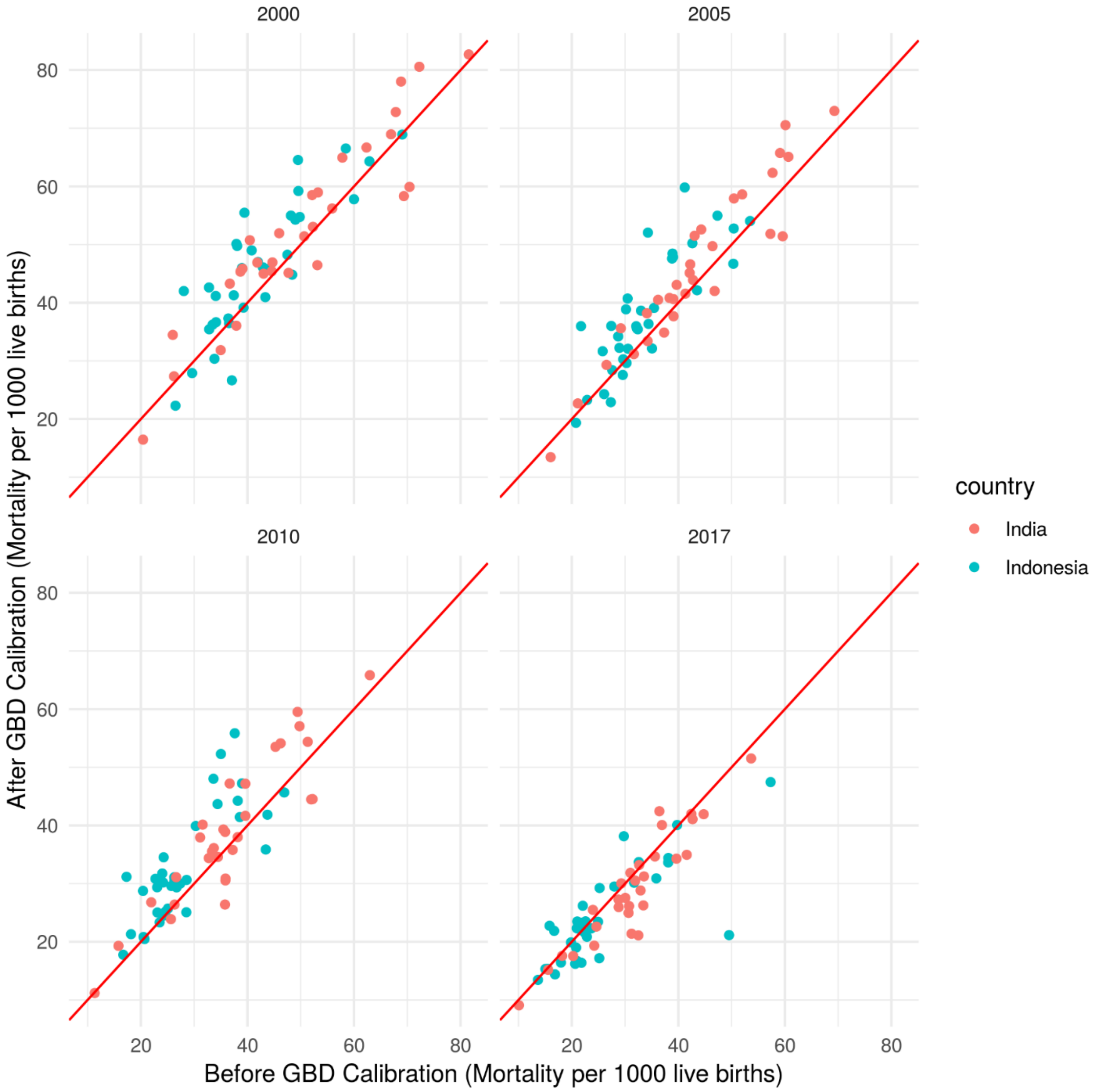

Supplementary Figure 5.8: Pre- and post-calibration comparisons, subnational-level GBD, neonatal

Comparison of neonatal mortality probability estimates for 2000, 2005, 2010, and 2017 in GBD reported subnational geographies derived by population weighting 5 x 5-km grid cell estimates before (x-axis) and after (y-axis) calibration to GBD 2017 by year.

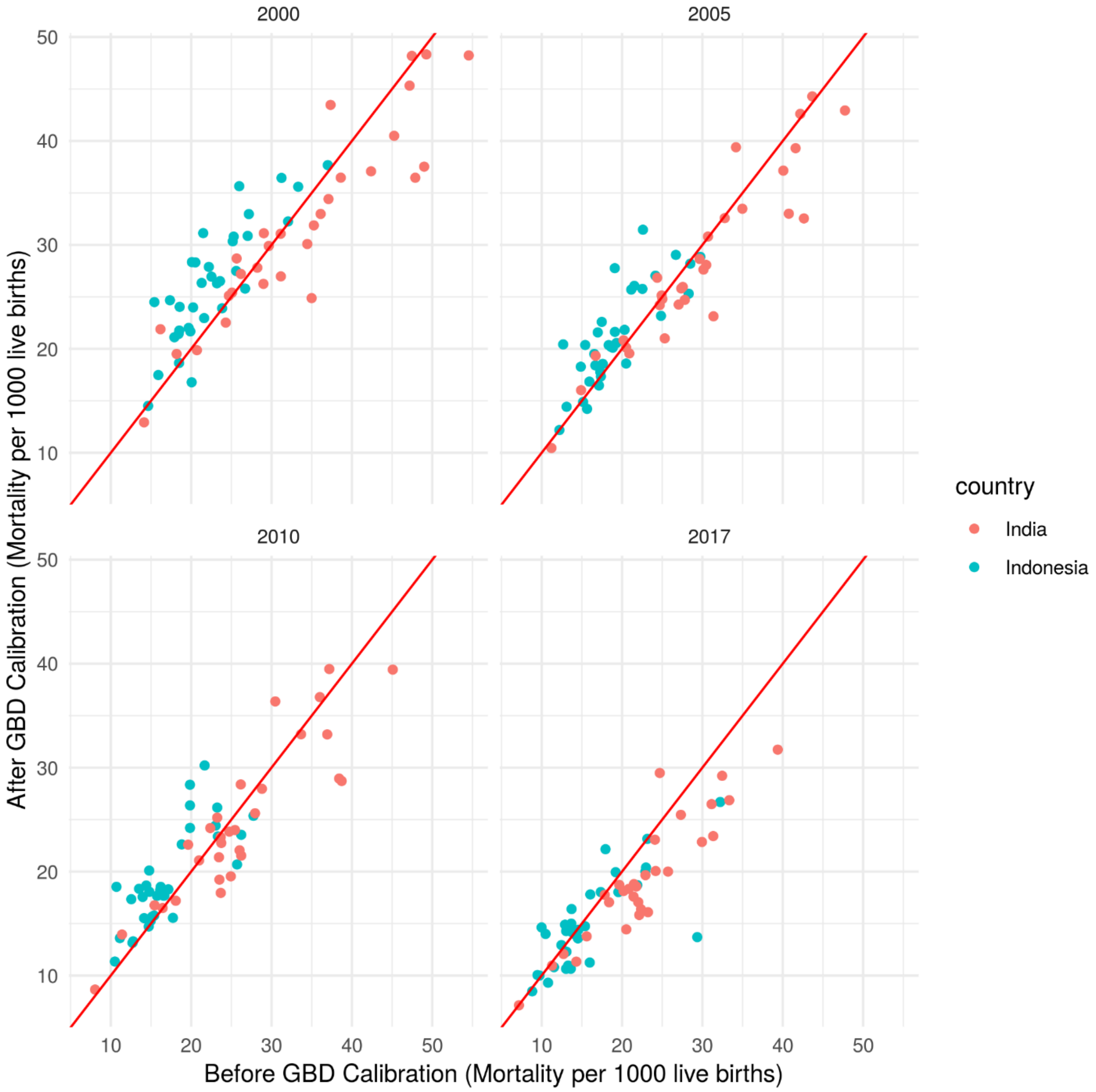

5.3 Validation

We utilized five-fold cross-validation to assess and compare model performance with respect to estimating local trends of age-specific mortality. Each fold was created by combining complete surveys into subsets of ~20% of data sources from the input data. Holding out entire surveys at a time served as a comparable approximation to the type of missingness in our data, essentially helping us to answer the question: how would our model estimates of mortality probabilities compare to empirical estimates of mortality probability from a new survey which did not inform the model?

We ran the geostatistical model described above five times for each modeling region, with each run holding out one fold. The withheld surveys in each holdout run thus gave us an empirical basis for assessing model performance. After we fit the model five times in a region, we compiled the held-out data for each fold and extracted the out of sample (OOS) estimated mortality probability at each data point. High sampling variability due to small sample sizes at the survey cluster level make data at that level generally insufficient for comparison. To address this, we aggregated each estimate from each survey to the country level as well as the first and second administrative level for each year of available data. At each level of aggregation, we took the weighted mean of empirical and estimated mortality probabilities based on sample sizes of data points. Estimates were aggregated at the draw level, and then means were taken at the aggregated level across draws. Using these aggregated data and aggregated estimate pairs, we calculated the difference between OOS empirical data estimates and modeled estimates (referred to as "errors") and we report the following summary metrics: mean error (ME), which serves as a measure of bias; the mean of absolute errors (MAE), which serves as a measure the total variation in the errors; the correlation. In addition, we calculated 95% coverage. For this, we constructed 95% prediction intervals based on the draws from the modeled estimates. For each draw, we took a binomial sample, with sample size based on that of the empirical data. Binomial samples – representing predicted numbers of deaths – were taken at the data-draw level for each observed age, period, location, data point from each survey. Simulated deaths were aggregated to the second administrative, first administrative, and country levels for each year. Empirical observations of death counts were also aggregated at these levels for comparison. We calculated coverage as the percentage of empirical observations at each of these levels which fell between the 2.5% and 97.5% simulated prediction interval.

In addition to OOS validation, we also assessed in-sample (IS) predictive validity. For IS predictive validity, we compared modelled estimates to the training data that informed the model itself. *A priori,* we expect IS to show better results than OOS if the model behaves as expected.

5.3.1 Validation results

In this section we present results for IS and OOS validation.

Supplementary Table 5.1 shows summary predictive validity metrics for under-5 mortality probability estimates across levels of aggregation and for IS and OOS. In general, IS performed slightly better than

OOS, as expected. Supplementary Table 5.2 shows the same high-level summary for all three age groups, neonatal, infant, and under-5, at the second administrative level of aggregation.

Supplementary Figures 5.9 through 5.14 show the data behind these high-level summaries for under-5 mortality probability, showing comparisons of predictions versus empirical estimates for IS and OOS and for the three levels of spatial aggregation. Each point represents an estimate corresponding to a survey-year-aggregate estimate. It is clear from these scatter plots that there is considerable data variation due to small sample sizes. For second administrative level, the average number of births informing a validation point was 18, at the first administrative the average sample size was 89, and at the country level it was 1,216. As such, it is more difficult to make direct comparisons at lower levels of aggregation, since empirical estimates are noisy. This is evident by the vertical bands of values in the scatter plots which arise from ratios with smaller sample sizes. Empirical estimates made from data points with a larger sample size tend to show more agreement.

Supplementary Table 5.1: High-level summary of validation metrics by administrative level

High-level summary of validation metrics for under-5 mortality probability across all years and regions, for three different levels of aggregation: second administrative level (Admin 2), first administrative level (Admin 1), and country.

Aggregation Level	IS/OOS	Mean Prediction	Mean Observed	Mean Error	Mean Absolute Error	Correlation	95% coverage
Admin 2	IS	0.0631	0.0616	-0.00148	0.0192	0.791	97.1%
Admin 2	OOS	0.0640	0.0616	-0.00240	0.0225	0.725	96.4%
Admin 1	IS	0.0633	0.0620	-0.00129	0.0127	0.905	93.1%
Admin 1	OOS	0.0641	0.0620	-0.00218	0.0161	0.837	90.6%

Supplementary Table 5.2: High-level summary of validation metrics by age bin

High-level summary of validation metrics for mortality probability for each reported age bin across all years and regions, for the second administrative level aggregation.

Age Bin	IS/OOS	Mean Prediction	Mean Observed	Mean Error	Mean Absolute Error	Correlation	95% Cov.
Neonatal	IS	0.0261	0.0255	-0.000583	0.0110	0.564	96.9%
Infant	IS	0.045	0.044	-0.00112	0.0158	0.706	97.3%
Under-5	IS	0.0631	0.0616	-0.00148	0.0192	0.791	97.1%
Neonatal	OOS	0.0264	0.0255	-0.000866	0.0125	0.414	96.6%
Infant	OOS	0.046	0.044	-0.0018	0.0183	0.604	96.8%
Under-5	OOS	0.0640	0.0616	-0.00240	0.0225	0.725	96.4%

Supplementary Figure 5.9: In-sample predictions versus aggregated data at the country level

Each point represents an estimate for under-5 mortality probability for a source-year.

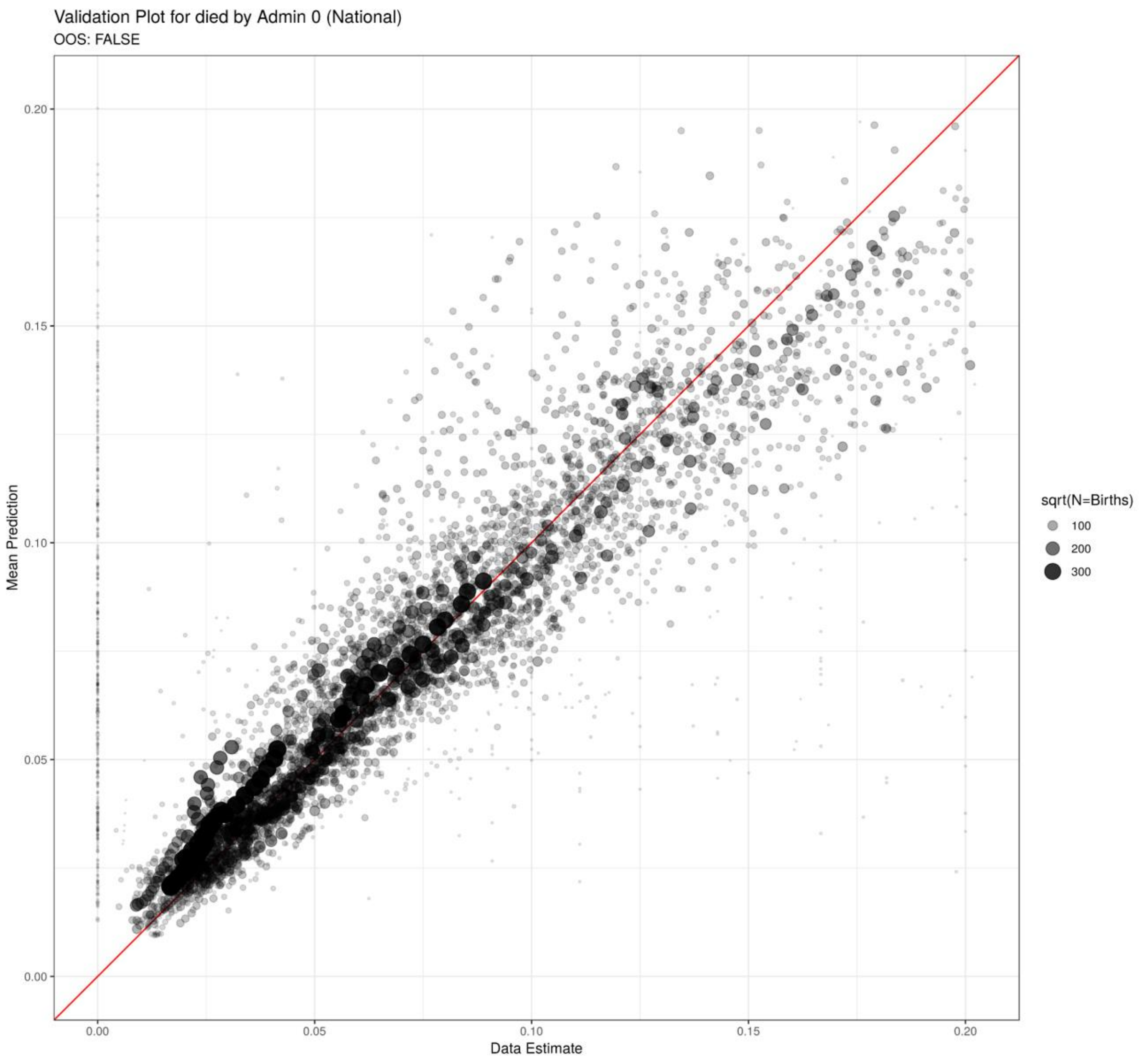

Validation Plot for died by Admin 0 (National)
OOS: FALSE
Mean Prediction
0.20
0.15
0.10
0.05
0.00
Data Estimate
0.00
0.05
0.10
0.15
0.20
sqrt(N=Births)
100
200
300

747

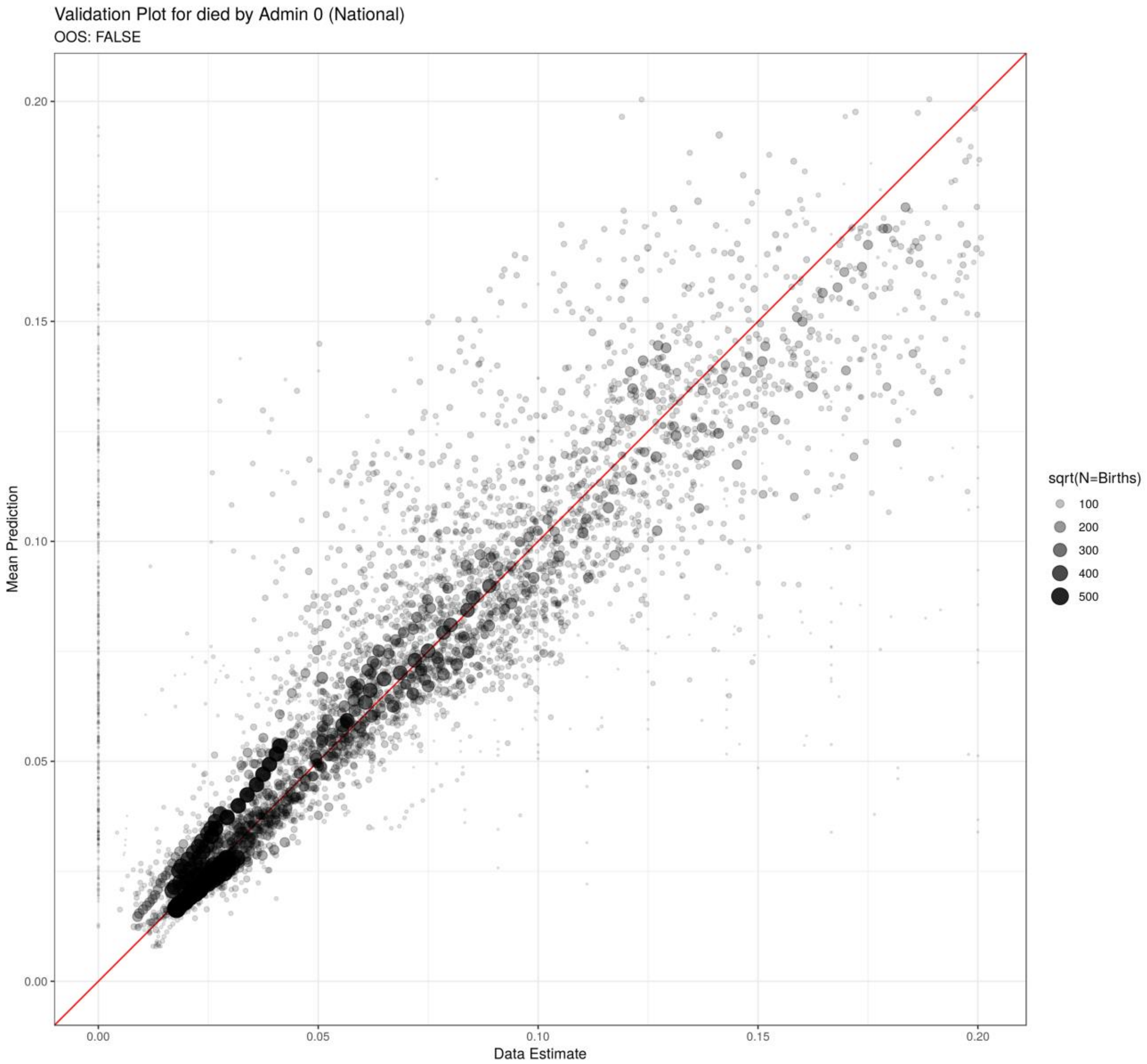
Validation Plot for died by Admin 0 (National)
OOS: FALSE
Mean Prediction
Data Estimate
0.00
0.05
0.10
0.15
0.20
sqrt(N=Births)
100
200
300
400
500

Supplementary Figure 5.10: Out-of-sample predictions vs. aggregated data at the country level

Each point represents an estimate for under-5 mortality probability for a source-year.

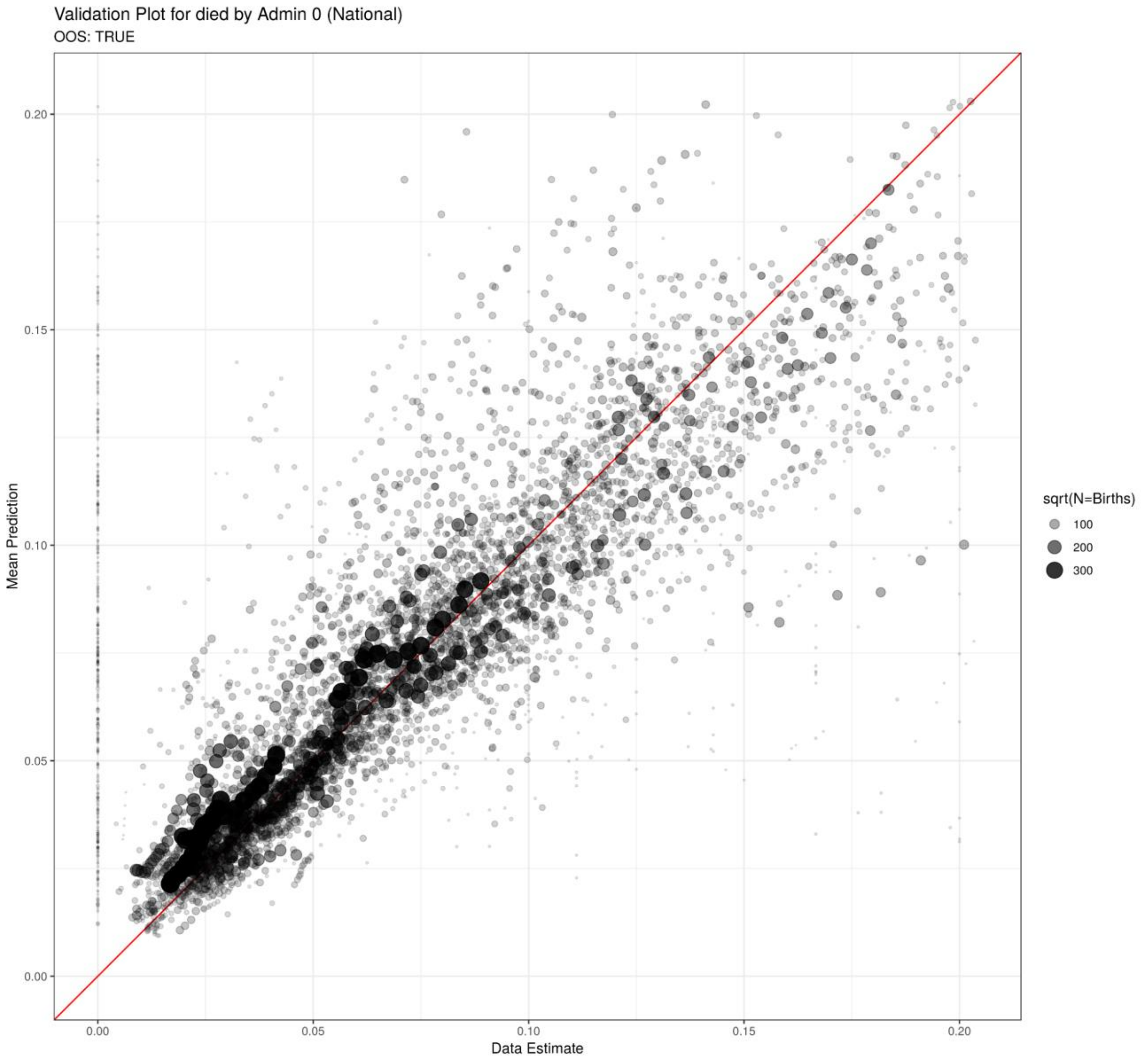
Validation Plot for died by Admin 0 (National)
OOS: TRUE
Mean Prediction
Data Estimate
0.00
0.05
0.10
0.15
0.20
sqrt(N=Births)
100
200
300

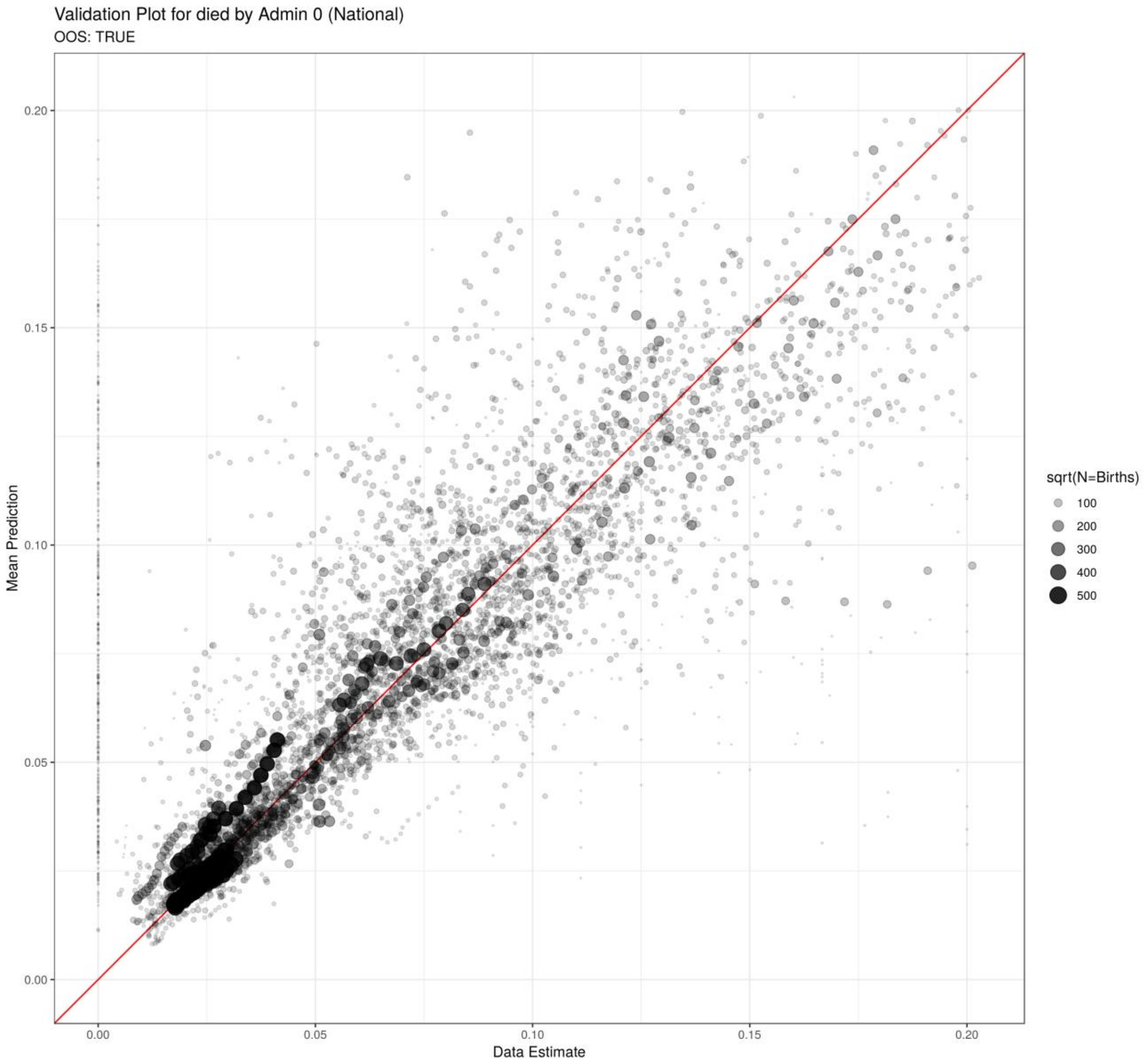
Validation Plot for died by Admin 0 (National)
OOS: TRUE
Mean Prediction
0.20
0.15
0.10
0.05
0.00
Data Estimate
0.00
0.05
0.10
0.15
0.20
sqrt(N=Births)
100
200
300
400
500

Supplementary Figure 5.11: In-sample predictions vs. aggregated data at the first administrative level

Each point represents an estimate for under-5 mortality probability for a source-year.

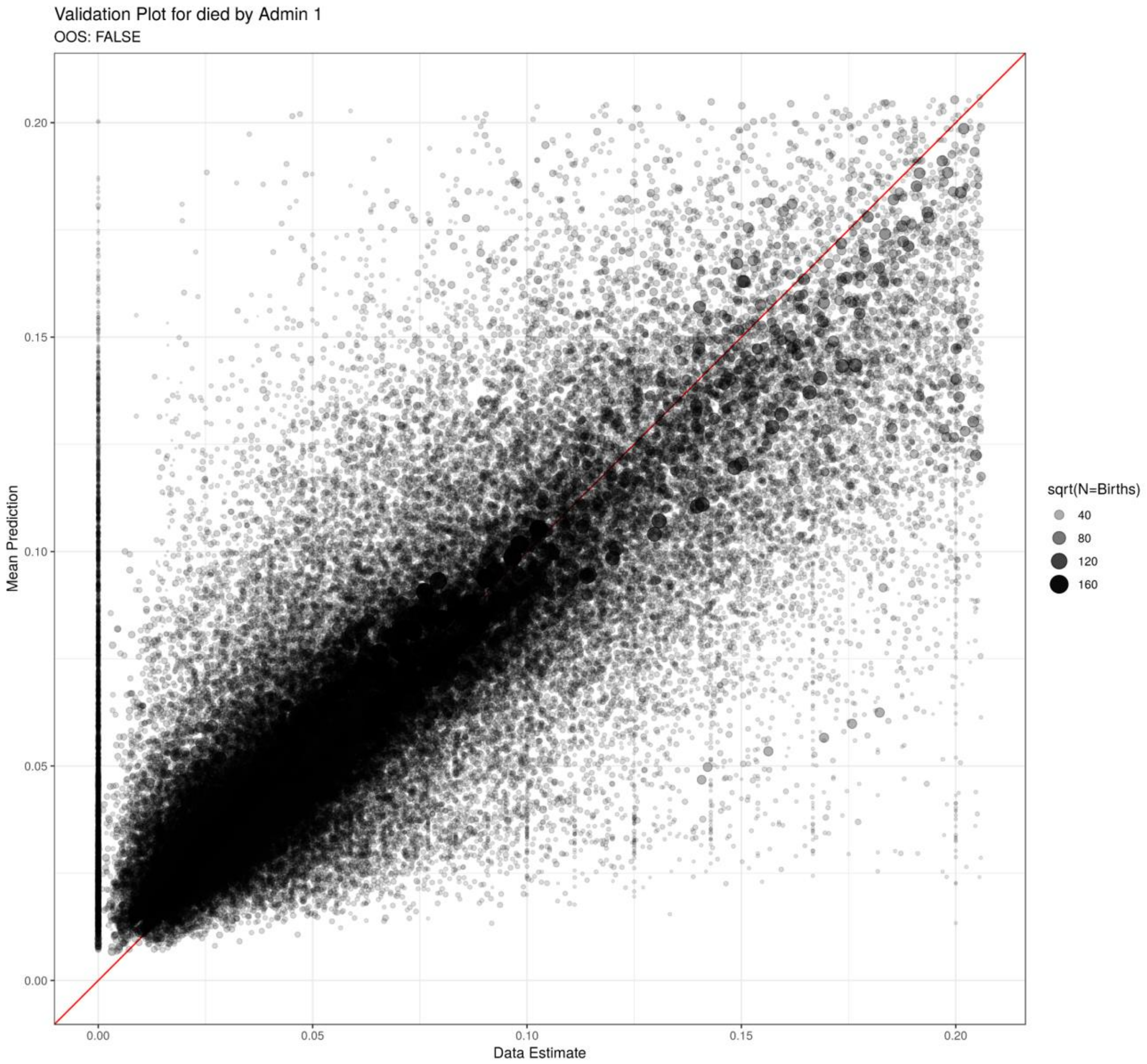

Validation Plot for died by Admin 1
OOS: FALSE
Mean Prediction
0.00
0.05
0.10
0.15
0.20
Data Estimate
0.00
0.05
0.10
0.15
0.20
sqrt(N=Births)
40
80
120
160

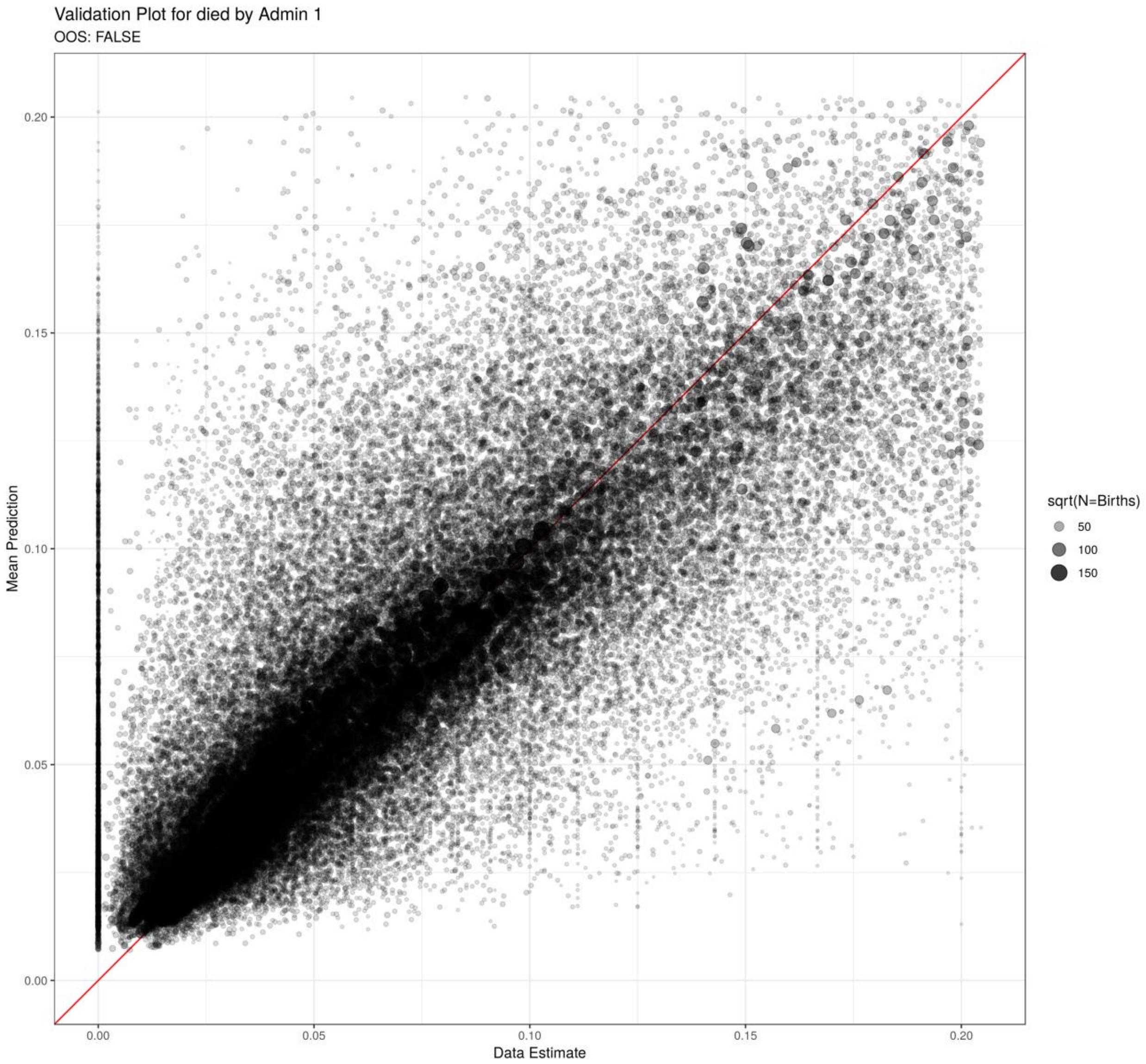
Validation Plot for died by Admin 1
OOS: FALSE
Mean Prediction
0.00
0.05
0.10
0.15
0.20
Data Estimate
0.00
0.05
0.10
0.15
0.20
sqrt(N=Births)
50
100
150

Supplementary Figure 5.12: Out-of-sample predictions vs. aggregated data at the first administrative level

Each point represents an estimate for under-5 mortality probability for a source-year.

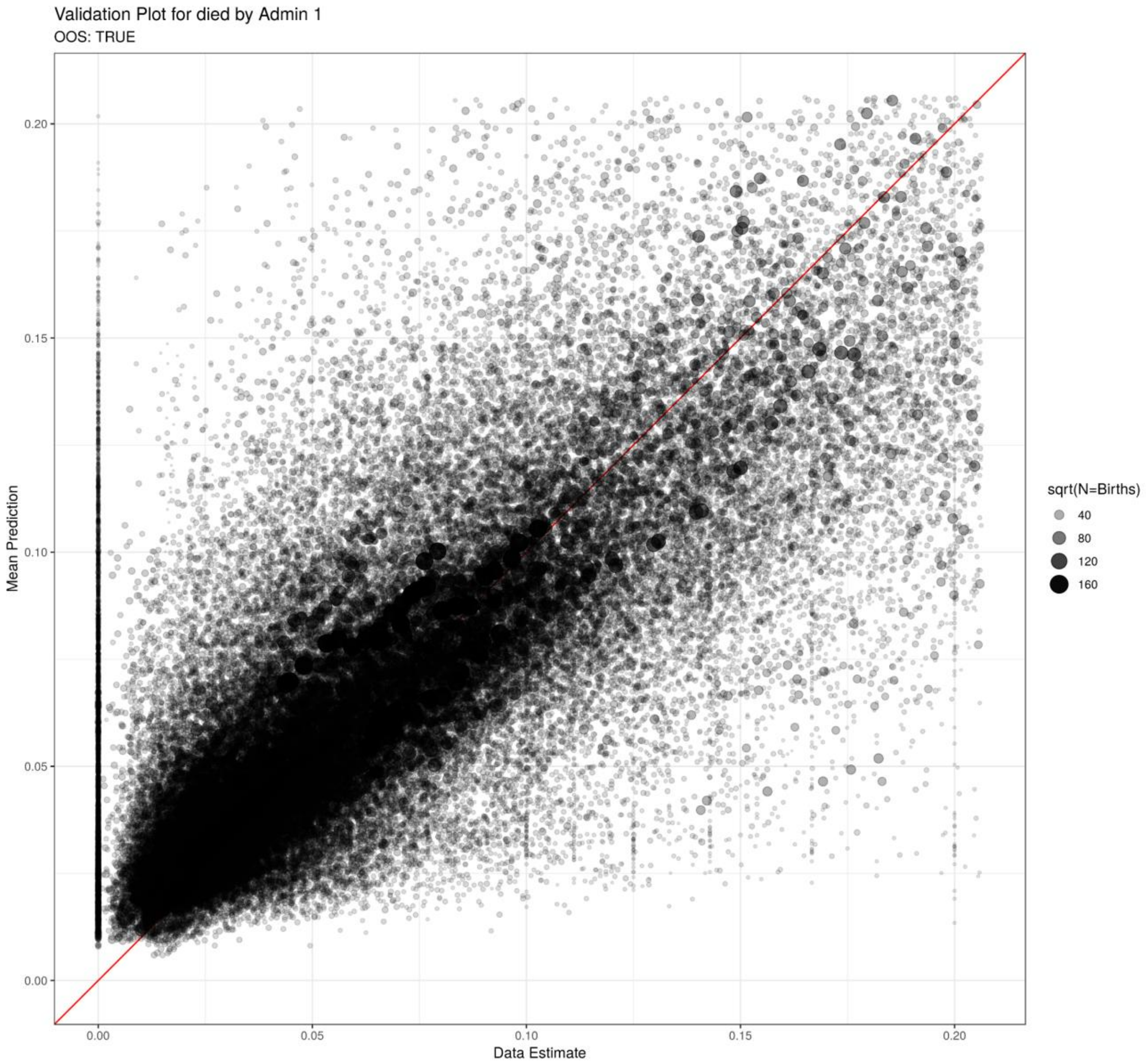
Validation Plot for died by Admin 1
OOS: TRUE
Mean Prediction
0.00
0.05
0.10
0.15
0.20
Data Estimate
sqrt(N=Births)
40
80
120
160

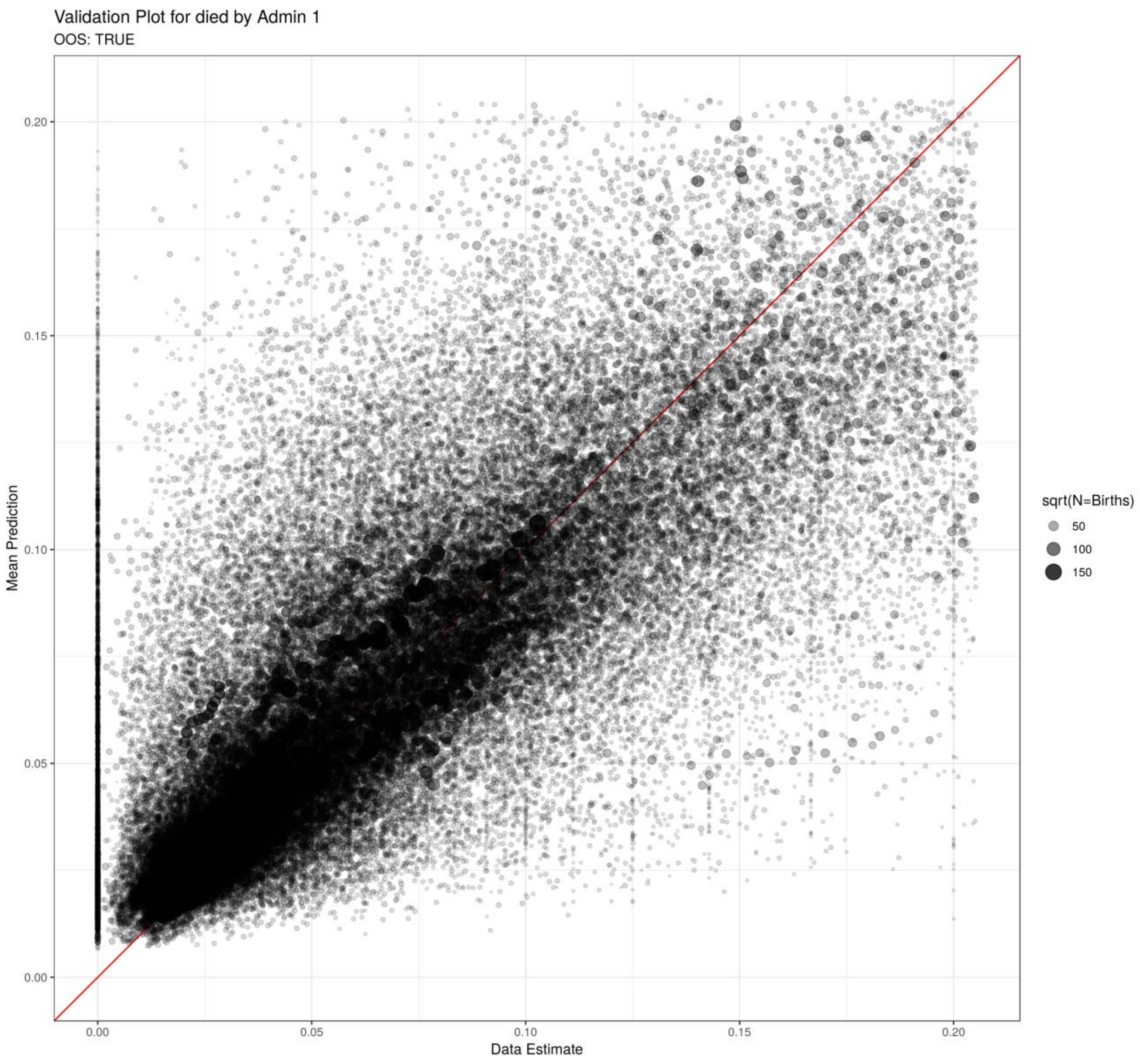
Validation Plot for died by Admin 1
OOS: TRUE
Mean Prediction
0.00
0.05
0.10
0.15
0.20
Data Estimate
0.00
0.05
0.10
0.15
0.20
sqrt(N=Births)
50
100
150

Supplementary Figure 5.13: In-sample predictions vs. aggregated data at the second administrative level

Each point represents an estimate for under-5 mortality probability for a source-year.

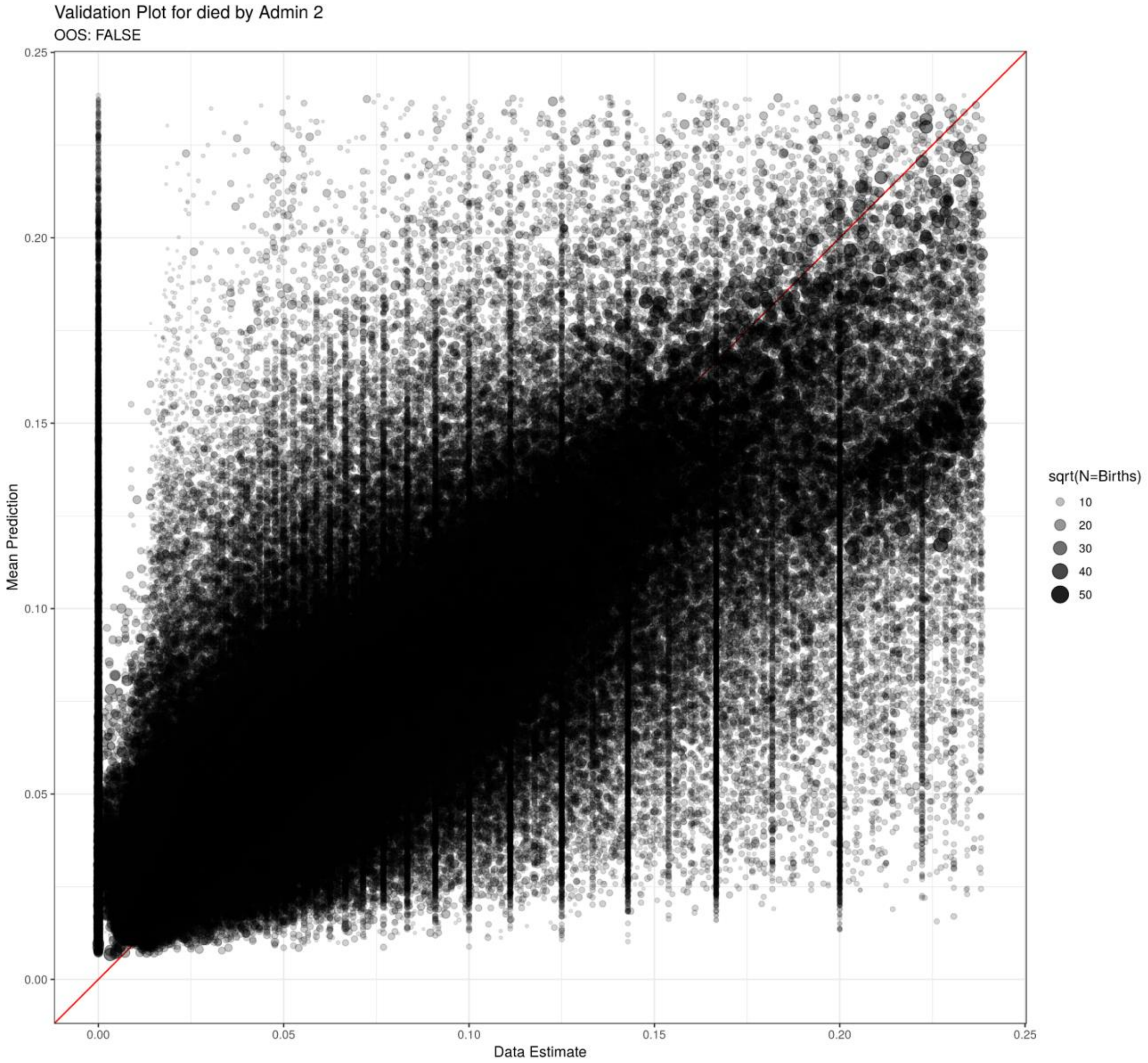
Validation Plot for died by Admin 2
OOS: FALSE
Mean Prediction
Data Estimate
0.00
0.05
0.10
0.15
0.20
0.25
sqrt(N=Births)
10
20
30
40
50

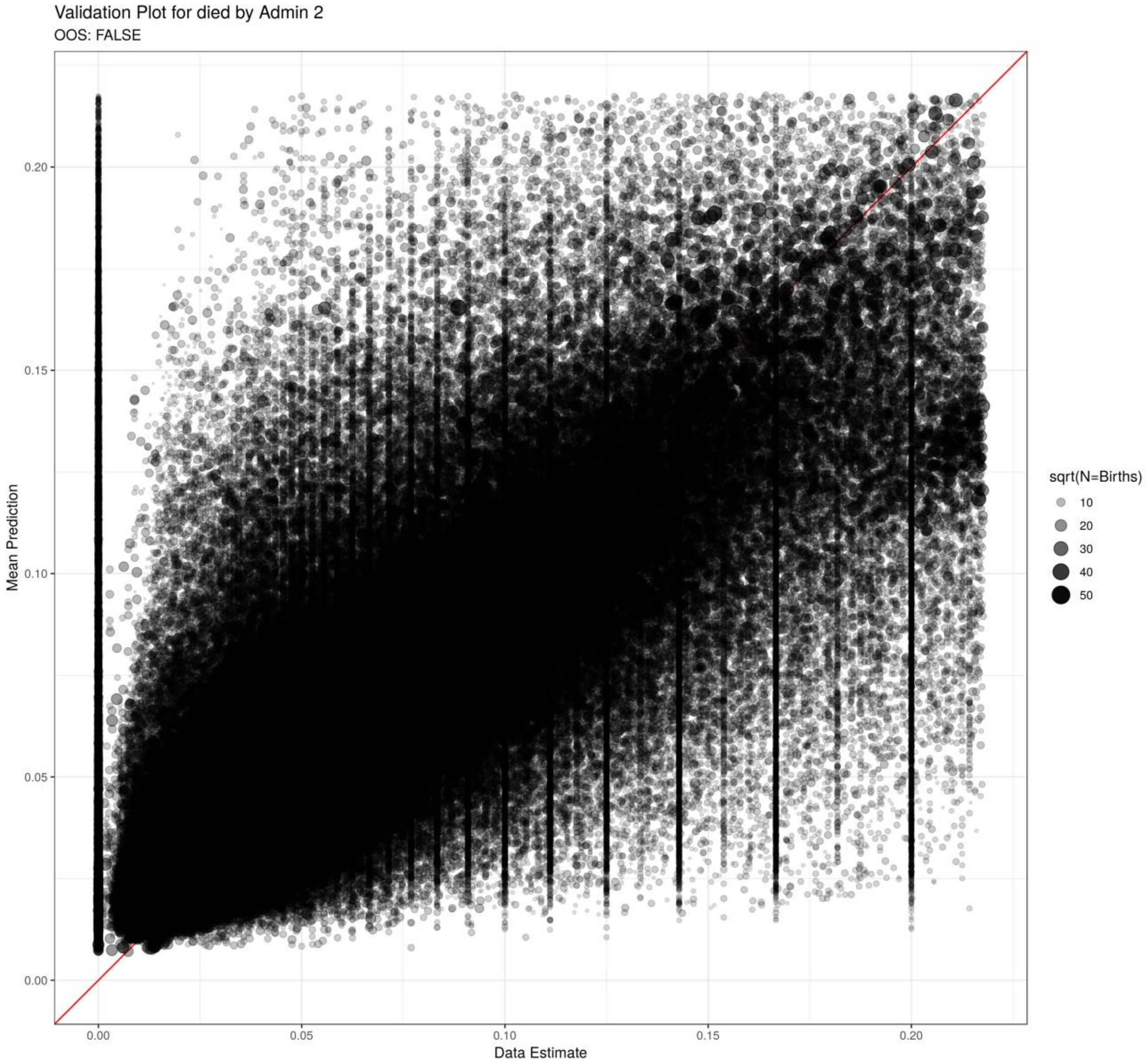
Validation Plot for died by Admin 2
OOS: FALSE
Mean Prediction
Data Estimate
0.00
0.05
0.10
0.15
0.20
sqrt(N=Births)
10
20
30
40
50

Supplementary Figure 5.14: Out-of-sample predictions vs. aggregated data at the second administrative level

Each point represents an estimate for under-5 mortality probability for a source-year.

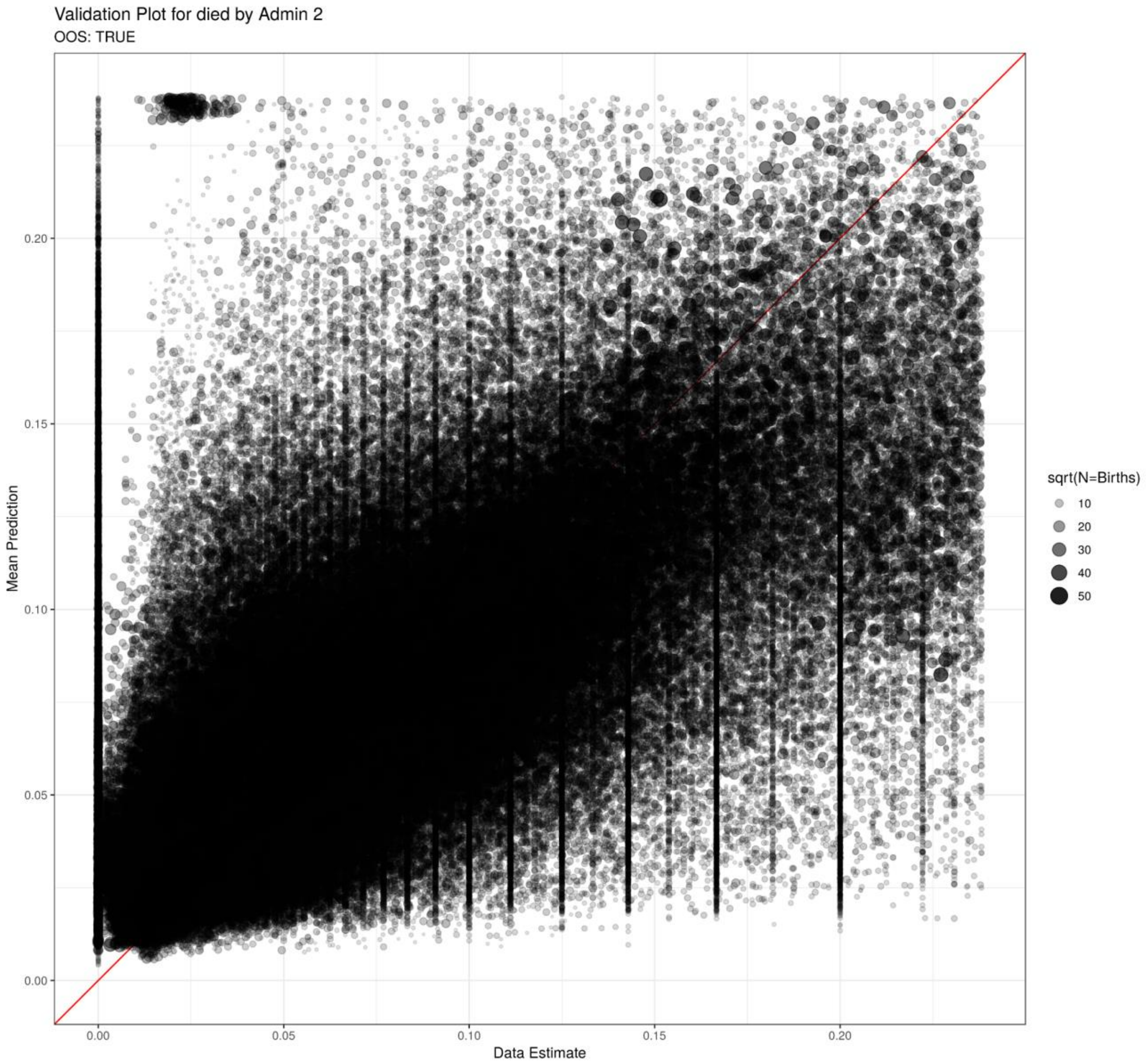
Validation Plot for died by Admin 2
OOS: TRUE
Mean Prediction
0.00
0.05
0.10
0.15
0.20
Data Estimate
0.00
0.05
0.10
0.15
0.20
sqrt(N=Births)
10
20
30
40
50

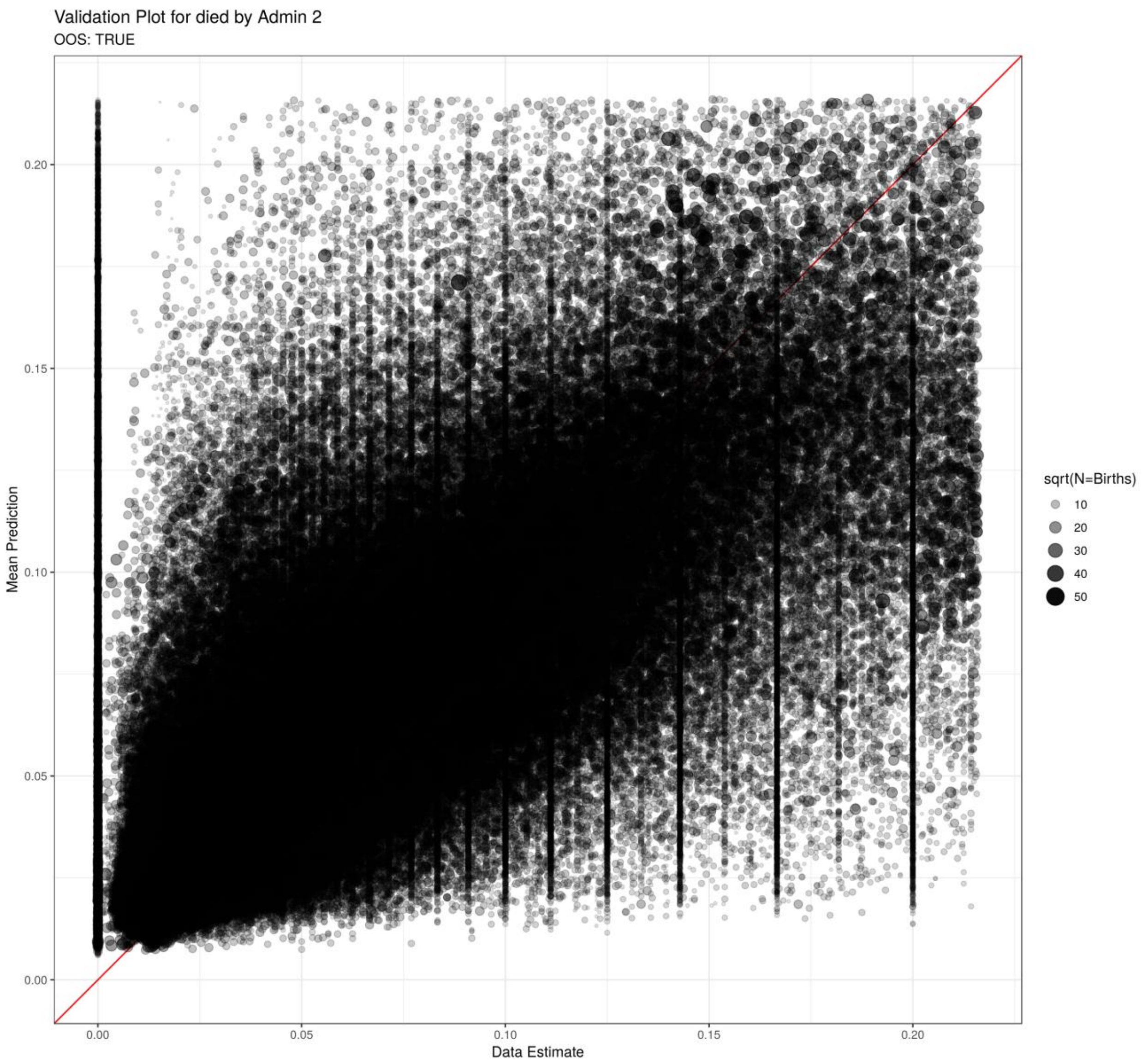
Validation Plot for died by Admin 2
OOS: TRUE
Mean Prediction
Data Estimate
0.00
0.05
0.10
0.15
0.20
sqrt(N=Births)
10
20
30
40
50

5.4 Other diagnostics

5.4.1 Comparison with previous analysis

Prior to the current analysis, we estimated mortality probabilities for neonates and under-5s in 46 African countries for four time periods: 1998–2002 (which we refer to as 2000), 2003–2007 (2005), 2008–2012 (2010), and 2012–2017 (2015)[1]. In this section we describe differences between those estimates and the ones we present for the current analysis.

Reasons for differences include changes in national-level estimates from GBD leading to differences in the calibration step, the addition of 44 data sources in these countries (see a list in Supplementary Table 5.3), and changes to the analytical process. Major changes to the analytical process included updates to the way SBH data were prepped, using seven age bins rather than four, and changes to the statistical model. Major changes to the statistical model included a smaller set of covariates fit linearly rather than using a generalized stacking method, jointly fitting the model across age bins and accounting for correlation across age bins in time and space, including data-source-specific random effects rather than using bias correction estimates from the GBD study, making estimates at an annual temporal scale rather than a five-year temporal scale, and using country random effects.

We compared our estimates of neonatal and under-5 mortality probabilities in these 46 countries at the second administrative level. In order to make them temporally comparable, we combined our estimates into 5-year bins at the draw level. We compared absolute and relative mean differences, and looked at the significance of the difference (whether or not we had non-overlapping uncertainty intervals at the 95% level at each location). Since we did not make estimates for 1998 or 1999 in this report, we only compared estimates for the 2005, 2010, and 2015 periods. We show comparisons in Supplementary Figures 5.15 – 5.19.

Across second administrative units, estimates for under-5 mortality probability had correlation coefficients 0.97, 0.96, and 0.94 for the 2005, 2010, and 2015 time periods, respectively. The correlation coefficients for neonatal mortality probability were 0.91, 0.87, and 0.86 for these time periods (see Supplementary Figures 5.15 – 5.16). The average relative difference for these time periods was 1.01, 1.01, and 1.03 for under-5 mortality probability and 1.01, 1.02, and 1.03 for neonatal mortality probability in these same time periods (see Supplementary Figure 5.17). The average absolute difference for these time periods was 0.14, -0.21, and 0.48 deaths per 1,000 live births for under-5 mortality and 0.06, 0.54, and 0.69 for neonatal mortality in these same time periods (see Supplementary Figure 5.18). Of 5,949 total administrative units, 43 (0.7%), 117 (2.0%), and 103 (1.7%) areas had significantly different results for under-5 mortality probability estimates in these years, as defined by non-overlapping uncertainty intervals. Likewise, 88 (1.5%), 98 (1.6%), and 43 (0.7%) areas had significantly different results for neonatal mortality probability estimates (see Supplementary Figure 5.19).

Supplementary Figure 5.15: Comparison between mean second administrative level estimates of under-5 mortality probability between current and previous analysis

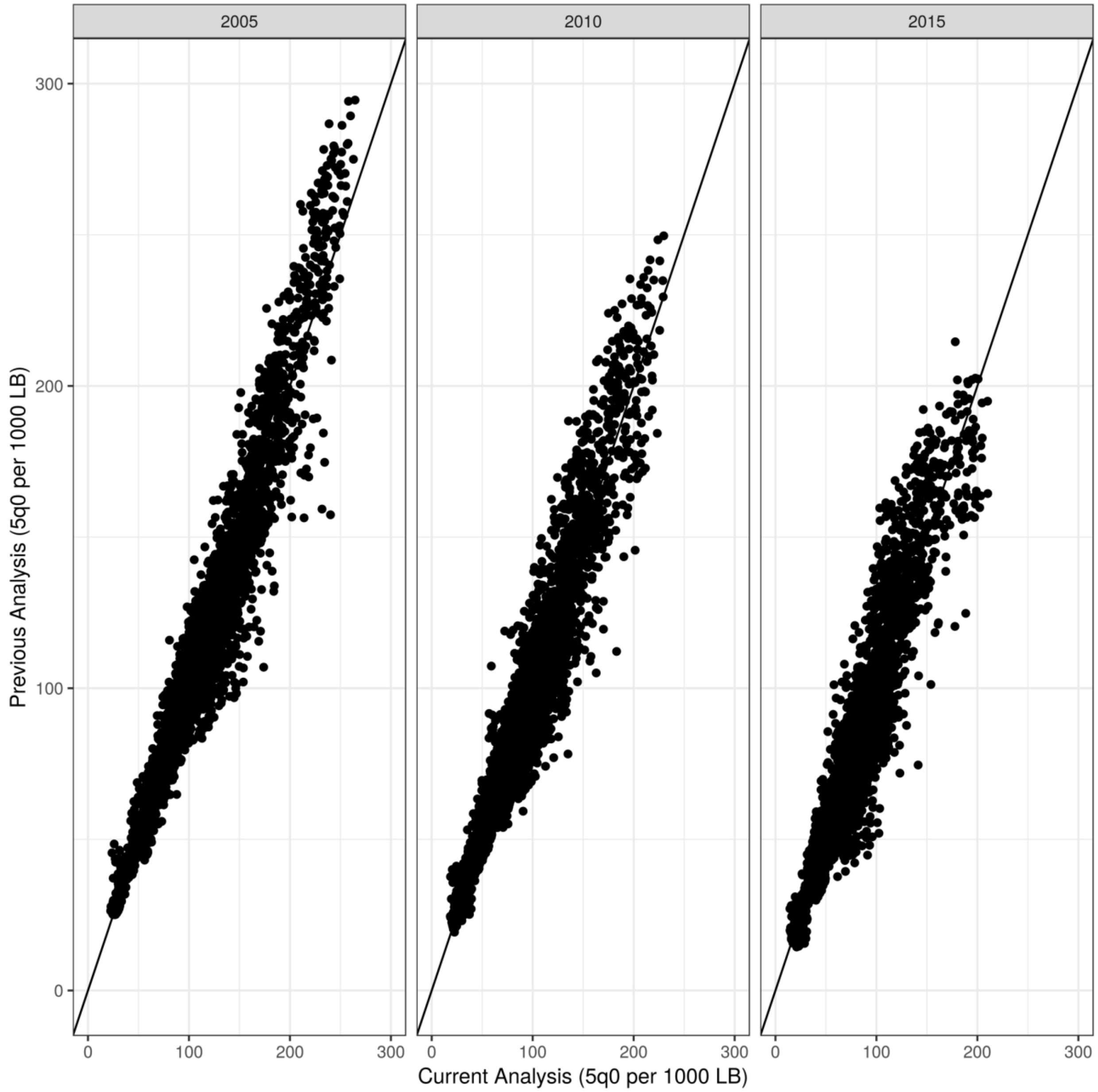

Supplementary Figure 5.16: Comparison between mean second administrative level estimates of neonatal mortality probability between current and previous analysis

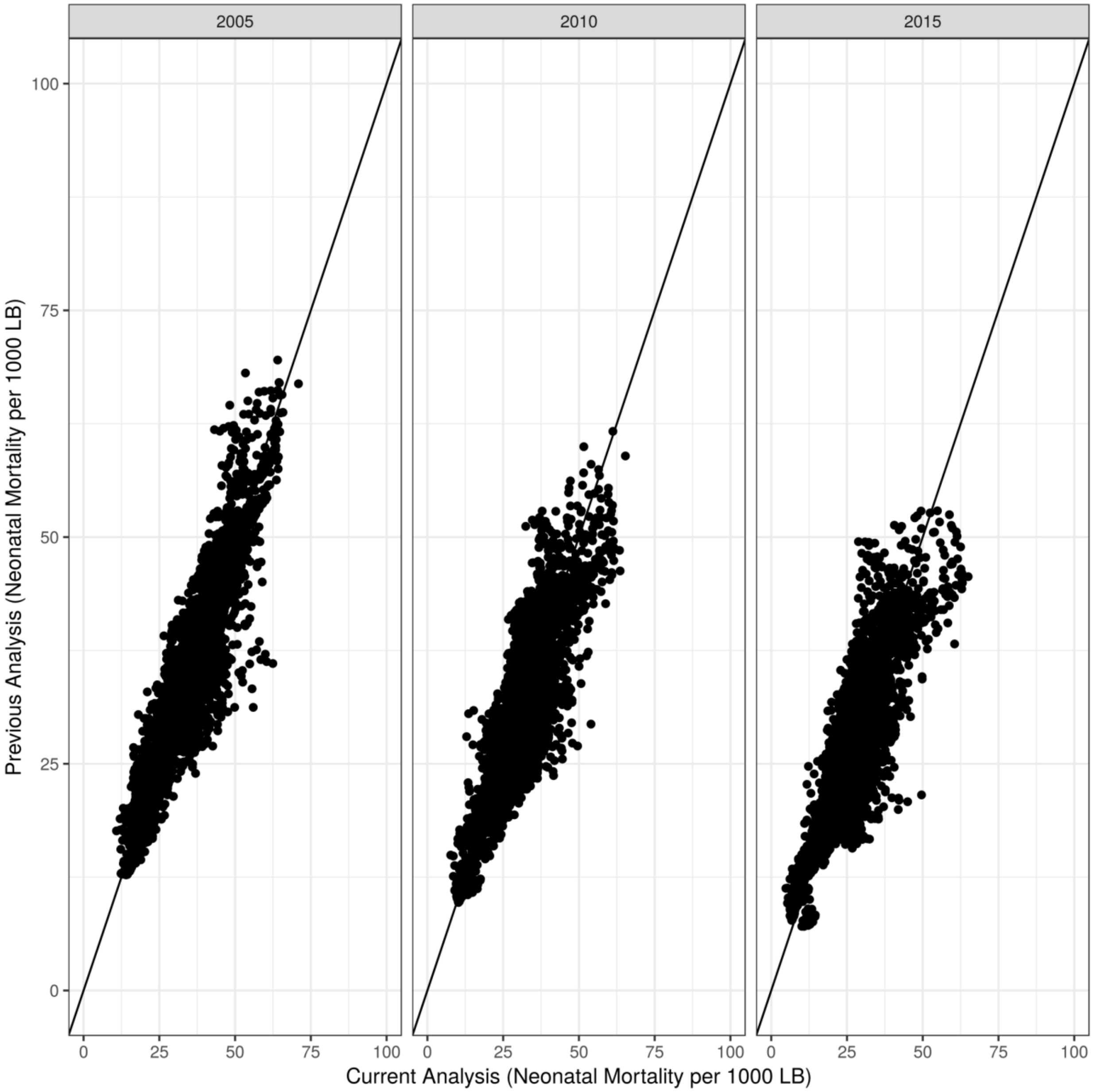

Supplementary Figure 5.17: Relative difference between current and previous estimates of under-5 mortality probability, 2015

Relative difference between current and previous estimates of under-5 mortality probability for the 2015 period (defined as 2013–2017) across all second administration units in the 46 countries analyzed in the previous analysis. The countries in gray were not analyzed in the previous analysis.

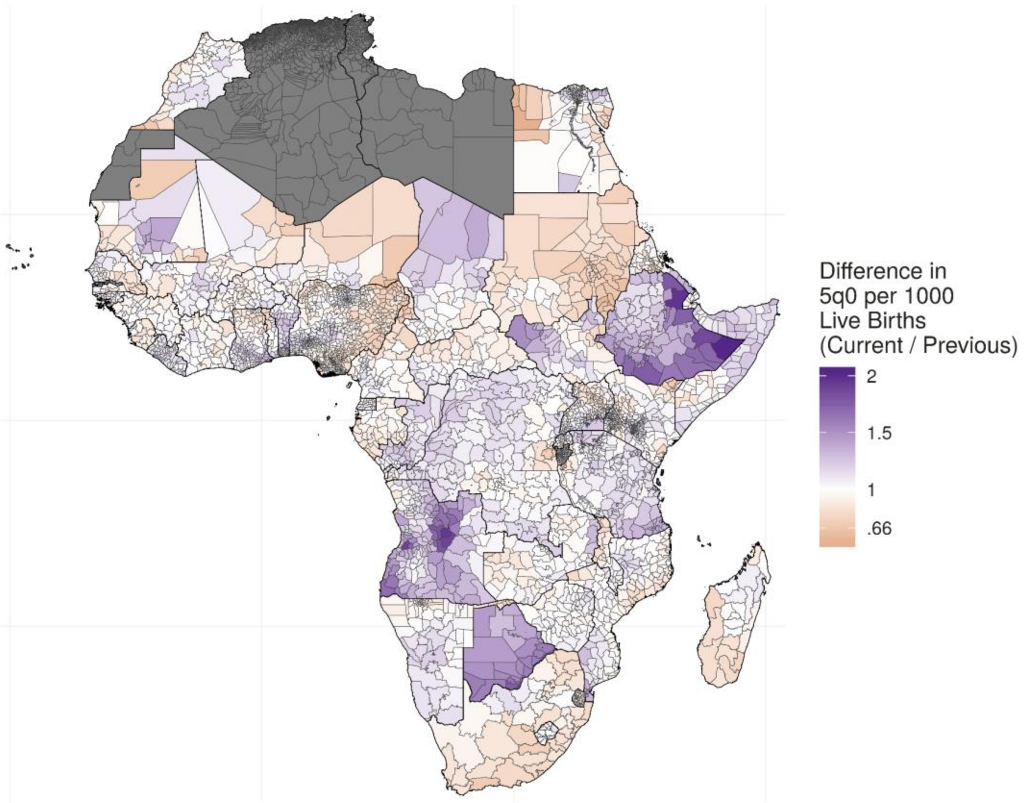

Supplementary Figure 5.18: Difference between current and previous estimates of under-5 mortality probability, 2015

Difference between current and previous estimates of under-5 mortality probability for the 2015 period (defined as 2013–2017) across all second administration units in the 46 countries analyzed in the previous analysis. The countries in gray were not analyzed in the previous analysis.

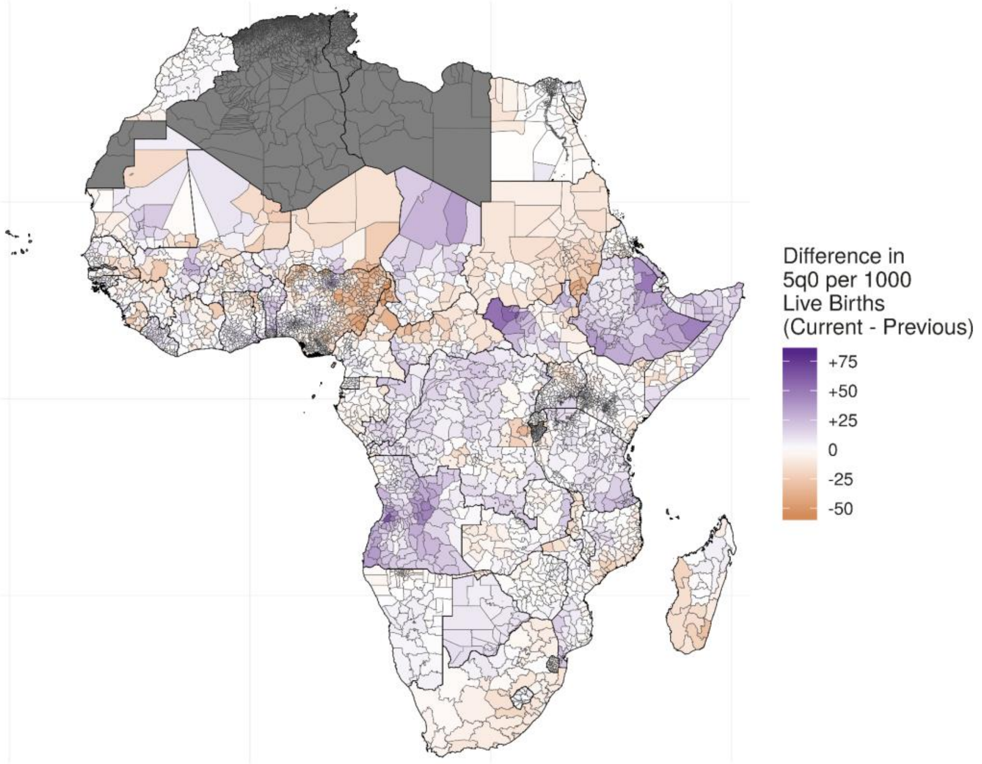

Supplementary Figure 5.19: Second administration units with significant difference between current and previous estimates of under-5 mortality probability, 2015

Second administration units with significant difference between current and previous estimates of under-5 mortality probability for the 2015 period (defined as 2013–2017). Areas in yellow had overlapping 95% uncertainty intervals. Areas in red have estimates in the current analysis which are higher and whose 95% UIs do not overlap with those from the previous analysis. Areas in blue have estimates in the current analysis which are lower and whose 95% UIs do not overlap with those from the previous analysis. Areas in gray were not analyzed in the previous analysis.

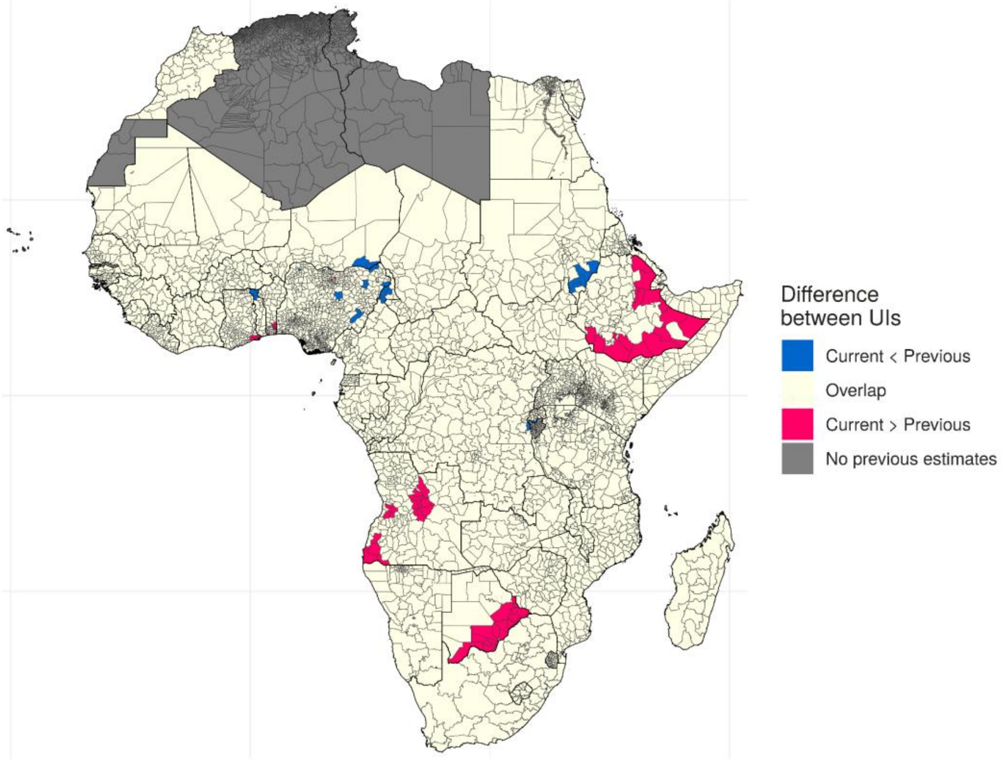

Supplementary Table 5.3: Data sources added in SSA countries since the previous analysis

GHDx ID	Country	Year	Data type	Survey Title
218555	Angola	2016	CBH	Angola Demographic and Health Survey 2015–2016
367347	Benin	2002	SBH	Benin Population and Housing Census 2002 – IPUMS
206075	Benin	2014	CBH	Benin Multiple Indicator Cluster Survey 2014
22125	Botswana	2008	CBH	Botswana Family Health Survey 2007–2008
286766	Burundi	2016	CBH	Burundi Demographic and Health Survey 2016
244455	Cameroon	2014	CBH	Cameroon Multiple Indicator Cluster Survey 2014
218611	Cote d'Ivoire	2016	CBH	Côte d’Ivoire Multiple Indicator Cluster Survey 2016
153507	Ethiopia	2014	CBH	Ethiopia Mini Demographic and Health Survey 2014
218568	Ethiopia	2016	CBH	Ethiopia Demographic and Health Survey 2016
160576	Ghana	2008	CBH	Ghana District Multiple Indicator Cluster Survey 2007–2008
286788	Ghana	2016	SBH	Ghana Malaria Indicator Survey 2016
218572	Ghana	2017	CBH	Ghana Special Demographic and Health Survey 2017
303458	Guinea	2016	CBH	Guinea Multiple Indicator Cluster Survey 2016
367585	Lesotho	2006	SBH	Lesotho Population and Housing Census 2006 – IPUMS
286768	Liberia	2016	SBH	Liberia Malaria Indicator Survey 2016
248224	Mali	2015	CBH	Mali Multiple Indicator Cluster Survey 2015
267343	Mauritania	2015	CBH	Mauritania Multiple Indicator Cluster Survey 2015
157060	Mozambique	2015	CBH	Mozambique AIDS Indicator Survey 2015
218613	Nigeria	2017	CBH	Nigeria Multiple Indicator Cluster Survey with National Immunization Coverage Survey Supplement 2016–2017
234733	Republic of the Congo	2014	CBH	Congo Multiple Indicator Cluster Survey 2014–2015
367645	Rwanda	2012	SBH	Rwanda Population and Housing Census 2012 – IPUMS
350836	Rwanda	2017	SBH	Rwanda Malaria Indicator Survey 2017
287639	Senegal	2015	CBH	Senegal Dakar Multiple Indicator Cluster Survey 2015–2016
286772	Senegal	2016	CBH	Senegal Continuous Demographic and Health Survey 2016
353526	Senegal	2017	CBH	Senegal Continuous Demographic and Health Survey 2017
286773	Sierra Leone	2016	SBH	Sierra Leone Malaria Indicator Survey 2016

218619	Sierra Leone	2017	CBH	Sierra Leone Multiple Indicator Cluster Survey 2017
20798	South Africa	2003	SBH	South Africa Demographic and Health Survey 2003–2004
280803	South Africa	2016	SBH	South Africa Community Survey 2016
350798	Tanzania	2017	SBH	Tanzania Malaria Indicator Survey 2017
91506	The Gambia	2010	SBH	Gambia Multiple Indicator Cluster Survey 2010
359318	Togo	2017	SBH	Togo Malaria Indicator Survey 2017
264959	Uganda	2014	SBH	Uganda Living Standards Measurement Survey – Integrated Survey on Agriculture 2013–2014
286780	Uganda	2016	CBH	Uganda Demographic and Health Survey 2016
367747	Zimbabwe	2012	SBH	Zimbabwe Population Census 2012 – IPUMS

5.4.2 Covariates out of range

Covariate values were extracted at each data point based on year and location. These values were then used in the statistical model, and the spatial covariate surfaces were then used in prediction. We checked the extent to which geospatial covariate surfaces had grid cell values that fell out of the range of values observed in the model training data.

Overall, we found we had good coverage of covariates, with training data representing the range of covariates values used in predictions in most areas. Comparing against the population surface from WorldPop, we found that only 0.29% of the total population lived in areas where at least one covariate had values out of the range observed in the training data. Supplementary Figure 5.20 shows the percentage of population across each year from 2000 to 2017 which lived outside of the range of at least one covariate. 2001 was the year with the greatest covariate coverage, with only 0.050% of the population living outside the training data range of at least one covariate, while 2017 was the year with the worst coverage, with 0.84% of the population living outside that training data range of at least one covariate. There is a trend toward less population coverage in more recent years because there is less data in more recent years, due to the fact that birth histories supply retrospective time series. Supplementary Figure 5.21 summarizes the locations of these uncovered areas, with the color scale indicating the percentage of covariates used in the area which were out of range, across all years. Areas with darker blue colors had more of their predictive covariates out of range in the training data.

Supplementary Figure 5.20: Percentage of the population living outside of the training data range of at least one covariate

Percentage is calculated for each year modeled 2000–2017.

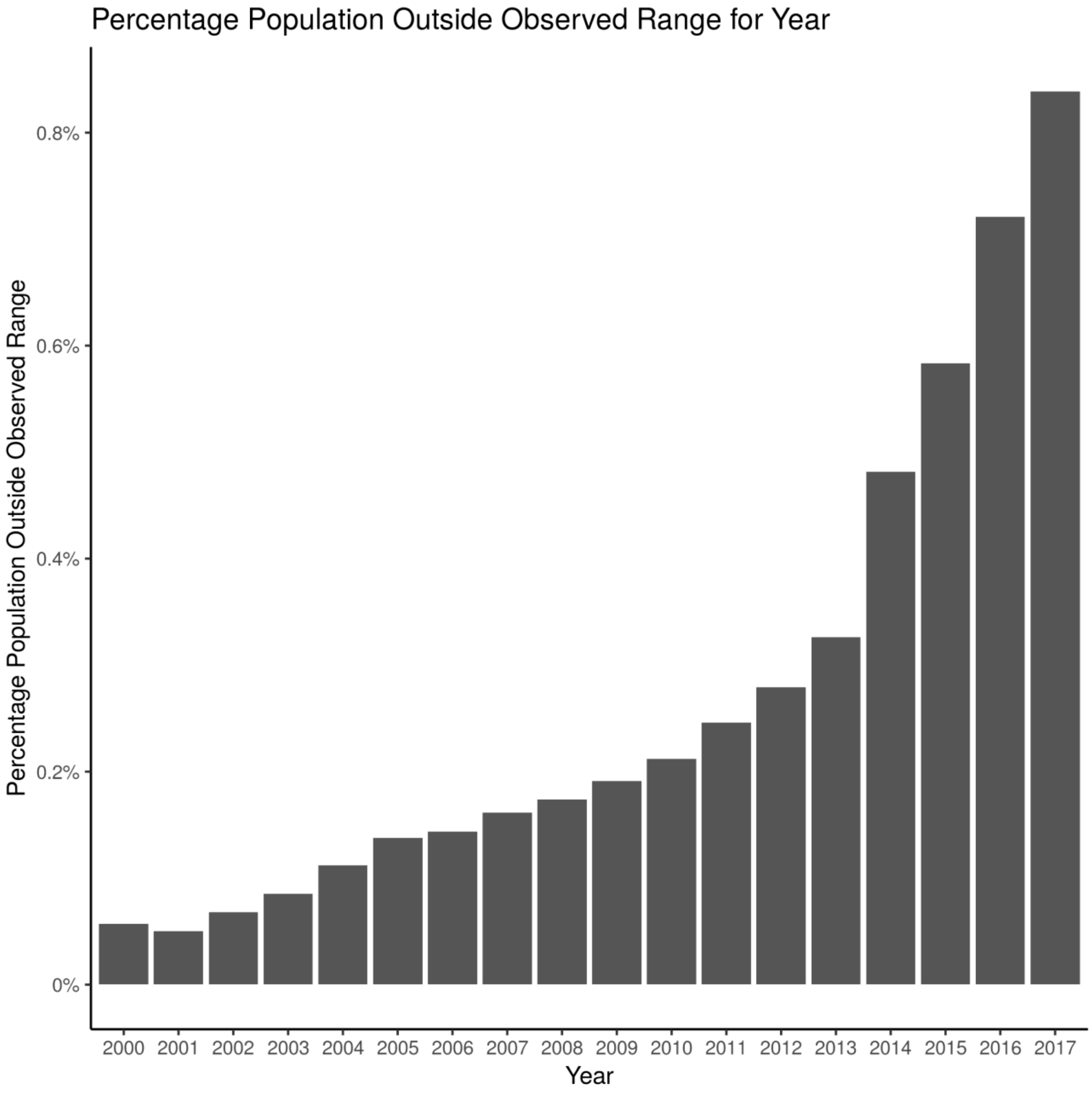

Supplementary Figure 5.21: Grid cells with covariate values outside the range of values observed in the model training data

Scale indicates the percentage of covariates covering any given grid cell with values not in range of training data, with increasing number of covariates as the color scales to blue. Areas in gray had covariate values that were completely in the range observed in the training data. This was most common in very sparsely populated desert or jungle areas.

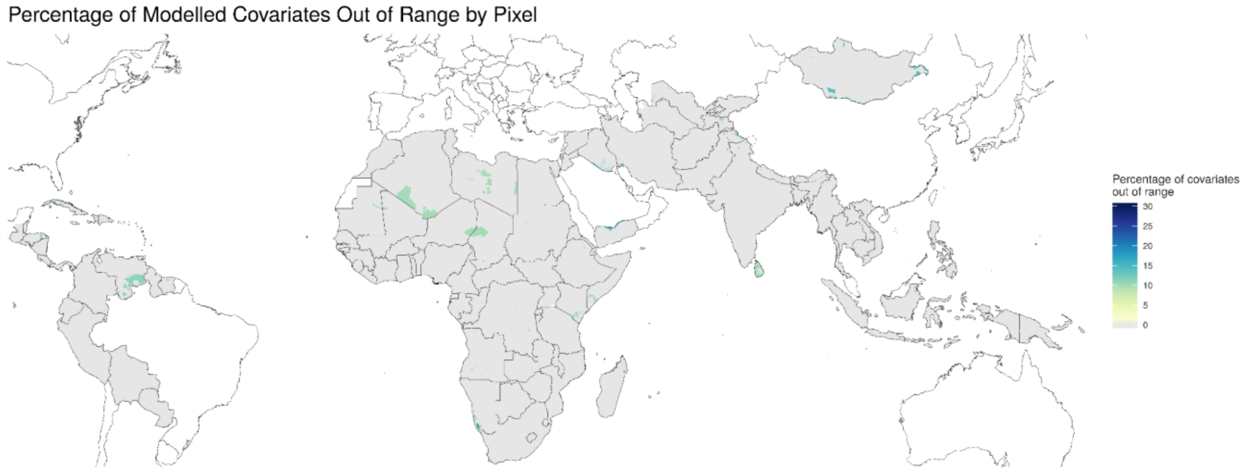

5.4.3 National-level time series plots

Simple time series plots with raw data overlaid on time series can be helpful to assess general in sample fits on the full model. Supplementary Figure 5.22 shows pre-calibration trends and data aggregates at the national level for under-5 mortality probability. Fitted model estimates follow the input data. Furthermore, we can see that uncertainty intervals tend to be wider where data are lacking, see for example Equatorial Guinea (GNQ). Supplementary Figure 5.23 shows the same tends after calibration to GBD; the similarity with Supplementary Figure 5.22 indicates that calibration only had a small effect at this level, and that the data and estimates generally agreed with estimates produced via GBD methodology. It is evident that certain aspects of the GBD analytical process did affect results in certain countries. For example, in Haiti (HTI) a significant death shock due to the 2010 earthquake was not evident in the raw data or geostatistical model. GBD has developed methodologies to deal with such fatal discontinuities[7] and by calibrating to GBD estimates, we can take advantage of these improvements. Future research efforts should focus on integrating geographically specific fatal discontinuities into geospatial mortality estimation.

Supplementary Figure 5.22: Raw data aggregates and estimated trends before calibration to GBD, under-5 mortality probability, 2000 to 2017

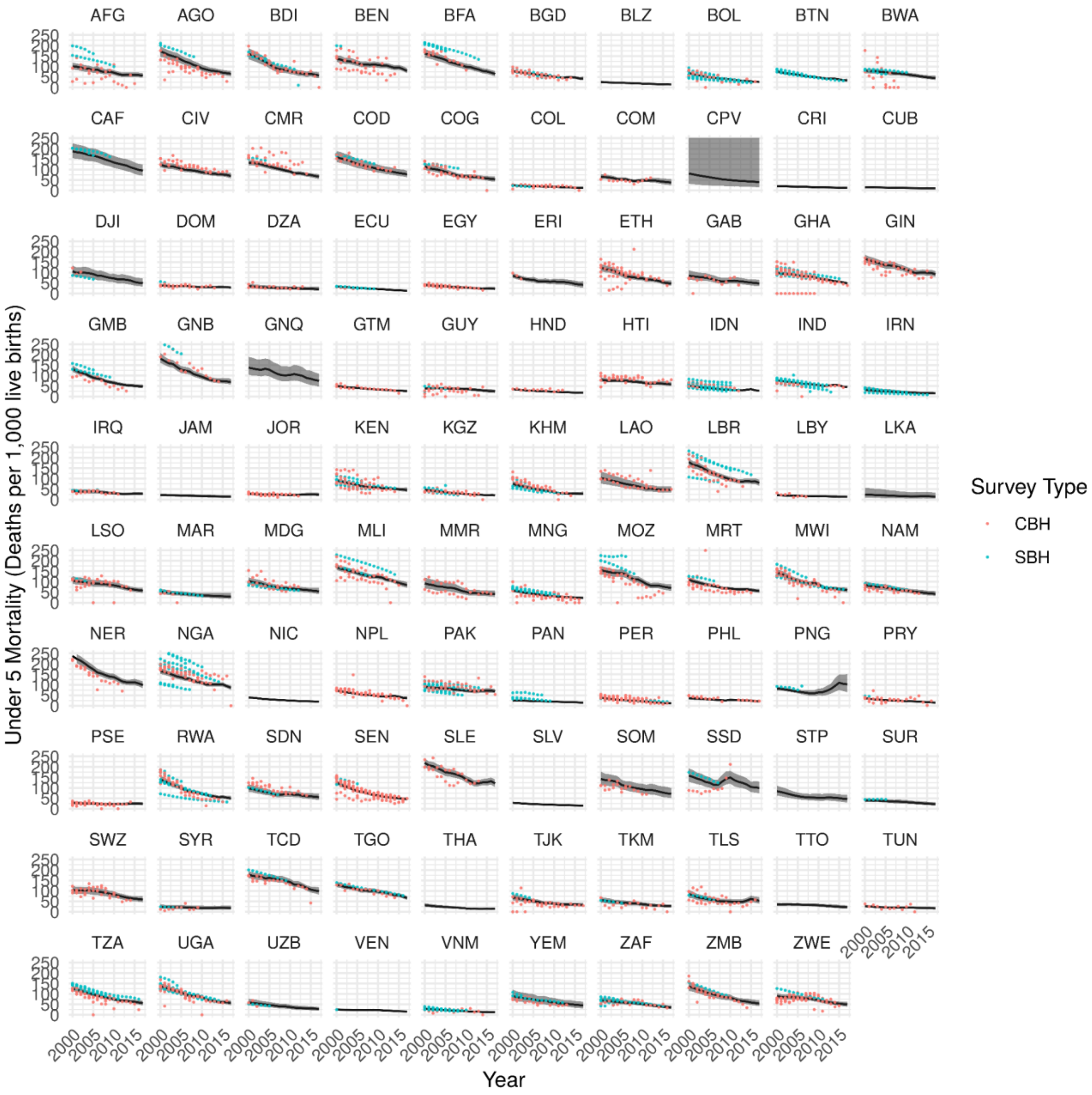

Supplementary Figure 5.23: Raw data aggregates and estimated trends after calibration to GBD, under-5 mortality probability, 2000 to 2017

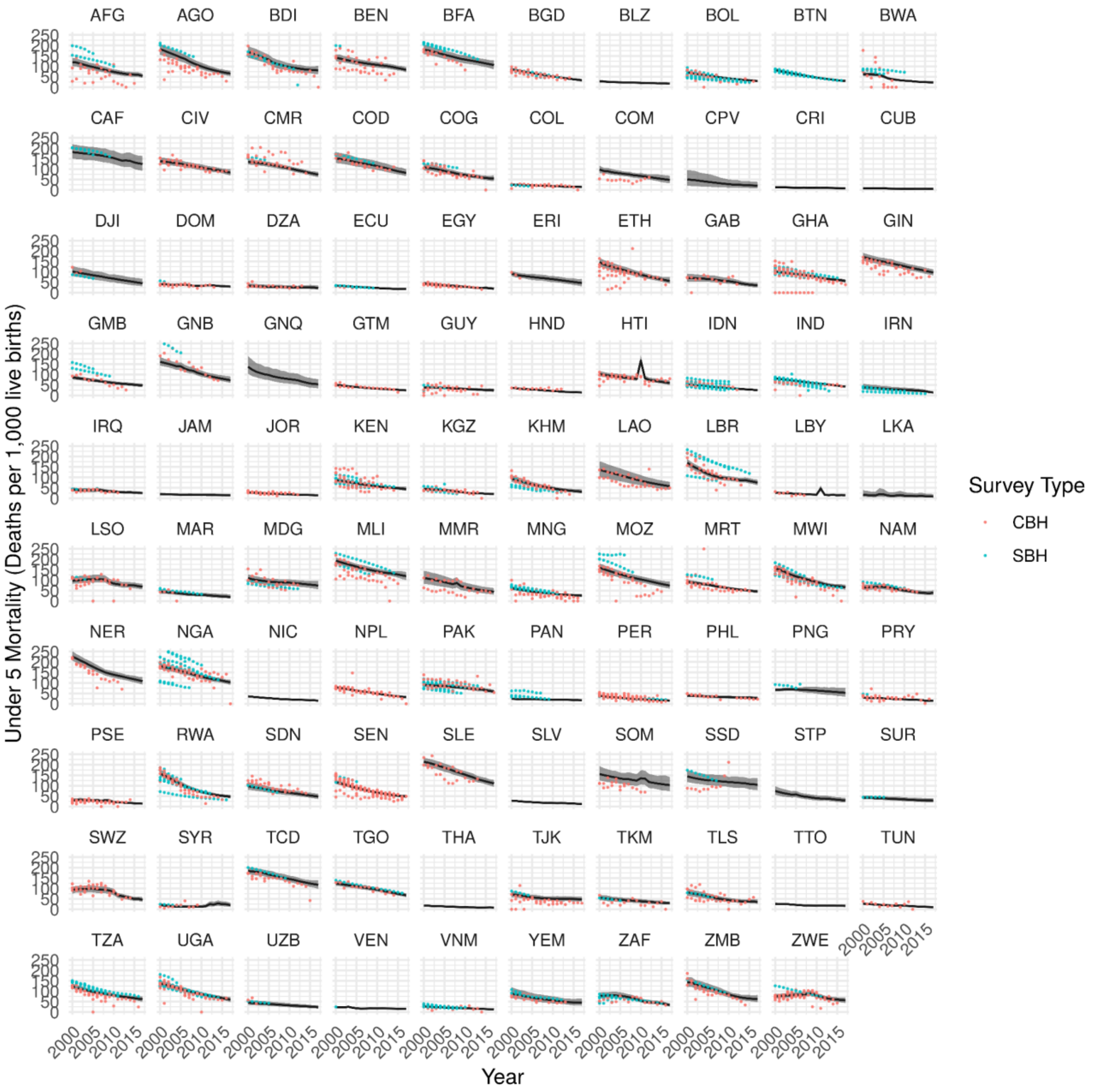

Supplementary Figure 5.24: Raw data aggregates and estimated trends before calibration to GBD, infant mortality probability, 2000 to 2017

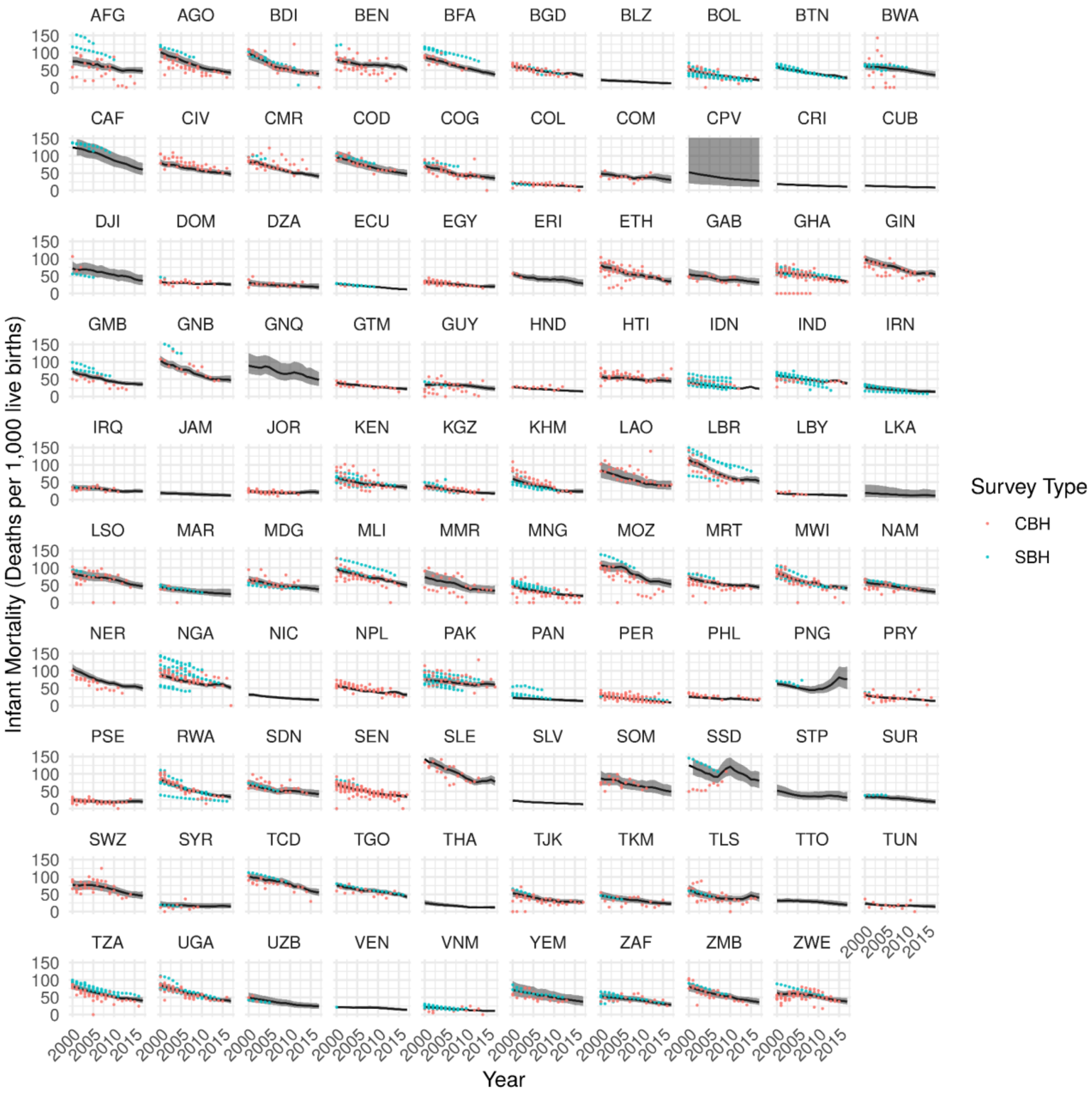

916 *Supplementary Figure 5.25: Raw data aggregates and estimated trends after calibration to GBD, infant*
917 *mortality probability, 2000 to 2017*

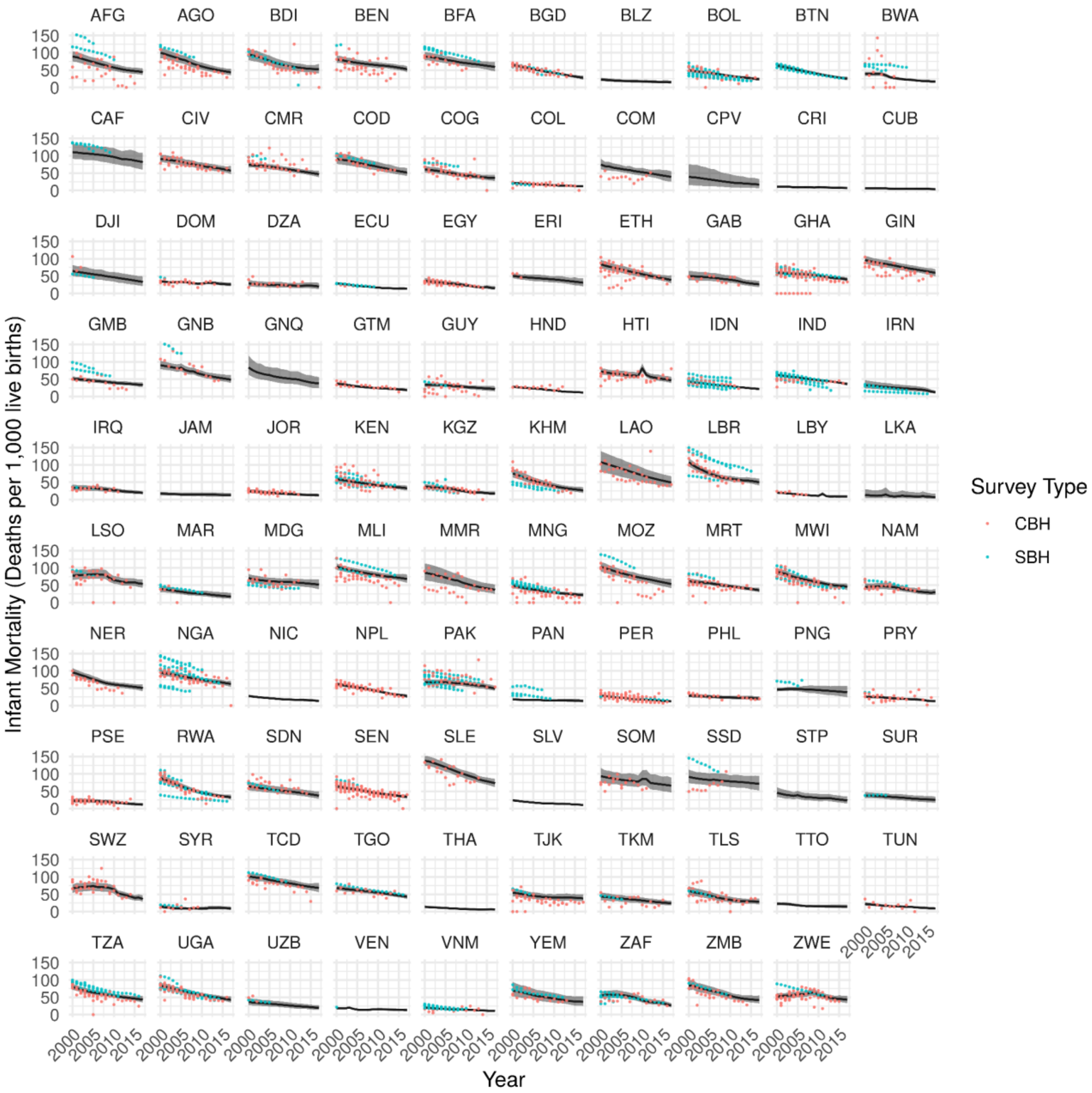

918

919

Supplementary Figure 5.26: Raw data aggregates and estimated trends before calibration to GBD, neonatal mortality probability, 2000 to 2017

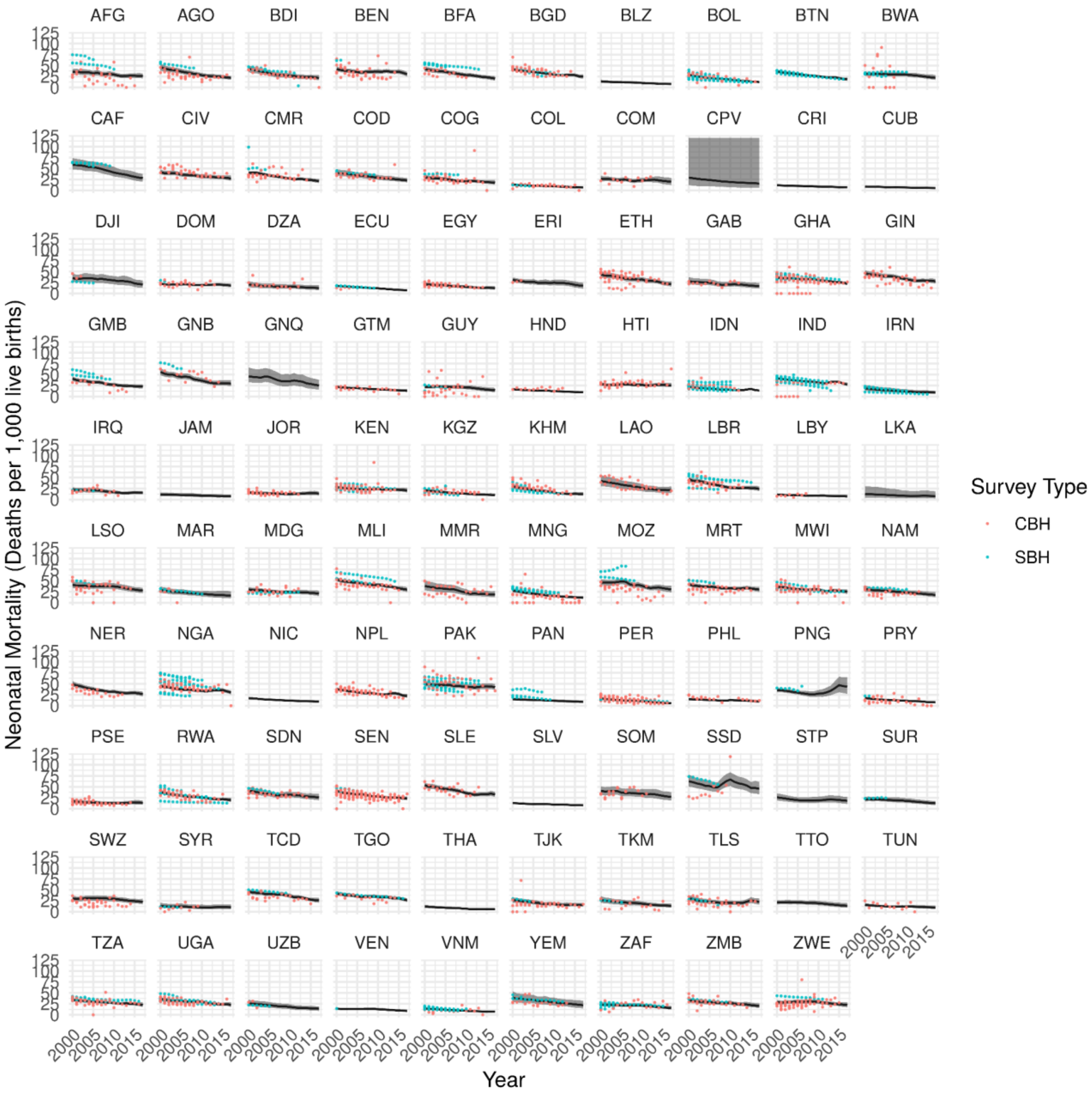

Supplementary Figure 5.27: Raw data aggregates and estimated trends after calibration to GBD, neonatal mortality probability, 2000 to 2017

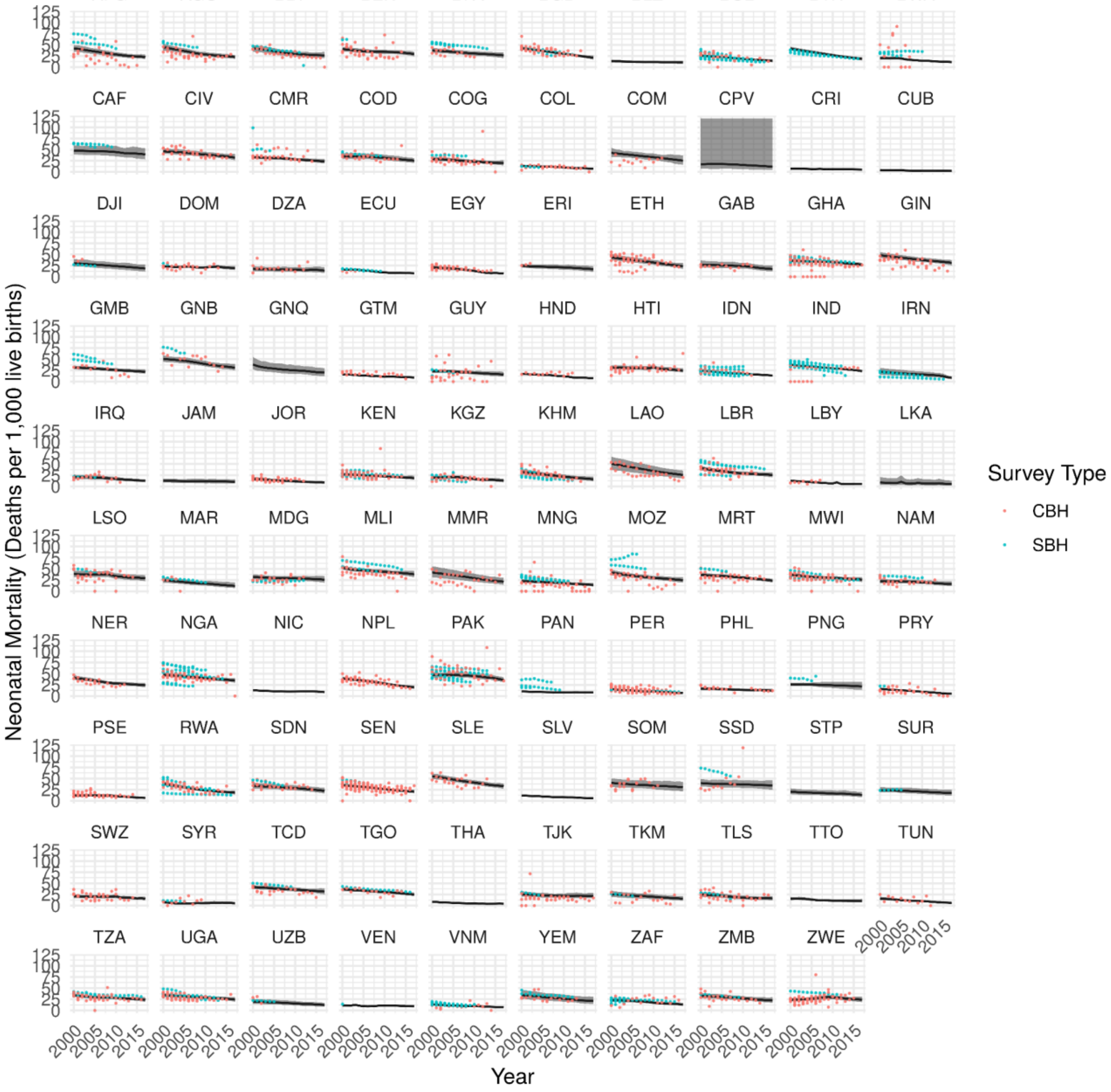

5.4.4 Migration sensitivity analysis

All birth histories are associated with the location of the household of the mother at the time of survey or census. Since birth histories are used to construct retrospective mortality trends in specific locations, it is possible that life and mortality experiences are being counted in places where they did not occur. Unfortunately, many of the data sources we rely on do not ask about migration history. We undertook a sensitivity analysis to better understood how this could be affecting our results.

We conducted a focused sensitivity analysis on six countries with varying levels of internal migration[31] and available complete birth history survey data conducted since 2002 with migration questions to draw from. These were Bangladesh (BGD), Kenya (KEN), Nepal (NPL), Peru (PER), Tanzania (TZA), and Uganda (UGA). In BGD, 2 of the 4 surveys checked asked a usable migration question. In KEN 2 of 5 did; in NPL 2 of 5; in PER 8 of 8; in TZA 3 of 5; and in UGA 2 of 4.

Using questions asking about how long the mothers lived continually at their current residence, we first assessed the rates of retrospective attrition in each country. In Bangladesh, Nepal, and Peru, around 90% of mothers surveyed had been in their current residence for the past five years. In Kenya, Uganda, and Tanzania, five-year relocation rate was closer to 80%. In the 2016 Peru DHS, we found nearly 60% relocation rate going back to 2000. It is important to note that as years since survey increase, the proportion of data contribution to the model decreases. For example, in the case of estimates from the 2016 Peru DHS, there have also been nine other surveys also contributing information for 2000. In the full global model dataset, about 50% of the total retrospective data come from surveys/censuses that were collected within the past five years.

National-level trends are not sensitive to the assumption of including children born in different locations. This assumption would cause problems if there are considerable differences in mortality between the movers and long-term stayers. Below are national-level aggregate trends of U5MR calculated from each of the surveys in the six example countries. The red trends represent the full dataset, as used in our model. The blue trends represent mortality rate trends calculated only from children who were born in the current residence. Differences are very minimal, meaning that any difference between the mortality experiences of children pre-migration and those born in current residence did not obviously bias results at this level of aggregation.

Supplementary Figure 5.288: National-level trends, comparing estimates made using the full survey of respondents, versus keeping only those children born in current residence

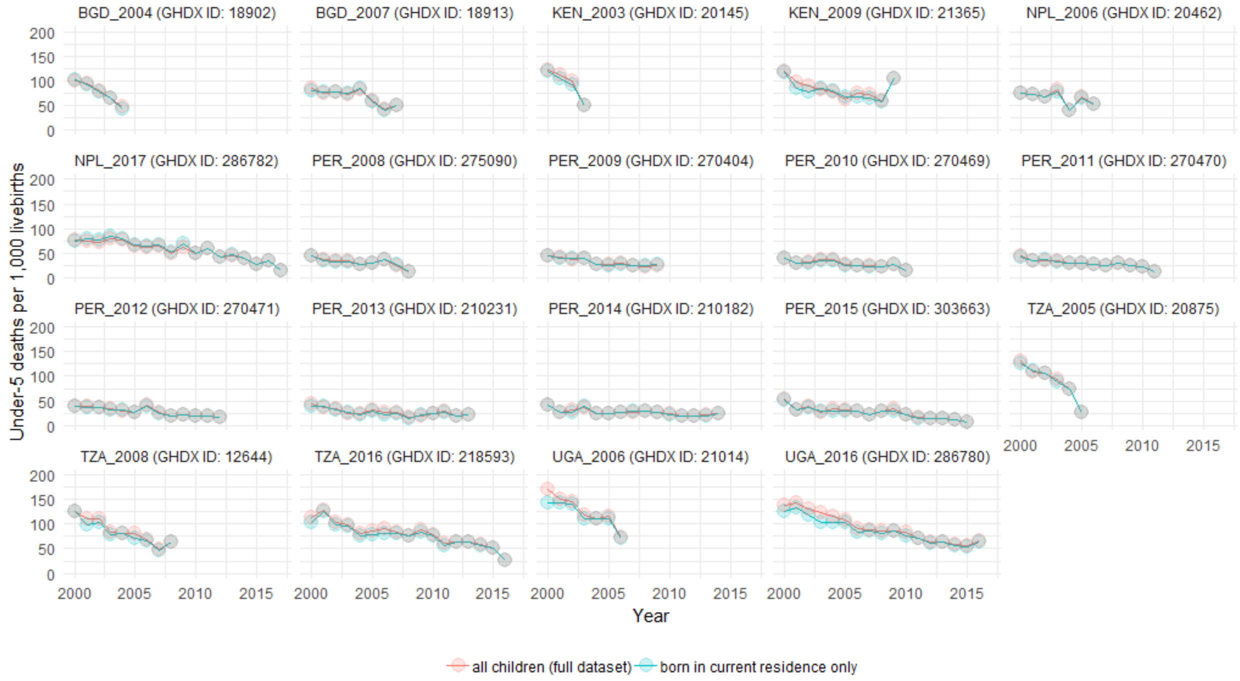

Of true interest, though, is how sensitive our subnational results are to this assumption. Using these same surveys, we fit our geospatial model for each country twice: once using the full dataset assembled of sources with a migration question, and once using the same surveys but keeping only keeping birth histories for children born in the current residence. These tests are somewhat conservative, because children of women who changed residences within the same village or neighborhood are excluded as well. The plots below compare our results for a select number of years for each country. The “sub” models only include the subset of non-movers, while the “full” model includes everyone. In order to minimize the number of plots here, we are only showing estimates from the last year of data availability for each country.

Supplementary Figure 5.299: Map and scatterplot comparisons between full data and a subset of those who did not move, Bangladesh, 2007. Second administrative area R-squared for years 2000, 2010, and 2017 = 0.89, 0.96, 0.90.

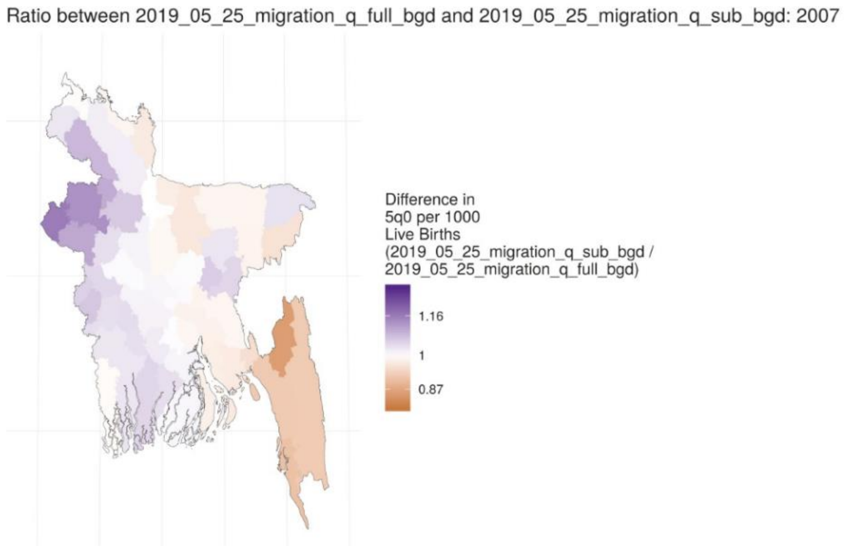

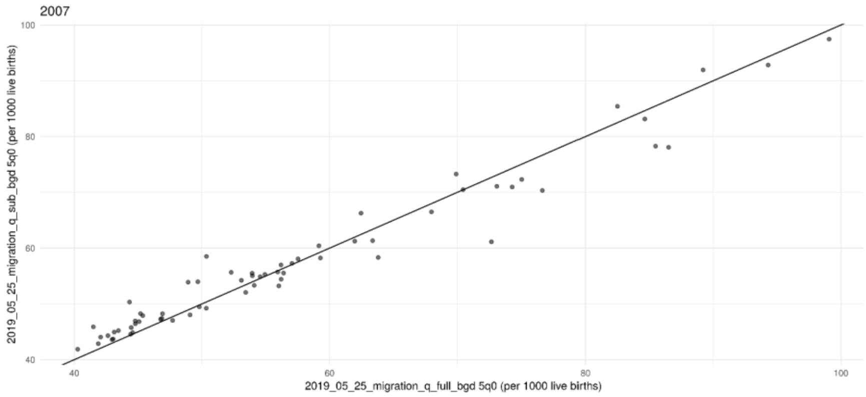

Supplementary Figure 5.30: Map and scatterplot comparisons between full data and a subset of those who did not move, Kenya, 2009. Second administrative area R-squared for years 2000, 2010, and 2017 = 0.75, 0.71, 0.65.

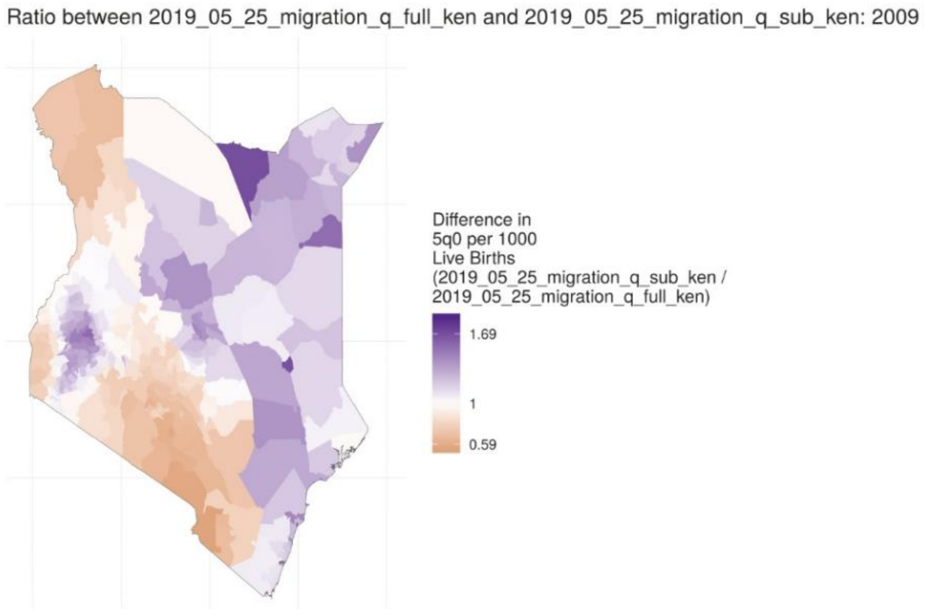

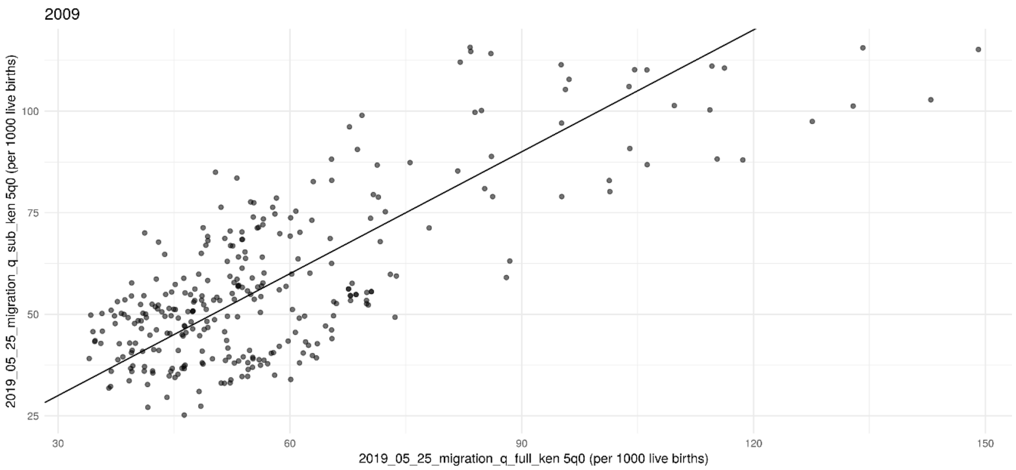

Supplementary Figure 5.31: Map and scatterplot comparisons between full data and a subset of those who did not move, Nepal, 2017. Second administrative area R-squared for years 2000, 2010, and 2017 = 0.99 for all years.

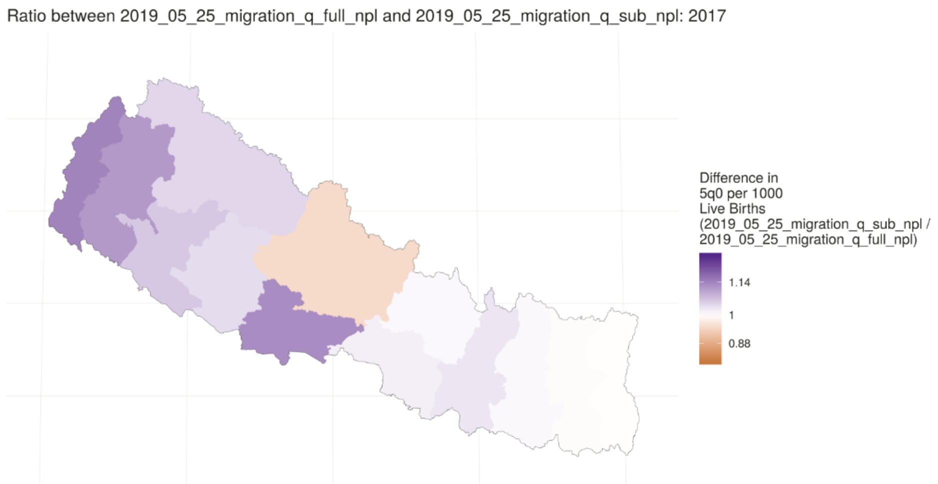

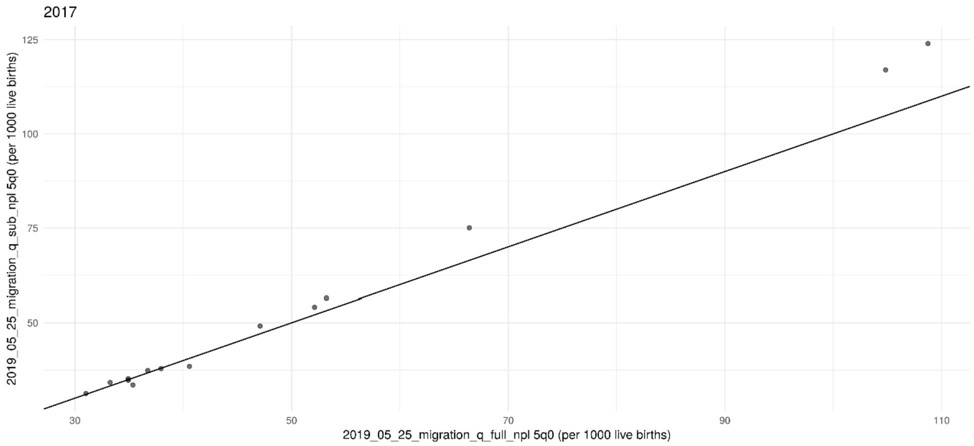

Supplementary Figure 5.32: Map and scatterplot comparisons between full data and a subset of those who did not move, Peru, 2015. Second administrative area R-squared for years 2000, 2010, and 2017 = 0.98, 0.98, 0.97.

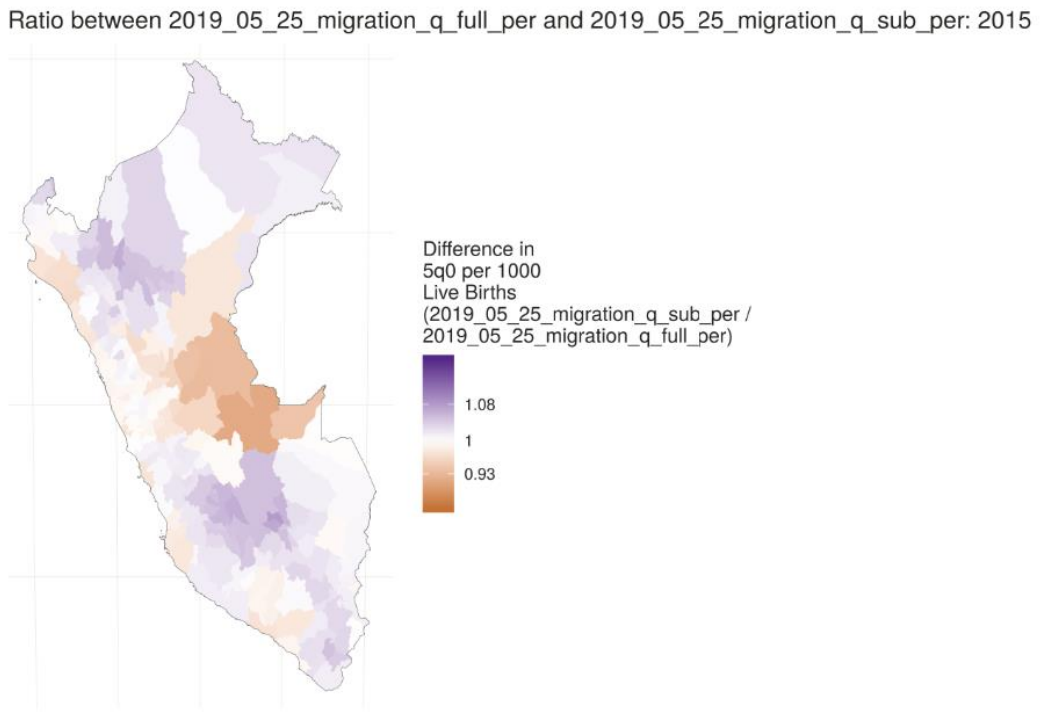

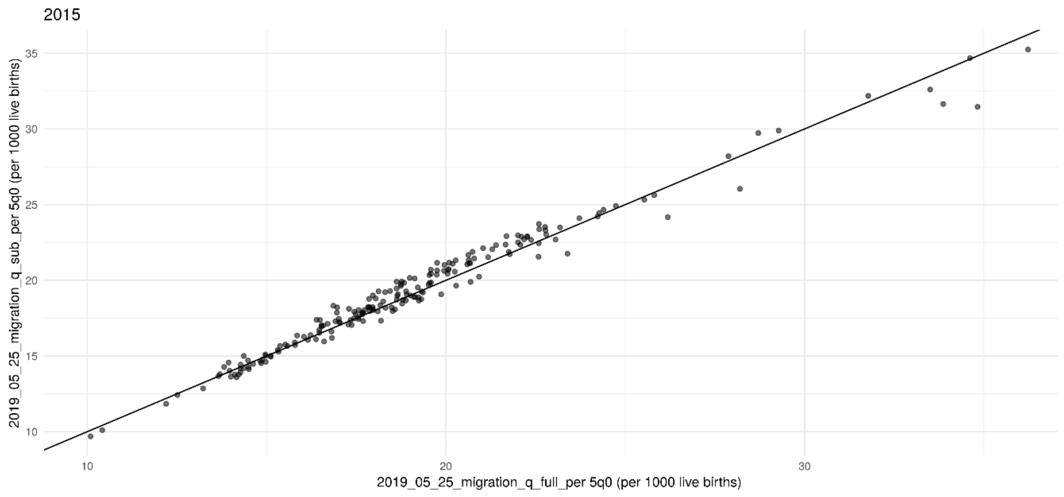

Supplementary Figure 5.33: Map and scatterplot comparisons between full data and a subset of those who did not move, Tanzania, 2008. Second administrative area R-squared for years 2000, 2010, and 2017 = 0.54, 0.58, 0.61.

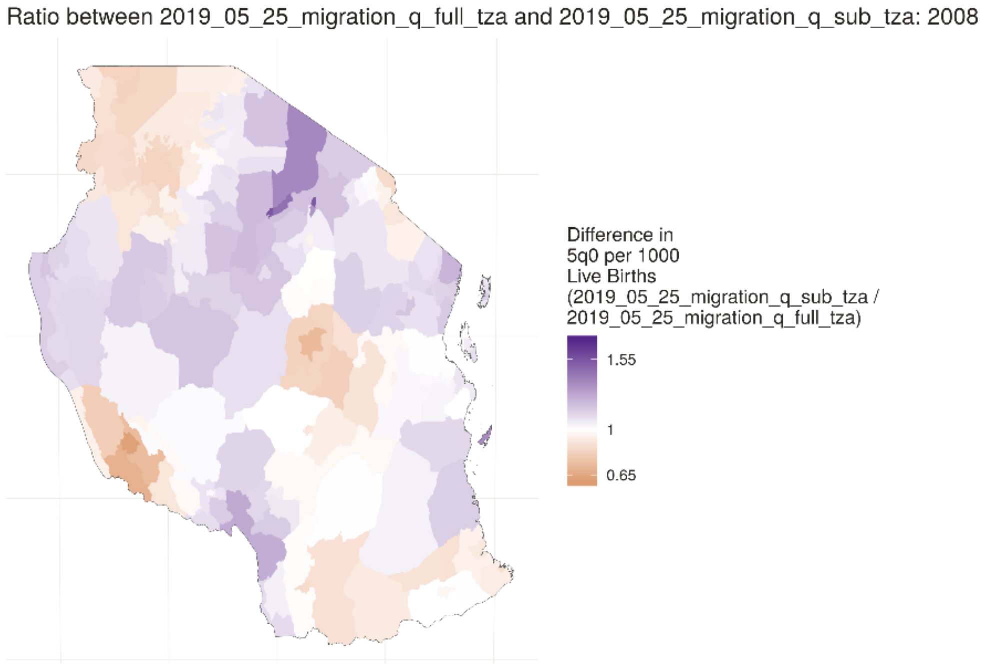

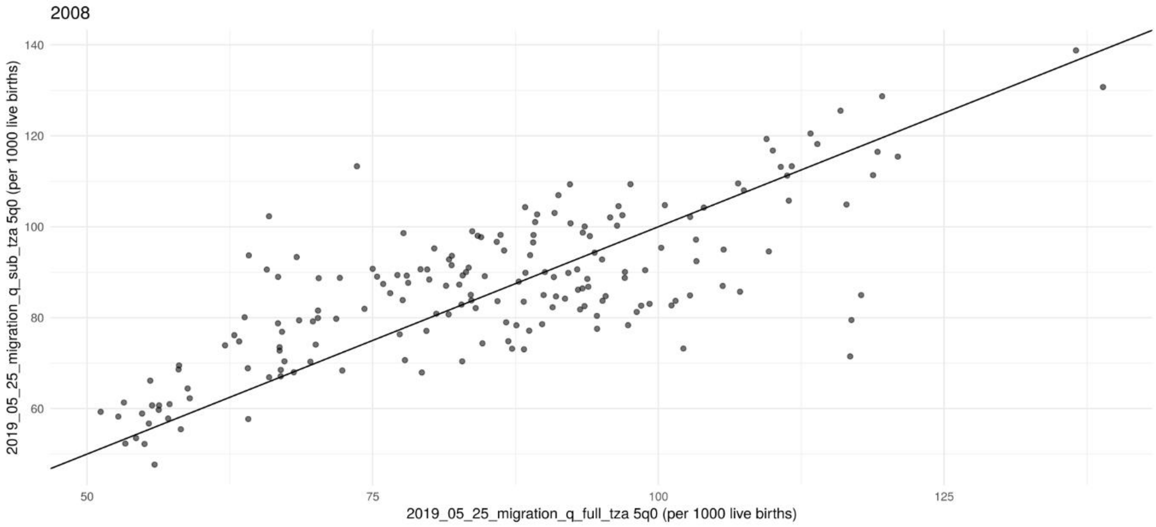

Supplementary Figure 5.34: Map and scatterplot comparisons between full data and a subset of those who did not move, Uganda, 2016. Second administrative area R-squared for years 2000, 2010, and 2017 = 0.58, 0.65, 0.64.

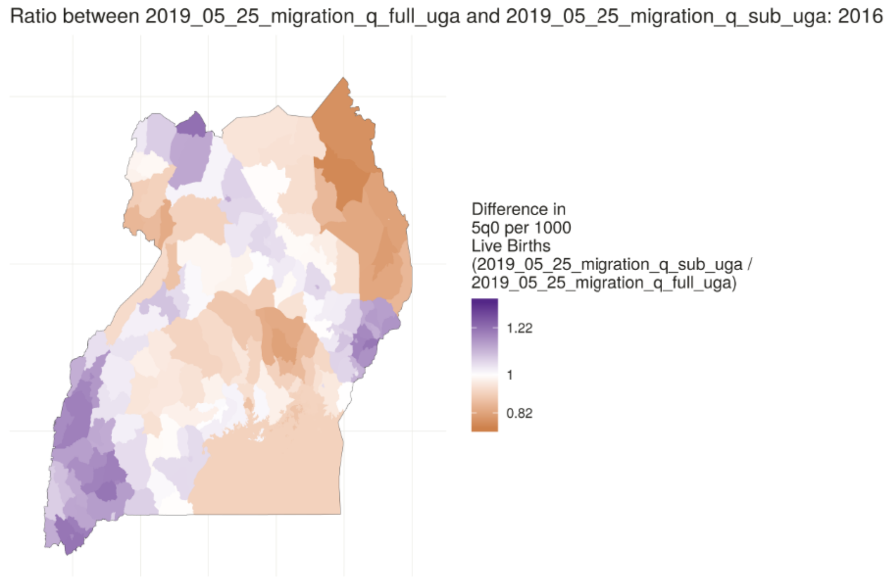

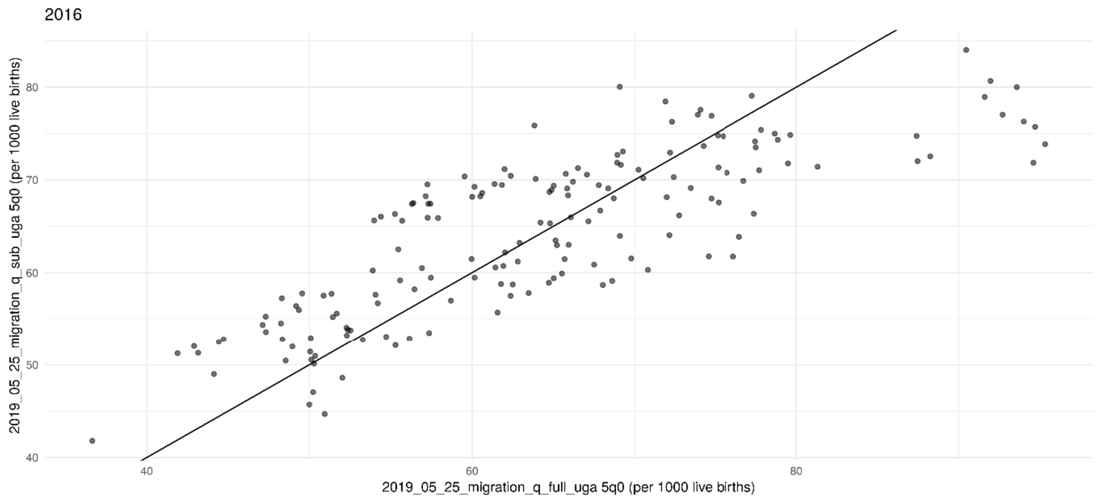

We ran a version of the full 99-country model only keeping retrospective data from only within five years since data collection. While we could not identify migrants in all data sources, this offered a simple way to potentially minimize retrospective attrition and test the sensitivity of our results. This again is a conservative test because we would expect that in most cases we would also drop the majority of children who have always resided where they were surveyed. In the plots below, we compare results from the full model run with all data (model run labelled as 2019_06_07_rtr) and the model run with only up to five years' retrospective data kept from each source (labelled 2019_06_11_migration_5yr). In the interest of space, we limit the plots we show to 2000 and 2010 but can supply the reviewer with all years upon request. In all we show three pairs of plots, each for 2000 and 2010, respectively, showing comparison between the estimates from the two model runs: the first pair of maps show the relative difference, the next pair shows a scatterplot comparing the two runs, and finally the last pair of maps

show where uncertainty intervals do not overlap. While there are some variations in the mean estimates (maps), the overall tendency is for estimates to remain quite similar (scatterplots). Where differences do remain, the vast majority are not significant (uncertainty intervals mostly remain overlapping, except for a few districts in India and Bangladesh). As would be expected, the differences are larger going back in time, since there is a larger difference in the input data further back in time.

Supplementary Figure 5.35: Maps showing relative difference between the full model run and a run where only five years of retrospective data were kept from each data source for 2000 and 2017

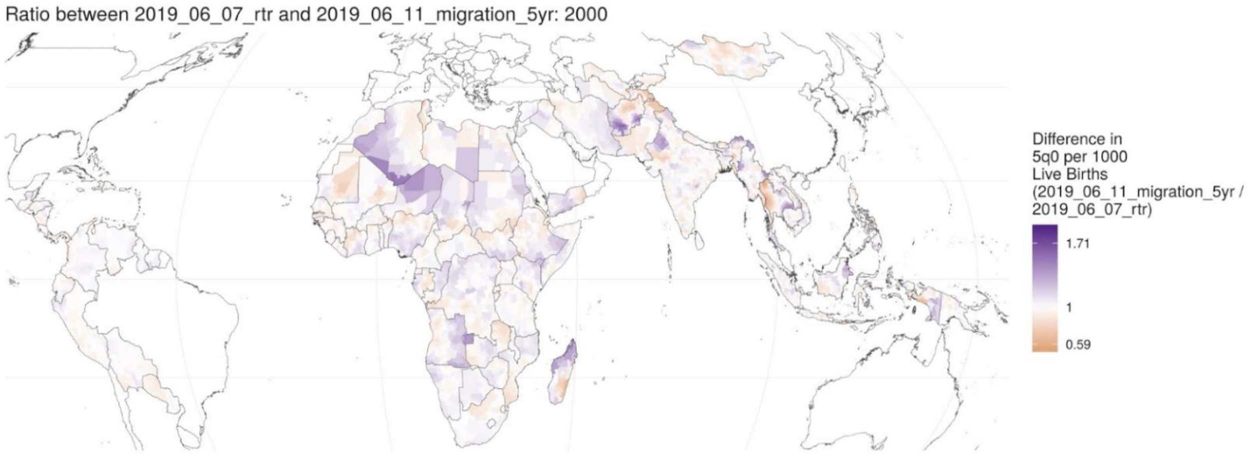

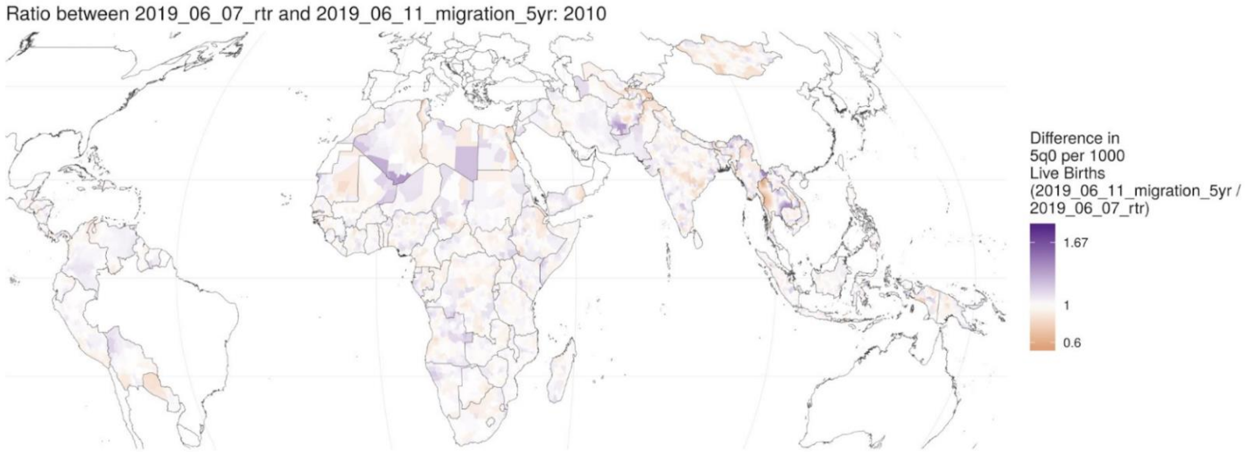

Supplementary Figure 5.36: Scatterplots showing difference between the full model run and a run where only five years of retrospective data were kept from each data source for 2000 and 2017

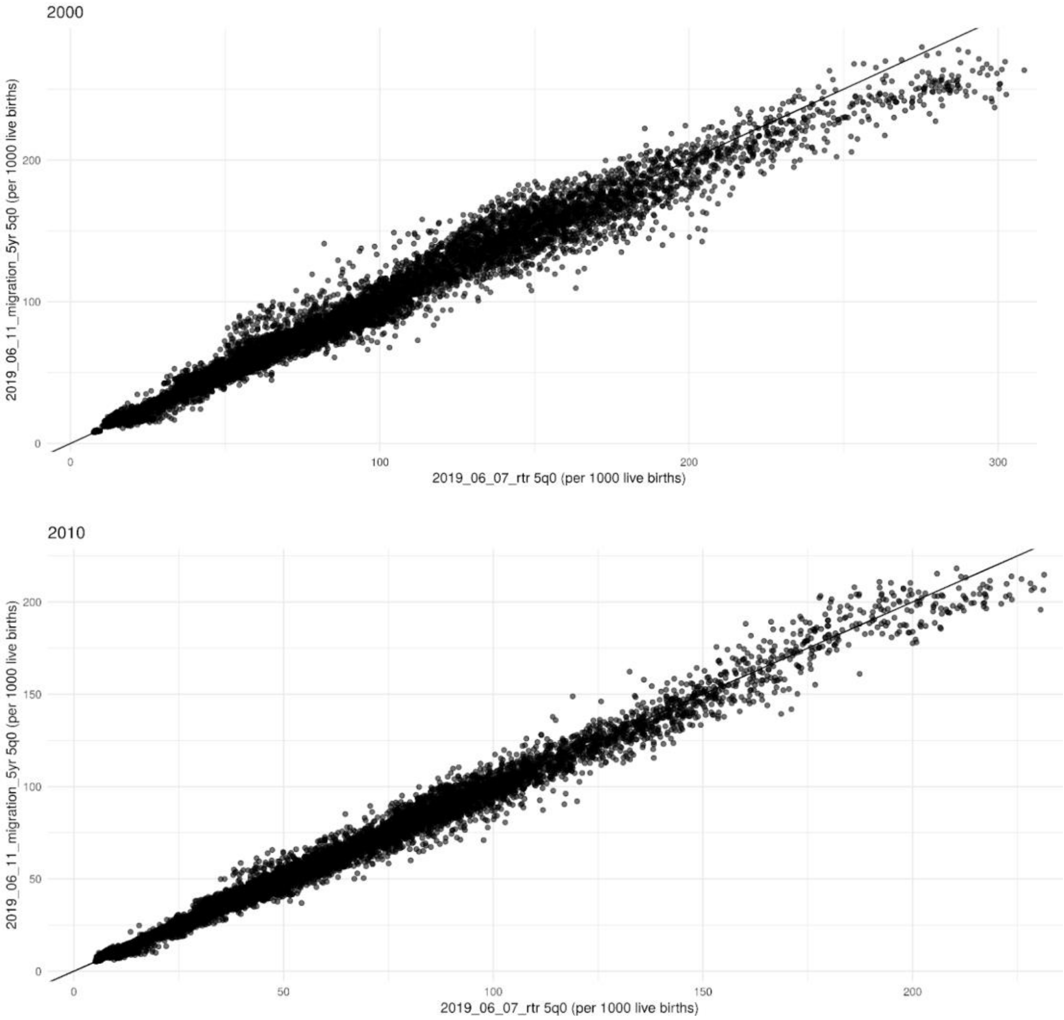

While migration is certainly a concern for subnational mapping of child mortality using survey and census data, our sensitivity analyses indicate that our estimates are generally robust to the assumptions we make, particularly since the tests we implemented here introduce biases which are likely greater than the effect of migration would be. In our deeper look at the six countries, we found that typically about half the available surveys ask a migration question with which to subset the sample; meaning that to use only a subset of non-movers would greatly reduce the number of data sources available to us.

5.4.5 Sources of reductions in deaths

In the paper, we describe how total child deaths, even in high-risk areas, are declining. This is despite population growth and high fertility in those areas. This is happening because declining mortality rates are outpacing population growth to still net a decline in deaths. In this section we unpack these dynamics.

First, the geographic area under a high mortality risk regime is shrinking. The maps below illustrate this.

Supplementary Figure 5.37: Maps for 2000 and 2017 indicating in green where the under-5 mortality probability was greater than 80 deaths per 1,000 live births

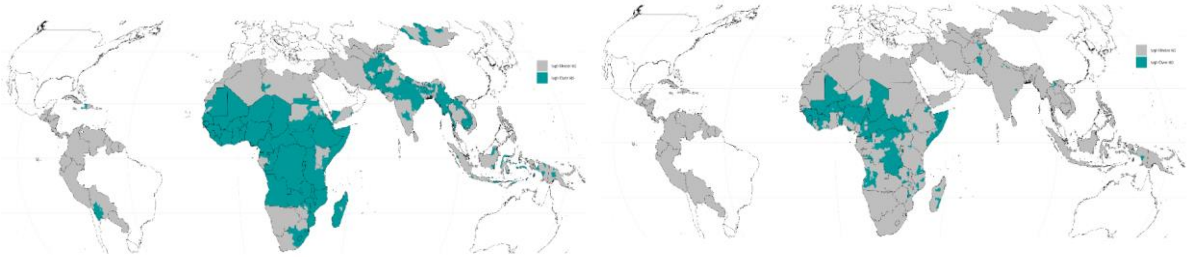

It is true that the total under-5 population in the study countries increased over the study period, from about 414 million in 2000 to 472 million in 2017, but as mortality risk declined, a greater share of that population is concentrated in lower mortality risk areas, as shown in the plot below of under-5 population distribution by U5MR for 2000 (red) and 2017 (blue).

Supplementary Figure 5.38: Distribution of under-5 population in 2000 and 2017, plotted by under-5 mortality probability strata

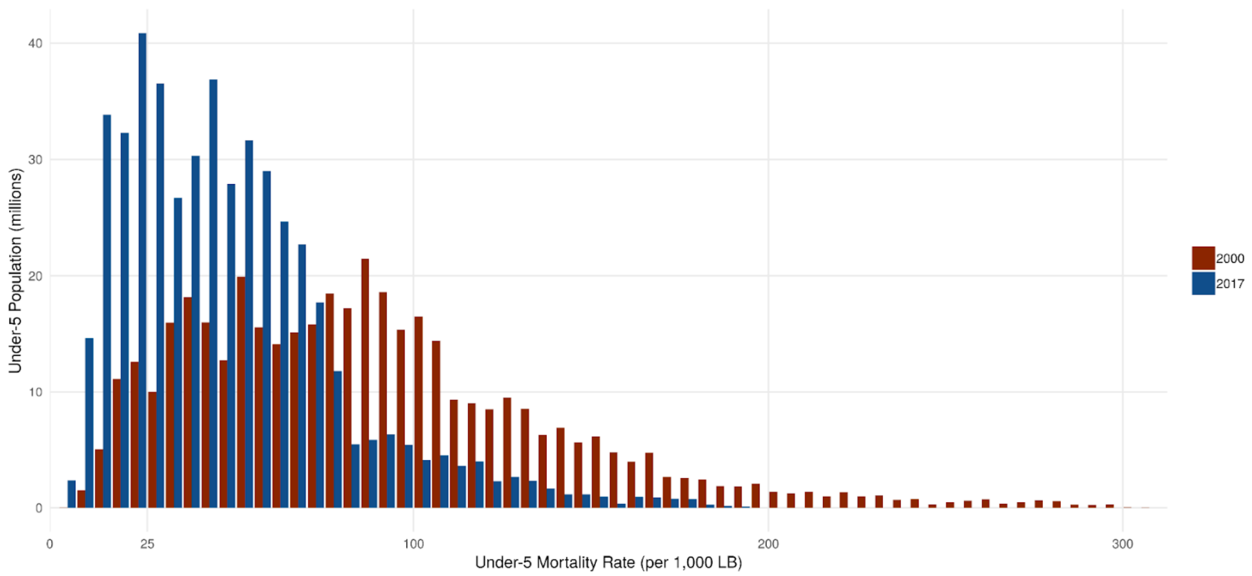

To better understand the contributions of mortality rate decline versus population change, we decomposed the change in deaths over time. In the plot below, we show the number of deaths by mortality risk strata in 2000 (band of 10 deaths per 1,000 live births). The blue line shows deaths in 2000, the red line shows how many deaths we would expect in those same areas (based on 2000 risk strata) in 2017 if the mortality rate had not changed but if the under-5 population had changed. The orange line shows the observed number of deaths in those same areas. Population increase counteracts the declines attributable to lower mortality rates. In other words, if the population increased substantially, the mortality rate would need to decrease even more for the number of deaths to decrease.

Increased deaths attributable to population growth are far greater in areas that were high-risk in 2000, meaning the decline in total deaths tends to be lower the higher the 2000 mortality risk was (particularly at the extreme end where U5MR>150). At the very low end (U5MR<25), we see instances where population change (decline in this case) contributed to a reduction in deaths. Overall, this plot shows that mortality rate declines outpace growth in the number of births, leading to fewer deaths overall.

Supplementary Figure 5.39: Arrow plot showing the counteracting forces of population change and mortality rate decline on total number of births. Plotted along an axis of mortality rate strata (bins of 10 per 1,000 livebirths) in 2000.

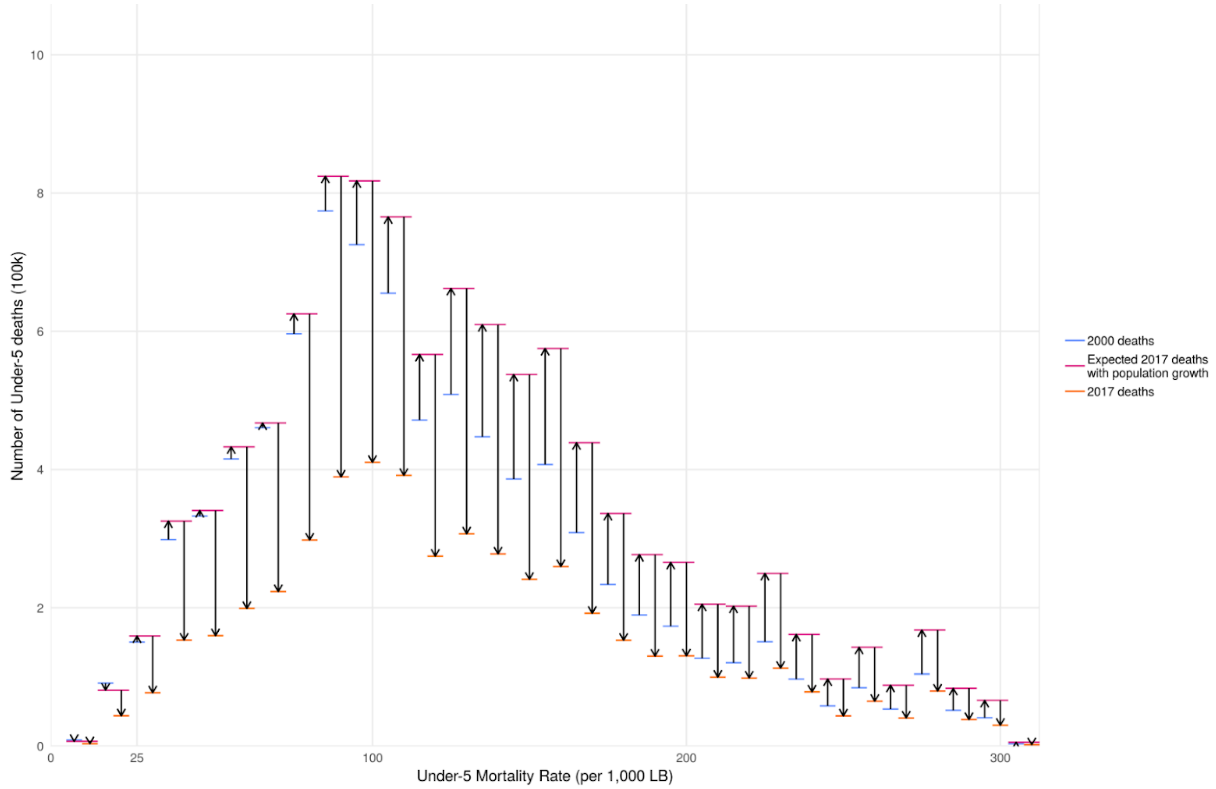

These dynamics can be seen in greater detail in maps. The first map below shows the absolute difference between deaths in 2000 (as indicated by the blue bar of the above graph) and deaths expected in 2017 in 100,000 deaths at the second administrative level, given population growth and holding U5MR constant (as indicated by the red bar of the above graph). We see large expected increases in most places based on the growing number of births:

Supplementary Figure 5.40: Map indicating the expected growth in deaths from 2000 to 2017 based on population alone (holding 2000 mortality rate constant)

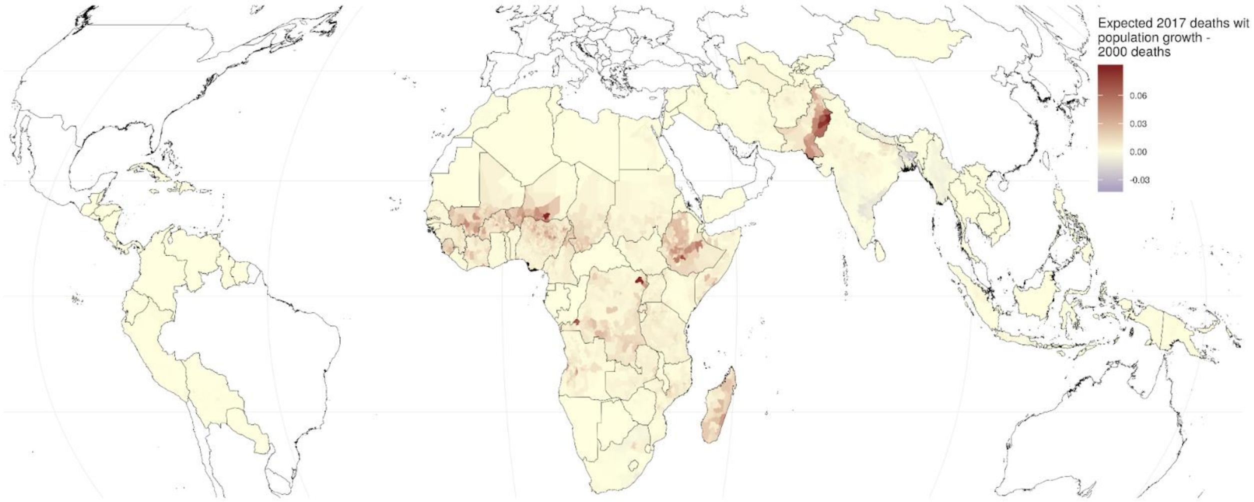

The next map shows the difference between that expected counterfactual in 2017 (2017 population with 2000 rate, as indicated by the red bar from the arrow diagram above), with the observed deaths in 2017 (as indicated by the orange bar from the arrow diagram above). Note that again the legend is in 100,000s of deaths, and the scale has changed significantly. The magnitude of these declines is generally much greater than the increases seen in the map above.

Supplementary Figure 5.41: Map indicating the difference between number of deaths in 2017 and the number expected based on population change alone

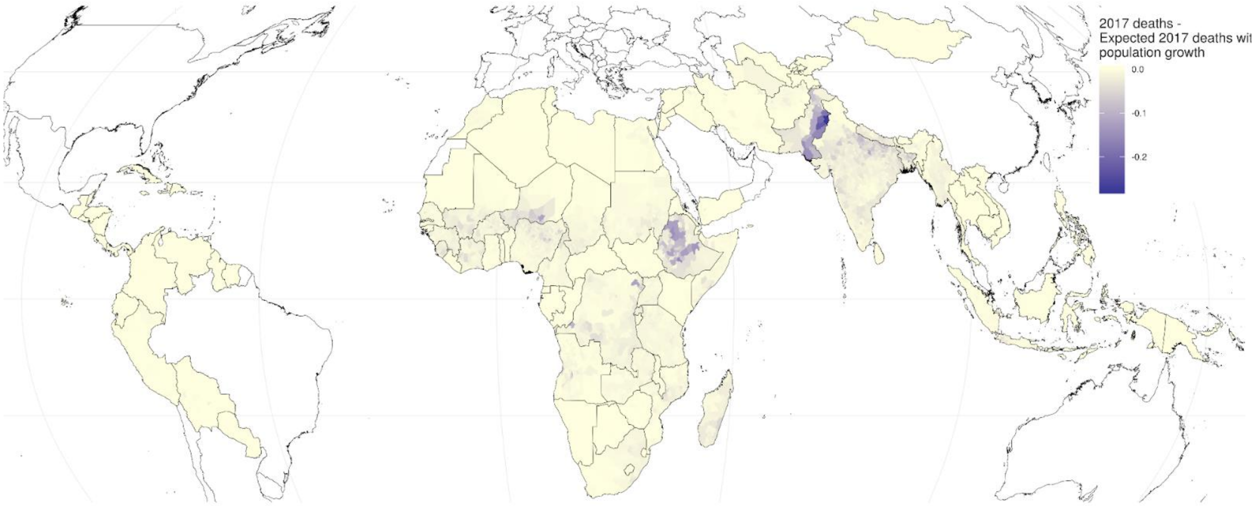

5.4.6 Incorporating uncertainty into estimates of progress

Because all estimates listed in the manuscript are based on samples from a posterior predictive distribution, or “draws”, we are able to incorporate uncertainty into our estimates of mortality decline. We defined mortality as significantly decreasing over the time period 2000-2017 if the 95% uncertainty intervals for the estimated mortality in an area (defined as the 2.5th and 97.5th quantiles of the sample distribution) were non-overlapping between 2000 and 2017. Using this criterion, we estimated that 60.3% of second-level administrative units had experienced significant declines in mortality between 2000 and 2017. This differs from an analysis examining changes in the mean estimate for mortality across the time period; if we define an improvement in U5M as a reduction in the mean estimate between 2000 and 2017, then 99.8% of second-level administrative units improved during this time period.

We are also able to incorporate draw-level uncertainty to check whether an area has passed a mortality threshold with varying levels of confidence. In our analysis, when assessing whether a second-level administrative unit has met the SDG 3.2 target of less than 25 child deaths per 1,000 live births, we choose a confidence level of 90%. This means that we only consider a unit to have met the SDG 3.2 target if 90% of its draws fall below the threshold of 25 per 1,000. Using this criterion, we found that 32% of units had met the SDG 3.2 target for under-5 mortality in 2017. This differs from an analysis that

assesses only whether the mean estimate for U5M is below the SDG 3.2 target; if only the mean estimate is considered, then 49% of units have met the target in 2017.

6 Additional results

In this section we provide additional figures of interest that did not make it into the main manuscript or extended data figures.

Furthermore, a fully interactive visualization covering all results at the grid cell and administrative subdivisions discussed in this paper is available at: https://vizhub.healthdata.org/lbd/under5. Using the interactive tool, you may view annual estimates for neonatal, infant, and under-5 mortality rates and death counts at the grid-cell level, first administrative level, second administrative level, and country level. The tool includes mean and uncertainty intervals for all estimates at all levels.

Supplementary Figure 6.1: Second administrative subdivisions with a significant decline in neonatal mortality probability between 2000 and 2017

Significant is defined as non-overlapping 95% uncertainty intervals in 2000 and 2017.

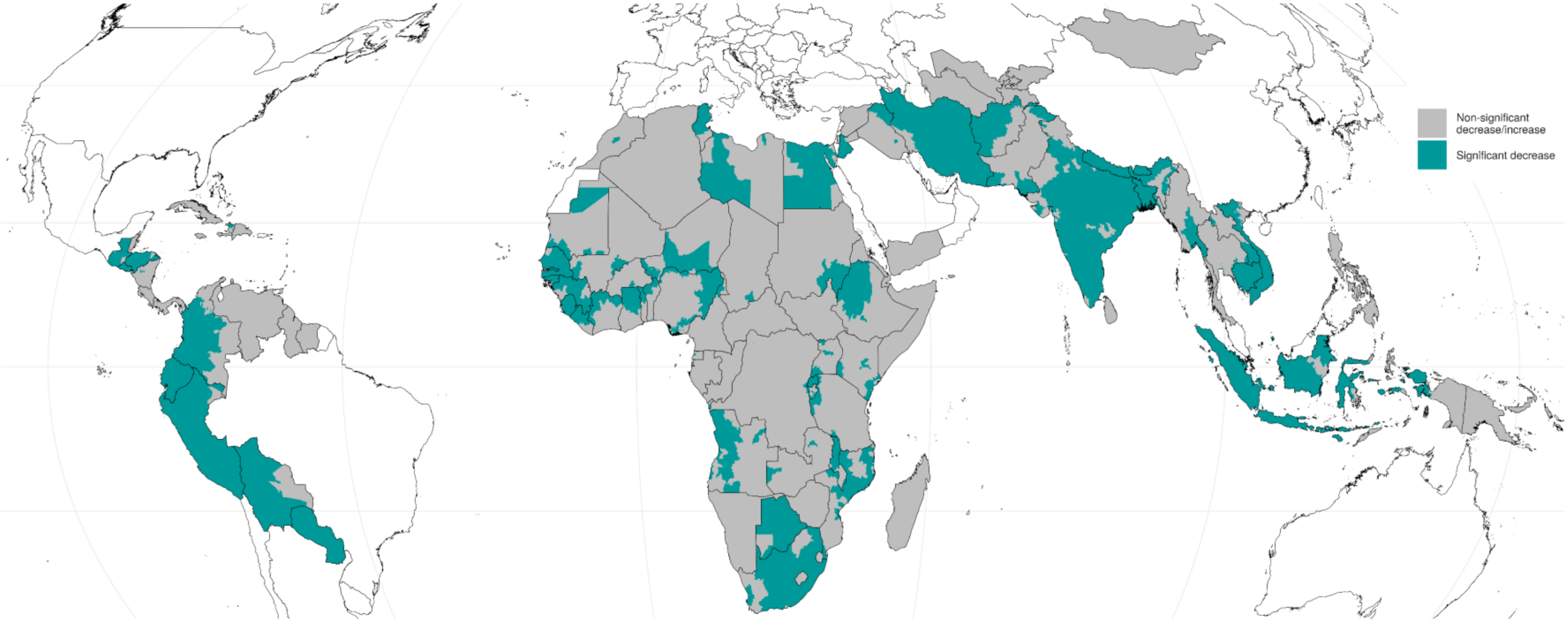

Supplementary Figure 6.2: Second administrative subdivisions with a significant decline in infant mortality probability between 2000 and 2017

Significant is defined as non-overlapping 95% uncertainty intervals in 2000 and 2017.

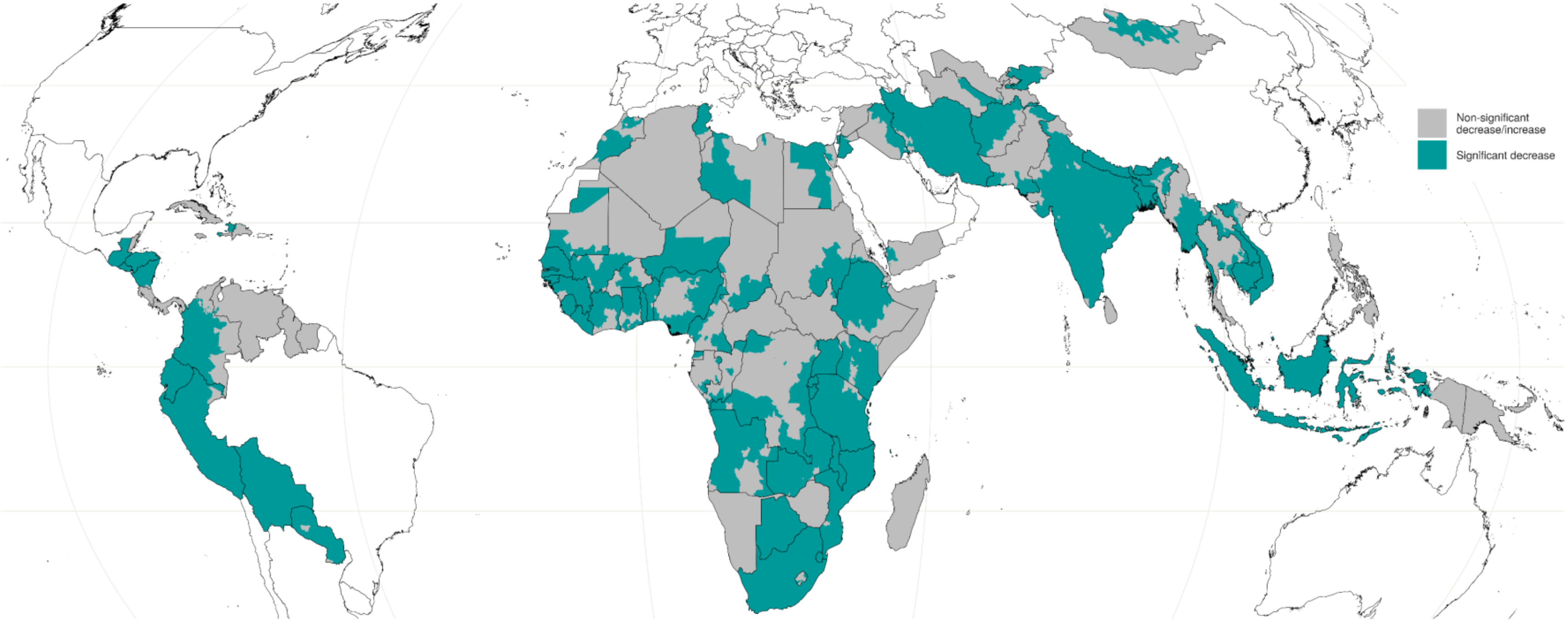

Supplementary Figure 6.3: Second administrative subdivisions with a significant decline in under-5 mortality probability between 2000 and 2017

Significant is defined as non-overlapping 95% uncertainty intervals in 2000 and 2017.

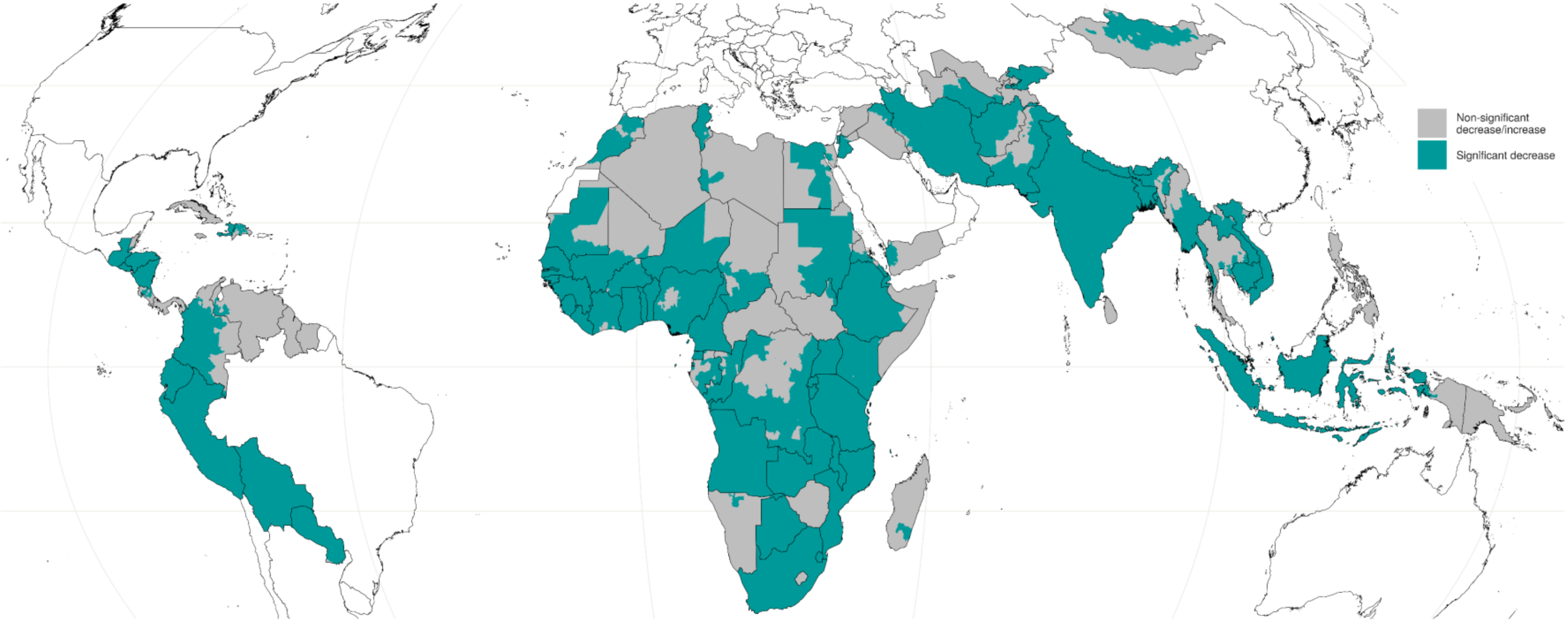

Supplementary Figure 6.4: Percentage of under-5 deaths in each bin of under-5 mortality probability that occur in the indicated world regions

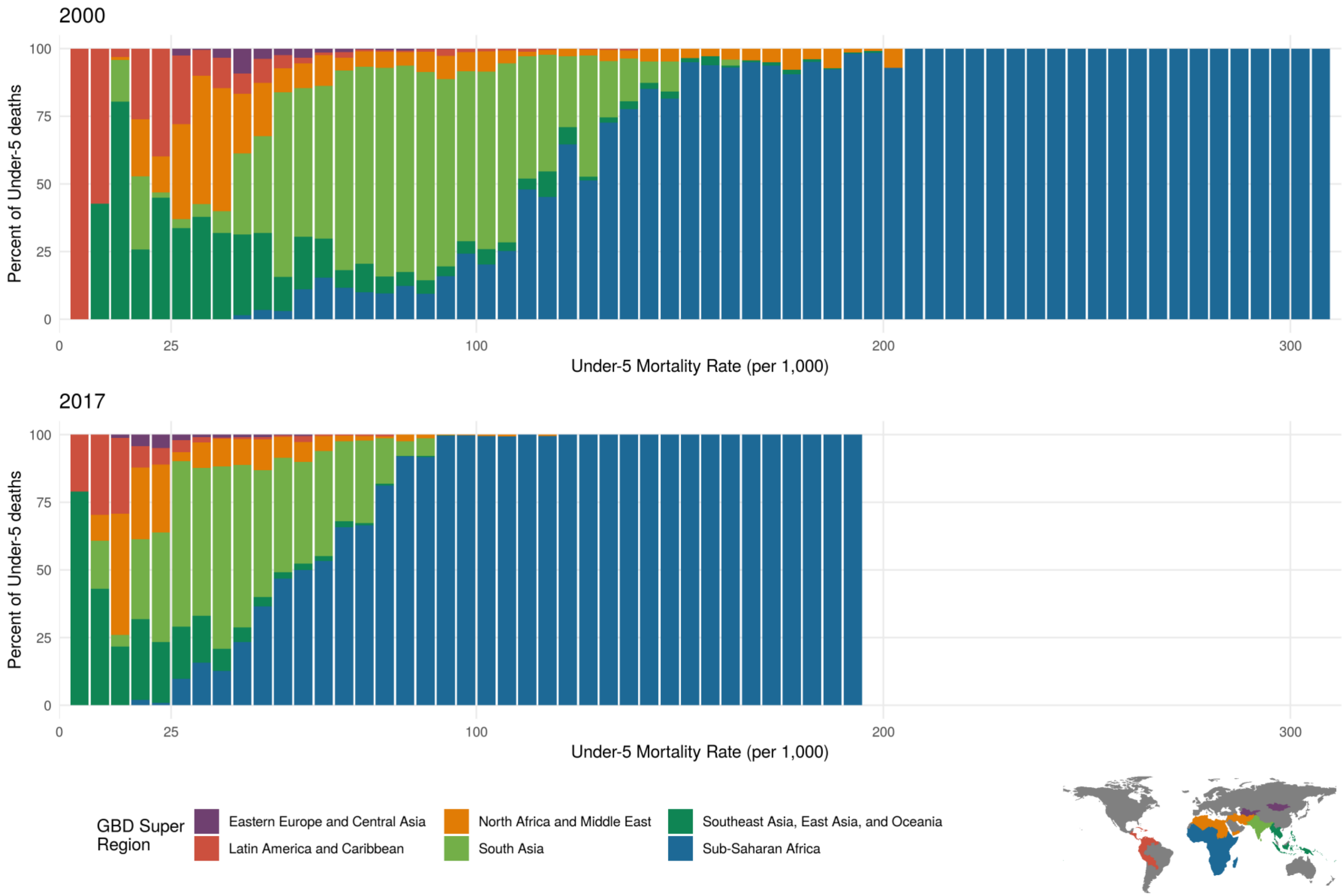

Supplementary Figure 6.5: Posterior probability of having met the SDG 3.2 target of 12 deaths per 1,000 live births for neonatal mortality probability in 2017

Note: mapped at the second administrative subdivision level.

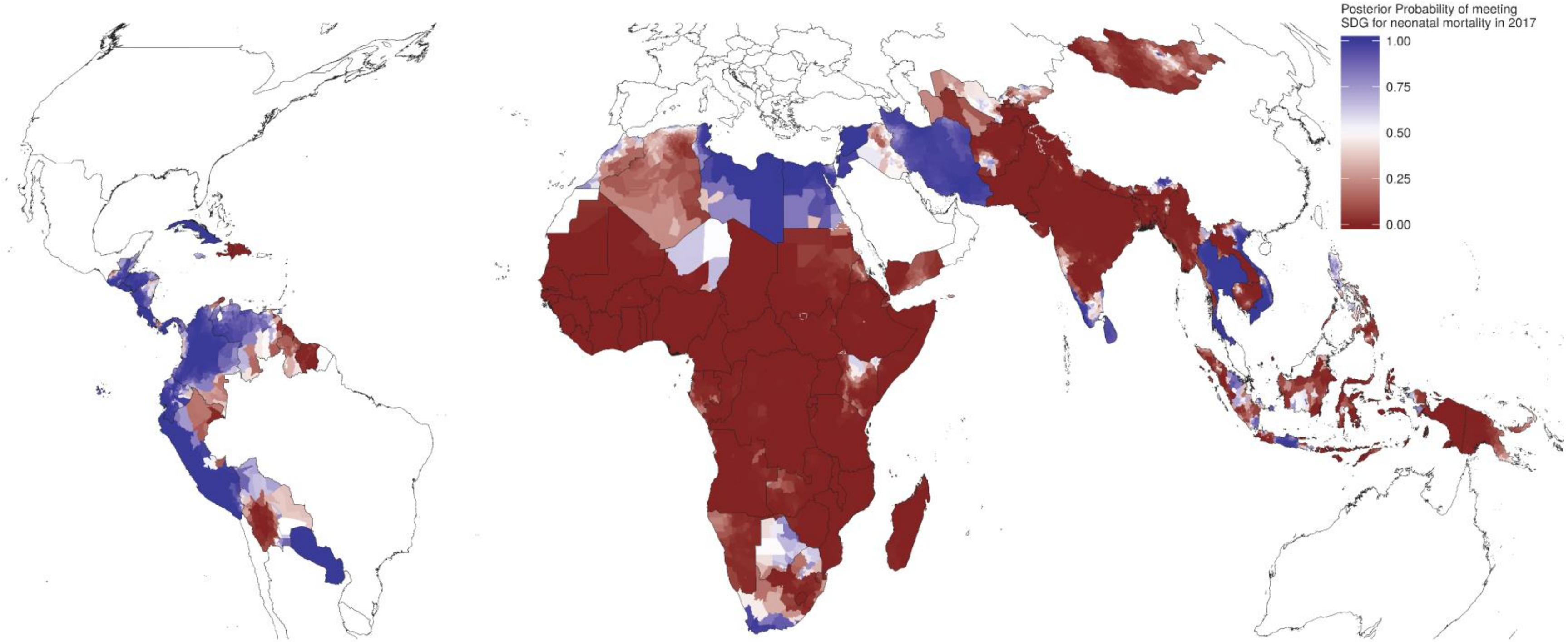

Supplementary Figure 6.6: Under-5 mortality probability by second administrative subdivision projected to 2030

Simple projections were used based on the rate of change in the estimates from 2000 to 2017.

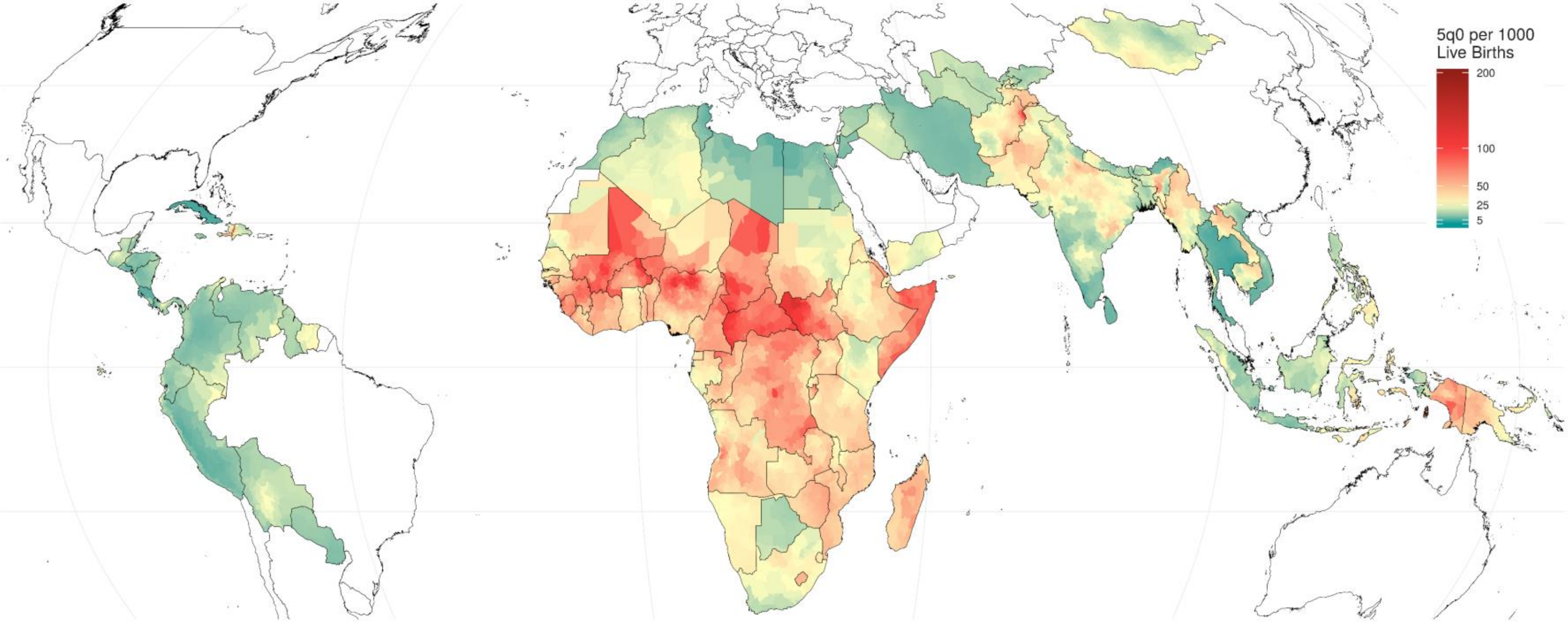

Supplementary Figure 6.7: Under-5 mortality probability by second administrative subdivision in 2000, normalized to the mean under-5 mortality probability within each country

The resulting map shows subnational deviation in under-5 mortality probability from national averages in the year 2000.

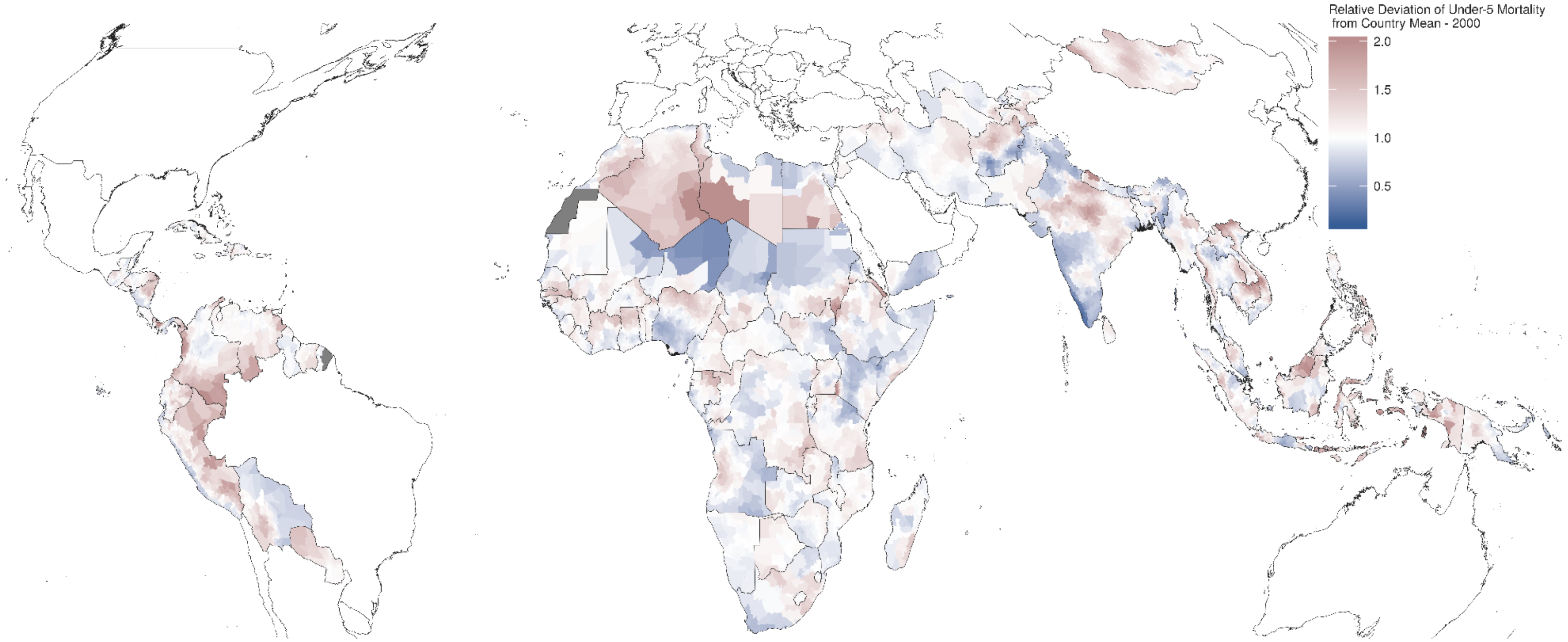

Supplementary Figure 6.8: Under-5 mortality probability by second administrative subdivision in 2017, normalized to the mean under-5 mortality probability within each country

The resulting map shows subnational deviation in under-5 mortality probability from national averages in the year 2017.

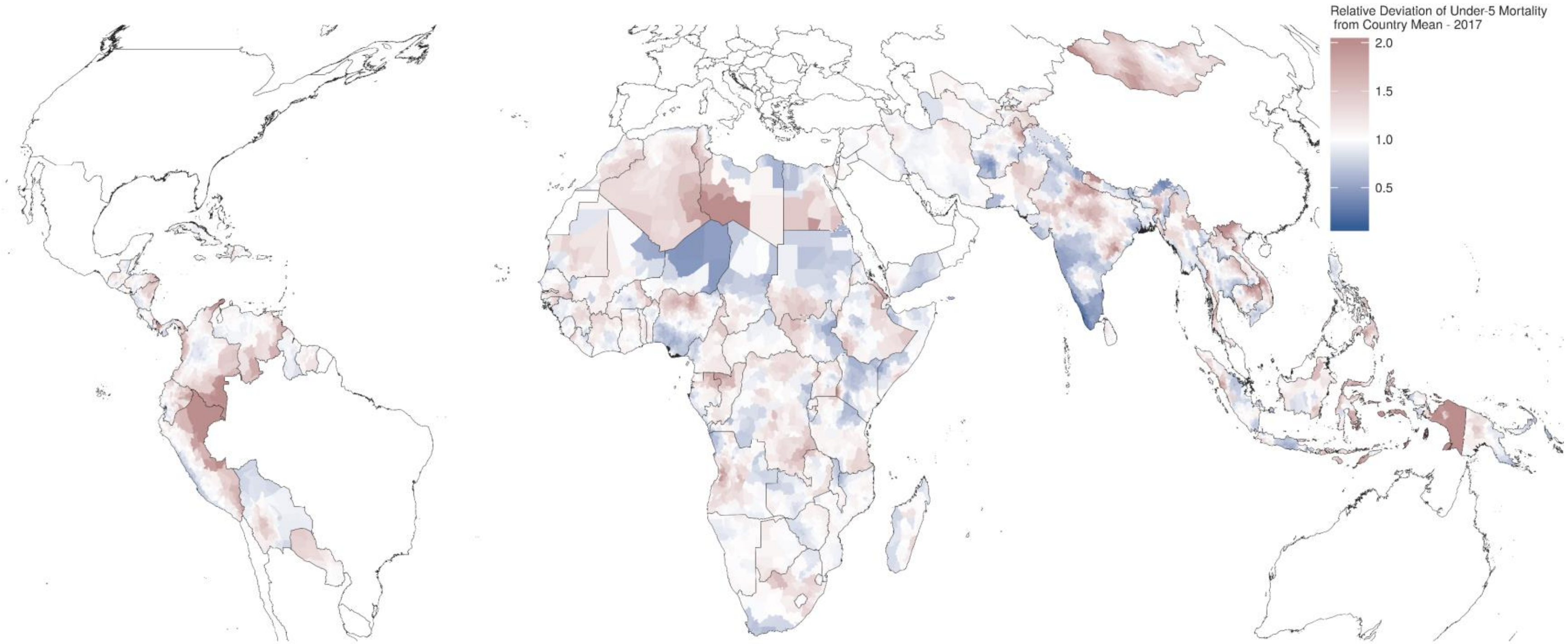

Supplementary Figure 6.9: Number of under-5 deaths, distributed across level of under-5 mortality rate in 2017 across 100 countries

The top figure represents total under-5 deaths in 2017 as distributed across bins of under-5 mortality rate. The color of each bar represents mutually exclusive age groups for neonatal (birth through 28 days), post-neonatal (28 days through one year), and child (1 year through 5 years). The bottom figure shows the same distribution such that each bar is normalized by the bar height to show the relative distribution in number of deaths across these age bins. At lower under-5 mortality rates, nearly 50% of child deaths occur in the first month of life, whereas in most areas with under-5 mortality rate greater than 100 deaths per 1,000 live births, around 30% of deaths occur in the first month of life, and nearly 50% occur in children 1 year and older.

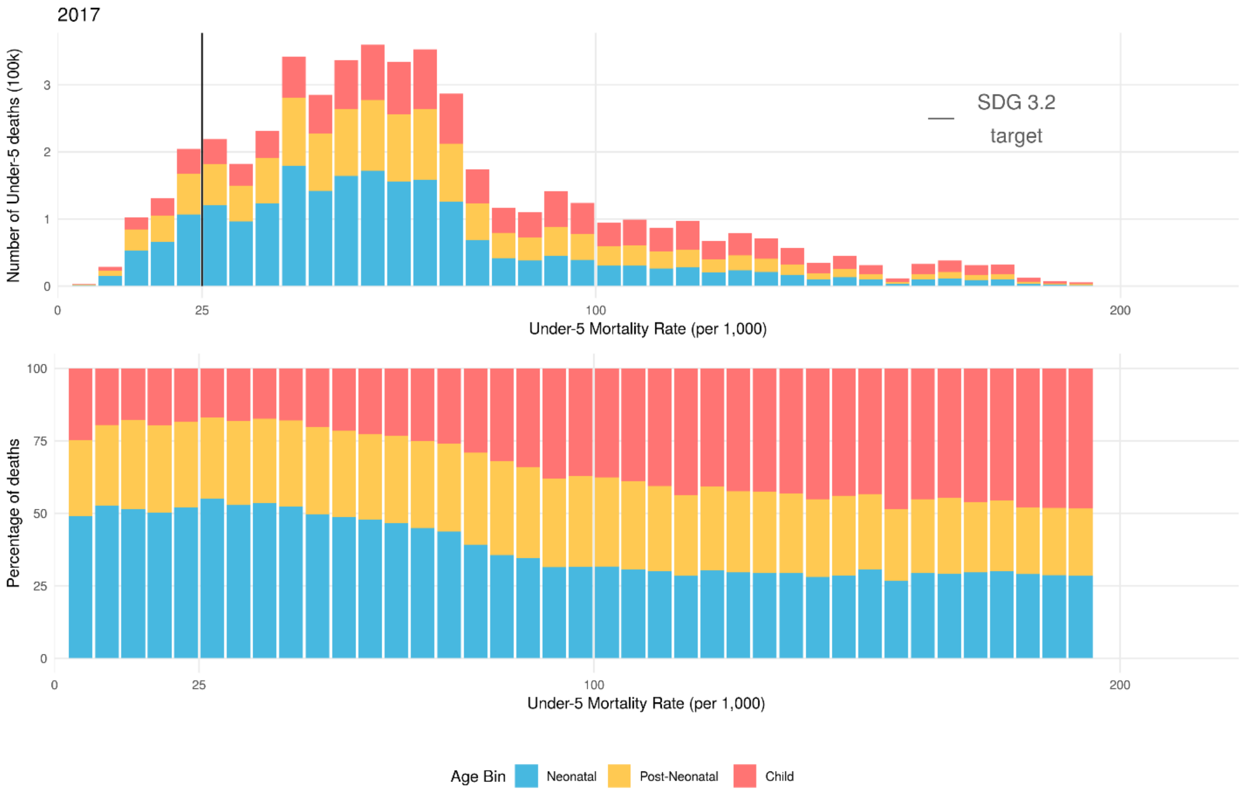

Supplementary Figure 6.10: Point data availability per pixel by modeling region, 2000–2017

Estimates of data availability were generated by counting the number of pixels in a modeling region with an overlapping data point in a given year, and dividing by the total number of pixels estimated in that modeling region and year after masking areas with very low population. The figure below shows data availability per pixel as it exists within the final modeling dataset; in this plot, observations at the polygon level have been resampled to points and are included in the estimate of data availability.

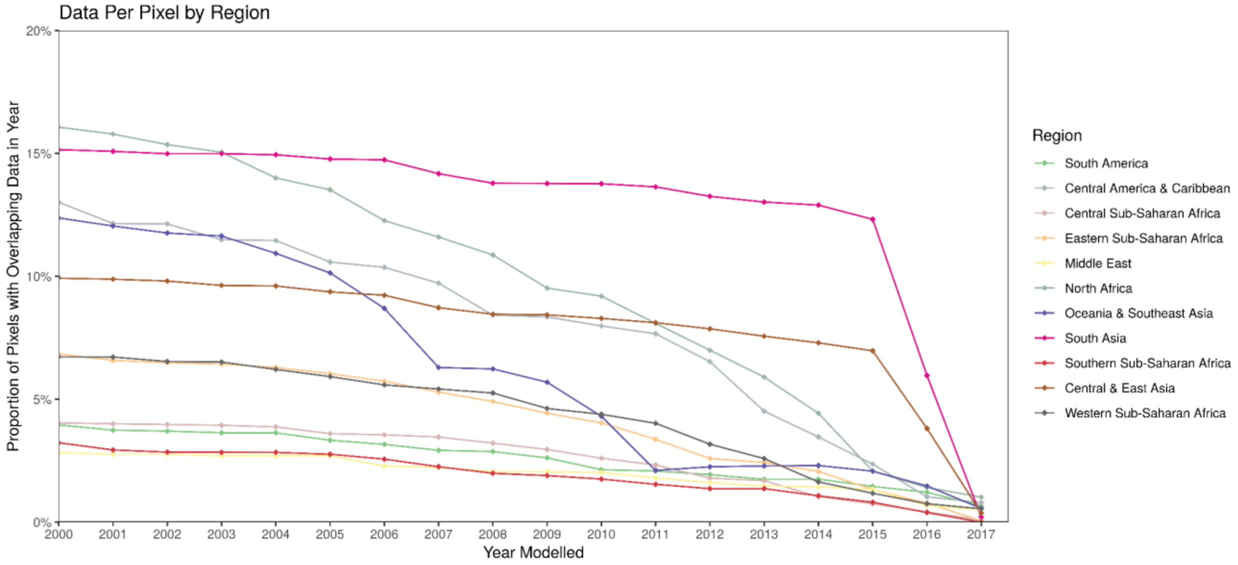

Supplementary Figure 6.11: Point data availability per pixel by modeling region, 2000–2017, after dropping observations

Estimates of data availability were generated by counting the number of pixels in a modeling region with an overlapping data point in a given year, and dividing by the total number of pixels estimated in that modeling region and year after masking areas with very low population. The figure below shows data availability per pixel after dropping all observations matched to areal (polygon) units such as administrative divisions, showing only data availability for true point observations.

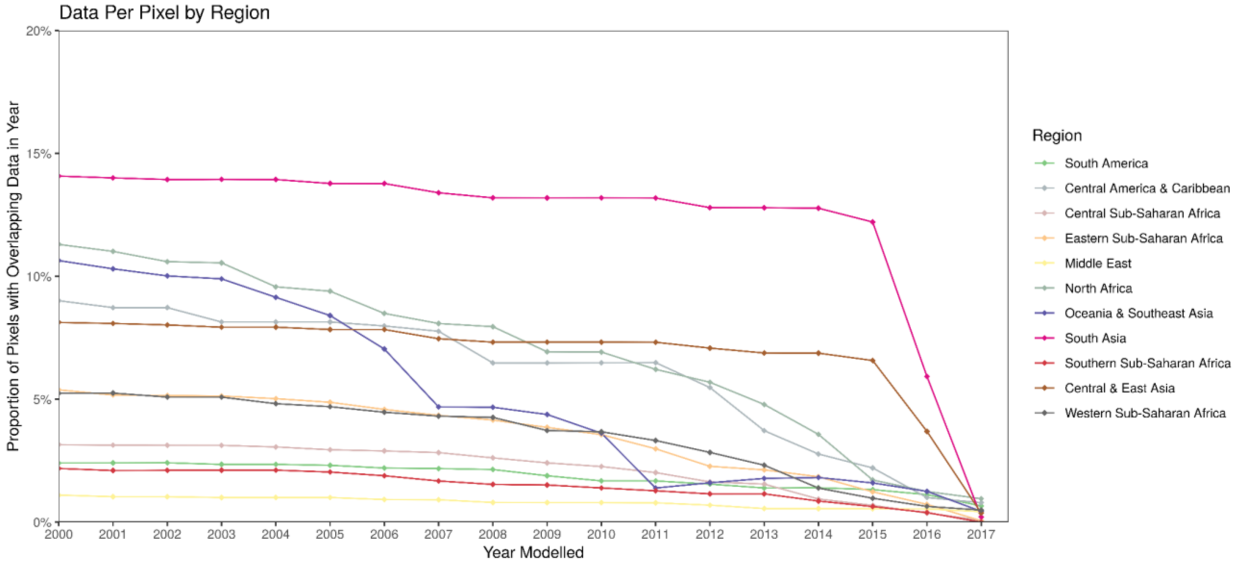

Supplementary Figure 6.12: Change in absolute and relative inequalities in under-5 mortality rate across second administrative level units between 2000 and 2017.

The first plot below looks at absolute inequalities (U5MR in a second administrative level unit minus the country mean U5MR). The second plot looks at relative inequalities (U5MR in a second administrative level unit divided by the country mean U5MR). A slope less than 45 degrees indicates declining inequality between 2000 and 2017, while a steeper slope indicates increasing inequality. In the absolute difference plot, we see an attenuated slope, indicating that absolute inequalities have on average declined since 2000. Since we would expect absolute inequality to decline as mortality rate declines, but would only expect relative inequalities to decline if certain policies/programs/processes were implicitly in place to do so (since its less sensitive to boundary effect), this indicates that reductions in absolute mortality inequality are more a function of the convergence toward a lower mortality, rather than a strictly egalitarian reduction of within-country inequality.

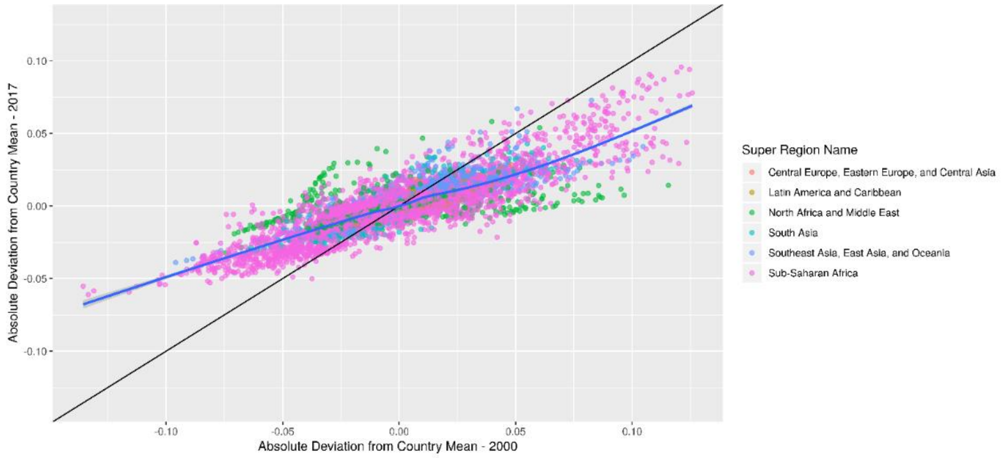

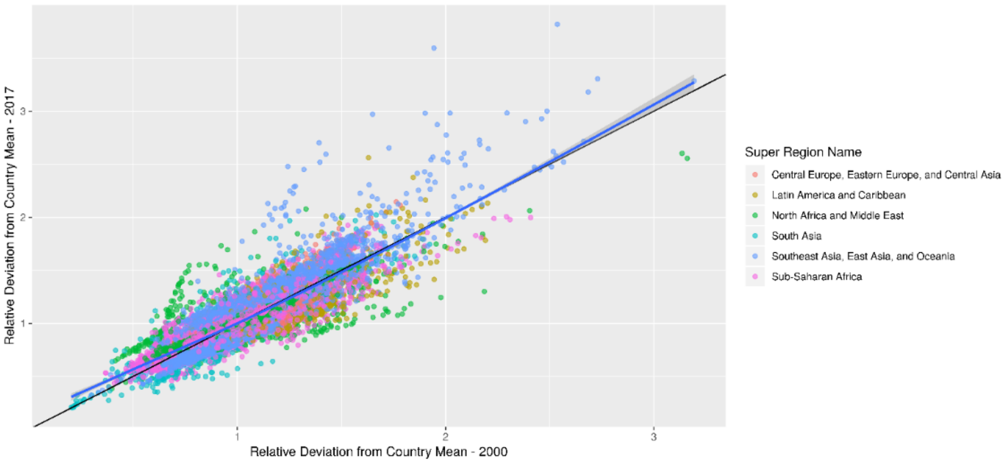

7 References

1. Golding, N. *et al.* Mapping under-5 and neonatal mortality in Africa, 2000–15: a baseline analysis for the Sustainable Development Goals. *The Lancet* **390**, 2171–2182 (2017).
2. Reiner, R. C. *et al.* Variation in Childhood Diarrheal Morbidity and Mortality in Africa, 2000–2015. *N. Engl. J. Med.* **379**, 1128–1138 (2018).
3. Osgood-Zimmerman, A. *et al.* Mapping child growth failure in Africa between 2000 and 2015. *Nature* **555**, 41–47 (2018).
4. Graetz, N. *et al.* Mapping local variation in educational attainment across Africa. *Nature* **555**, 48–53 (2018).
5. Burstein, R., Wang, H., Jr, R. C. R. & Hay, S. I. Development and validation of a new method for indirect estimation of neonatal, infant, and child mortality trends using summary birth histories. *PLOS Med.* **15**, e1002687 (2018).
6. Kristensen, K. *et al.* *TMB: Template Model Builder: A General Random Effect Tool Inspired by 'ADMB'*. (2018).
7. GBD 2017 Mortality Collaborators. Global, regional, and national age-sex-specific mortality and life expectancy, 1950–2017: a systematic analysis for the Global Burden of Disease Study 2017. *The Lancet* **392**, 1684–1735 (2018).
8. Ahmad, O. B., Lopez, A. D. & Inoue, M. The decline in child mortality: a reappraisal. *Bull. World Health Organ.* **78**, 1175–1191 (2000).
9. Somoza, J. L. Illustrative analysis of infant and child mortality in Colombia; draft for comments. (1980).
10. Infant and child mortality: levels, trends and demographic differentials. | POPLINE.org. Available at: https://www.popline.org/node/399179. (Accessed: 21st January 2019)

11. Preston, S., Heuveline, P. & Guillot, M. *Demography: Measuring and Modeling Population Processes*. (Wiley-Blackwell, 2000).
12. Karra, M., Fink, G. & Canning, D. Facility distance and child mortality: a multi-country study of health facility access, service utilization, and child health outcomes. *Int. J. Epidemiol.* **46**, 817–826 (2017).
13. Wang, L. Determinants of child mortality in LDCs: Empirical findings from demographic and health surveys. *Health Policy* **65**, 277–299 (2003).
14. Proville, J., Zavala-Araiza, D. & Wagner, G. Night-time lights: A global, long term look at links to socio-economic trends. *PLoS ONE* **12**, (2017).
15. Browne, A. W. & Barrett, H. R. Female Education in Sub-Saharan Africa: The Key to Development? *Comp. Educ.* **27**, 275–285 (1991).
16. Gakidou, E., Cowling, K., Lozano, R. & Murray, C. J. Increased educational attainment and its effect on child mortality in 175 countries between 1970 and 2009: a systematic analysis. *The Lancet* **376**, 959–974 (2010).
17. Heft-Neal, S., Burney, J., Bendavid, E. & Burke, M. Robust relationship between air quality and infant mortality in Africa. *Nature* **559**, 254–258 (2018).
18. Sah, R. K. The Effects of Child Mortality Changes on Fertility Choice and Parental Welfare. *J. Polit. Econ.* **99**, 582–606 (1991).
19. Coale, A. J. Demographic Transition. in *Social Economics* (eds. Eatwell, J., Milgate, M. & Newman, P.) 16–23 (Palgrave Macmillan UK, 1989). doi:10.1007/978-1-349-19806-1_4
20. Eckert, S. & Kohler, S. Urbanization and health in developing countries: a systematic review. *World Health Popul.* **15**, 7–20 (2014).
21. Akachi, Y., Steenland, M. & Fink, G. Associations between key intervention coverage and child mortality: an analysis of 241 sub-national regions of sub-Saharan Africa. *Int. J. Epidemiol.* (2017). doi:10.1093/ije/dyx262

22. Ehreth, J. The global value of vaccination. *Vaccine* **21**, 596–600 (2003).

23. Madhi, S. A., Levine, O. S., Hajjeh, R., Mansoor, O. D. & Cherian, T. Vaccines to prevent pneumonia and improve child survival. *Bull. World Health Organ.* **86**, 365–372 (2008).

24. Akachi, Y. & Atun, R. Effect of investment in malaria control on child mortality in sub-Saharan Africa in 2002-2008. *PloS One* **6**, e21309 (2011).

25. Eisele, T. P. *et al.* Estimates of child deaths prevented from malaria prevention scale-up in Africa 2001-2010. *Malar. J.* **11**, 93 (2012).

26. GBD 2017 Risk Factors Collaborators. Global, regional, and national comparative risk assessment of 84 behavioural, environmental and occupational, and metabolic risks or clusters of risks, 1990–2017: a systematic analysis for the Global Burden of Disease Study 2017. *The Lancet* (In Press).

27. Black, R. E. *et al.* Maternal and child undernutrition: global and regional exposures and health consequences. *The Lancet* **371**, 243–260 (2008).

28. Pelletier, D. L. & Frongillo, E. A. Changes in Child Survival Are Strongly Associated with Changes in Malnutrition in Developing Countries. *J. Nutr.* **133**, 107–119 (2003).

29. Lindgren, F., Rue, H. & Lindström, J. An explicit link between Gaussian fields and Gaussian Markov random fields: the stochastic partial differential equation approach. *R. Stat. Soc.* **73**, 423–498 (2011).

30. Patil, A. P., Gething, P. W., Piel, F. B. & Hay, S. I. Bayesian geostatistics in health cartography: the perspective of malaria. *Trends Parasitol.* **27**, 246–253 (2011).

31. Bell, M. *et al.* Internal Migration and Development: Comparing Migration Intensities Around the World. *Popul. Dev. Rev.* **41**, 33–58 (2015).

8 Data sources

Supplementary Table 8.1: Data sources included in analysis

Country	Year	Data Type	Children Born	Polygons	Points	Citation	GHDx ID
Afghanistan	2006	SBH	39724	29	0	Indian Institute of Health Management Research (IIHMR), Johns Hopkins University, Ministry of Public Health (Afghanistan). Afghanistan Health Survey 2006.	18468
Afghanistan	2010	CBH	113806	34	0	Central Statistics Organization (Afghanistan), ICF Macro, Indian Institute of Health Management Research (IIHMR), Ministry of Public Health (Afghanistan), World Health Organization Regional Office for the Eastern Mediterranean (EMRO-WHO). Afghanistan Special Demographic and Health Survey 2010. Fairfax, United States: ICF International.	56099
Afghanistan	2011	SBH	64660	34	0	Central Statistics Organization (Afghanistan), United Nations Children’s Fund (UNICEF). Afghanistan Multiple Indicator Cluster Survey 2010-2011. New York, United States: United Nations Children’s Fund (UNICEF), 2013.	56830
Afghanistan	2016	CBH	125715	310	0	Central Statistics Organization (Afghanistan), ICF International, Ministry of Public Health (Afghanistan). Afghanistan Demographic and Health Survey 2015-2016. Fairfax, United States: ICF International, 2017.	157018
Algeria	2002	CBH	29406	47	0	National Office of Statistics (Algeria), Ministry of Health, Population and Hospital Reform (Algeria), League of Arab States. Algeria Family Health Survey 2002-2003.	627
Algeria	2013	CBH	58390	7	0	Ministry of Health and Population (Algeria), United Nations Children’s Fund (UNICEF). Algeria Multiple Indicator Cluster Survey 2012-2013. New York, United States: United	210614

Country	Year	Data Type	Children Born	Polygons	Points	Citation	GHDx ID
						Nations Children's Fund (UNICEF), 2018.	
Angola	2001	SBH	23916	18	0	National Institute of Statistics (Angola), United Nations Children's Fund (UNICEF). Angola Multiple Indicator Cluster Survey 2001. New York, United States: United Nations Children's Fund (UNICEF).	687
Angola	2007	CBH	2932	0	115	COSEP-Consulting Ltd., Consaude Ltd., Macro International, Inc, Ministry of Health (Angola). Angola Malaria Indicator Survey 2006-2007. Fairfax, United States: ICF International.	672
Angola	2009	SBH	41890	18	0	National Institute of Statistics (Angola), Oxford Policy Management, United Nations Children's Fund (UNICEF). Angola Integrated Inquiry into People's Well-Being 2008-2009.	30394
Angola	2011	CBH	22925	0	230	COSEP-Consulting Ltd., Consaude Ltd., ICF International, National Malaria Control (Angola), President's Malaria Initiative (PMI). Angola Malaria Indicator Survey 2011. Fairfax, United States: ICF International.	56169
Angola	2016	CBH	42002	18	625	ICF International, Ministry of Health (Angola), National Institute of Statistics (Angola), United Nations Children's Fund (UNICEF). Angola Demographic and Health Survey 2015-2016. Fairfax, United States: ICF International, 2017.	218555
Bangladesh	2000	CBH	31925	0	341	Macro Systems, Inc, Mitra and Associates, National Institute of Population Research and Training (NIPORT). Bangladesh Demographic and Health Survey 1999-2000. Fairfax, United States: ICF International.	26826

Country	Year	Data Type	Children Born	Polygons	Points	Citation	GHDx ID
Bangladesh	2001	CBH	319622	64	0	Associates for Community and Population Research (ACPR), International Centre for Diarrhoeal Disease Research, Bangladesh (ICDDR,B), Johns Hopkins University, Mitra and Associates, National Institute of Population Research and Training (NIPORT), ORC Macro. Bangladesh Special Demographic and Health Survey 2001. Fairfax, United States: ICF International.	18920
Bangladesh	2004	CBH	33605	0	359	Mitra and Associates, ORC Macro. Bangladesh Demographic and Health Survey 2004. Fairfax, United States: ICF International.	18902
Bangladesh	2007	CBH	30527	0	361	Macro International, Inc, Mitra and Associates, National Institute of Population Research and Training (NIPORT). Bangladesh Demographic and Health Survey 2007. Fairfax, United States: ICF International, 2009.	18913
Bangladesh	2009	SBH	705724	6	0	Bangladesh Bureau of Statistics (BBS). Bangladesh Multiple Indicator Cluster Survey 2009. Dhaka, Bangladesh: Bangladesh Bureau of Statistics (BBS).	126906
Bangladesh	2012	CBH	45844	0	600	ICF Macro, Mitra and Associates, National Institute of Population Research and Training (NIPORT). Bangladesh Demographic and Health Survey 2011-2012. Calverton, United States: ICF Macro.	55956
Bangladesh	2013	SBH	112041	0	2628	Bangladesh Bureau of Statistics (BBS), Government of Bangladesh, Ministry of Planning (Bangladesh), United Nations Children's Fund (UNICEF). Bangladesh Multiple Indicator Cluster Survey 2012-2013. New York, United States: United Nations Children's Fund (UNICEF), 2015.	151086

Country	Year	Data Type	Children Born	Polygons	Points	Citation	GHDx ID
Bangladesh	2014	CBH	43772	7	0	ICF International, Mitra and Associates, National Institute of Population Research and Training (NIPORT). Bangladesh Demographic and Health Survey 2014. Fairfax, United States: ICF International, 2015.	157021
Belize	2006	SBH	4206	6	0	Statistical Institute of Belize, United Nations Children's Fund (UNICEF). Belize Multiple Indicator Cluster Survey 2006. New York, United States: United Nations Children's Fund (UNICEF).	1089
Belize	2011	SBH	8888	7	0	Statistical Institute of Belize, United Nations Children's Fund (UNICEF). Belize Multiple Indicator Cluster Survey 2011. New York, United States: United Nations Children's Fund (UNICEF), 2013.	76699
Belize	2015	CBH	19904	6	0	Government of Belize, Statistical Institute of Belize, UN Resident Coordinator Fund (UN ResCor), United Nations Children's Fund (UNICEF), United Nations Development Programme (UNDP). Belize Multiple Indicator Cluster Survey 2015-2016. New York, United States: United Nations Children's Fund (UNICEF), 2018.	264910
Benin	2001	CBH	19398	0	247	National Institute of Statistics and Economic Analysis (INSAE) (Benin), ORC Macro. Benin Demographic and Health Survey 2001. Fairfax, United States: ICF International.	18950
Benin	2002	SBH	440265	76	0	National Institute of Statistics and Economic Analysis (INSAE) (Benin), Minnesota Population Center. Benin Population and Housing Census 2002 from the Integrated Public Use Microdata Series, International. Minneapolis, MN: IPUMS, 2018. https://doi.org/10.18128/D020.V7.1	367347

Country	Year	Data Type	Children Born	Polygons	Points	Citation	GHDx ID
Benin	2006	CBH	57232	12	0	Macro International, Inc, National Institute of Statistics and Economic Analysis (INSAE) (Benin), National Program Against AIDS (PNLS) (Benin). Benin Demographic and Health Survey 2006. Fairfax, United States: ICF International.	18959
Benin	2012	CBH	47152	0	746	ICF International, National Institute of Statistics and Economic Analysis (INSAE) (Benin), National Program Against AIDS (PNLS) (Benin). Benin Demographic and Health Survey 2011-2012. Fairfax, United States: ICF International, 2014.	79839
Benin	2014	CBH	45183	12	0	National Institute of Statistics and Economic Analysis (INSAE) (Benin), United Nations Children's Fund (UNICEF). Benin Multiple Indicator Cluster Survey 2014. New York, United States: United Nations Children's Fund (UNICEF), 2017.	206075
Bhutan	2010	SBH	31697	20	0	National Statistics Bureau (Bhutan), United Nations Children's Fund (UNICEF), United Nations Population Fund (UNFPA). Bhutan Multiple Indicator Cluster Survey 2010. New York, United States: United Nations Children's Fund (UNICEF).	40028
Bhutan	2013	SBH	58980	19	0	Ministry of Health (Bhutan), National Statistics Bureau (Bhutan), United Nations Population Fund (UNFPA). Bhutan Health Survey 2012-2013.	165290[†]
Bhutan	2017	SBH	307521	20	0	National Statistics Bureau (Bhutan). Bhutan Population and Housing Census 2017.	325119[†]

Country	Year	Data Type	Children Born	Polygons	Points	Citation	GHDx ID
Bolivia	2001	SBH	486216	83	0	National Institute of Statistics (Bolivia), Minnesota Population Center. Bolivia National Census of Population and Housing 2001 from the Integrated Public Use Microdata Series, International: [Machine-readable database]. Minneapolis: University of Minnesota.	1362
Bolivia	2004	CBH	45116	8	0	Macro International, Inc, Ministry of Health and Sports (Bolivia), National Institute of Statistics (Bolivia). Bolivia Demographic and Health Survey 2003-2004. Fairfax, United States: ICF International.	19001
Bolivia	2006	SBH	9130	9	0	National Institute of Statistics (Bolivia). Bolivia Household Survey 2006. La Paz, Bolivia: National Institute of Statistics (Bolivia).	148343
Bolivia	2007	SBH	9062	9	0	National Institute of Statistics (Bolivia). Bolivia Household Survey 2007. La Paz, Bolivia: National Institute of Statistics (Bolivia).	148344
Bolivia	2008	CBH	40355	0	998	Macro International, Inc, Ministry of Health and Sports (Bolivia), National Institute of Statistics (Bolivia). Bolivia Demographic and Health Survey 2008. Fairfax, United States: ICF International.	19016
Bolivia	2008	SBH	7851	9	0	National Institute of Statistics (Bolivia). Bolivia Household Survey 2008. La Paz, Bolivia: National Institute of Statistics (Bolivia).	148345
Bolivia	2009	SBH	8316	9	0	National Institute of Statistics (Bolivia). Bolivia Household Survey 2009. La Paz, Bolivia: National Institute of Statistics (Bolivia).	148346
Bolivia	2011	SBH	17415	9	0	National Institute of Statistics (Bolivia). Bolivia Household Survey 2011. La Paz, Bolivia: National Institute of Statistics (Bolivia).	164634

Country	Year	Data Type	Children Born	Polygons	Points	Citation	GHDx ID
Bolivia	2013	SBH	16035	9	0	National Institute of Statistics (Bolivia). Bolivia Household Survey 2013. La Paz, Bolivia: National Institute of Statistics (Bolivia).	164635
Bolivia	2014	SBH	17204	9	0	National Institute of Statistics (Bolivia). Bolivia Household Survey 2014. La Paz, Bolivia: National Institute of Statistics (Bolivia).	283486
Bolivia	2015	SBH	17208	9	0	National Institute of Statistics (Bolivia), United Nations Economic Commission for Latin America and the Caribbean (ECLAC). Bolivia Household Survey 2015. La Paz, Bolivia: National Institute of Statistics (Bolivia), 2015.	317285
Bolivia	2016	CBH	24072	9	0	Ministry of Health (Bolivia), National Institute of Statistics (Bolivia). Bolivia Demographic and Health Survey 2016. La Paz, Bolivia: National Institute of Statistics (Bolivia), 2017.	323944
Bolivia	2016	SBH	17632	9	0	National Institute of Statistics (Bolivia), United Nations Economic Commission for Latin America and the Caribbean (ECLAC). Bolivia Household Survey 2016.	336686
Botswana	2001	SBH	92210	22	0	Central Statistics Office (Botswana), Minnesota Population Center. Botswana Population and Housing Census 2001 from the Integrated Public Use Microdata Series, International. Minneapolis: University of Minnesota, 2017.	294205
Botswana	2006	SBH	22725	25	0	Central Statistics Office (Botswana). Botswana Demographic Survey 2006. Gaborone, Botswana: Central Statistics Office (Botswana).	21970
Botswana	2008	CBH	10858	330	0	Central Statistics Office (Botswana). Botswana Family Health Survey 2007-2008. Gaborone, Botswana: Central Statistics Office (Botswana), 2009.	22125

Country	Year	Data Type	Children Born	Polygons	Points	Citation	GHDx ID
Botswana	2011	SBH	95455	22	0	Central Statistics Office (Botswana), Minnesota Population Center. Botswana Population and Housing Census 2011 from the Integrated Public Use Microdata Series, International. Minneapolis: University of Minnesota, 2017.	294235
Burkina Faso	2003	CBH	41520	0	397	Macro International, Inc, National Institute of Statistics and Demography (Burkina Faso). Burkina Faso Demographic and Health Survey 2003. Fairfax, United States: ICF International.	19088
Burkina Faso	2006	SBH	25541	0	195	National Institute of Statistics and Demography (Burkina Faso), United Nations Children’s Fund (UNICEF). Burkina Faso Multiple Indicator Cluster Survey 2006. New York, United States: United Nations Children’s Fund (UNICEF).	1927
Burkina Faso	2006	SBH	946930	236	0	Minnesota Population Center, National Institute of Statistics and Demography (Burkina Faso). Burkina Faso Population and Housing Census 2006 from the Integrated Public Use Microdata Series, International: [Machine-readable database]. Minneapolis: University of Minnesota, 2013.	105403
Burkina Faso	2010	SBH	5816	2	0	Institut de Recherche pour le Developpement (IRD); Institut de Recherche en Sciences de la Sante (IRSS); and HarvestPlus, International Food Policy Research Institute (IFPRI). 2016. Food consumption and iron status survey in two provinces of rural Burkina Faso. Washington, DC: International Food Policy Research Institute (IFPRI). http://dx.doi.org/10.7910/DVN/5CXCLX	283273

Country	Year	Data Type	Children Born	Polygons	Points	Citation	GHDx ID
Burkina Faso	2011	CBH	56178	0	541	ICF Macro, Ministry of Health (Burkina Faso), National Institute of Statistics and Demography (Burkina Faso). Burkina Faso Demographic and Health Survey 2010-2011. Fairfax, United States: ICF International.	19133
Burkina Faso	2014	SBH	27986	0	248	ICF International, National Institute of Statistics and Demography (Burkina Faso), National Program for the Fight Against Malaria (PNLP) (Burkina Faso). Burkina Faso Malaria Indicator Survey 2014. Fairfax, United States: ICF International, 2015.	188785
Burundi	2005	SBH	25768	554	0	United Nations Children's Fund (UNICEF), Burundi Institute of Statistics and Economic Studies, United Nations Population Fund (UNFPA). Burundi Multiple Indicator Cluster Survey 2005. New York, United States: United Nations Children's Fund (UNICEF).	1981
Burundi	2011	CBH	24520	0	376	Burundi Institute of Statistics and Economic Studies, ICF International, Ministry of Public Health and the Fight Against AIDS (Burundi). Burundi Demographic and Health Survey 2010-2011. Fairfax, United States: ICF International, 2012.	30431
Burundi	2013	SBH	13647	0	200	Burundi Institute of Statistics and Economic Studies, ICF Macro, Ministry of Public Health and the Fight Against AIDS (Burundi), National Institute of Public Health (Burundi). Burundi Malaria Indicator Survey 2012-2013. Fairfax, United States: ICF International, 2013.	108080
Burundi	2016	CBH	45201	0	552	Burundi Institute of Statistics and Economic Studies, ICF International, Ministry of Public Health and the Fight Against AIDS (Burundi). Burundi Demographic and Health Survey 2016-2017. Fairfax, United States: ICF International, 2018.	286766

Country	Year	Data Type	Children Born	Polygons	Points	Citation	GHDx ID
Cambodia	2000	CBH	40990	0	470	Macro International, Inc, Ministry of Health (Cambodia), National Institute of Statistics (Cambodia). Cambodia Demographic and Health Survey 2000. Fairfax, United States: ICF International.	19156
Cambodia	2004	SBH	58962	24	0	National Institute of Statistics (Cambodia). Cambodia Intercensal Population Survey 2004.	2002
Cambodia	2006	CBH	40457	0	548	Macro International, Inc, National Institute of Public Health (Cambodia), National Institute of Statistics (Cambodia). Cambodia Demographic and Health Survey 2005-2006. Fairfax, United States: ICF International.	19167
Cambodia	2008	SBH	663330	173	0	National Institute of Statistics (Cambodia), Minnesota Population Center. Cambodia General Population Census 2008 from the Integrated Public Use Microdata Series, International: [Machine-readable database]. Minneapolis: University of Minnesota, 2011.	35329
Cambodia	2011	CBH	37511	0	607	ICF Macro, Ministry of Health (Cambodia), National Institute of Statistics (Cambodia). Cambodia Demographic and Health Survey 2010-2011. Fairfax, United States: ICF International.	30379
Cambodia	2013	SBH	62285	24	0	National Institute of Statistics (Cambodia), United Nations Population Fund (UNFPA). Cambodia Intercensal Population Survey 2013. Phnom Penh, Cambodia: National Institute of Statistics (Cambodia).	164729
Cambodia	2014	CBH	33290	0	611	ICF International, Ministry of Health (Cambodia), National Institute of Statistics (Cambodia). Cambodia Demographic and Health Survey 2014. Fairfax, United States: ICF International, 2017.	157024

Country	Year	Data Type	Children Born	Polygons	Points	Citation	GHDx ID
Cameroon	2004	CBH	29455	0	463	Macro International, Inc, National Institute of Statistics (Cameroon). Cameroon Demographic and Health Survey 2004. Fairfax, United States: ICF International.	19211
Cameroon	2005	SBH	1046500	209	0	Minnesota Population Center, National Institute of Statistics (Cameroon), Central Bureau of the Census and Population Studies (Cameroon). Cameroon Population and Housing Census 2005 from the Integrated Public Use Microdata Series, International: [Machine-readable database]. Minneapolis: University of Minnesota, 2013	105800
Cameroon	2011	CBH	42312	0	577	ICF International, Ministry of Economy, Planning and Regional Development (Cameroon), Ministry of Public Health (Cameroon), National Institute of Statistics (Cameroon), Pasteur Center of Cameroon. Cameroon Demographic and Health Survey 2011. Fairfax, United States: ICF International.	19274
Cameroon	2014	CBH	26201	12	0	Ministry of Public Health (Cameroon), National Institute of Statistics (Cameroon), United Nations Children's Fund (UNICEF). Cameroon Multiple Indicator Cluster Survey 2014. New York, United States: United Nations Children's Fund (UNICEF), 2017.	244455
Central African Republic	2006	SBH	35974	16	0	United Nations Children's Fund (UNICEF). Central African Republic Multiple Indicator Cluster Survey 2006. New York, United States: United Nations Children's Fund (UNICEF).	2223
Central African Republic	2011	SBH	37331	17	0	Central African Institute of Statistics, Economic and Social Studies (ICASEES) (Central African Republic), ICF International. Central African Republic Multiple Indicator Cluster Survey 2010-2011. Fairfax, United States: ICF International, 2013.	82832

Country	Year	Data Type	Children Born	Polygons	Points	Citation	GHDx ID
Chad	2004	CBH	21448	9	0	Macro International, Inc, National Institute of Statistical, Economic and Demographic Studies (Chad). Chad Demographic and Health Survey 2004. Fairfax, United States: ICF International.	19315
Chad	2010	SBH	61128	60	0	Ministry of Planning, Economy, and International Cooperation (Chad), National Institute of Statistical, Economic and Demographic Studies (Chad), United Nations Children's Fund (UNICEF). Chad Multiple Indicator Cluster Survey 2010. New York, United States: United Nations Children's Fund (UNICEF), 2014.	76701
Chad	2015	CBH	68989	0	624	ICF International, National Institute of Statistical, Economic and Demographic Studies (Chad). Chad Demographic and Health Survey 2014-2015. Fairfax, United States: ICF International, 2016.	157025
Colombia	2000	CBH	21267	23	0	Macro International, Inc, Profamilia (Colombia). Colombia Demographic and Health Survey 2000. Fairfax, United States: ICF International, 2000.	19359
Colombia	2005	CBH	71278	33	0	Macro International, Inc, Profamilia (Colombia). Colombia Demographic and Health Survey 2004-2005. Fairfax, United States: ICF International, 2005.	19324
Colombia	2006	SBH	1812545	489	0	National Administrative Department of Statistics (DANE) (Colombia), Minnesota Population Center. Colombia General Census 2005-2006 from the Integrated Public Use Microdata Series, International: [Machine-readable database]. Minneapolis: University of Minnesota.	3029
Colombia	2010	CBH	55239	21	0	ICF Macro, Profamilia (Colombia). Colombia Demographic and Health Survey 2009-2010. Fairfax, United States: ICF International, 2011.	21281

Country	Year	Data Type	Children Born	Polygons	Points	Citation	GHDx ID
Colombia	2016	CBH	62593	33	0	ICF International, Ministry of Health (Colombia), Profamilia (Colombia). Colombia Demographic and Health Survey 2015-2016. Fairfax, United States: ICF International, 2017.	218566
Comoros	2013	CBH	11497	0	242	General Directorate of Statistics and Forecasting (Comoros), ICF International. Comoros Demographic and Health Survey 2012-2013. Fairfax, United States: ICF International.	76850
Costa Rica	2011	SBH	175689	64	0	Minnesota Population Center, Costa Rica National Institute of Statistics and Census. Costa Rica Census 2011 from the Integrated Public Use Microdata Series, International: [Machine-readable database]. Minneapolis: University of Minnesota, 2015.	227111
Cote d'Ivoire	2005	CBH	13358	11	0	CDC Retro-CI, Ministry of the Fight Against AIDS (Cote d'Ivoire), National Institute of Statistics (Cote d'Ivoire), ORC Macro. Cote d'Ivoire AIDS Indicator Survey 2005. Fairfax, United States: ICF International.	56148
Cote d'Ivoire	2012	CBH	28211	0	341	ICF International, Ministry of the Fight Against AIDS (Cote d'Ivoire), National Institute of Statistics (Cote d'Ivoire). Cote d'Ivoire Demographic and Health Survey 2011-2012. Fairfax, United States: ICF International.	18533
Cote d'Ivoire	2016	CBH	32126	11	0	National Institute of Statistics (Cote d'Ivoire), United Nations Children's Fund (UNICEF). Cote d'Ivoire Multiple Indicator Cluster Survey 2016. New York, United States: United Nations Children's Fund (UNICEF), 2018.	218611
Cuba	2011	SBH	13154	15	0	Ministry of Public Health (Cuba), United Nations Children's Fund (UNICEF). Cuba Multiple Indicator Cluster Survey 2010-2011. New York, United States: United Nations Children's Fund (UNICEF).	60935

Country	Year	Data Type	Children Born	Polygons	Points	Citation	GHDx ID
Cuba	2014	SBH	12553	4	0	Ministry of Public Health (Cuba), National Office of Statistics (Cuba), United Nations Children's Fund (UNICEF). Cuba Multiple Indicator Cluster Survey 2014. New York, United States: United Nations Children's Fund (UNICEF), 2005.	169975
Democratic Republic of the Congo	2001	SBH	39356	11	0	Ministry of Planning and Reconstruction (Congo, DR), United Nations Children's Fund (UNICEF). Congo, DR Multiple Indicator Cluster Survey 2001. New York, United States: United Nations Children's Fund (UNICEF).	3161
Democratic Republic of the Congo	2007	CBH	29548	0	293	Macro International, Inc, Ministry of Planning (Congo, DR). Democratic Republic of the Congo Demographic and Health Survey 2007. Fairfax, United States: ICF International.	19381
Democratic Republic of the Congo	2010	SBH	40648	10	358	National Statistical Institute (Congo, DR), Ministry of Planning (Congo, DR), United Nations Children's Fund (UNICEF). Congo, DR Multiple Indicator Cluster Survey 2010. New York, United States: United Nations Children's Fund (UNICEF).	26998
Democratic Republic of the Congo	2013	CBH	59276	0	492	ICF International, Ministry of Planning and Monitoring Implementation of the Revolution of Modernity (Congo, DR), Ministry of Public Health (Congo, DR), National Institute of Statistics (Congo, DR). Democratic Republic of the Congo Demographic and Health Survey 2013-2014. Fairfax, United States: ICF International, 2014.	76878
Djibouti	2002	CBH	10574	1	0	Department of Statistics and Demographic Studies (Djibouti), League of Arab States, Ministry of Health (Djibouti), Pan Arab Project for Family Health (PAPFAM). Djibouti Family Health Survey 2002.	3392

Country	Year	Data Type	Children Born	Polygons	Points	Citation	GHDx ID
Djibouti	2006	SBH	10479	1	35	Ministry of Economy, Finance, and Planning in charge of Privatization (Djibouti), Ministry of Health (Djibouti), United Nations Children's Fund (UNICEF). Djibouti Multiple Indicator Cluster Survey 2006. New York, United States: United Nations Children's Fund (UNICEF).	3404
Dominican Republic	2002	CBH	53667	32	0	Center for Social and Demographic Studies (Dominican Republic) (CESDEM), Macro International, Inc. Dominican Republic Demographic and Health Survey 2002. Fairfax, United States: ICF International.	19444
Dominican Republic	2002	SBH	415435	100	1	National Statistics Office (Dominican Republic), Minnesota Population Center. Dominican Republic Census 2002 from the Integrated Public Use Microdata Series, International: [Machine-readable database]. Minneapolis: University of Minnesota.	151296
Dominican Republic	2006	CBH	18903	32	0	National Statistics Office (Dominican Republic), United Nations Children's Fund (UNICEF). Dominican Republic National Multipurpose Household Survey 2006. Santo Domingo, Dominican Republic: National Statistics Office (Dominican Republic).	3455
Dominican Republic	2007	CBH	58037	0	1426	Center for Social and Demographic Studies (Dominican Republic) (CESDEM), Macro International, Inc. Dominican Republic Demographic and Health Survey 2007. Fairfax, United States: ICF International.	19456
Dominican Republic	2010	SBH	466537	97	0	National Statistics Office (Dominican Republic), Minnesota Population Center. Dominican Republic Census 2010 from the Integrated Public Use Microdata Series, International: [Machine-readable database]. Minneapolis: University of Minnesota.	151304

Country	Year	Data Type	Children Born	Polygons	Points	Citation	GHDx ID
Dominican Republic	2013	CBH	18167	0	524	Center for Social and Demographic Studies (Dominican Republic) (CESDEM), ICF International, Ministry of Public Health and Social Assistance (Dominican Republic). Dominican Republic Demographic and Health Survey 2013. Fairfax, United States: ICF International, 2014.	77819
Dominican Republic	2014	CBH	58946	10	0	National Statistics Office (Dominican Republic), United Nations Children's Fund (UNICEF). Dominican Republic Multiple Indicator Cluster Survey 2014. New York, United States: United Nations Children's Fund (UNICEF), 2016.	200697
Ecuador	2001	SBH	615368	133	0	National Institute of Statistics and Censuses (Ecuador), Minnesota Population Center. Ecuador Population and Housing Census 2001 from the Integrated Public Use Microdata Series, International: [Machine-readable database]. Minneapolis: University of Minnesota.	3549
Ecuador	2004	CBH	24696	22	0	Center for Studies of Population and Social Development (CEPAR) (Ecuador) and Division of Reproductive Health-Centers for Disease Control and Prevention (CDC). (2005) Ecuador Reproductive Health Survey 2004. Quito, Ecuador: CEPAR.	27630
Ecuador	2005	SBH	28735	87	285	National Institute of Statistics and Censuses (Ecuador), Inter-American Development Bank (IDB). Ecuador Living Conditions Survey 2005-2006. Quito, Ecuador: National Institute of Statistics and Censuses (Ecuador).	46924

Country	Year	Data Type	Children Born	Polygons	Points	Citation	GHDx ID
Ecuador	2010	SBH	676039	140	0	Minnesota Population Center, National Institute of Statistics and Census (INEC) (Ecuador). Ecuador Population and Housing Census 2010 from the Integrated Public Use Microdata Series, International: [Machine-readable database]. Minneapolis: University of Minnesota, 2013.	105801
Egypt	2000	CBH	54780	0	997	Macro International, Inc, Population Council (Egypt). Egypt Demographic and Health Survey 2000. Fairfax, United States: ICF International.	19511
Egypt	2003	CBH	30298	0	924	El-Zanaty and Associates, Macro International, Inc, Ministry of Health and Population (Egypt), Population Council (Egypt). Egypt Interim Demographic and Health Survey 2003. Fairfax, United States: ICF International.	19529
Egypt	2005	CBH	61455	0	1298	El-Zanaty and Associates, Macro International, Inc, Ministry of Health and Population (Egypt), Population Council (Egypt). Egypt Demographic and Health Survey 2005. Fairfax, United States: ICF International.	19521
Egypt	2008	CBH	48619	0	1244	El-Zanaty and Associates, Macro International, Inc, Ministry of Health and Population (Egypt). Egypt Demographic and Health Survey 2008. Fairfax, United States: ICF International.	26842
Egypt	2014	CBH	59266	0	1807	El-Zanaty and Associates, ICF International, Ministry of Health and Population (Egypt). Egypt Demographic and Health Survey 2014. Fairfax, United States: ICF International.	154897
Egypt	2015	SBH	16984	25	0	El-Zanaty and Associates, ICF International, Ministry of Health and Population (Egypt), Population Council (Egypt). Egypt Special Demographic and Health Survey 2015. Fairfax, United States: ICF International.	157026

Country	Year	Data Type	Children Born	Polygons	Points	Citation	GHDx ID
El Salvador	2003	CBH	24442	14	0	Asociacion Demografica Salvadorena (ADS), Division of Reproductive Health-Centers for Disease Control and Prevention (CDC). (2004) El Salvador Reproductive Health Survey 2002-2003. San Salvador, El Salvador: ADS.	27599
El Salvador	2007	SBH	281507	103	0	Minnesota Population Center, General Administration of Statistics and Censuses (El Salvador), Ministry of Economy (El Salvador). El Salvador Population and Housing Census 2007 from the Integrated Public Use Microdata Series, International: [Machine-readable database]. Minneapolis: University of Minnesota, 2012.	56476
El Salvador	2008	CBH	25228	14	0	Asociacion Demografica Salvadorena (ADS), Division of Reproductive Health-Centers for Disease Control and Prevention (CDC). (2009) El Salvador Reproductive Health Survey 2008. San Salvador, El Salvador: ADS.	27606
El Salvador	2014	CBH	24689	14	0	General Administration of Statistics and Censuses (El Salvador), Ministry of Health (El Salvador), United Nations Children's Fund (UNICEF). El Salvador Multiple Indicator Cluster Survey 2014. New York, United States: United Nations Children's Fund (UNICEF), 2017.	200636
Eritrea	2002	CBH	24370	6	0	Macro International, Inc, National Statistics and Evaluation Office (Eritrea). Eritrea Demographic and Health Survey 2002. Fairfax, United States: ICF International.	19539
Ethiopia	2000	CBH	44174	0	533	Central Statistical Agency (Ethiopia), ORC Macro. Ethiopia Demographic and Health Survey 2000. Calverton, United States: ORC Macro, 2001.	19571

Country	Year	Data Type	Children Born	Polygons	Points	Citation	GHDx ID
Ethiopia	2005	CBH	39881	0	528	Macro International, Inc, Population and Housing Census Commissions Office (PHCCO). Ethiopia Demographic and Health Survey 2005. Fairfax, United States: ICF International.	19557
Ethiopia	2011	CBH	45540	0	571	Central Statistical Agency (Ethiopia), ICF Macro, Ministry of Health (Ethiopia). Ethiopia Demographic and Health Survey 2010-2011. Fairfax, United States: ICF International.	21301
Ethiopia	2014	CBH	22036	11	0	Central Statistical Agency (Ethiopia), Ministry of Health (Ethiopia), World Bank. Ethiopia Mini Demographic and Health Survey 2014.	153507
Ethiopia	2016	CBH	41392	0	622	Central Statistical Agency (Ethiopia), ICF International. Ethiopia Demographic and Health Survey 2016. Fairfax, United States: ICF International, 2017.	218568
Gabon	2001	CBH	16878	40	0	General Directorate of Statistics and Economic Studies (Gabon), Macro International, Inc. Gabon Demographic and Health Survey 2000-2001. Fairfax, United States: ICF International.	19579
Gabon	2012	CBH	23109	0	331	General Directorate of Statistics (Gabon), ICF International, Ministry of Economy, Employment and Sustainable Development (Gabon), Ministry of Health (Gabon). Gabon Demographic and Health Survey 2012. Fairfax, United States: ICF International, 2013.	76706
Ghana	2003	CBH	15086	0	410	Ghana Statistical Service, Macro International, Inc. Ghana Demographic and Health Survey 2003. Fairfax, United States: ICF International.	19627
Ghana	2006	SBH	22705	10	0	Ghana Statistical Service. Ghana Living Standards Measurement Survey 2005-2006. Accra, Ghana: Ghana Statistical Service.	4679

Country	Year	Data Type	Children Born	Polygons	Points	Citation	GHDx ID
Ghana	2006	SBH	15689	10	0	Ministry of Health (MOH) (Ghana), Ghana Statistical Service and United Nations Children's Fund (UNICEF). Ghana Multiple Indicator Cluster Survey 2006. New York, United States: United Nations Children's Fund (UNICEF).	4694
Ghana	2008	CBH	25710	347	0	Ghana Health Service, Ghana Statistical Service, Macro International, Inc. Ghana Special Demographic and Health Survey 2007-2008. Fairfax, United States: ICF International.	21173
Ghana	2008	CBH	11888	0	404	Ghana Statistical Service, Macro International, Inc, Ministry of Health (Ghana). Ghana Demographic and Health Survey 2008. Fairfax, United States: ICF International.	21188
Ghana	2008	CBH	34751	4	0	Ghana Statistical Service, Ministry of Health (Ghana), United Nations Children's Fund (UNICEF). Ghana District Multiple Indicator Cluster Survey 2007-2008.	160576
Ghana	2010	SBH	1275941	169	0	Ghana Statistical Service, Minnesota Population Center. Ghana Census 2010 from the Integrated Public Use Microdata Series, International: [Machine-readable database]. Minneapolis: University of Minnesota.	151306
Ghana	2011	SBH	2016	0	5	Institute of Statistical, Social and Economic Research, University of Ghana, United Nations Children's Fund (UNICEF). Ghana - Accra Multiple Indicator Cluster Survey 2010-2011. New York, United States: United Nations Children's Fund (UNICEF), 2014.	56241

Country	Year	Data Type	Children Born	Polygons	Points	Citation	GHDx ID
Ghana	2011	CBH	31145	0	741	Centers for Disease Control and Prevention (CDC), Ghana Statistical Service, Government of Japan, ICF Macro, Ministry of Health (Ghana), Navrongo Health Research Centre, United Nations Children's Fund (UNICEF), United Nations Population Fund (UNFPA), United States Agency for International Development (USAID). Ghana Multiple Indicator Cluster Survey 2011. New York, United States: United Nations Children's Fund (UNICEF), 2013.	63993
Ghana	2013	SBH	40591	10	0	Ghana Statistical Service, World Bank. Ghana Living Standards Measurement Survey 2012-2013. Accra, Ghana: Ghana Statistical Service.	165101
Ghana	2014	CBH	23118	0	424	Ghana Health Service, Ghana Statistical Service, ICF International. Ghana Demographic and Health Survey 2014. Fairfax, United States: ICF International, 2016.	157027
Ghana	2016	SBH	13050	0	192	Ghana Health Service, Ghana Statistical Service, ICF International, National Malaria Control Program (Ghana), National Public Health and Reference Laboratory (NHPRL)(Ghana). Ghana Malaria Indicator Survey 2016. Fairfax, United States: ICF International.	286788
Ghana	2017	CBH	59198	0	897	Ghana Health Service, Ghana Statistical Service, ICF International. Ghana Special Demographic and Health Survey 2017. Fairfax, United States: ICF International, 2018.	218572
Guatemala	2002	CBH	28731	373	370	Guatemala Ministry of Health and Social Assistance, University of Valle, Division of Reproductive Health-Centers for Disease Control and Prevention (CDC). (2003) Guatemala Reproductive Health Survey 2002. Atlanta, United States: Centers for Disease Control and Prevention (CDC).	27563

Country	Year	Data Type	Children Born	Polygons	Points	Citation	GHDx ID
Guatemala	2009	CBH	45017	22	0	Guatemala Ministry of Health and Social Assistance, University of Valle and Division of Reproductive Health-Centers for Disease Control and Prevention (CDC). Guatemala Reproductive Health Survey 2008-2009. Atlanta, United States: Centers for Disease Control and Prevention (CDC).	4779
Guatemala	2011	SBH	37495	21	0	National Statistics Institute (Guatemala). Guatemala National Survey of Living Conditions 2011. Guatemala City, Guatemala: National Statistics Institute (Guatemala).	352625
Guatemala	2015	CBH	55398	0	853	ICF International, Institute of Nutrition of Central America and Panama, Ministry of Public Health and Social Assistance (Guatemala), National Statistics Institute (Guatemala), Secretary of Planning and Programming of the Presidency (Segeplan) (Guatemala). Guatemala Demographic and Health Survey 2014-2015. Fairfax, United States: ICF International, 2017.	157031
Guinea	2005	CBH	27115	0	291	Macro International, Inc, National Statistics Directorate (Guinea). Guinea Demographic and Health Survey 2005. Fairfax, United States: ICF International.	19683
Guinea	2012	CBH	27683	0	300	ICF Macro, Ministry of Health and Public Hygiene (Guinea), National Institute of Statistics (Guinea). Guinea Demographic and Health Survey 2012. Fairfax, United States: ICF International.	69761
Guinea	2016	CBH	27046	6	0	National Institute of Public Health (NPHI) (Guinea), National Institute of Statistics (Guinea), National Malaria Control Program (Guinea), United Nations Children’s Fund (UNICEF). Guinea Multiple Indicator Cluster Survey 2016. New York, United States: United Nations Children’s Fund (UNICEF), 2018.	303458

Country	Year	Data Type	Children Born	Polygons	Points	Citation	GHDx ID
Guinea-Bissau	2006	SBH	24016	9	0	United Nations Children's Fund (UNICEF), Government of Guinea-Bissau. Guinea-Bissau Multiple Indicator Cluster Survey 2006. New York, United States: United Nations Children's Fund (UNICEF).	4818
Guinea-Bissau	2014	CBH	27607	9	0	National Statistics Institute (Guinea-Bissau), United Nations Children's Fund (UNICEF). Guinea-Bissau Multiple Indicator Cluster Survey 2014. New York, United States: United Nations Children's Fund (UNICEF), 2016.	174049
Guyana	2000	SBH	11691	10	0	Bureau of Statistics (Guyana), United Nations Children's Fund (UNICEF). Guyana Multiple Indicator Cluster Survey 2000. New York, United States: United Nations Children's Fund (UNICEF)	4916
Guyana	2005	CBH	4923	9	0	Central Bureau of Statistics (Ghana), Guyana Responsible Parenthood Association (GRPA), Ministry of Health (Guyana), ORC Macro, Pan American Health Organization (PAHO). Guyana AIDS Indicator Survey 2005. Fairfax, United States: ICF International.	4837
Guyana	2007	SBH	12157	10	0	United Nations Children's Fund (UNICEF), Bureau of Statistics (Guyana). Guyana Multiple Indicator Cluster Survey 2006. New York, United States: United Nations Children's Fund (UNICEF).	4926
Guyana	2009	CBH	10929	0	312	Bureau of Statistics (Guyana), ICF Macro, Ministry of Health (Guyana). Guyana Demographic and Health Survey 2009. Fairfax, United States: ICF International, 2011.	21348
Guyana	2014	CBH	11161	10	0	Bureau of Statistics (Guyana), Ministry of Health (Guyana), United Nations Children's Fund (UNICEF). Guyana Multiple Indicator Cluster Survey 2014. New York, United States: United	200598

Country	Year	Data Type	Children Born	Polygons	Points	Citation	GHDx ID
						Nations Children's Fund (UNICEF), 2016.	
Haiti	2000	CBH	26437	0	317	Haitian Institute of Childhood (IHE), Macro International, Inc. Haiti Demographic and Health Survey 2000. Fairfax, United States: ICF International.	19708
Haiti	2003	SBH	439200	42	0	Minnesota Population Center, Haitian Institute of Statistics and Informatics. Haiti Population and Housing Census 2003 from the Integrated Public Use Microdata Series, International: [Machine-readable database]. Minneapolis: University of Minnesota, 2013	106473
Haiti	2006	CBH	24830	0	333	Haitian Institute of Childhood (IHE), Haitian Institute of Statistics and Informatics, Macro International, Inc. Haiti Demographic and Health Survey 2005-2006. Fairfax, United States: ICF International.	19720
Haiti	2008	CBH	4711	9	0	Global Fund to Fight Aids Tuberculosis and Malaria (GFATM). Haiti Global Fund Household Survey 2008.	26680
Haiti	2012	CBH	29013	0	438	Centers for Disease Control and Prevention (CDC), Haitian Institute of Childhood (IHE), Haitian Institute of Statistics and Informatics, Macro International, Inc. Haiti Demographic and Health Survey 2012. Fairfax, United States: ICF International.	65118
Haiti	2016	CBH	27809	10	450	Haitian Institute of Childhood (IHE), Haitian Institute of Statistics and Informatics, ICF International, Ministry of Public Health and Population (Haiti). Haiti Demographic and Health Survey 2016-2017. Fairfax, United States: ICF International.	218574

Country	Year	Data Type	Children Born	Polygons	Points	Citation	GHDx ID
Honduras	2001	CBH	23535	16	0	Honduras Family Planning Association (ASHONPLAFA), Ministry of Health (Honduras), and Division of Reproductive Health-Centers for Disease Control and Prevention (CDC). Honduras Reproductive Health Survey 2001. Tegucigalpa, Honduras: Honduras Family Planning Association (ASHONPLAFA).	27551
Honduras	2001	SBH	343847	111	0	National Institute of Statistics (Honduras), Minnesota Population Center. Honduras Population and Housing Census 2001 from the Integrated Public Use Microdata Series, International. Minneapolis, MN: IPUMS, 2018. https://doi.org/10.18128/D020.V7.1	367563
Honduras	2004	SBH	21811	18	0	National Institute of Statistics (Honduras). Honduras Survey of Living Conditions 2004. Tegucigalpa, Honduras: National Institute of Statistics (Honduras).	5009
Honduras	2006	CBH	50093	16	0	Macro International, Inc, National Institute of Statistics (Honduras), Secretary of Health (Honduras). Honduras Demographic and Health Survey 2005-2006. Fairfax, United States: ICF International.	19728
Honduras	2012	CBH	49263	0	1127	ICF Macro, National Institute of Statistics (Honduras). Honduras Demographic and Health Survey 2011-2012. Fairfax, United States: ICF International.	95440
India	2000	CBH	268879	26	0	International Institute for Population Sciences (India), Macro International, Inc. India Demographic and Health Survey 1998-1999. Calverton, United States: Macro International, Inc.	19950

Country	Year	Data Type	Children Born	Polygons	Points	Citation	GHDx ID
India	2004	SBH	1391228	585	0	International Institute for Population Sciences (India). India District Level Household Survey 2002-2005. Mumbai, India: International Institute for Population Sciences (India).	23219†
India	2006	CBH	256782	29	0	International Institute for Population Sciences (India), Macro International, Inc. India Demographic and Health Survey 2005-2006. Fairfax, United States: ICF International.	19963
India	2008	SBH	1818042	590	0	International Institute for Population Sciences (India). India District Level Household Survey 2007-2008. Mumbai, India: International Institute for Population Sciences (India), 2010.	23258†
India	2013	SBH	31956497	277	0	Office of the Registrar General and Census Commissioner (India). India Annual Health Survey Data 2010-2013.	234353†
India	2014	SBH	259034	275	0	International Institute for Population Sciences (India). India District Level Household Survey 2012-2014. New Delhi, India: Ministry of Health and Family Welfare (India).	165390
India	2016	CBH	1315617	82	28387	ICF International, International Institute for Population Sciences (India), Ministry of Health and Family Welfare (India). India Demographic and Health Survey 2015-2016. Fairfax, United States: ICF International, 2018.	157050
Indonesia	2001	SBH	458846	6	9488	Central Bureau of Statistics (Indonesia), Ministry of Health (Indonesia), World Bank. Indonesia National Socioeconomic Survey 2001.	6842
Indonesia	2002	SBH	432996	6	9009	Statistics Indonesia. Indonesia National Socioeconomic Survey 2002.	43510
Indonesia	2003	SBH	449842	37	0	Statistics Indonesia. Indonesia National Socioeconomic Survey 2003.	6874

Country	Year	Data Type	Children Born	Polygons	Points	Citation	GHDx ID
Indonesia	2003	CBH	79791	0	1318	Macro International, Inc, Ministry of Health (Indonesia), National Family Planning Coordinating Board (Indonesia), Statistics Indonesia. Indonesia Demographic and Health Survey 2002-2003. Fairfax, United States: ICF International.	20011
Indonesia	2004	SBH	501844	6	10427	Statistics Indonesia. Indonesia National Socioeconomic Survey 2004.	6904
Indonesia	2005	SBH	510692	5	11494	Statistics Indonesia. Indonesia National Socioeconomic Survey 2005.	5376
Indonesia	2006	SBH	545163	84	12575	Statistics Indonesia. Indonesia National Socioeconomic Survey 2006.	5401
Indonesia	2007	SBH	582333	92	12521	Statistics Indonesia. Indonesia National Socioeconomic Survey 2007.	6970
Indonesia	2007	CBH	84726	33	0	Macro International, Inc, Ministry of Health (Indonesia), National Family Planning Coordinating Board (Indonesia), Statistics Indonesia. Indonesia Demographic and Health Survey 2007. Fairfax, United States: ICF International.	20021
Indonesia	2008	SBH	131645	99	3400	Statistics Indonesia. Indonesia National Socioeconomic Survey 2008.	43526
Indonesia	2009	SBH	129963	93	3355	Statistics Indonesia. Indonesia National Socioeconomic Survey 2009.	43552
Indonesia	2010	SBH	125345	98	3318	Statistics Indonesia. Indonesia National Socioeconomic Survey 2010.	30235
Indonesia	2010	SBH	10489232	485	0	Minnesota Population Center, Statistics Indonesia. Indonesia Population Census 2010 from the Integrated Public Use Microdata Series, International: [Machine-readable database]. Minneapolis: University of Minnesota, 2012.	56558
Indonesia	2011	SBH	130728	103	5685	Statistics Indonesia. Indonesia National Socioeconomic Survey 2011.	85265

Country	Year	Data Type	Children Born	Polygons	Points	Citation	GHDx ID
Indonesia	2011	SBH	6028	3	0	Ministry of Home Affairs (Indonesia), National Development Planning Agency (BAPPENAS) (Indonesia), Statistics Indonesia, United Nations Children's Fund (UNICEF). Indonesia - West Papua Multiple Indicator Cluster Survey 2011. New York, United States: United Nations Children's Fund (UNICEF), 2013.	104042
Indonesia	2011	SBH	6246	3	0	Ministry of Home Affairs (Indonesia), National Development Planning Agency (BAPPENAS) (Indonesia), Statistics Indonesia, United Nations Children's Fund (UNICEF). Indonesia - Papua Multiple Indicator Cluster Survey 2011. New York, United States: United Nations Children's Fund (UNICEF), 2013.	104043
Indonesia	2012	CBH	83650	33	0	ICF International, Ministry of Health (Indonesia), National Population and Family Planning Board (Indonesia), Statistics Indonesia. Indonesia Demographic and Health Survey 2012. Fairfax, United States: ICF International.	76705
Indonesia	2012	SBH	534256	497	0	Central Bureau of Statistics (Indonesia). Indonesia National Socioeconomic Survey 2012. Jakarta, Indonesia: Central Bureau of Statistics (Indonesia).	150884
Indonesia	2013	SBH	518178	497	0	Statistics Indonesia. Indonesia National Socioeconomic Survey 2013. Jakarta, Indonesia: Statistics Indonesia.	151184
Indonesia	2014	SBH	517089	445	0	Statistics Indonesia. Indonesia National Socioeconomic Survey 2014.	165186
Indonesia	2015	CBH	1228790	476	0	Statistics Indonesia. Indonesia Intercensal Population Survey 2015.	237943
Indonesia	2015	SBH	493641	449	0	Statistics Indonesia. Indonesia National Socioeconomic Survey 2015.	238332
Indonesia	2016	SBH	483674	450	0	Statistics Indonesia. Indonesia National Socioeconomic Survey 2016.	282087

Country	Year	Data Type	Children Born	Polygons	Points	Citation	GHDx ID
Indonesia	2017	SBH	487604	34	0	Central Bureau of Statistics (Indonesia). Indonesia National Socioeconomic Survey 2017. Jakarta, Indonesia: Central Bureau of Statistics (Indonesia), 2018.	395694
Iran	2006	SBH	632125	333	0	Statistical Centre of Iran, Minnesota Population Center. Iran General Census of Population and Housing 2006 from the Integrated Public Use Microdata Series, International: Version 6.1 [Machine-readable database]. Minneapolis: University of Minnesota, 2011.	39396
Iran	2011	SBH	624428	396	0	Statistical Centre of Iran. Iran National Population and Housing Census 2011. Tehran, Iran: Statistical Centre of Iran.	81291
Iran	2016	SBH	608289	429	0	Statistical Centre of Iran. Iran Population and Housing Census 2016.	299134
Iraq	2006	CBH	62359	18	0	United Nations Children’s Fund (UNICEF), Central Organization for Statistics and Information Technology (Iraq), Kurdistan Regional Statistics Office. Iraq Multiple Indicator Cluster Survey 2006. New York, United States: United Nations Children’s Fund (UNICEF).	7028
Iraq	2007	SBH	34284	18	0	Ministry of Health (Iraq), Central Organization for Statistics and Information Technology (Iraq), Kurdistan Regional Statistics Office, World Health Organization (WHO), Ministry of Health (Kurdistan). Iraq Family Health Survey 2006-2007.	23429
Iraq	2011	CBH	136878	48	0	Central Organization for Statistics and Information Technology (Iraq), Kurdistan Regional Statistics Office, Ministry of Health (Iraq), United Nations Children’s Fund (UNICEF). Iraq Multiple Indicator Cluster Survey 2011. New York, United States: United Nations Children’s Fund (UNICEF), 2013.	76707

Country	Year	Data Type	Children Born	Polygons	Points	Citation	GHDx ID
Jamaica	2001	SBH	93846	14	0	Statistical Institute of Jamaica (STATIN), Minnesota Population Center. Jamaica Population Census 2001 from the Integrated Public Use Microdata Series, International: [Machine-readable database]. Minneapolis: University of Minnesota, 2011.	39450
Jamaica	2005	SBH	7206	14	0	Statistical Institute of Jamaica (STATIN) and United Nations Children's Fund (UNICEF). Jamaica Multiple Indicator Cluster Survey 2005. New York, United States: United Nations Children's Fund (UNICEF).	7149
Jordan	2002	CBH	25296	0	495	Department of Statistics (Jordan), Macro International, Inc. Jordan Demographic and Health Survey 2002. Fairfax, United States: ICF International.	20073
Jordan	2007	CBH	43460	0	924	Department of Statistics (Jordan), Macro International, Inc. Jordan Demographic and Health Survey 2007. Fairfax, United States: ICF International.	20083
Jordan	2009	CBH	38199	12	0	Department of Statistics (Jordan), ICF Macro. Jordan Interim Demographic and Health Survey 2009. Fairfax, United States: ICF International, 2010.	21206
Jordan	2012	CBH	42275	0	806	Department of Statistics (Jordan), ICF International. Jordan Demographic and Health Survey 2012. Fairfax, United States: ICF International.	77517
Kenya	2003	CBH	22074	0	399	Centers for Disease Control and Prevention (CDC), Central Bureau of Statistics (Kenya), Macro International, Inc, Ministry of Health (Kenya), National Council for Population and Development (Kenya). Kenya Demographic and Health Survey 2003. Fairfax, United States: ICF International.	20145

Country	Year	Data Type	Children Born	Polygons	Points	Citation	GHDx ID
Kenya	2006	SBH	37588	0	1339	Central Bureau of Statistics (Kenya), UK Department for International Development (DFID), United States Agency for International Development (USAID), European Union (EU), Danish International Development Agency (DANIDA), World Bank (WB), United Nations Development Programme (UNDP). Kenya Integrated Household Budget Survey 2005-2006. Nairobi, Kenya: Central Bureau of Statistics (Kenya).	7375†
Kenya	2007	SBH	14769	0	383	Centers for Disease Control and Prevention (CDC), KEMRI Wellcome Trust Research Programme (KWTRP), Kenya National Bureau of Statistics, Ministry of Public Health and Sanitation (Kenya), National Coordinating Agency for Population and Development (Kenya), Population Services International (PSI). Kenya Malaria Indicator Survey 2007.	57990†
Kenya	2007	SBH	26279	0	387	Centers for Disease Control and Prevention (CDC), Kenya Medical Research Institute (KEMRI), Kenya National Bureau of Statistics, Ministry of Public Health and Sanitation (Kenya), National AIDS Control Council (Kenya), National AIDS and STI Control Program (Kenya), National Coordinating Agency for Population and Development (Kenya), National Public Health Laboratory Services, Ministry of Public Health and Sanitation (Kenya), United States Agency for International Development (USAID). Kenya AIDS Indicator Survey 2007. Nairobi, Kenya: Kenya National Bureau of Statistics.	133219

Country	Year	Data Type	Children Born	Polygons	Points	Citation	GHDx ID
Kenya	2007	SBH	4036	0	76	Kenya National Bureau of Statistics, United Nations Children's Fund (UNICEF). Kenya - North Eastern Province Multiple Indicator Cluster Survey 2007. Nairobi, Kenya: Kenya National Bureau of Statistics.	155335
Kenya	2008	CBH	41800	0	590	Kenya National Bureau of Statistics, United Nations Children's Fund (UNICEF). Kenya - Eastern Province Multiple Indicator Cluster Survey 2008. Nairobi, Kenya: Kenya National Bureau of Statistics.	7401
Kenya	2009	CBH	22534	0	397	ICF Macro, Kenya Medical Research Institute (KEMRI), Kenya National Bureau of Statistics, Ministry of Public Health and Sanitation (Kenya), National AIDS and STI Control Programme (NASCOP) (Kenya), National Aids Control Council (NACC), National Coordinating Agency for Population and Development (Kenya). Kenya Demographic and Health Survey 2008-2009. Fairfax, United States: ICF International.	21365
Kenya	2009	SBH	2388668	158	0	Minnesota Population Center, Kenya National Bureau of Statistics. Kenya Population Census 2009 from the Integrated Public Use Microdata Series, International: [Machine-readable database]. Minneapolis: University of Minnesota, 2013.	106512
Kenya	2011	CBH	18631	0	289	Kenya National Bureau of Statistics, United Nations Children's Fund (UNICEF). Kenya - Nyanza Province Multiple Indicator Cluster Survey 2011. Nairobi, Kenya: Kenya National Bureau of Statistics.	135416

Country	Year	Data Type	Children Born	Polygons	Points	Citation	GHDx ID
Kenya	2014	CBH	83591	0	1585	ICF International, Kenya Medical Research Institute (KEMRI), Kenya National Bureau of Statistics, Ministry of Health (Kenya), National AIDS Control Council (Kenya), National Council for Population and Development (Kenya). Kenya Demographic and Health Survey 2014. Fairfax, United States: ICF International.	157057
Kenya	2015	SBH	14087	0	245	ICF International, Kenya National Bureau of Statistics, National Malaria Control Program (NMCP) (Kenya). Kenya Malaria Indicator Survey 2015. Fairfax, United States: ICF International, 2015.	218579
Kyrgyzstan	2006	SBH	12820	8	0	United Nations Children's Fund (UNICEF), National Statistical Committee of the Kyrgyz Republic. Kyrgyzstan Multiple Indicator Cluster Survey 2005-2006. New York, United States: United Nations Children's Fund (UNICEF).	7540
Kyrgyzstan	2009	SBH	280046	56	0	Minnesota Population Center, National Statistical Committee of the Kyrgyz Republic. Kyrgyzstan Population and Housing Census 2009 from the Integrated Public Use Microdata Series, International: [Machine-readable database]. Minneapolis: University of Minnesota, 2013.	106520
Kyrgyzstan	2012	CBH	16180	0	314	ICF International, Ministry of Health (Kyrgyzstan), National Statistical Committee of the Kyrgyz Republic. Kyrgyzstan Demographic and Health Survey 2012. Fairfax, United States: ICF International.	77518

Country	Year	Data Type	Children Born	Polygons	Points	Citation	GHDx ID
Kyrgyzstan	2014	CBH	14527	9	0	National Statistical Committee of the Kyrgyz Republic, United Nations Children's Fund (UNICEF). Kyrgyzstan Multiple Indicator Cluster Survey 2014. New York, United States: United Nations Children's Fund (UNICEF), 2015.	162283
Laos	2012	CBH	56802	17	0	Ministry of Education and Sports (Laos), Ministry of Health (Laos), Ministry of Planning and Investment (Laos). Laos Multiple Indicator Cluster Survey 2011-2012. New York, United States: United Nations Children's Fund (UNICEF), 2013.	103973
Laos	2017	CBH	54163	18	0	Lao Statistics Bureau, Ministry of Education and Sports (Laos), Ministry of Health (Laos), United Nations Children's Fund (UNICEF). Laos Multiple Indicator Cluster Survey 2017. New York, United States: United Nations Children's Fund (UNICEF), 2018.	375362
Lesotho	2005	CBH	14708	0	381	Bureau of Statistics (Lesotho), Macro International, Inc, Ministry of Health and Social Welfare (Lesotho). Lesotho Demographic and Health Survey 2004-2005. Fairfax, United States: ICF International.	20167
Lesotho	2006	SBH	84487	63	0	Bureau of Statistics (Lesotho), Minnesota Population Center. Lesotho Population and Housing Census 2006 from the Integrated Public Use Microdata Series, International. Minneapolis, MN: IPUMS, 2018. https://doi.org/10.18128/D020.V7.1	367585
Lesotho	2010	CBH	14429	0	395	ICF Macro, Ministry of Health and Social Welfare (Lesotho). Lesotho Demographic and Health Survey 2009-2010. Fairfax, United States: ICF International.	21382
Lesotho	2014	CBH	11710	0	399	ICF International, Ministry of Health and Social Welfare (Lesotho). Lesotho Demographic and Health Survey 2014.	157058

Country	Year	Data Type	Children Born	Polygons	Points	Citation	GHDx ID
						Fairfax, United States: ICF International.	
Liberia	2007	CBH	22123	0	291	Liberia Institute for Statistics and Geo-information Services (LISGIS), Macro International, Inc. Liberia Demographic and Health Survey 2006-2007. Fairfax, United States: ICF International.	20191
Liberia	2008	SBH	209401	47	0	Liberia Institute for Statistics and Geo-information Services (LISGIS), Minnesota Population Center. Liberia Census 2008 from the Integrated Public Use Microdata Series, International: [Machine-readable database]. Minneapolis: University of Minnesota.	151310
Liberia	2009	CBH	14872	0	150	ICF Macro, Liberia Institute for Statistics and Geo-information Services (LISGIS), National Malaria Control Program (Liberia). Liberia Malaria Indicator Survey 2008-2009. Fairfax, United States: ICF International.	34279
Liberia	2011	SBH	13917	0	150	ICF International, Liberia Institute for Statistics and Geo-information Services (LISGIS), National Malaria Control Program (Liberia). Liberia Malaria Indicator Survey 2011. Fairfax, United States: ICF International, 2012.	56828
Liberia	2013	CBH	30804	0	322	ICF International, Liberia Institute for Statistics and Geo-information Services (LISGIS), National AIDS and STI Control Program (NACP), Ministry of Health and Social Welfare (Liberia). Liberia Demographic and Health Survey 2013. Fairfax, United States: ICF International.	77385
Liberia	2016	SBH	13869	0	150	ICF International, Liberia Institute for Statistics and Geo-information Services (LISGIS), National Malaria Control Program (Liberia). Liberia Malaria Indicator Survey 2016. Fairfax, United States: ICF International, 2017.	286768

Country	Year	Data Type	Children Born	Polygons	Points	Citation	GHDx ID
Libya	2007	CBH	49554	22	0	League of Arab States, National Center for Disease Control (Libya), Pan Arab Project for Family Health (PAPFAM). Libya Family Health Survey 2007.	107340
Madagascar	2004	CBH	20799	6	0	Macro International, Inc, National Institute of Statistics (Madagascar). Madagascar Demographic and Health Survey 2003-2004. Fairfax, United States: ICF International.	20223
Madagascar	2009	CBH	48464	0	585	ICF Macro, National Institute of Statistics (Madagascar). Madagascar Demographic and Health Survey 2008-2009. Fairfax, United States: ICF International.	21409
Madagascar	2011	SBH	23462	0	266	ICF International, National Institute of Statistics (Madagascar), National Program for the Fight Against Malaria (PNLP) (Madagascar), Pasteur Institute of Madagascar (IPM). Madagascar Malaria Indicator Survey 2011. Fairfax, United States: ICF International.	69806
Madagascar	2012	CBH	9956	0	127	National Institute of Statistics (Madagascar), United Nations Children's Fund (UNICEF). Madagascar - South Multiple Indicator Cluster Survey 2012. New York, United States: United Nations Children's Fund (UNICEF), 2015.	125594
Madagascar	2013	SBH	22075	0	274	ICF International, National Institute of Statistics (Madagascar), National Program for the Fight Against Malaria (PNLP) (Madagascar), Pasteur Institute of Madagascar (IPM). Madagascar Malaria Indicator Survey 2013. Fairfax, United States: ICF International, 2013.	111438

Country	Year	Data Type	Children Born	Polygons	Points	Citation	GHDx ID
Madagascar	2016	SBH	27819	22	0	ICF International, Ministry of Public Health (Madagascar), National Institute of Statistics (Madagascar), National Program for the Fight Against Malaria (PNLP) (Madagascar), Pasteur Institute of Madagascar (IPM). Madagascar Malaria Indicator Survey 2016. Fairfax, United States: ICF International, 2017.	218580
Malawi	2000	CBH	40421	0	559	Macro International, Inc, National Statistical Office of Malawi. Malawi Demographic and Health Survey 2000. Fairfax, United States: ICF International.	20252
Malawi	2005	CBH	35883	0	520	Macro International, Inc, National Statistical Office of Malawi. Malawi Demographic and Health Survey 2004-2005. Fairfax, United States: ICF International.	20263
Malawi	2006	CBH	78960	26	0	United Nations Children's Fund (UNICEF), National Statistics Office (Malawi). Malawi Multiple Indicator Cluster Survey 2006. New York, United States: United Nations Children's Fund (UNICEF).	7919
Malawi	2008	SBH	875423	225	0	National Statistical Office (Malawi), Minnesota Population Center. Malawi Population and Housing Census 2008 from the Integrated Public Use Microdata Series, International: [Machine-readable database]. Minneapolis: University of Minnesota, 2011.	40186
Malawi	2010	CBH	72301	0	827	ICF Macro, National Statistical Office of Malawi. Malawi Demographic and Health Survey 2010. Fairfax, United States: ICF International.	21393
Malawi	2012	SBH	8026	0	140	ICF International, National Malaria Control Program (Malawi). Malawi Malaria Indicator Survey 2012. Fairfax, United States: ICF International.	77387

Country	Year	Data Type	Children Born	Polygons	Points	Citation	GHDx ID
Malawi	2014	SBH	8126	0	140	ICF International, Ministry of Health (Malawi), National Malaria Control Program (Malawi), National Statistical Office of Malawi. Malawi Malaria Indicator Survey 2014. Fairfax, United States: ICF International, 2015.	157059
Malawi	2014	CBH	72579	31	0	National Statistical Office of Malawi, United Nations Children's Fund (UNICEF). Malawi Multiple Indicator Cluster Survey 2013-2014. New York, United States: United Nations Children's Fund (UNICEF), 2015.	161662
Malawi	2016	CBH	68074	0	850	Emory University and Centers for Disease Control & Prevention Collaboration, ICF International, Ministry of Health (Malawi), National Statistical Office of Malawi. Malawi Demographic and Health Survey 2015-2016. Fairfax, United States: ICF International, 2017.	218581
Malawi	2017	SBH	39128	0	148	ICF International, National Malaria Control Program (Malawi). Malawi Malaria Indicator Survey 2017. Fairfax, United States: ICF International, 2018.	286769
Mali	2001	CBH	48407	0	399	Macro International, Inc, National Directorate of Statistics and Informatics (DNSI) (Mali), Planning and Statistics Unit, Ministry of Health (Mali). Mali Demographic and Health Survey 2001. Fairfax, United States: ICF International.	20315
Mali	2006	CBH	52140	0	405	Macro International, Inc, Ministry of Health (Mali), National Directorate of Statistics and Informatics (DNSI) (Mali). Mali Demographic and Health Survey 2006. Fairfax, United States: ICF International.	20274

Country	Year	Data Type	Children Born	Polygons	Points	Citation	GHDx ID
Mali	2009	SBH	840735	244	0	Central Census Bureau (Mali), Minnesota Population Center. Mali Census 2009 from the Integrated Public Use Microdata Series, International: [Machine-readable database]. Minneapolis: University of Minnesota.	151311
Mali	2013	CBH	33803	0	413	ICF International, INFO-STAT (Mali), Ministry of Health (Mali), National Institute of Statistics (INSTAT) (Mali), Planning and Statistics Unit, Ministry of Health (Mali). Mali Demographic and Health Survey 2012-2013. Fairfax, United States: ICF International, 2014.	77388
Mali	2015	SBH	28960	0	177	ICF International, INFO-STAT (Mali), Ministry of Health and Public Hygiene (Mali), National Institute of Public Health Research (INRSP) (Mali), National Institute of Statistics (INSTAT) (Mali). Mali Malaria Indicator Survey 2015. Fairfax, United States: ICF International, 2016.	218587
Mali	2015	CBH	55820	8	0	Ministry of Health (Mali), Ministry of Planning (Mali), National Institute of Statistics (INSTAT) (Mali), United Nations Children’s Fund (UNICEF). Mali Multiple Indicator Cluster Survey 2015. New York, United States: United Nations Children’s Fund (UNICEF), 2017.	248224
Mauritania	2001	CBH	19202	13	0	Macro International, Inc, National Office of Statistics (Mauritania). Mauritania Demographic and Health Survey 2000-2001. Fairfax, United States: ICF International.	20322
Mauritania	2004	CBH	13246	13	0	Macro International, Inc, Ministry of Health and Social Affairs (Mauritania), National Office of Statistics (Mauritania). Mauritania Special Demographic and Health Survey 2003-2004.	26871

Country	Year	Data Type	Children Born	Polygons	Points	Citation	GHDx ID
Mauritania	2007	SBH	35683	196	0	National Office of Statistics (Mauritania), United Nations Children's Fund (UNICEF). Mauritania Multiple Indicator Cluster Survey 2007. New York, United States: United Nations Children's Fund (UNICEF).	8115
Mauritania	2011	CBH	33648	194	0	National Office of Statistics (Mauritania), United Nations Children's Fund (UNICEF). Mauritania Multiple Indicator Cluster Survey 2011. New York, United States: United Nations Children's Fund (UNICEF), 2015.	152783
Mauritania	2015	CBH	75012	13	0	National Office of Statistics (Mauritania), United Nations Children's Fund (UNICEF). Mauritania Multiple Indicator Cluster Survey 2015. New York, United States: United Nations Children's Fund (UNICEF), 2018.	267343
Mongolia	2003	SBH	21076	20	0	Ministry of Health (Mongolia), National Statistical Office of Mongolia, United Nations Population Fund (UNFPA). Mongolia Reproductive Health Survey 2003. Ulaanbaatar, Mongolia: National Statistical Office of Mongolia, 2004.	24159
Mongolia	2005	SBH	15378	22	0	National Statistical Office of Mongolia, United Nations Children's Fund (UNICEF). Mongolia Multiple Indicator Cluster Survey 2005. New York, United States: United Nations Children's Fund (UNICEF).	8777
Mongolia	2010	SBH	18061	217	0	National Statistical Office of Mongolia, United Nations Children's Fund (UNICEF). Mongolia Multiple Indicator Cluster Survey 2010. New York, United States: United Nations Children's Fund (UNICEF), 2013.	76704
Mongolia	2012	SBH	3471	23	0	National Statistical Office of Mongolia, United Nations Children's Fund (UNICEF). Mongolia - Khuvsgul Multiple Indicator Cluster Survey 2012. New York, United States: United Nations Children's Fund (UNICEF), 2015.	189045

Country	Year	Data Type	Children Born	Polygons	Points	Citation	GHDx ID
Mongolia	2012	SBH	1628	1	0	National Statistical Office of Mongolia, United Nations Children's Fund (UNICEF). Mongolia - Nalaikh District Multiple Cluster Indicator Survey 2012. New York, United States: United Nations Children's Fund (UNICEF), 2017.	189048
Mongolia	2016	CBH	24448	528	0	Government of Mongolia, National Statistical Office of Mongolia, United Nations Children's Fund (UNICEF), United Nations Population Fund (UNFPA). Mongolia Multiple Indicator Cluster Survey 2013. New York, United States: United Nations Children's Fund (UNICEF), 2016.	150866
Mongolia	2016	CBH	4453	82	0	National Statistical Office of Mongolia, United Nations Children's Fund (UNICEF). Mongolia - Khuvsgul Multiple Indicator Cluster Survey 2016. New York, United States: United Nations Children's Fund (UNICEF), 2018.	335994
Morocco	2004	CBH	32494	0	480	League of Arab States, Macro International, Inc, Ministry of Health (Morocco). Morocco Demographic and Health Survey 2003-2004. Fairfax, United States: ICF International.	20361
Morocco	2004	SBH	726578	60	0	Minnesota Population Center, High Commission for Planning (Morocco). Morocco Population and Housing Census 2004 from the Integrated Public Use Microdata Series, International: [Machine-readable database]. Minneapolis: University of Minnesota, 2012.	56492
Morocco	2011	SBH	30414	59	0	Ministry of Health (Morocco), Pan Arab Project for Family Health (PAPFAM), United Nations Children's Fund (UNICEF), United Nations Population Fund (UNFPA), World Health Organization (WHO). Morocco National Survey on Population and Family Health 2010-2011.	126909

Country	Year	Data Type	Children Born	Polygons	Points	Citation	GHDx ID
Mozambique	2003	CBH	37443	11	0	Macro International, Inc, National Institute of Statistics (INE) (Mozambique). Mozambique Demographic and Health Survey 2003-2004. Fairfax, United States: ICF International.	20394
Mozambique	2007	SBH	1359379	337	0	Minnesota Population Center, Mozambique National Statistics Institute. Mozambique Census 2007 from the Integrated Public Use Microdata Series, International: [Machine-readable database]. Minneapolis: University of Minnesota, 2015.	227143
Mozambique	2009	SBH	29263	0	270	ICF Macro, Ministry of Health (Mozambique), National Institute of Statistics (INE) (Mozambique). Mozambique AIDS Indicator Survey 2009. Fairfax, United States: ICF International, 2010.	8906
Mozambique	2009	CBH	42215	618	67	United Nations Children's Fund (UNICEF), National Statistics Institute (Mozambique). Mozambique Multiple Indicator Cluster Survey 2008-2009. New York, United States: United Nations Children's Fund (UNICEF).	27031
Mozambique	2011	CBH	37984	0	609	ICF Macro, Manhica Health Research Center (CISM), Ministry of Health (Mozambique), National Institute of Statistics (INE) (Mozambique). Mozambique Demographic and Health Survey 2011. Fairfax, United States: ICF International.	55975
Mozambique	2015	CBH	6356	0	20	Centers for Disease Control and Prevention (CDC), ICF International, Ministry of Health (Mozambique), National Institute of Health (Mozambique), National Institute of Statistics (INE) (Mozambique). Mozambique AIDS Indicator Survey 2015. Fairfax, United States: ICF International, 2018.	157060

Country	Year	Data Type	Children Born	Polygons	Points	Citation	GHDx ID
Myanmar	2010	CBH	60796	17	0	Ministry of Health (Myanmar), Ministry of National Planning and Economic Development (Myanmar), United Nations Children's Fund (UNICEF). Myanmar Multiple Indicator Cluster Survey 2009-2010.	90696
Myanmar	2016	CBH	22989	0	441	ICF International, Ministry of Health and Sports (Myanmar). Myanmar Demographic and Health Survey 2015-2016. Fairfax, United States: ICF International, 2017.	157061
Namibia	2000	CBH	14946	0	259	Macro International, Inc, Ministry of Health and Social Services (Namibia), National Planning Commission (Namibia). Namibia Demographic and Health Survey 2000. Calverton, United States: Macro International, Inc.	20417
Namibia	2007	CBH	19522	0	491	Macro International, Inc, Ministry of Health and Social Services (Namibia). Namibia Demographic and Health Survey 2006-2007. Fairfax, United States: ICF International.	20428
Namibia	2011	SBH	206980	13	0	Namibia Statistics Agency, Statistics South Africa. Namibia Population and Housing Census 2011. Windhoek, Namibia: Namibia Statistics Agency, 2013.	134132
Namibia	2013	CBH	18090	0	549	ICF International, Ministry of Health and Social Services (Namibia), Namibia Institute of Pathology, Namibia Statistics Agency. Namibia Demographic and Health Survey 2013. Fairfax, United States: ICF International.	150382
Nepal	2001	CBH	28955	0	251	Macro International, Inc, Ministry of Health and Population (Nepal), New ERA. Nepal Demographic and Health Survey 2001. Fairfax, United States: ICF International.	20450

Country	Year	Data Type	Children Born	Polygons	Points	Citation	GHDx ID
Nepal	2004	CBH	11800	72	0	Central Bureau of Statistics (Nepal), World Bank. Nepal Living Standards Measurement Survey 2003-2004. Kathmandu, Nepal: Central Bureau of Statistics (Nepal).	46480
Nepal	2006	CBH	26394	0	260	Macro International, Inc, Ministry of Health and Population (Nepal), New ERA. Nepal Demographic and Health Survey 2006. Fairfax, United States: ICF International.	20462
Nepal	2011	CBH	26615	0	289	ICF Macro, Ministry of Health and Population (Nepal), New ERA. Nepal Demographic and Health Survey 2011. Fairfax, United States: ICF International.	21240
Nepal	2014	CBH	28647	0	510	Central Bureau of Statistics (Nepal), United Nations Children's Fund (UNICEF). Nepal Multiple Indicator Cluster Survey 2014. New York, United States: United Nations Children's Fund (UNICEF), 2015.	162317
Nepal	2017	CBH	26028	73	383	ICF International, Ministry of Health (Nepal), New ERA. Nepal Demographic and Health Survey 2016-2017. Fairfax, United States: ICF International, 2017.	286782
Nicaragua	2001	SBH	13963	116	0	National Institute of Statistics and Censuses (Nicaragua), World Bank. Nicaragua Living Standards Measurement Survey 2001.	9422
Nicaragua	2001	CBH	34157	131	0	Macro International, Inc, Ministry of Health (Nicaragua), National Institute of Statistics and Censuses (Nicaragua). Nicaragua Demographic and Health Survey 2001. Fairfax, United States: ICF International.	20487
Nicaragua	2005	SBH	20528	135	0	National Institute of Statistics and Censuses (Nicaragua), World Bank. Nicaragua Living Standards Measurement Survey 2005.	44645

Country	Year	Data Type	Children Born	Polygons	Points	Citation	GHDx ID
Nicaragua	2005	SBH	295730	87	0	Minnesota Population Center, National Institute of Statistics and Censuses (Nicaragua). Nicaragua Population and Housing Census 2005 from the Integrated Public Use Microdata Series, International: [Machine-readable database]. Minneapolis: University of Minnesota, 2012.	56520
Nicaragua	2007	CBH	34055	141	0	Division of Reproductive Health, Centers for Disease Control and Prevention (CDC), National Institute for Development Information (Nicaragua). Nicaragua Reproductive Health Survey 2006-2007. Managua, Nicaragua: National Institute for Development Information (Nicaragua).	9270
Nicaragua	2012	CBH	31815	134	0	Ministry of Health (Nicaragua), National Institute for Development Information (Nicaragua). Nicaragua National Demographic and Health Survey 2011-2012. Managua, Nicaragua: National Institute for Development Information (Nicaragua).	126952
Niger	2006	CBH	34378	8	0	Department of Statistics and National Accounts (Niger), Macro International, Inc. Niger Demographic and Health Survey 2006. Fairfax, United States: ICF International.	20499
Niger	2012	CBH	44183	8	0	ICF International, Ministry of Public Health (Niger), National Institute of Statistics (Niger). Niger Demographic and Health Survey 2012. Fairfax, United States: ICF International.	74393
Nigeria	2003	CBH	23038	0	360	Department for International Development (DFiD) (United Kingdom), National Population Commission of Nigeria, ORC Macro, United Nations Children’s Fund (UNICEF), United Nations Population Fund (UNFPA). Nigeria Demographic and Health Survey 2003. Fairfax, United States: ICF International.	20567

Country	Year	Data Type	Children Born	Polygons	Points	Citation	GHDx ID
Nigeria	2004	SBH	53051	37	0	Federal Office of Statistics (Nigeria). Nigeria Living Standards Survey 2003-2004.	25006
Nigeria	2007	SBH	68689	37	0	United Nations Children's Fund (UNICEF), National Bureau of Statistics (Nigeria). Nigeria Multiple Indicator Cluster Survey 2007. New York, United States: United Nations Children's Fund (UNICEF).	9516
Nigeria	2008	CBH	104808	0	886	Macro International, Inc, National Population Commission of Nigeria. Nigeria Demographic and Health Survey 2008. Fairfax, United States: ICF International, 2009.	21433
Nigeria	2008	SBH	55508	34	0	Central Bank of Nigeria, National Bureau of Statistics (Nigeria), Nigerian Communications Commission (NCC). Nigeria General Household Survey 2008.	24915
Nigeria	2008	SBH	16333	37	0	Federal Ministry of Health (Nigeria), National Agency for the Control of AIDS (NACA) (Nigeria), National Bureau of Statistics (Nigeria), National Population Commission (NPC), Society for Family Health (Nigeria), University College Hospital, Ibadan. Nigeria National HIV/AIDS and Reproductive Health Survey 2007.	325046
Nigeria	2010	CBH	19644	0	239	ICF Macro, National Malaria Control Programme (Nigeria), National Population Commission of Nigeria. Nigeria Malaria Indicator Survey 2010. Fairfax, United States: ICF International.	30991
Nigeria	2011	SBH	100531	37	0	National Bureau of Statistics (Nigeria), United Nations Children's Fund (UNICEF). Nigeria Multiple Indicator Cluster Survey 2011. New York, United States: United Nations Children's Fund (UNICEF), 2013.	76703

Country	Year	Data Type	Children Born	Polygons	Points	Citation	GHDx ID
Nigeria	2012	SBH	42204	679	0	Expanded Social Marketing Project in Nigeria (ESMPIN), Federal Ministry of Health (Nigeria), Joint United Nations Program on HIV/AIDS (UNAIDS), National Population Commission (NPC), Society for Family Health (Nigeria), University College Hospital, Ibadan, World Health Organization (WHO). Nigeria National HIV/AIDS and Reproductive Health Survey 2012.	324443
Nigeria	2013	CBH	119386	0	889	ICF International, National Population Commission of Nigeria. Nigeria Demographic and Health Survey 2013. Fairfax, United States: ICF International.	77390
Nigeria	2015	SBH	25450	0	322	ICF International, National Bureau of Statistics (Nigeria), National Malaria Control Programme (Nigeria), National Population Commission of Nigeria. Nigeria Malaria Indicator Survey 2015. Fairfax, United States: ICF International, 2016.	218590
Nigeria	2017	CBH	101649	37	0	National Agency for the Control of AIDS (Nigeria), National Bureau of Statistics (Nigeria), National Primary Health Care Development Agency (NPHCDA) (Nigeria), United Nations Children's Fund (UNICEF). Nigeria Multiple Indicator Cluster Survey with National Immunization Coverage Survey Supplement 2016-2017. New York, United States: United Nations Children's Fund (UNICEF), 2018.	218613
Pakistan	2002	SBH	68782	7	0	Federal Bureau of Statistics (Pakistan). Pakistan Integrated Household Survey 2001-2002. Islamabad, Pakistan: Federal Bureau of Statistics (Pakistan).	9720
Pakistan	2006	SBH	60656	4	0	Federal Bureau of Statistics (Pakistan). Pakistan Social and Living Standards Measurement Survey 2005-2006. Islamabad, Pakistan: Federal Bureau of Statistics (Pakistan).	24818

Country	Year	Data Type	Children Born	Polygons	Points	Citation	GHDx ID
Pakistan	2007	CBH	39049	0	955	Macro International, Inc, National Institute of Population Studies (Pakistan). Pakistan Demographic and Health Survey 2006-2007. Fairfax, United States: ICF International.	20595
Pakistan	2008	SBH	56298	4	0	Federal Bureau of Statistics (Pakistan). Pakistan Social and Living Standards Measurement Survey 2007-2008. Islamabad, Pakistan: Federal Bureau of Statistics (Pakistan).	30634
Pakistan	2010	SBH	45966	31	0	Government of Balochistan (Pakistan), United Nations Children's Fund (UNICEF). Pakistan - Balochistan Multiple Indicator Cluster Survey 2010. New York, United States: United Nations Children's Fund (UNICEF).	60942
Pakistan	2011	SBH	297998	145	0	Bureau of Statistics (Punjab), United Nations Children's Fund (UNICEF), United Nations Development Programme (UNDP). Pakistan - Punjab Multiple Indicator Cluster Survey 2011. New York, United States: United Nations Children's Fund (UNICEF), 2013.	104236
Pakistan	2013	CBH	50238	6	0	ICF International, National Institute of Population Studies (Pakistan), Pakistan Bureau of Statistics. Pakistan Demographic and Health Survey 2012-2013. Fairfax, United States: ICF International.	77521
Pakistan	2014	SBH	68680	28	0	Bureau of Statistics, Planning and Development Department, Government of Sindh (Pakistan), Global Alliance for Improved Nutrition (GAIN), Pakistan Council of Research in Water Resource (PCRWR), United Nations Children's Fund (UNICEF). Pakistan - Sindh Multiple Indicator Cluster Survey 2014. Fairfax, United States: ICF International, 2016.	232763

Country	Year	Data Type	Children Born	Polygons	Points	Citation	GHDx ID
Pakistan	2014	SBH	121066	36	0	Bureau of Statistics (Punjab), United Nations Children's Fund (UNICEF). Pakistan - Punjab Multiple Indicator Cluster Survey 2014. New York, United States: United Nations Children's Fund (UNICEF), 2015.	236266
Pakistan	2018	CBH	50495	0	561	ICF International, Ministry of National Health Services, Regulations & Coordination (Pakistan), National Institute of Population Studies (Pakistan). Pakistan Demographic and Health Survey 2017-2018. Fairfax, United States: ICF International, 2018.	286783
Palestine	2000	CBH	26074	2	0	Ministry of Health (Palestine), Palestinian Central Bureau of Statistics, United Nations Children's Fund (UNICEF), United Nations Population Fund (UNFPA). Palestine - West Bank and Gaza Strip Multiple Indicator Cluster Survey 2000. Ramallah, Palestine: Palestinian Central Bureau of Statistics.	10001
Palestine	2004	CBH	22478	2	0	Palestinian Central Bureau of Statistics. Palestine Demographic and Health Survey 2004.	20596
Palestine	2007	CBH	51635	16	0	League of Arab States, Palestinian Central Bureau of Statistics, United Nations Children's Fund (UNICEF). Palestine Family Health Survey 2006-2007.	9999
Palestine	2010	CBH	55823	16	0	Ministry of Health (Palestine), Palestinian Central Bureau of Statistics, United Nations Children's Fund (UNICEF), United Nations Population Fund (UNFPA). Palestine Multiple Indicator Cluster Survey 2010. New York, United States: United Nations Children's Fund (UNICEF), 2014.	125591

Country	Year	Data Type	Children Born	Polygons	Points	Citation	GHDx ID
Palestine	2014	CBH	31817	16	0	Ministry of Health (Palestine), Palestinian Central Bureau of Statistics, United Nations Children’s Fund (UNICEF). Palestine Multiple Indicator Cluster Survey 2014. New York, United States: United Nations Children’s Fund (UNICEF), 2015.	161590
Panama	2003	SBH	13641	12	0	Census and Statistics Directorate (Panama), Ministry of Economy and Finance (Panama), World Bank. Panama Living Standard Measurement Survey 2003. Washington DC, United States: World Bank.	10224
Panama	2008	SBH	13333	12	0	Census and Statistics Directorate (Panama), Ministry of Economy and Finance (Panama), World Bank. Panama Living Standard Measurement Survey 2008. Washington DC, United States: World Bank.	46517
Panama	2010	SBH	156544	39	0	Minnesota Population Center, National Institute of Statistics and Census (Panama). Panama Population and Housing Census 2010 from the Integrated Public Use Microdata Series, International: [Machine-readable database]. Minneapolis: University of Minnesota, 2013.	106529
Papua New Guinea	2006	SBH	48396	99	0	National Statistical Office (Papua New Guinea), National Statistics Office (Philippines). Papua New Guinea Demographic and Health Survey 2006-2007.	44870
Paraguay	2002	SBH	272785	76	0	Minnesota Population Center, Paraguay Department of Statistics, Surveys and Censuses. Paraguay Population and Housing Census 2002 from the Integrated Public Use Microdata Series, International: [Machine-readable database]. Minneapolis: University of Minnesota, 2015.	227167

Country	Year	Data Type	Children Born	Polygons	Points	Citation	GHDx ID
Paraguay	2004	CBH	14799	16	0	Division of Reproductive Health-Centers for Disease Control and Prevention (CDC). (2005): Paraguay Reproductive Health Survey 2004. Asuncion, Paraguay, Paraguayan Center for Population Studies (CEPEP).	10370
Paraguay	2008	CBH	11368	16	0	Paraguay Center for Population Studies (CEPEP). Paraguay Reproductive Health Survey 2008. Asuncion, Paraguay: Paraguayan Center for Population Studies (CEPEP).	27525
Paraguay	2016	CBH	14355	9	0	General Directorate of Statistics, Surveys and Censuses (Paraguay), Ministry of Public Health and Social Welfare (Paraguay), United Nations Children's Fund (UNICEF). Paraguay Multiple Indicator Cluster Survey 2016. New York, United States: United Nations Children's Fund (UNICEF), 2017.	324470
Peru	2000	CBH	65453	0	1409	Macro International, Inc, National Institute of Statistics (Peru). Peru Demographic and Health Survey 2000. Fairfax, United States: ICF International.	20649
Peru	2007	SBH	1255851	175	0	National Institute of Statistics and Informatics (INEI) (Peru), Minnesota Population Center. Peru National Population and Housing Census 2007 from the Integrated Public Use Microdata Series, International: [Machine-readable database]. Minneapolis: University of Minnesota.	41267
Peru	2008	CBH	89220	0	1408	Ministry of Economy and Finance (Peru), National Institute of Statistics and Informatics (Peru), ORC Macro. Peru Continuous Demographic and Health Survey 2003-2008. Fairfax, United States: ICF International.	275090

Country	Year	Data Type	Children Born	Polygons	Points	Citation	GHDx ID
Peru	2009	CBH	50084	0	1132	National Institute of Statistics and Informatics (Peru), ORC Macro. Peru Continuous Demographic and Health Survey 2009. Fairfax, United States: ICF International.	270404
Peru	2010	CBH	46780	24	0	National Institute of Statistics and Informatics (Peru). Peru Continuous Demographic and Health Survey 2010. Fairfax, United States: ICF International.	270469
Peru	2011	CBH	46194	24	0	Macro International, Inc, National Institute of Statistics and Informatics (Peru). Peru Continuous Demographic and Health Survey 2011. Fairfax, United States: ICF International.	270470
Peru	2012	CBH	47261	24	0	Macro International, Inc, National Institute of Statistics and Informatics (Peru). Peru Continuous Demographic and Health Survey 2012. Fairfax, United States: ICF International.	270471
Peru	2013	CBH	44725	24	0	ICF International, National Institute of Statistics and Informatics (Peru). Peru Continuous Demographic and Health Survey 2013. Lima, Peru: National Institute of Statistics and Informatics (Peru), 2014.	146860
Peru	2014	CBH	47633	24	0	ICF International, Ministry of Health (Peru), National Institute of Statistics and Informatics (Peru), National Police of Peru (PNP). Peru Continuous Demographic and Health Survey 2014. Fairfax, United States: ICF International, 2015.	209930
Peru	2015	CBH	74559	0	1617	National Institute of Statistics and Informatics (Peru). Peru Demographic and Family Health Survey 2015. Lima, Peru: National Institute of Statistics and Informatics (Peru), 2017.	303663

Country	Year	Data Type	Children Born	Polygons	Points	Citation	GHDx ID
Peru	2016	CBH	67481	0	1976	National Institute of Statistics and Informatics (Peru). Peru Demographic and Family Health Survey 2016. Lima, Peru: National Institute of Statistics and Informatics (Peru), 2017.	303664
Peru	2017	SBH	64444	0	1933	National Center for Food and Nutrition, National Institute of Health (Peru), National Institute of Statistics and Informatics (Peru), National Police of Peru (PNP). Peru Demographic and Family Health Survey 2017. Lima, Peru: National Institute of Statistics and Informatics (Peru).	358824
Philippines	2003	CBH	30443	0	815	Macro International, Inc, National Statistics Office (Philippines). Philippines Demographic and Health Survey 2003. Fairfax, United States: ICF International.	20699
Philippines	2008	CBH	28518	0	784	Macro International, Inc, National Statistics Office (Philippines). Philippines Demographic and Health Survey 2008. Fairfax, United States: ICF International, 2010.	21421
Philippines	2010	SBH	4151720	1308	0	Philippines Statistics Authority, Minnesota Population Center. Philippines Population and Housing Census 2010 from the Integrated Public Use Microdata Series, International. Minneapolis, MN: IPUMS, 2018. https://doi.org/10.18128/D020.V7.1	367607
Philippines	2011	CBH	99962	82	0	ICF International, National Statistics Office (Philippines). Philippines Demographic and Health Survey 2011.	135803
Philippines	2013	CBH	31680	17	0	ICF International, Philippines Statistics Authority. Philippines Demographic and Health Survey 2013. Fairfax, United States: ICF International, 2014.	142943

Country	Year	Data Type	Children Born	Polygons	Points	Citation	GHDx ID
Philippines	2017	CBH	10551	0	1206	ICF International, Philippines Statistics Authority, United States Agency for International Development (USAID). Philippines Demographic and Health Survey 2017. Fairfax, United States: ICF International, 2018.	337877
Republic of the Congo	2005	CBH	16687	12	0	Macro International, Inc, National Center for Statistics and Economic Studies (Congo, Rep.). Congo Demographic and Health Survey 2005. Fairfax, United States: ICF International.	19391
Republic of the Congo	2009	SBH	18574	12	0	ICF Macro, National Center for Statistics and Economic Studies (Congo, Rep.). Congo AIDS Indicator Survey 2009. Fairfax, United States: ICF International.	3133
Republic of the Congo	2012	CBH	31948	12	0	ICF International, Ministry of Health (Congo, Rep.), National Center for Statistics and Economic Studies (Congo, Rep.). Congo Demographic and Health Survey 2011-2012. Fairfax, United States: ICF International.	56151
Republic of the Congo	2014	CBH	31640	11	0	National Institute of Statistics (INS) (Congo, Rep.), United Nations Children’s Fund (UNICEF). Congo Multiple Indicator Cluster Survey 2014-2015. New York, United States: United Nations Children’s Fund (UNICEF), 2018.	234733
Rwanda	2000	CBH	27602	12	0	Macro International, Inc, National Office of Population (Rwanda). Rwanda Demographic and Health Survey 2000. Fairfax, United States: ICF International.	20722
Rwanda	2002	SBH	518181	104	0	National Census Commission (Rwanda), Minnesota Population Center. Rwanda Population and Housing Census 2002 from the Integrated Public Use Microdata Series, International: [Machine-readable database]. Minneapolis: University of Minnesota.	42432

Country	Year	Data Type	Children Born	Polygons	Points	Citation	GHDx ID
Rwanda	2005	CBH	30072	0	456	Macro International, Inc, National Institute of Statistics of Rwanda. Rwanda Demographic and Health Survey 2005. Fairfax, United States: ICF International.	20740
Rwanda	2006	SBH	21031	30	0	National Institute of Statistics of Rwanda (NISR), Oxford Policy Management. Rwanda Integrated Living Conditions Survey 2005-2006. Kigali, Rwanda: National Institute of Statistics of Rwanda (NISR).	11324
Rwanda	2008	CBH	18421	0	246	Macro International, Inc, Ministry of Health (Rwanda), National Institute of Statistics of Rwanda. Rwanda Interim Demographic and Health Survey 2007-2008. Fairfax, United States: ICF International.	21222
Rwanda	2011	CBH	32639	0	492	ICF Macro, Ministry of Health (Rwanda), National Institute of Statistics of Rwanda. Rwanda Demographic and Health Survey 2010-2011. Fairfax, United States: ICF International.	56040
Rwanda	2012	SBH	550802	30	0	National Institute of Statistics of Rwanda. Rwanda Population and Housing Census 2012. Kigali, Rwanda: National Institute of Statistics of Rwanda, 2015.	218773
Rwanda	2012	SBH	552005	29	0	National Institute of Statistics (Rwanda), Minnesota Population Center. Rwanda Population and Housing Census 2012 from the Integrated Public Use Microdata Series, International. Minneapolis, MN: IPUMS, 2018. https://doi.org/10.18128/D020.V7.1	367645
Rwanda	2013	SBH	11726	5	0	ICF International, Ministry of Health (Rwanda). Rwanda Malaria Indicator Survey 2013. Fairfax, United States: ICF International, 2014.	77391

Country	Year	Data Type	Children Born	Polygons	Points	Citation	GHDx ID
Rwanda	2015	CBH	30058	0	492	ICF International, Ministry of Health (Rwanda), National Institute of Statistics of Rwanda. Rwanda Demographic and Health Survey 2014-2015. Fairfax, United States: ICF International, 2016.	157063
Rwanda	2017	SBH	11349	30	0	ICF International, Ministry of Health (Rwanda), Rwanda Biomedical Center. Rwanda Malaria Indicator Survey 2017. Fairfax, United States: ICF International, 2018.	350836
Sao Tome and Principe	2009	CBH	7620	7	0	ICF Macro, Ministry of Health (Sao Tome and Principe), National Institute of Statistics (Sao Tome and Principe). Sao Tome and Principe Demographic and Health Survey 2008-2009. Fairfax, United States: ICF International.	26866
Sao Tome and Principe	2014	CBH	7492	7	0	Global Fund to Fight Aids Tuberculosis and Malaria (GFATM), ICF International, National Center for Endemic Diseases (CNE) (Sao Tome and Principe), National Institute of Statistics (Sao Tome and Principe), United Nations Children's Fund (UNICEF), United Nations Development Programme (UNDP). Sao Tome and Principe Multiple Indicator Cluster Survey 2014. New York, United States: United Nations Children's Fund (UNICEF), 2016.	214640
Senegal	2002	SBH	571816	34	0	Directorate of Forecasting and Statistics (Senegal), Minnesota Population Center. Senegal General Population and Housing Census 2002 from the Integrated Public Use Microdata Series, International: [Machine-readable database]. Minneapolis: University of Minnesota.	43142
Senegal	2005	CBH	39895	0	366	Ministry of Health and Prevention (Senegal), Research Center for Human Development (Senegal). Senegal Demographic and Health Survey 2005. Fairfax, United States: ICF International.	26855

Country	Year	Data Type	Children Born	Polygons	Points	Citation	GHDx ID
Senegal	2006	SBH	18520	11	0	Macro International, Inc, Research Center for Human Development (Senegal). Senegal Malaria Indicator Survey 2006. Fairfax, United States: ICF International.	11516
Senegal	2009	CBH	53608	0	319	Macro International, Inc, Research Center for Human Development (Senegal). Senegal Malaria Indicator Survey 2008-2009. Fairfax, United States: ICF International.	11540
Senegal	2011	CBH	42510	0	385	Center for Research in Human Development (CRDH), Cheikh Anta Diop University, Hospital Aristide Le Dantec, ICF Macro, National Agency of Statistics and Demography (Senegal). Senegal Demographic and Health Survey 2010-2011. Fairfax, United States: ICF International.	56063
Senegal	2013	CBH	22563	0	200	ICF International, Ministry of Health and Social Action (Senegal), National Agency of Statistics and Demography (Senegal). Senegal Continuous Demographic and Health Survey 2012-2013. Fairfax, United States: ICF International.	111432
Senegal	2014	CBH	22365	14	0	Cheikh Anta Diop University, ICF International, National Agency of Statistics and Demography (Senegal). Senegal Continuous Demographic and Health Survey 2014. Fairfax, United States: ICF International.	191270
Senegal	2015	CBH	23250	0	214	Cheikh Anta Diop University, ICF International, National Agency of Statistics and Demography (Senegal). Senegal Continuous Demographic and Health Survey 2015. Fairfax, United States: ICF International, 2016.	218592

Country	Year	Data Type	Children Born	Polygons	Points	Citation	GHDx ID
Senegal	2015	CBH	16532	4	0	National Agency of Statistics and Demography (Senegal), United Nations Children's Fund (UNICEF). Senegal - Dakar Urban Multiple Indicator Cluster Survey 2015-2016. New York, United States: United Nations Children's Fund (UNICEF), 2018.	287639
Senegal	2016	CBH	22740	0	214	ICF International, Ministry of Health and Social Action (Senegal), National Agency of Statistics and Demography (Senegal). Senegal Continuous Demographic and Health Survey 2016. Fairfax, United States: ICF International, 2017.	286772
Senegal	2017	CBH	42944	14	0	ICF International, Ministry of Health and Social Action (Senegal), National Agency of Statistics and Demography (Senegal), Unit for the Fight Against Malnutrition (Senegal). Senegal Continuous Demographic and Health Survey 2017. Fairfax, United States: ICF International, 2018.	353526
Sierra Leone	2004	SBH	344320	100	0	Statistics Sierra Leone and Minnesota Population Center. Sierra Leone Population and Housing Census 2004 from the Integrated Public Use Microdata Series, International: [Machine-readable database]. Minneapolis: University of Minnesota, 2011.	11661
Sierra Leone	2005	SBH	28284	14	0	United Nations Children's Fund (UNICEF), Statistics Sierra Leone. Sierra Leone Multiple Indicator Cluster Survey 2005. New York, United States: United Nations Children's Fund (UNICEF).	11649
Sierra Leone	2008	CBH	21136	0	350	Macro International, Inc, Statistics Sierra Leone. Sierra Leone Demographic and Health Survey 2008. Fairfax, United States: ICF International.	21258

Country	Year	Data Type	Children Born	Polygons	Points	Citation	GHDx ID
Sierra Leone	2010	SBH	39257	14	0	Statistics Sierra Leone, United Nations Children's Fund (UNICEF). Sierra Leone Multiple Indicator Cluster Survey 2010. New York, United States: United Nations Children's Fund (UNICEF).	76700
Sierra Leone	2013	CBH	47392	0	435	ICF International, Ministry of Health and Sanitation (Sierra Leone), Statistics Sierra Leone. Sierra Leone Demographic and Health Survey 2013. Fairfax, United States: ICF International, 2014.	131467
Sierra Leone	2016	SBH	28463	14	0	Catholic Relief Services (CRS), College of Medicine and Allied Health Sciences, University of Sierra Leone (COMAHS), ICF International, National Malaria Control Programme (Sierra Leone), Roll Back Malaria Partnership, Statistics Sierra Leone. Sierra Leone Malaria Indicator Survey 2016. Fairfax, United States: ICF International, 2017.	286773
Sierra Leone	2017	CBH	42070	14	0	Statistics Sierra Leone, United Nations Children's Fund (UNICEF). Sierra Leone Multiple Indicator Cluster Survey 2017. New York, United States: United Nations Children's Fund (UNICEF), 2018.	218619
Somalia	2006	CBH	20034	18	0	Pan Arab Project for Family Health (PAPFAM), United Nations Children's Fund (UNICEF). Somalia Multiple Indicator Cluster Survey 2006. New York, United States: United Nations Children's Fund (UNICEF).	11774
Somalia	2011	CBH	16700	0	244	Ministry of National Planning and Development (Somaliland), United Nations Children's Fund (UNICEF). Somalia - Somaliland Multiple Indicator Cluster Survey 2011. New York, United States: United Nations Children's Fund (UNICEF), 2015.	91507

Country	Year	Data Type	Children Born	Polygons	Points	Citation	GHDx ID
South Africa	2001	SBH	1799625	277	0	Statistics South Africa, Minnesota Population Center. South Africa Census 2001 from the Integrated Public Use Microdata Series, International: [Machine-readable database]. Minneapolis: University of Minnesota.	43152
South Africa	2002	SBH	43580	9	0	Statistics South Africa. South Africa General Household Survey 2002. Pretoria, South Africa: Statistics South Africa.	115481
South Africa	2007	SBH	469555	218	0	Statistics South Africa, Minnesota Population Center. South Africa Community Survey 2007 from the Integrated Public Use Microdata Series, International: [Machine-readable database]. Minneapolis: University of Minnesota.	43158
South Africa	2016	CBH	14144	0	714	Department of Health (South Africa), ICF International, South African Medical Research Council, Statistics South Africa. South Africa Demographic and Health Survey 2016. Fairfax, United States: ICF International, 2019.	157064
South Africa	2016	SBH	1247106	212	0	Statistics South Africa. South Africa Community Survey 2016. Pretoria, South Africa: Statistics South Africa, 2016.	280803
South Sudan	2008	SBH	337728	72	0	Minnesota Population Center, Southern Sudan Centre for Census, Statistics and Evaluation. Sudan - South Sudan Population and Housing Census 2008 from the Integrated Public Use Microdata Series, International: [Machine-readable database]. Minneapolis: University of Minnesota, 2013	106548

Country	Year	Data Type	Children Born	Polygons	Points	Citation	GHDx ID
South Sudan	2010	CBH	28987	10	0	Central Bureau of Statistics (Sudan), Federal Ministry of Health (Sudan), Government of Sudan, Ministry of Health (South Sudan), Southern Sudan Centre for Census, Statistics and Evaluation. Sudan - South Multiple Indicator Cluster Survey 2010. New York, United States: United Nations Children's Fund (UNICEF), 2015.	32189
Sudan	2006	CBH	77520	25	0	Ministry of Health (Southern Sudan), Federal Ministry of Health (Sudan), Southern Sudan Centre for Census, Statistics and Evaluation (SSCCSE), Central Bureau of Statistics (Sudan). Sudan Family Health Survey 2006.	24143
Sudan	2008	SBH	2810742	128	0	National Population Census Council (Sudan), Central Bureau of Statistics (Sudan), Southern Sudan Centre for Census, Statistics and Evaluation (SSCCSE), Minnesota Population Center. Sudan Population and Housing Census 2008 from the Integrated Public Use Microdata Series, International: [Machine-readable database]. Minneapolis: University of Minnesota, 2011.	43167
Sudan	2010	CBH	47092	15	0	Central Bureau of Statistics (Sudan), Ministry of Health (South Sudan). Sudan - North Multiple Indicator Cluster Survey 2010. New York, United States: United Nations Children's Fund (UNICEF), 2015.	153643
Sudan	2014	CBH	52245	18	0	Central Bureau of Statistics (Sudan), Federal Ministry of Health (Sudan), United Nations Children's Fund (UNICEF). Sudan Multiple Indicator Cluster Survey 2014. New York, United States: United Nations Children's Fund (UNICEF), 2016.	200617

Country	Year	Data Type	Children Born	Polygons	Points	Citation	GHDx ID
Suriname	2006	SBH	10503	5	0	General Statistical Office (Suriname), United Nations Children's Fund (UNICEF). Suriname Multiple Indicator Cluster Survey 2006. New York, United States: United Nations Children's Fund (UNICEF).	12289
Swaziland	2007	CBH	11410	0	270	Central Statistical Office (Swaziland), Macro International, Inc. Swaziland Demographic and Health Survey 2006-2007. Fairfax, United States: ICF International.	20829
Swaziland	2010	CBH	9805	4	0	Central Statistical Office (Swaziland), United Nations Children's Fund (UNICEF). Swaziland Multiple Indicator Cluster Survey 2010. New York, United States: United Nations Children's Fund (UNICEF).	30325
Swaziland	2014	CBH	9830	4	0	Central Statistical Office (Swaziland), United Nations Children's Fund (UNICEF), United Nations Educational, Scientific and Cultural Organization (UNESCO), United Nations Population Fund (UNFPA). Swaziland Multiple Indicator Cluster Survey 2014. New York, United States: United Nations Children's Fund (UNICEF), 2016.	200707
Syria	2006	SBH	55015	60	0	United Nations Children's Fund (UNICEF), Central Bureau of Statistics (Syria), Ministry of Health (Syria), Pan Arab Project for Family Health (PAPFAM). Syria Multiple Indicator Cluster Survey 2006. New York, United States: United Nations Children's Fund (UNICEF).	12399
Syria	2009	CBH	7365	2	0	Central Bureau of Statistics (Syria), League of Arab States. Syria Family Health Survey 2009.	126911
Tajikistan	2003	CBH	13458	63	4	National State Statistical Agency (Tajikistan), World Bank. Tajikistan Living Standards Measurement Survey 2003.	12489

Country	Year	Data Type	Children Born	Polygons	Points	Citation	GHDx ID
Tajikistan	2005	SBH	23127	5	0	United Nations Children's Fund (UNICEF), State Committee on Statistics of the Republic of Tajikistan. Tajikistan Multiple Indicator Cluster Survey 2005. New York, United States: United Nations Children's Fund (UNICEF).	12608
Tajikistan	2012	CBH	19938	0	342	ICF International, Ministry of Health (Tajikistan), Statistical Agency under the President of the Republic of Tajikistan. Tajikistan Demographic and Health Survey 2012. Fairfax, United States: ICF International, 2013.	74460
Tajikistan	2017	CBH	21985	0	365	ICF International, Statistical Agency under the President of the Republic of Tajikistan. Tajikistan Demographic and Health Survey 2017. Fairfax, United States: ICF International, 2018.	341838
Tanzania	2002	SBH	2664423	129	0	National Bureau of Statistics (Tanzania), Minnesota Population Center. Tanzania Population and Housing Census 2002 from the Integrated Public Use Microdata Series, International: [Machine-readable database]. Minneapolis: University of Minnesota.	43212
Tanzania	2004	SBH	33014	0	345	National Bureau of Statistics (Tanzania), ORC Macro, Tanzania Commission for AIDS (TACAIDS). Tanzania AIDS Indicator Survey 2003-2004. Fairfax, United States: ICF International.	12630
Tanzania	2005	CBH	30557	26	0	Macro International, Inc, National Bureau of Statistics (Tanzania). Tanzania Demographic and Health Survey 2004-2005. Fairfax, United States: ICF International.	20875

Country	Year	Data Type	Children Born	Polygons	Points	Citation	GHDx ID
Tanzania	2008	CBH	27511	0	466	Macro International, Inc, National Bureau of Statistics (Tanzania), Office of Chief Government Statistician (OCGS-Zanzibar), Tanzania Commission for AIDS (TACAIDS), Zanzibar AIDS Commission (ZAC). Tanzania HIV/AIDS and Malaria Indicator Survey 2007-2008. Fairfax, United States: ICF International.	12644
Tanzania	2010	CBH	29777	0	458	ICF Macro, National Bureau of Statistics (Tanzania). Tanzania Demographic and Health Survey 2009-2010. Fairfax, United States: ICF International.	21331
Tanzania	2012	SBH	32522	0	573	ICF International, National Bureau of Statistics (Tanzania), Office of Chief Government Statistician (OCGS-Zanzibar), Tanzania Commission for AIDS (TACAIDS), Zanzibar AIDS Commission (ZAC). Tanzania AIDS Indicator Survey 2011-2012. Fairfax, United States: ICF International, 2013.	77395
Tanzania	2012	SBH	3225395	169	0	National Bureau of Statistics (Tanzania), Minnesota Population Center. Tanzania Population and Housing Census 2012 from the Integrated Public Use Microdata Series, International. Minneapolis: University of Minnesota, 2017.	294725
Tanzania	2016	CBH	37169	0	608	ICF International, Ministry of Health (Zanzibar), Ministry of Health, Community Development, Gender, Elderly and Children (MoHCDEC) (Tanzania), National Bureau of Statistics (Tanzania), Office of Chief Government Statistician (OCGS-Zanzibar). Tanzania Demographic and Health Survey 2015-2016. Fairfax, United States: ICF International, 2016.	218593

Country	Year	Data Type	Children Born	Polygons	Points	Citation	GHDx ID
Tanzania	2017	SBH	29279	29	0	ICF International, Ministry of Health (Zanzibar), Ministry of Health, Community Development, Gender, Elderly and Children (MoHCDEC) (Tanzania), National Bureau of Statistics (Tanzania), Office of Chief Government Statistician (OCGS-Zanzibar). Tanzania Malaria Indicator Survey 2017. Fairfax, United States: ICF International, 2018.	350798
Thailand	2006	SBH	48610	4	0	National Statistical Office (Thailand), United Nations Children's Fund (UNICEF). Thailand Multiple Indicator Cluster Survey 2005-2006. New York, United States: United Nations Children's Fund (UNICEF).	12732
Thailand	2012	SBH	30853	5	0	College of Population Studies, Chulalongkorn University (Thailand), Institute for Population and Social Research, Mahidol University (Thailand), International Health Policy Program (Thailand), Ministry of Education (Thailand), Ministry of Public Health (Thailand), Ministry of Social Development and Human Security (MSDHS) (Thailand), National Health Security Office (Thailand), National Statistical Office (Thailand), Thai Health Promotion Foundation, United Nations Children's Fund (UNICEF). Thailand Multiple Indicator Cluster Survey 2012. New York, United States: United Nations Children's Fund (UNICEF), 2016.	148649
Thailand	2015	SBH	37245	1	0	National Health Security Office (Thailand), National Statistical Office (Thailand), United Nations Children's Fund (UNICEF). Thailand Multiple Indicator Cluster Survey 2015-2016. New York, United States: United Nations Children's Fund (UNICEF), 2018.	296646

Country	Year	Data Type	Children Born	Polygons	Points	Citation	GHDx ID
Thailand	2016	SBH	3448	1	0	National Health Security Office (Thailand), National Statistical Office (Thailand), United Nations Children's Fund (UNICEF). Thailand - Bangkok Small Community Multiple Indicator Cluster Survey 2016. New York, United States: United Nations Children's Fund (UNICEF), 2018.	331377
The Gambia	2006	SBH	27475	37	0	Gambia Bureau of Statistics (GBOS), United Nations Children's Fund (UNICEF). Gambia Multiple Indicator Cluster Survey 2005-2006. New York, United States: United Nations Children's Fund (UNICEF).	3935
The Gambia	2010	SBH	42194	6	0	Gambia Bureau of Statistics (GBOS), United Nations Children's Fund (UNICEF). Gambia Multiple Indicator Cluster Survey 2010. New York, United States: United Nations Children's Fund (UNICEF), 2018.	91506
The Gambia	2013	CBH	26601	37	0	Gambia Bureau of Statistics (GBOS), ICF International, Ministry of Health and Social Welfare (Gambia). Gambia Demographic and Health Survey 2013. Fairfax, United States: ICF International, 2015.	77384
Timor-Leste	2003	CBH	17889	92	287	ACIL Australia Pty Ltd., Australian National University, Ministry of Health (Timor-Leste), National Statistics Directorate (Timor-Leste), University of Newcastle (Australia). Timor-Leste Demographic and Health Survey 2003. Newcastle, Australia: University of Newcastle (Australia).	20888[†]
Timor-Leste	2008	SBH	14113	64	0	National Statistics Directorate (Timor-Leste), World Bank. Timor-Leste Living Standards and Measurement Survey 2007-2008. Washington DC, United States: World Bank.	46682

Country	Year	Data Type	Children Born	Polygons	Points	Citation	GHDx ID
Timor-Leste	2010	CBH	35998	13	0	ICF Macro, Ministry of Finance (Timor-Leste), National Statistics Directorate (Timor-Leste). Timor-Leste Demographic and Health Survey 2009-2010. Fairfax, United States: ICF International.	21274
Timor-Leste	2016	CBH	28682	0	455	ICF International, National Statistics Directorate (Timor-Leste). Timor-Leste Demographic and Health Survey 2016. Fairfax, United States: ICF International, 2018.	286785
Togo	2006	SBH	17832	6	0	Directorate General of Statistics and National Accounting (Togo), United Nations Children's Fund (UNICEF). Togo Multiple Indicator Cluster Survey 2006. New York, United States: United Nations Children's Fund (UNICEF).	12896
Togo	2010	SBH	18954	6	0	Directorate General of Statistics and National Accounting (Togo), United Nations Children's Fund (UNICEF). Togo Multiple Indicator Cluster Survey 2010. New York, United States: United Nations Children's Fund (UNICEF).	40021
Togo	2013	CBH	26264	0	330	Directorate General of Statistics and National Accounts (Togo), ICF International, Ministry of Health (Togo), Ministry of Planning, Development and Zoning (Togo). Togo Demographic and Health Survey 2013-2014. Fairfax, United States: ICF International, 2015.	77515
Togo	2017	SBH	13603	0	171	ICF International, National Institute of Hygiene, Ministry of Health (Togo), National Institute of Statistics and Economic and Demographic Studies (INSEED) (Togo). Togo Malaria Indicator Survey 2017. Fairfax, United States: ICF International, 2018.	359318

Country	Year	Data Type	Children Born	Polygons	Points	Citation	GHDx ID
Trinidad and Tobago	2006	SBH	6551	15	0	Central Statistical Office (Trinidad and Tobago) and United Nations Children's Fund (UNICEF). Trinidad and Tobago Multiple Indicator Cluster Survey 2006. New York, United States: United Nations Children's Fund (UNICEF).	12950
Trinidad and Tobago	2011	SBH	35781	15	0	Central Statistical Office (Trinidad and Tobago), Minnesota Population Center. Trinidad and Tobago Population and Housing Census 2011 from the Integrated Public Use Microdata Series, International [Machine-readable database]. Minneapolis: University of Minnesota, 2017.	294807
Trinidad and Tobago	2011	SBH	5617	5	0	Central Statistical Office (Trinidad and Tobago), Ministry of Social Development and Family Services (Trinidad and Tobago), United Nations Children's Fund (UNICEF). Trinidad and Tobago Multiple Indicator Cluster Survey 2011. New York, United States: United Nations Children's Fund (UNICEF), 2018.	332558
Tunisia	2012	CBH	13569	9	0	Ministry of Regional Development and Planning (Tunisia), National Institute of Statistics (Tunisia), United Nations Children's Fund (UNICEF). Tunisia Multiple Indicator Cluster Survey 2011-2012. New York, United States: United Nations Children's Fund (UNICEF), 2014.	76709
Turkmenistan	2006	SBH	12070	6	0	Ministry of Foreign Affairs (Turkmenistan), Ministry of Health and Medical Industry (Turkmenistan), United Nations Children's Fund (UNICEF). Turkmenistan Multiple Indicator Cluster Survey 2006. New York, United States: United Nations Children's Fund (UNICEF), 2016.	13064

Country	Year	Data Type	Children Born	Polygons	Points	Citation	GHDx ID
Turkmenistan	2016	CBH	12800	6	0	State Committee on Statistics of Turkmenistan, United Nations Children's Fund (UNICEF). Turkmenistan Multiple Indicator Cluster Survey 2015-2016. New York, United States: United Nations Children's Fund (UNICEF), 2017.	264583
Uganda	2001	CBH	23410	0	266	Macro International, Inc, Uganda Bureau of Statistics. Uganda Demographic and Health Survey 2000-2001. Fairfax, United States: ICF International.	20993
Uganda	2002	SBH	1811659	161	0	Uganda Bureau of Statistics, Minnesota Population Center. Uganda Population and Housing Census 2002 from the Integrated Public Use Microdata Series, International: [Machine-readable database]. Minneapolis: University of Minnesota.	43328
Uganda	2005	SBH	36564	55	0	Division of Reproductive Health, Centers for Disease Control and Prevention (CDC), Ministry of Health (Uganda). Uganda AIDS Indicator Survey 2004-2005.	13084
Uganda	2006	CBH	30090	0	336	Macro International, Inc, Uganda Bureau of Statistics. Uganda Demographic and Health Survey 2006. Fairfax, United States: ICF International.	21014
Uganda	2010	CBH	13863	0	170	ICF Macro, Ministry of Health (Uganda), Mulago Hospital, Uganda Bureau of Statistics, United Nations Children's Fund (UNICEF), World Health Organization (WHO). Uganda Malaria Indicator Survey 2009-2010. Fairfax, United States: ICF International.	13109
Uganda	2010	SBH	9416	7	328	Uganda Bureau of Statistics. Uganda Living Standards Measurement Survey - Integrated Survey on Agriculture 2009-2010. Washington DC, United States: World Bank.	81004

Country	Year	Data Type	Children Born	Polygons	Points	Citation	GHDx ID
Uganda	2011	SBH	68086	0	470	Centers for Disease Control and Prevention (CDC), ICF Macro, Ministry of Health (Uganda), Uganda Bureau of Statistics, Uganda Virus Research Institute. Uganda AIDS Indicator Survey 2011. Fairfax, United States: ICF International.	55973
Uganda	2011	CBH	28609	0	400	ICF Macro, Uganda Bureau of Statistics. Uganda Demographic and Health Survey 2011. Fairfax, United States: ICF International.	56021
Uganda	2014	SBH	6353	412	0	Government of the Netherlands, Uganda Bureau of Statistics, World Bank. Uganda Living Standards Measurement Survey - Integrated Survey on Agriculture 2013-2014. Washington DC, United States: World Bank.	264959
Uganda	2015	SBH	17128	0	208	ICF International, National Malaria Control Program, Ministry of Health (Uganda), Uganda Bureau of Statistics. Uganda Malaria Indicator Survey 2014-2015. Fairfax, United States: ICF International.	157065
Uganda	2016	CBH	56868	0	685	ICF International, Uganda Bureau of Statistics. Uganda Demographic and Health Survey 2016. Fairfax, United States: ICF International, 2018.	286780
Uzbekistan	2002	CBH	11607	0	218	Analytical and Information Center of the Ministry of Health of Uzbekistan, Macro International, Inc, Ministry of Macroeconomics and Statistics (Uzbekistan). Uzbekistan Special Demographic and Health Survey 2002. Fairfax, United States: ICF International.	21039

Country	Year	Data Type	Children Born	Polygons	Points	Citation	GHDx ID
Uzbekistan	2006	SBH	26751	6	0	United Nations Children's Fund (UNICEF), State Committee of the Republic of Uzbekistan on Statistics. Uzbekistan Multiple Indicator Cluster Survey 2006. New York, United States: United Nations Children's Fund (UNICEF).	13445
Venezuela	2001	SBH	1176917	237	0	National Institute of Statistics (Venezuela), Minnesota Population Center. Venezuela Population and Housing Census 2002 from the Integrated Public Use Microdata Series, International: [Machine-readable database]. Minneapolis: University of Minnesota.	43412
Vietnam	2002	CBH	14383	41	0	General Statistics Office (Vietnam), Macro International, Inc. Vietnam Demographic and Health Survey 2002. Fairfax, United States: ICF International.	21058
Vietnam	2005	SBH	20964	64	0	General Statistics Office (Vietnam), National Institute of Hygiene and Epidemiology (Viet Nam), ORC Macro. Vietnam AIDS Indicator Survey 2005. Fairfax, United States: ICF International.	13544
Vietnam	2006	SBH	16447	8	0	General Statistics Office (Vietnam), United Nations Children's Fund (UNICEF). Vietnam Multiple Indicator Cluster Survey 2006. New York, United States: United Nations Children's Fund (UNICEF).	13719
Vietnam	2009	SBH	6004427	673	0	General Statistics Office (Viet Nam), Minnesota Population Center. Viet Nam Population and Housing Census 2009 from the Integrated Public Use Microdata Series, International: [Machine-readable database]. Minneapolis: University of Minnesota.	43726

Country	Year	Data Type	Children Born	Polygons	Points	Citation	GHDx ID
Vietnam	2011	SBH	18127	6	0	General Statistics Office (Vietnam), United Nations Children's Fund (UNICEF). Vietnam Multiple Indicator Cluster Survey 2010-2011. New York, United States: United Nations Children's Fund (UNICEF).	57999
Vietnam	2014	CBH	15479	6	0	General Statistics Office (Vietnam), United Nations Children's Fund (UNICEF). Vietnam Multiple Indicator Cluster Survey 2013-2014. New York, United States: United Nations Children's Fund (UNICEF), 2015.	152735
Yemen	2003	SBH	54378	20	0	Central Statistical Organization (Yemen), League of Arab States, Ministry of Public Health and Population (Yemen), Pan Arab Project for Family Health (PAPFAM). Yemen Family Health Survey 2003.	13795
Yemen	2006	CBH	17213	21	0	Ministry of Health (Yemen) and United Nations Children's Fund (UNICEF). Yemen Multiple Indicator Cluster Survey 2006. New York, United States: United Nations Children's Fund (UNICEF).	13816
Yemen	2013	CBH	64602	21	0	Central Statistical Organization (Yemen), ICF International, Ministry of Public Health and Population (Yemen). Yemen Demographic and Health Survey 2013. Fairfax, United States: ICF International.	112500
Yemen	2013	SBH	60675	19	0	Ministry of Planning and International Cooperation (Yemen), International Policy Center for Inclusive Growth, Interaction in Development (Yemen), UNICEF Yemen. Yemen National Social Protection Monitoring Survey 2012-2013.	249499

Country	Year	Data Type	Children Born	Polygons	Points	Citation	GHDx ID
Zambia	2002	CBH	23805	9	0	Central Board of Health (Zambia), Central Statistical Office (Zambia), Macro International, Inc. Zambia Demographic and Health Survey 2001-2002. Fairfax, United States: ICF International.	21102
Zambia	2007	CBH	21366	0	319	Central Statistical Office (Zambia), Macro International, Inc. Zambia Demographic and Health Survey 2007. Fairfax, United States: ICF International.	21117
Zambia	2010	SBH	768988	150	0	Central Statistical Office (Zambia), Minnesota Population Center. Zambia Census 2010 from the Integrated Public Use Microdata Series, International: [Machine-readable database]. Minneapolis: University of Minnesota.	151326
Zambia	2014	CBH	49207	0	719	Central Statistical Office (Zambia), ICF International, Ministry of Health (Zambia), Tropical Diseases Research Centre, University Teaching Hospital (Zambia), University of Zambia. Zambia Demographic and Health Survey 2013-2014. Fairfax, United States: ICF International.	77516
Zimbabwe	2006	CBH	19489	0	396	Central Statistical Office (Zimbabwe), Macro International, Inc. Zimbabwe Demographic and Health Survey 2005-2006. Calverton, United States: Macro International, Inc.	21163
Zimbabwe	2009	CBH	23716	10	0	Central Statistical Office (Zimbabwe). Zimbabwe Multiple Indicator Monitoring Survey 2009. New York, United States: United Nations Children’s Fund (UNICEF).	35493
Zimbabwe	2011	CBH	19279	0	393	ICF Macro, Zimbabwe National Statistics Agency. Zimbabwe Demographic and Health Survey 2010-2011. Calverton, United States: ICF Macro, 2012.	55992

Country	Year	Data Type	Children Born	Polygons	Points	Citation	GHDx ID
Zimbabwe	2012	SBH	312920	88	0	National Statistical Agency (Zimbabwe), Minnesota Population Center. Zimbabwe Population Census 2012 from the Integrated Public Use Microdata Series, International. Minneapolis, MN: IPUMS, 2018. https://doi.org/10.18128/D020.V7.1	367747
Zimbabwe	2014	CBH	32285	10	0	United Nations Children's Fund (UNICEF), Zimbabwe National Statistics Agency. Zimbabwe Multiple Indicator Cluster Survey 2014. New York, United States: United Nations Children's Fund (UNICEF), 2015.	152720
Zimbabwe	2015	CBH	20791	0	400	ICF International, National Microbiology Reference Laboratory, Harare Central Hospital (NMRL) (Zimbabwe), Zimbabwe National Statistics Agency. Zimbabwe Demographic and Health Survey 2015. Fairfax, United States: ICF International, 2016.	157066

Supplementary Table 8.2: Data sources excluded from analysis, with rationale

Country	Year	Data Type	Reason	Citation	GHDx ID
Bangladesh	2010-2013	SBH	Excluded for multiple visits to same household	Fogarty International Center, National Institutes of Health (NIH), Foundation for the National Institutes of Health (FNIH), International Centre for Diarrhoeal Disease Research, Bangladesh (ICDDR,B). Bangladesh - Dhaka Malnutrition and Enteric Disease Study 2009-2014.	261683
Bhutan	2005	SBH	Unable to geomatch	Office of the Census Commissioner (Bhutan). Bhutan Population and Housing Census 2005. Thimphu, Bhutan: Office of the Census Commissioner (Bhutan), 2006.	1175
Burkina Faso	2008	CBH	Only has CBH for <5	Global Fund to Fight Aids Tuberculosis and Malaria (GFATM). Burkina Faso Global Fund Household Health Coverage Survey 2008.	26642
Burkina Faso	2015	SBH	Excluded pma2020 for inflated mortality and high missingness in reported child deaths	Burkina Faso Institut national de la statistique et de la demographie (National Institute of Statistics and Demography), and The Bill & Melinda Gates Institute for Population and Reproductive Health at The Johns Hopkins Bloomberg School of Public Health. Performance Monitoring and Accountability 2020 (PMA2020) Survey round 2, PMA2015/Burkina Faso-R2. 2015. Ouagadougou, Burkina Faso and Baltimore, Maryland, USA.	257045
Burkina Faso	2016	SBH	Excluded pma2020 for inflated mortality and high missingness in reported child deaths	Burkina Faso Institut national de la statistique et de la demographie (National Institute of Statistics and Demography), and The Bill & Melinda Gates Institute for Population and Reproductive Health at The Johns Hopkins Bloomberg School of Public Health. Performance Monitoring and Accountability 2020 (PMA2020) Survey round 3, PMA2016/Burkina Faso-R3. 2016. Ouagadougou, Burkina Faso and Baltimore, Maryland, USA.	285993

Country	Year	Data Type	Reason	Citation	GHDx ID
Burkina Faso	2016-2017	SBH	Excluded pma2020 for inflated mortality and high missingness in reported child deaths	Burkina Faso Institut national de la statistique et de la demographie (National Institute of Statistics and Demography), and The Bill & Melinda Gates Institute for Population and Reproductive Health at The Johns Hopkins Bloomberg School of Public Health. Performance Monitoring and Accountability 2020 (PMA2020) Survey round 4, PMA2016/Burkina Faso-R4. 2016. Ouagadougou, Burkina Faso and Baltimore, Maryland, USA‹.	307751
Burkina Faso	2017-2018	SBH	Excluded pma2020 for inflated mortality and high missingness in reported child deaths	Burkina Faso Institut national de la statistique et de la demographie (National Institute of Statistics and Demography), and The Bill & Melinda Gates Institute for Population and Reproductive Health at The Johns Hopkins Bloomberg School of Public Health. Performance Monitoring and Accountability 2020 (PMA2020) Survey round 5, PMA2017/Burkina Faso-R5. 2017. Ouagadougou, Burkina Faso and Baltimore, Maryland, USAâ‹.	375719
Cambodia	2003-2005	SBH	Excluded due to high number missing interview dates	National Institute of Statistics (Cambodia), Statistics Sweden. Cambodia Socio-Economic Survey 2003-2005. Phnom Penh, Cambodia: National Institute of Statistics (Cambodia).	30963
Cambodia	2004	SBH	These age ranges dont match training data for SBH-CBH model	Institute for Social Research, University of Michigan. Cambodia Elderly Survey 2004. Ann Arbor, United States: Institute for Social Research, University of Michigan.	135505
Cambodia	2008	CBH	CBH has unreasonably low mortality	National Institute of Statistics (Cambodia). Cambodia Anthropometric Survey 2008 - National Institute of Statistics. Phnom Penh, Cambodia: National Institute of Statistics (Cambodia), 2011.	135773

Country	Year	Data Type	Reason	Citation	GHDx ID
Cameroon	2001	SBH	Low N and unrealistic age trend of CEB/CED	National Institute of Statistics (Cameroon), Directorate of Statistics and National Accounts, Ministry of Economics and Finance (Cameroon), AFRISTAT. Cameroon Household Survey 2001. Yaounde, Cameroon: National Institute of Statistics (Cameroon).	2039
Colombia	2008	SBH	Need age of woman to use SBH	National Administrative Department of Statistics (Colombia). Colombia National Quality of Life Survey 2008. BogotÃ¡, Colombia: National Administrative Department of Statistics (Colombia).	68235
Cote d'Ivoire	2006	SBH	Poor quality dataset, CEB values negative	United Nations Children’s Fund (UNICEF), National Institute of Statistics (Cote d'Ivoire). Cote d'Ivoire Multiple Indicator Cluster Survey 2006. New York, United States: United Nations Children’s Fund (UNICEF).	26433
Cote d'Ivoire	2017	SBH	Excluded pma2020 for inflated mortality and high missingness in reported child deaths	Institut National de la Statistique de la Cote d'Ivoire (INS-Cote d'�™Ivoire), La Direction de Coordination du Programme National de Sante de la Mare et de l'Enfant (DC-PNSME), and The Bill & Melinda Gates Institute for Population and Reproductive Health at The Johns Hopkins Bloomberg School of Public Health. Performance Monitoring and Accountability 2020 (PMA2020) Survey round 1, PMA2017/Cote d'Ivoire-R1. 2017. Abidjan, Cote d'Ivoire and Baltimore, Maryland, USAâ�‹.	350350
Democratic Republic of the Congo	2015	SBH	Excluded pma2020 for inflated mortality and high missingness in reported child deaths	Tulane University School of Public Health, University of Kinshasa School of Public Health and The Bill & Melinda Gates Institute for Population and Reproductive Health at The Johns Hopkins Bloomberg School of Public Health. Performance Monitoring and Accountability 2020 (PMA2020) Survey round 3, PMA2015/DRC-R3 (Kinshasa). 2015. Kinshasa, DRC and Baltimore, Maryland, USA.	257826

Country	Year	Data Type	Reason	Citation	GHDx ID
Democratic Republic of the Congo	2015-2016	SBH	Excluded pma2020 for inflated mortality and high missingness in reported child deaths	Tulane University School of Public Health, University of Kinshasa School of Public Health and The Bill & Melinda Gates Institute for Population and Reproductive Health at The Johns Hopkins Bloomberg School of Public Health. Performance Monitoring and Accountability 2020 (PMA2020) Survey round 4, PMA2015/DRC-R4 (Kinshasa & Kongo Central). 2015. Kinshasa, DRC and Baltimore, Maryland, USA.	286019
Democratic Republic of the Congo	2016	SBH	Excluded pma2020 for inflated mortality and high missingness in reported child deaths	Tulane University School of Public Health, University of Kinshasa School of Public Health and The Bill & Melinda Gates Institute for Population and Reproductive Health at The Johns Hopkins Bloomberg School of Public Health. Performance Monitoring and Accountability 2020 (PMA2020) Survey round 5, PMA2016/DRC-R5 (Kinshasa & Kongo Central). 2016. Kinshasa, DRC and Baltimore, Maryland, USA.	286054
Djibouti	2012	CBH	missing birth or death date exceeded 10% (Africa Paper)	Department of Statistics and Demographic Studies (Djibouti), League of Arab States, Ministry of Health (Djibouti), Pan Arab Project for Family Health (PAPFAM). Djibouti Family Health Survey 2012.	218035
Ecuador	2012	CBH	Excluded, no age of death in dataset	Ministry of Public Health (Ecuador), National Institute of Statistics and Censuses (Ecuador). Ecuador National Health and Nutrition Survey 2012.	153674
Egypt	2013-2014	SBH	Excluded due to high number missing ced	El-Zanaty and Associates, Ministry of Health and Population (Egypt), United Nations Children’s Fund (UNICEF). Egypt IPHN Rural Districts Multiple Indicator Cluster Survey 2013-2014. New York, United States: United Nations Children’s Fund (UNICEF), 2016.	159617

Country	Year	Data Type	Reason	Citation	GHDx ID
Ethiopia	2007	SBH	80% missingness in SBH data and unrealistic geographic distribution of mortality risk (Excluded in prior paper)	Minnesota Population Center, Ethiopia Central Statistical Agency. Ethiopia Population and Housing Census 2007 from the Integrated Public Use Microdata Series, International: [Machine-readable database]. Minneapolis: University of Minnesota, 2015.	227133
Ethiopia	2008	CBH	Only has CBH for <5	Ethiopian Health and Nutrition Research Center (EHNRI), Macro International, Inc, Ministry of Health (Ethiopia). Ethiopia Global Fund Household Health Coverage Survey 2008.	26661
Ethiopia	2016	SBH	Excluded pma2020 for inflated mortality and high missingness in reported child deaths	Addis Ababa University School of Public Health and The Bill & Melinda Gates Institute for Population and Reproductive Health at The Johns Hopkins Bloomberg School of Public Health. Performance Monitoring and Accountability 2020 (PMA2020) Survey round 4, PMA2016/Ethiopia-R4. 2016. Ethiopia and Baltimore, Maryland, USA.PMA2020) Survey round 3, PMA2016/Burkina Faso-R3. 2016. Ouagadougou, Burkina Faso and Baltimore, Maryland, USA.	285891
Ethiopia	2017	SBH	Excluded pma2020 for inflated mortality and high missingness in reported child deaths	Addis Ababa University School of Public Health and The Bill & Melinda Gates Institute for Population and Reproductive Health at The Johns Hopkins Bloomberg School of Public Health. Performance Monitoring and Accountability 2020 (PMA2020) Survey round 5, PMA2017/Ethiopia-R5. 2017. Ethiopia and Baltimore, Maryland, USA.	347050
Ghana	2009-2010	SBH	Need age of woman to use SBH	Economic Growth Center, Yale University, Institute of Statistical, Social and Economic Research, University of Ghana. Ghana Socioeconomic Panel Survey 2009-2010. Washington DC, United States: World Bank.	236205

Country	Year	Data Type	Reason	Citation	GHDx ID
Ghana	2014	SBH	Excluded pma2020 for inflated mortality and high missingness in reported child deaths	Kwame Nkrumah University of Science & Technology School of Medicine and The Bill & Melinda Gates Institute for Population and Reproductive Health at The Johns Hopkins Bloomberg School of Public Health. Performance Monitoring and Accountability 2020 (PMA2020) Survey round 3, PMA2014/Ghana-R3. 2014. Ghana and Baltimore, Maryland, USA.	256243
Ghana	2015	SBH	Excluded pma2020 for inflated mortality and high missingness in reported child deaths	Bill and Melinda Gates Institute for Population and Reproductive Health, Johns Hopkins Bloomberg School of Public Health, Ghana Health Service, Ghana Statistical Service, Kwame Nkrumah University of Science and Technology (KNUST), University for Development Studies (Ghana). Ghana Performance Monitoring and Accountability 2020 Survey, Round 4 2015. Baltimore, United States: Bill and Melinda Gates Institute for Population and Reproductive Health, Johns Hopkins Bloomberg School of Public Health.	256244
Ghana	2016	SBH	Excluded pma2020 for inflated mortality and high missingness in reported child deaths	Kwame Nkrumah University of Science & Technology School of Medicine and The Bill & Melinda Gates Institute for Population and Reproductive Health at The Johns Hopkins Bloomberg School of Public Health. Performance Monitoring and Accountability 2020 (PMA2020) Survey round 5, PMA2016/Ghana-R5. 2016. Ghana and Baltimore, Maryland, USA.	286146
Guinea-Bissau	2010	SBH	Excluded due to high number missing ced	Centers for Disease Control and Prevention (CDC), National Statistics Institute (Guinea-Bissau), United Nations Children's Fund (UNICEF). Guinea-Bissau Multiple Indicator Cluster Survey 2010. New York, United States: United Nations Children's Fund (UNICEF), 2018.	27215

Country	Year	Data Type	Reason	Citation	GHDx ID
India	2005	CBH	Missingness, small sample size, and convoluted sampling methods	Desai, Sonalde, Reeve Vanneman, and National Council of Applied Economic Research, New Delhi. India Human Development Survey (IHDS), 2005. ICPSR22626-v8. Ann Arbor, MI: Inter-university Consortium for Political and Social Research [distributor], 2010-06-29. doi:10.3886/ICPSR22626.v8.	26919
India	2010	SBH	These age ranges dont match training data for SBH-CBH model	Harvard School of Public Health, International Institute for Population Sciences (India), RAND Corporation. India Longitudinal Aging Study Pilot 2010.	174154
India	2011-2012	CBH	Missingness, small sample size, and convoluted sampling methods	Desai, Sonalde, and Reeve Vanneman. India Human Development Survey-II (IHDS-II), 2011-12. ICPSR36151-v5. Ann Arbor, MI: Inter-university Consortium for Political and Social Research [distributor], 2016-08-01. http://doi.org/10.3886/ICPSR36151.v5	165498
Indonesia	2000	CBH	Excluded due to high number missing date of birth, skewed births towards past 5 years	Center for Population and Policy Studies, Gadjah Mada University (Indonesia), RAND Corporation. Indonesia Family Life Survey 2000. Santa Monica, United States: RAND Corporation.	6111
Indonesia	2000	CBH	Excluded due to high number missing date of birth	Statistics Indonesia. Indonesia Population and Housing Census 2000.	22674
Indonesia	2005	CBH	Excluded due to high number missing date of birth	Statistics Indonesia. Indonesia Intercensal Population Survey 2005.	6547
Indonesia	2007-2008	CBH	Excluded due to high number missing date of birth, skewed births towards past 5 years	Center for Population and Policy Studies, Gadjah Mada University (Indonesia), RAND Corporation, SurveyMETER. Indonesia Family Life Survey 2007-2008. Santa Monica, United States: RAND Corporation.	6464
Indonesia	2012	CBH	Excluded due to high number missing date of birth	National Team for the Acceleration of Poverty Reduction (TNP2K) (Indonesia), SurveyMETER, University of Southern California, World Bank. Indonesia Family Life Survey East 2012.	219201

Country	Year	Data Type	Reason	Citation	GHDx ID
Indonesia	2014-2015	CBH	Excluded due to high number missing date of birth	RAND Corporation, SurveyMETER. Indonesia Family Life Survey 2014-2015. Santa Monica, United States: RAND Corporation, 2016.	264956
Kenya	2009	SBH	Not representative sample	Kenya National Bureau of Statistics, United Nations Children's Fund (UNICEF). Kenya - Coast Multiple Indicator Cluster Survey 2009. New York, United States: United Nations Children's Fund (UNICEF), 2014.	56420
Kenya	2012-2013	CBH	Only collected data on youngest 3 children	Kenya National Bureau of Statistics, Ministry of Devolution and Planning (Kenya), Ministry of Health (Kenya), National AIDS and STI Control Program (Kenya). Kenya AIDS Indicator Survey 2012-2013. Nairobi, Kenya: Kenya National Bureau of Statistics.	133304
Kenya	2013-2014	CBH	missing birth or death date exceeded 10% (Excluded in prior paper)	Kenya National Bureau of Statistics, Population Studies and Research Institute, University of Nairobi (Kenya), United Nations Children's Fund (UNICEF). Kenya - Bungoma County Multiple Indicator Survey 2013-2014. New York, United States: United Nations Children's Fund (UNICEF), 2015.	203654
Kenya	2013-2014	CBH	missing birth or death date exceeded 10% (Excluded in prior paper)	Kenya National Bureau of Statistics, Population Studies and Research Institute, University of Nairobi (Kenya), United Nations Children's Fund (UNICEF). Kenya - Kakamega County Multiple Indicator Survey 2013-2014. New York, United States: United Nations Children's Fund (UNICEF), 2015.	203663
Kenya	2013-2014	CBH	missing birth or death date exceeded 10% (Excluded in prior paper)	Kenya National Bureau of Statistics, Population Studies and Research Institute, University of Nairobi (Kenya), United Nations Children's Fund (UNICEF). Kenya - Turkana County Multiple Indicator Survey 2013-2014. New York, United States: United Nations Children's Fund (UNICEF), 2015.	203664

Country	Year	Data Type	Reason	Citation	GHDx ID
Kenya	2015	SBH	Excluded pma2020 for inflated mortality and high missingness in reported child deaths	International Centre for Reproductive Health Kenya (ICRHK) and The Bill & Melinda Gates Institute for Population and Reproductive Health at The Johns Hopkins Bloomberg School of Public Health. Performance Monitoring and Accountability 2020 (PMA2020) Survey round 3, PMA2015/Kenya-R3. 2015. Kenya and Baltimore, Maryland, USA.	256365
Kenya	2015	SBH	Excluded pma2020 for inflated mortality and high missingness in reported child deaths	International Centre for Reproductive Health Kenya (ICRHK) and The Bill & Melinda Gates Institute for Population and Reproductive Health at The Johns Hopkins Bloomberg School of Public Health. Performance Monitoring and Accountability 2020 (PMA2020) Survey round 4, PMA2015/Kenya-R4. 2015. Kenya and Baltimore, Maryland, USA.	256366
Kenya	2016	SBH	Excluded pma2020 for inflated mortality and high missingness in reported child deaths	International Centre for Reproductive Health Kenya (ICRHK) and The Bill & Melinda Gates Institute for Population and Reproductive Health at The Johns Hopkins Bloomberg School of Public Health. Performance Monitoring and Accountability 2020 (PMA2020) Survey round 5, PMA2016/Kenya-R5. 2016. Kenya and Baltimore, Maryland, USA.	347047
Malawi	2007-2008	CBH	No SBH mortality and CBH only for children died	Ministry of Economic Planning and Development (Malawi), National Statistical Office of Malawi. Malawi Global Fund Household Health Coverage Survey 2007-2008.	26683
Mongolia	2008	SBH	Unable to geomatch	Ministry of Health (Mongolia), National Statistical Office of Mongolia. Mongolia Reproductive Health Survey 2008. Ulaanbaatar, Mongolia: National Statistical Office of Mongolia.	125230

Country	Year	Data Type	Reason	Citation	GHDx ID
Nepal	2010-2013	SBH	Excluded for multiple visits to same household	Fogarty International Center, National Institutes of Health (NIH), Foundation for the National Institutes of Health (FNIH), Institute of Medicine, Tribhuvan University, University of Bergen, Walter Reed/AFRIMS Research Unit Nepal (WARUN). Nepal - Bhaktapur Malnutrition and Enteric Disease Study 2009-2014.	261880
Niger	2012	CBH	dropped points from Diffa region for discordance with data points from the same surveys in nearby regions with similar sociodemographic profiles (Excluded in prior paper)	ICF International, Ministry of Public Health (Niger), National Institute of Statistics (Niger). Niger Demographic and Health Survey 2012. Fairfax, United States: ICF International.	74393
Niger	2017	SBH	Excluded pma2020 for inflated mortality and high missingness in reported child deaths	Niger/Niamey Institut National de la Statistique (National Institute of Statistics) and The Bill & Melinda Gates Institute for Population and Reproductive Health at The Johns Hopkins Bloomberg School of Public Health. Performance Monitoring and Accountability 2020 (PMA2020) Survey round 4, PMA2017/Niger-R4 (National). 2017. Niamey, Niger and Baltimore, Maryland, USA.	349890
Nigeria	2007	SBH	Excluded, over 50% missingness in ceb and ced	National Bureau of Statistics (Nigeria), Minnesota Population Center. Nigeria General Household Survey 2007 from the Integrated Public Use Microdata Series, International: [Machine-readable database]. Minneapolis: University of Minnesota.	151312
Nigeria	2008	SBH	Large amounts of missing data	National Bureau of Statistics (Nigeria), Minnesota Population Center. Nigeria General Household Survey 2008 from the Integrated Public Use Microdata Series, International: [Machine-readable database]. Minneapolis: University of Minnesota.	151313

Country	Year	Data Type	Reason	Citation	GHDx ID
Nigeria	2009	SBH	Data appeared unrelible with multiple rows stating persons aged <5 to have given birth to multiple children. Choosing not to use as have good qualilty data for 2008 and 2010. Excluded.	National Bureau of Statistics (Nigeria), Minnesota Population Center. Nigeria General Household Survey 2009 from the Integrated Public Use Microdata Series, International: [Machine-readable database]. Minneapolis: University of Minnesota.	151314
Nigeria	2010	SBH	Unrealistic distribution of CED	National Bureau of Statistics (Nigeria). Nigeria Living Standards Survey 2008-2010. Abuja, Nigeria: National Bureau of Statistics (Nigeria).	151719
Nigeria	2016	SBH	Excluded pma2020 for inflated mortality and high missingness in reported child deaths	Centre for Research, Evaluation Resources and Development (CRERD), Bayero University Kano (BUK), and The Bill & Melinda Gates Institute for Population and Reproductive Health at The Johns Hopkins Bloomberg School of Public Health. Performance Monitoring and Accountability 2020 (PMA2020) Survey round 3, PMA2016/Nigeria-R3 (National). 2016. Nigeria and Baltimore, Maryland, USA.	286022
Pakistan	2010-2013	SBH	Excluded for multiple visits to same household	Aga Khan University, Fogarty International Center, National Institutes of Health (NIH), Foundation for the National Institutes of Health (FNIH). Pakistan - Naushahro Feroze Malnutrition and Enteric Disease Study 2009-2014.	261883
Palestine	2007	SBH	Missing ced, ages are binned to 5yrs, Have CBH in this year	Palestinian Central Bureau of Statistics, Minnesota Population Center. Palestine Population, Housing, and Establishment Census 2007 from the Integrated Public Use Microdata Series, International: [Machine-readable database]. Minneapolis: University of Minnesota, 2011.	41088
Palestine	2007-2008	SBH	Excluded as IPUMS has this data and this data has spuriously high CED	Palestinian Central Bureau of Statistics. Palestine Population, Housing and Establishment Census 2007-2008.	10040

Country	Year	Data Type	Reason	Citation	GHDx ID
Paraguay	2010	SBH	Only has SBH for <15	General Directorate of Statistics, Surveys and Censuses (Paraguay). Paraguay Permanent Household Survey 2010. AsunciÃ³n, Paraguay: General Directorate of Statistics, Surveys and Censuses (Paraguay).	243537
Peru	2010-2013	SBH	Excluded for multiple visits to same household	Fogarty International Center, National Institutes of Health (NIH), Foundation for the National Institutes of Health (FNIH), Johns Hopkins Bloomberg School of Public Health. Peru - Loreto Malnutrition and Enteric Disease Study 2009-2014.	261879
Somalia	2011	CBH	missing birth or death date exceeded 10% (Excluded in prior paper)	Puntland Ministry of Planning and International Cooperation (Somalia), United Nations Children's Fund (UNICEF). Somalia - Northeast Zone Multiple Indicator Cluster Survey 2011. New York, United States: United Nations Children's Fund (UNICEF), 2015.	91508
South Africa	2004	SBH	Excluded due to high number missing ced	University of Kwazulu-Natal, University of Wisconsin, London School of Hygiene and Tropical Medicine, International Food Policy Research Institute (IFPRI), Department of Social Development (South Africa), Norwegian Institute for Urban and Regional Research (NIBR). South Africa KwaZulu-Natal Income Dynamics Study 2004. Durban, South Africa: University of Kwazulu-Natal.	31142
South Africa	2008	SBH	Survey series revisits households	University of Cape Town, Southern Africa Labour and Development Research Unit. National Income Dynamics Study (NIDS) Wave 1 [computer files]. Cape Town: Southern Africa Labour and Development Research Unit [producer], 2009. Cape Town: DataFirst [distributor], 2009	27885

Country	Year	Data Type	Reason	Citation	GHDx ID
South Africa	2010-2013	SBH	Excluded for multiple visits to same household	Fogarty International Center, National Institutes of Health (NIH), Foundation for the National Institutes of Health (FNIH), University of Venda. South Africa - Venda Malnutrition and Enteric Disease Study 2009-2014.	261887
South Africa	2011	SBH	Reported 0 ced	Statistics South Africa. South Africa Population and Housing Census 2011.	12146
South Africa	2011	CBH	missing birth or death date exceeded 10% (Excluded in prior paper)	Southern Africa Labour and Development Research Unit. National Income Dynamics Study 2010-2011, Wave 2. Version 1.0. Cape Town: Southern Africa Labour and Development Research Unit [producer], 2012. Cape Town: DataFirst [distributor], 2013.	133731
South Africa	2011-2012	SBH	Excluded due to high number missing ced	Centers for Disease Control and Prevention (CDC), Global Clinical and Viral Laboratory (South Africa), Human Sciences Research Council, National Institute for Communicable Diseases (South Africa), South African Medical Research Council, University of Cape Town. South Africa National HIV Prevalence, Incidence, and Behavior Survey 2011-2012. Pretoria, South Africa: Human Sciences Research Council, 2016.	313076
South Africa	2012	SBH	Survey series revisits households	Southern Africa Labour and Development Research Unit. National Income Dynamics Study 2012, Wave 3 [dataset]. Version 1.2. Cape Town: Southern Africa Labour and Development Research Unit [producer], 2013. Cape Town: DataFirst [distributor], 2013	133732

Country	Year	Data Type	Reason	Citation	GHDx ID
South Africa	2014-2015	SBH	Survey series revisits households	Southern Africa Labour and Development Research Unit. National Income Dynamics Study 2014 - 2015, Wave 4 [dataset]. Version 1.1. Cape Town: Southern Africa Labour and Development Research Unit [producer], 2016. Cape Town: DataFirst [distributor], 2016. Pretoria: Department of Planning Monitoring and Evaluation [commissioner], 2014	265153
South Africa	2017	SBH	Survey series revisits households	Southern Africa Labour and Development Research Unit. National Income Dynamics Study 2017, Wave 5 [dataset]. Version 1.0.0 Pretoria: Department of Planning, Monitoring, and Evaluation [funding agency]. Cape Town: Southern Africa Labour and Development Research Unit [implementer], 2018. Cape Town: DataFirst [distributor], 2018. 10.25828/fw3h-v708	369644
South Sudan	2010	CBH	Cannot currently handle country splits within time series	Central Bureau of Statistics (Sudan), Federal Ministry of Health (Sudan), Government of Sudan, Ministry of Health (South Sudan), Southern Sudan Centre for Census, Statistics and Evaluation. Sudan - South Multiple Indicator Cluster Survey 2010. New York, United States: United Nations Children's Fund (UNICEF), 2015.	32189
Tajikistan	2007	SBH	Need age of woman to use SBH	National State Statistical Agency (Tajikistan), World Bank. Tajikistan Living Standards Measurement Survey 2007.	12584
Tanzania	2009	SBH	Only has SBH for <5	Economic Development Initiatives (EDI). Tanzania Mainland Truck Roads and Zanzibar Rural Roads Activities Impact Evaluation 2009. High Wycombe, England: Economic Development Initiatives (EDI), 2010.	32332

Country	Year	Data Type	Reason	Citation	GHDx ID
Tanzania	2009-2014	SBH	Excluded for multiple visits to same household	Fogarty International Center, National Institutes of Health (NIH), Foundation for the National Institutes of Health (FNIH), Haydom Lutheran Hospital. Tanzania - Haydom Malnutrition and Enteric Disease Study 2009-2014.	261889
Timor-Leste	2001	SBH	Unable to geomatch	National Statistics Directorate (Timor-Leste), World Bank. Timor-Leste Living Standards and Measurement Survey 2001. Washington DC, United States: World Bank.	12863
Tunisia	2001	CBH	Excluded due to high number missing date of birth	League of Arab States, National Office for Family and Population, Ministry of Public Health (Tunisia), Pan Arab Project for Family Health (PAPFAM). Tunisia Family Health Survey 2001.	12978
Uganda	2010-2011	SBH	Panel Survey - Not representative	Uganda Bureau of Statistics. Uganda Living Standards Measurement Survey - Integrated Survey on Agriculture 2011-2012. Washington DC, United States: World Bank.	142935
Uganda	2015	SBH	Excluded pma2020 for inflated mortality and high missingness in reported child deaths	Makerere University, School of Public Health at the College of Health Sciences and The Bill & Melinda Gates Institute for Population and Reproductive Health at The Johns Hopkins Bloomberg School of Public Health. Performance Monitoring and Accountability 2020 (PMA2020) Survey round 3, PMA2015/Uganda-R3. 2015. Uganda and Baltimore, Maryland, USA.	256201
Uganda	2016	SBH	Excluded pma2020 for inflated mortality and high missingness in reported child deaths	Makerere University, School of Public Health at the College of Health Sciences and The Bill & Melinda Gates Institute for Population and Reproductive Health at The Johns Hopkins Bloomberg School of Public Health. Performance Monitoring and Accountability 2020 (PMA2020) Survey round 4, PMA2016/Uganda-R4. 2016. Uganda and Baltimore, Maryland, USA.	285893

Country	Year	Data Type	Reason	Citation	GHDx ID
Uganda	2017	SBH	Excluded pma2020 for inflated mortality and high missingness in reported child deaths	Makerere University, School of Public Health at the College of Health Sciences and The Bill & Melinda Gates Institute for Population and Reproductive Health at The Johns Hopkins Bloomberg School of Public Health. Performance Monitoring and Accountability 2020 (PMA2020) Survey round 5, PMA2017/Uganda-R5. 2017. Uganda and Baltimore, Maryland, USA.	347043
Zambia	2008	CBH	Only has CBH for <5	Central Statistical Office (Zambia). Zambia Global Fund Household Health Coverage Survey 2008. Lusaka, Zambia: Central Statistical Office (Zambia).	26702
