# Supplementary material for: Mapping 123 million neonatal, infant and child deaths between 2000 and 2017
Source: Nature. 2019 Oct 16;574(7778):353–8. doi: 10.1038/s41586-019-1545-0 (PMC6800389; doi:10.1038/s41586-019-1545-0)
Supplement: Supplementary file 3 — A full list of LBD Under-5 Mortality Collaborators and their affiliations. [file 41586_2019_1545_MOESM3_ESM.pdf]

LBD Under-5 Mortality Collaborators:

Roy Burstein*, Nathaniel J. Henry*, Michael L. Collison, Laurie B. Marczak, Amber Sligar, Stefanie Watson, Neal Marquez, Mahdieh Abbasalizad-Farhangi, Masoumeh Abbasi, Foad Abd-Allah, Amir Abdoli, Mohammad Abdollahi, Ibrahim Abdollahpour, Rizwan Suliankatchi Abdulkader, Michael R. M. Abrigo, Dilaram Acharya, Oladimeji M. Adebayo, Victor Adekanmbi, Davoud Adham, Mahdi Afshari, Mohammad Aghaali, Keivan Ahmadi, Mehdi Ahmadi, Ehsan Ahmadpour, Rushdia Ahmed, Chalachew Genet Akal, Joshua O. Akinyemi, Fares Alahdab, Noore Alam, Genet Melak Alamene, Kefyalew Addis Alene, Mehran Alijanzadeh, Cyrus Alinia, Vahid Alipour, Syed Mohamed Aljunid, Mohammed J. Almalki, Hesham M. Al-Mekhlafi, Khalid Altirkawi, Nelson Alvis-Guzman, Adeladza Kofi Amegah, Saeed Amini, Arianna Maever Loreche Amit, Zohreh Anbari, Sofia Androudi, Mina Anjomshoa, Fereshteh Ansari, Carl Abelardo T. Antonio, Jalal Arabloo, Zohreh Arefi, Olatunde Aremu, Bahram Armoon, Amit Arora, Al Artaman, Anvar Asadi, Mehran Asadi-Aliabadi, Amir Ashraf-Ganjouei, Reza Assadi, Bahar Ataeinia, Sachin R. Atre, Beatriz Paulina Ayala Quintanilla, Martin Amogre Ayanore, Samad Azari, Ebrahim Babaee, Arefeh Babazadeh, Alaa Badawi, Soghra Bagheri, Mojtaba Bagherzadeh, Nafiseh Baheiraei, Abbas Balouchi, Aleksandra Barac, Quique Bassat, Bernhard T. Baune, Mohsen Bayati, Neeraj Bedi, Ettore Beghi, Masoud Behzadifar, Meysam Behzadifar, Yared Belete Belay, Brent Bell, Michelle L. Bell, Dessalegn Ajema Berbada, Robert S. Bernstein, Natalia V. Bhattacharjee, Suraj Bhattarai, Zulfiqar A. Bhutta, Ali Bijani, Somayeh Bohlouli, Nicholas J. K. Breitborde, Gabrielle Britton, Annie J. Browne, Sharath Burugina Nagaraja, Reinhard Busse, Zahid A. Butt, Josip Car, Rosario Cárdenas, Carlos A. Castañeda-Orjuela, Ester Cerin, Wagaye Fentahun Chanie, Pranab Chatterjee, Dinh-Toi Chu, Cyrus Cooper, Vera M. Costa, Koustuv Dalal, Lalit Dandona, Rakhi Dandona, Farah Daoud, Ahmad Daryani, Rajat Das Gupta, Ian Davis, Nicole Davis Weaver, Dragos Virgil Davitoiu, Jan-Walter De Neve, Feleke Mekonnen Demeke, Gebre Teklemariam Demoz, Kebede Deribe, Rupak Desai, Aniruddha Deshpande, Hanna Demelash Desyibelew, Sagnik Dey, Samath Dhamminda Dharmaratne, Meghnath Dhimal, Daniel Diaz, Leila Doshmangir, Andre R. Duraes, Laura Dwyer-Lindgren, Lucas Earl, Roya Ebrahimi, Soheil Ebrahimpour, Andem Effiong, Aziz Eftekhari, Elham Ehsani-Chimeh, Iman El Sayed, Maysaa El Sayed Zaki, Maha El Tantawi, Ziad El-Khatib, Mohammad Hassan Emamian, Shymaa Enany, Sharareh Eskandarieh, Oghenowede Eyawo, Maha Ezalarab, Mahbobeh Faramarzi, Mohammad Fareed, Roghiyeh Faridnia, Andre Faro, Ali Akbar Fazaeli, Mehdi Fazlzadeh, Netsanet Fentahun, Seyed-Mohammad Fereshtehnejad, João C. Fernandes, Irina Filip, Florian Fischer, Nataliya A. Foigt, Masoud Foroutan, Joel Msafiri Francis, Takeshi Fukumoto, Nancy Fullman, Silvano Gallus, Destallem Gebremedhin Gebre, Tsegaye Tewelde Gebrehiwot, Gebreamlak Gebremedhn Gebremeskel, Bradford D. Gessner, Birhanu Geta, Peter W. Gething, Reza Ghadimi, Keyghobad Ghadiri, Mahsa Ghajarzadeh, Ahmad Ghashghaee, Paramjit Singh Gill, Tiffany K. Gill, Nick Golding, Nelson G. M. Gomes, Philimon N. Gona, Sameer Vali Gopalani, Giuseppe Gorini, Bárbara Niegia Garcia Goulart, Nicholas Graetz, Felix Greaves, Manfred S. Green, Yuming Guo, Arvin Haj-Mirzaian, Arya Haj-Mirzaian, Brian James Hall, Samer Hamidi, Hamidreza Haririan, Josep Maria Haro, Milad Hasankhani, Edris Hasanpoor, Amir Hasanzadeh, Hadi Hassankhani, Hamid Yimam Hassen, Mohamed I. Hegazy, Delia Hendrie, Fatemeh Heydarpour, Thomas R. Hird, Chi Linh Hoang, Gillian Hollerich, Enayatollah Homaie Rad, Mojtaba Hoseini-Ghahfarokhi, Naznin Hossain, Mostafa Hosseini, Mehdi Hosseinzadeh, Mihaela Hostiuc, Sorin Hostiuc, Mowafa Househ, Mohamed Hsairi, Olayinka Stephen Ilesanmi, Mohammad Hasan Imani-Nasab, Usman Iqbal, Seyed Sina Naghibi Irvani, Nazrul Islam, Sheikh Mohammed Shariful Islam, Mikk Jürisson, Nader Jafari Balalami, Amir Jalali, Javad Javidnia, Achala Upendra Jayatilleke, Ensiyeh Jenabi, John S. Ji, Yash B. Jobanputra, Kimberly Johnson, Jost B. Jonas, Zahra Jorjoran Shushtari, Jacek Jerzy Jozwiak, Ali Kabir, Amaha Kahsay, Hamed Kalani, Rohollah

Kalhor, Manoochehr Karami, Surendra Karki, Amir Kasaeian, Nicholas J. Kassebaum, Peter Njenga Keiyoro, Grant Rodgers Kemp, Roghayeh Khabiri, Yousef Saleh Khader, Morteza Abdullatif Khafaie, Ejaz Ahmad Khan, Junaid Khan, Muhammad Shahzeb Khan, Young-Ho Khang, Khaled Khatab, Amir Khater, Mona M. Khater, Alireza Khatony, Mohammad Khazaei, Salman Khazaei, Maryam Khazaei-Pool, Jagdish Khubchandani, Neda Kianipour, Yun Jin Kim, Ruth W. Kimokoti, Damaris K. Kinyoki, Adnan Kisa, Sezer Kisa, Tufa Kolola, Soewarta Kosen, Parvaiz A. Koul, Ai Koyanagi, Moritz U. G. Kraemer, Kewal Krishan, Kris J. Krohn, Nuworza Kugbey, G Anil Kumar, Manasi Kumar, Pushpendra Kumar, Desmond Kuupiel, Ben Lacey, Sheetal D. Lad, Faris Hasan Lami, Anders O. Larsson, Paul H. Lee, Mostafa Leili, Aubrey J. Levine, Shanshan Li, Lee-Ling Lim, Stefan Listl, Joshua Longbottom, Jaifred Christian F. Lopez, Stefan Lorkowski, Sameh Magdeldin, Hassan Magdy Abd El Razek, Muhammed Magdy Abd El Razek, Azeem Majeed, Afshin Maleki, Reza Malekzadeh, Deborah Carvalho Malta, Abdullah A. Mamun, Navid Manafi, Ana-Laura Manda, Morteza Mansourian, Francisco Rogerlândio Martins-Melo, Anthony Masaka, Benjamin Ballard Massenburg, Pallab K. Maulik, Bejamin K. Mayala, Mohsen Mazidi, Martin McKee, Ravi Mehrotra, Kala M. Mehta, Gebrekiros Gebremichael Meles, Walter Mendoza, Ritesh G. Menezes, Atte Meretoja, Tuomo J. Meretoja, Tomislav Mestrovic, Ted R. Miller, Molly K. Miller-Petrie, Edward J. Mills, George J. Milne, GK Mini, Seyed Mostafa Mir, Hamed Mirjalali, Erkin M. Mirrakhimov, Efat Mohamadi, Dara K. Mohammad, Aso Mohammad Darwesh, Naser Mohammad Gholi Mezerji, Ammas Siraj Mohammed, Shafiu Mohammed, Ali H. Mokdad, Mariam Molokhia, Lorenzo Monasta, Yoshan Moodley, Mahmood Moosazadeh, Ghobad Moradi, Masoud Moradi, Yousef Moradi, Maziar Moradi-Lakeh, Mehdi Moradinazar, Paula Moraga, Lidia Morawska, Abbas Mosapour, Seyyed Meysam Mousavi, Ulrich Otto Mueller, Atalay Goshu Muluneh, Ghulam Mustafa, Behnam Nabavizadeh, Mehdi Naderi, Ahamarshan Jayaraman Nagarajan, Azin Nahvijou, Farid Najafi, Vinay Nangia, Duduzile Edith Ndwandwe, Nahid Neamati, Ionut Negoi, Ruxandra Irina Negoi, Josephine W. Ngunjiri, Huong Lan Thi Nguyen, Long Hoang Nguyen, Son Hoang Nguyen, Katie R. Nielsen, Dina Nur Anggraini Ningrum, Yirga Legesse Nirayo, Molly R. Nixon, Chukwudi A. Nnaji, Marzieh Nojomi, Mehdi Noroozi, Shirin Nosratnejad, Jean Jacques Noubiap, Soraya Nouraei Motlagh, Richard Ofori-Asenso, Felix Akpojene Ogbo, Kelechi E. Oladimeji, Andrew T. Olagunju, Meysam Olfatifar, Solomon Olum, Bolajoko Olubukunola Olusanya, Mojisola Morenike Oluwasanu, Obinna E. Onwujekwe, Eyal Oren, Doris D. V. Ortega-Altamirano, Alberto Ortiz, Osayomwanbo Osarenotor, Frank B. Osei, Aaron E. Osgood-Zimmerman, Stanislav S. Otstavnov, Mayowa Ojo Owolabi, Mahesh P A, Abdol Sattar Pagheh, Smita Pakhale, Songhomitra Panda-Jonas, Animika Pandey, Eun-Kee Park, Hadi Parsian, Tahereh Pashaei, Sangram Kishor Patel, Veincent Christian Filipino Pepito, Alexandre Pereira, Samantha Perkins, Brandon V. Pickering, Thomas Pilgrim, Majid Pirestani, Bakhtiar Piroozi, Meghdad Pirsaheb, Oleguer Plana-Ripoll, Hadi Pourjafar, Parul Puri, Mostafa Qorbani, Hedley Quintana, Mohammad Rabiee, Navid Rabiee, Amir Radfar, Alireza Rafiei, Fakher Rahim, Zohreh Rahimi, Vafa Rahimi-Movaghar, Shadi Rahimzadeh, Fatemeh Rajati, Sree Bhushan Raju, Azra Ramezankhani, Chhabi Lal Ranabhat, Davide Rasella, Vahid Rashedi, Lal Rawal, Robert C. Reiner, Andre M. N. Renzaho, Satar Rezaei, Aziz Rezapour, Seyed Mohammad Riahi, Ana Isabel Ribeiro, Leonardo Roever, Elias Merdassa Roro, Max Roser, Gholamreza Roshandel, Daem Roshani, Ali Rostami, Enrico Rubagotti, Salvatore Rubino, Siamak Sabour, Nafis Sadat, Ehsan Sadeghi, Reza Saeedi, Yahya Safari, Roya Safari-Faramani, Mahdi Safdarian, Amirhossein Sahebkar, Mohammad Reza Salahshoor, Nasir Salam, Payman Salamati, Farkhonde Salehi, Saleh Salehi Zahabi, Yahya Salimi, Hamideh Salimzadeh, Joshua A. Salomon, Evanson Zondani Sambala, Abdallah M. Samy, Milena M. Santric Milicevic, Bruno Piassi Sao Jose, Sivan Yegnanarayana Iyer Saraswathy, Rodrigo Sarmiento-Suárez, Benn Sartorius, Brijesh Sathian, Sonia Saxena, Alyssa N. Sbarra, Lauren E. Schaeffer, David C. Schwebel, Sadaf G. Sepanlou,

Seyedmojtaba Seyedmousavi, Faramarz Shaahmadi, Masood Ali Shaikh, Mehran Shams-Beyranvand, Amir Shamshirian, Morteza Shamsizadeh, Kiomars Sharafi, Mehdi Sharif, Mahdi Sharif-Alhoseini, Hamid Sharifi, Jayendra Sharma, Rajesh Sharma, Aziz Sheikh, Chloe Shields, Mika Shigematsu, Rahman Shiri, Ivy Shiue, Kerem Shuval, Tariq J. Siddiqi, João Pedro Silva, Jasvinder A. Singh, Dhirendra Narain Sinha, Malede Mequanent Sisay, Solomon Sisay, Karen Sliwa, David L. Smith, Ranjani Somayaji, Moslem Soofi, Joan B. Soriano, Chandrashekhar T. Sreeramareddy, Agus Sudaryanto, Mu'awiyyah Babale Sufiyan, Bryan L. Sykes, PN Sylaja, Rafael Tabarés-Seisdedos, Karen M. Tabb, Takahiro Tabuchi, Nuno Taveira, Mohamad-Hani Temsah, Abdullah Sulieman Terkawi, Zemenu Tadesse Tessema, Kavumpurathu Raman Thankappan, Sathish Thirunavukkarasu, Quyen G. To, Marcos Roberto Tovani-Palone, Bach Xuan Tran, Khanh Bao Tran, Irfan Ullah, Muhammad Shariq Usman, Olalekan A. Uthman, Amir Vahedian-Azimi, Pascual R. Valdez, Job F. M. van Boven, Tommi Juhani Vasankari, Yasser Vasseghian, Yousef Veisani, Narayanaswamy Venketasubramanian, Francesco S. Violante, Sergey Konstantinovitch Vladimirov, Vasily Vlassov, Theo Vos, Giang Thu Vu, Isidora S. Vujcic, Yasir Waheed, Jon Wakefield, Haidong Wang, Yafeng Wang, Yuan-Pang Wang, Joseph L. Ward, Robert G. Weintraub, Kidu Gidey Weldegwergs, Girmay Teklay Weldesamuel, Ronny Westerman, Charles Shey Wiysonge, Dawit Dawit Zewdu Wondafrash, Lauren Woyczynski, Ai-Min Wu, Gelin Xu, Abbas Yadegar, Tomohide Yamada, Vahid Yazdi-Feyzabadi, Christopher Sabo Yilgwan, Paul Yip, Naohiro Yonemoto, Javad Yoosefi Lebni, Mustafa Z. Younis, Mahmoud Yousefifard, Hebat-Allah Salah A. Yousof, Chuanhua Yu, Hasan Yusefzadeh, Erfan Zabeh, Telma Zahirian Moghadam, Sojib Bin Zaman, Mohammad Zamani, Hamed Zandian, Alireza Zangeneh, Taddese Alemu Zerfu, Yunquan Zhang, Arash Ziapour, Sanjay Zodpey, Christopher J. L. Murray[§] & Simon I. Hay[§†].

*These authors contributed equally

§These authors jointly supervised the work

†Corresponding author

Institute for Health Metrics and Evaluation, University of Washington, Seattle, WA, USA
Roy Burstein, Nathaniel J. Henry, Michael L. Collison, Laurie B. Marczak, Amber Sligar, Stefanie Watson, Neal Marquez, Brent Bell, Natalia V. Bhattacharjee, Lalit Dandona, Rakhi Dandona, Farah Daoud, Ian Davis, Nicole Davis Weaver, Aniruddha Deshpande, Samath D. Dharmaratne, Laura Dwyer-Lindgren, Lucas Earl, Maha Ezalarab, Nancy Fullman, Nicholas Graetz, Gillian Hollerich, Kimberly Johnson, Nicholas J. Kassebaum, Grant R. Kemp, Damaris K. Kinyoki, Kris J. Krohn, Aubrey J. Levine, Bejamin K. Mayala, Molly K. Miller-Petrie, Ali H. Mokdad, Molly R. Nixon, Aaron E. Osgood-Zimmerman, Samantha Perkins, Brandon V. Pickering, Robert C. Reiner, Nafis Sadat, Alyssa N. Sbarra, Lauren E. Schaeffer, Chloe Shields, David L. Smith, Theo Vos, Haidong Wang, Lauren Woyczynski, Christopher J. L. Murray & Simon I. Hay

Department of Health Metrics Sciences, School of Medicine, University of Washington, Seattle, WA, USA
Laura Dwyer-Lindgren, Damaris K. Kinyoki, Ali H. Mokdad, Robert C. Reiner, Benn Sartorius, David L. Smith, Theo Vos, Haidong Wang, Christopher J. L. Murray & Simon I. Hay

Department of Anesthesiology & Pain Medicine, University of Washington, Seattle, WA, USA
Nicholas J. Kassebaum

Division of Plastic Surgery, University of Washington, Seattle, WA, USA
Benjamin B. Massenburg

Global Health Department, University of Washington, Seattle, WA, USA
Katie R. Nielsen

Department of Medicine, University of Washington, Seattle, WA, USA
Ranjani Somayaji

Department of Statistics, University of Washington, Seattle, WA, USA
Jon Wakefield

Department of Pediatrics, University of Washington, Seattle, WA, USA
Katie R. Nielsen

University of Washington, Seattle, WA, USA
Eyal Oren

Department of Nutrition, Tabriz University of Medical Sciences, Tabriz, Iran
Mahdieh Abbasalizad-Farhangi

Department of Parasitology and Mycology, Tabriz University of Medical Sciences, Tabriz, Iran
Ehsan Ahmadpour

Research Center for Evidence Based Medicine-Health Management and Safety Promotion Research Institute, Tabriz University of Medical Sciences, Tabriz, Iran
Fereshteh Ansari

Department of Health Policy and Economy, Tabriz University of Medical Sciences, Tabriz, Iran
Leila Doshmangir

Department of Toxicology and Pharmacology, Tabriz University of Medical Sciences, Tabriz, Iran
Aziz Eftekhari

School of Nutrition and Food Sciences, Tabriz University of Medical Sciences, Tabriz, Iran
Milad Hasankhani

School of Nursing and Midwifery Tabriz University of Medical Sciences, Tabriz, Iran
Hadi Hassankhani

Tabriz Health Management Research Center, Tabriz University of Medical Sciences, Tabriz, Iran
Roghayeh Khabiri

Department of Health Economics, Tabriz University of Medical Sciences, Tabriz, Iran
Shirin Nosratnejad

Tabriz University of Medical Sciences, Tabriz, Iran
Hamidreza Haririan

Research Center for Environmental Determinants of Health, Kermanshah University of Medical Sciences, Kermanshah, Iran
Anvar Asadi, Mehdi Moradinazar, Ehsan Sadeghi & Masoud Moradi

Medical Biology Research Center, Kermanshah University of Medical Sciences, Kermanshah, Iran
Fatemeh Heydarpour

Radiology and Nuclear Medicine Department, Kermanshah University of Medical Sciences, Kermanshah, Iran
Mojtaba Hoseini-Ghahfarokhi

Psychiatric Department, Kermanshah University of Medical Sciences, Kermanshah, Iran
Amir Jalali

Operating Room Department, Kermanshah University of Medical Sciences, Kermanshah, Iran
Mehdi Naderi

Department of Epidemiology & Biostatistics, Kermanshah University of Medical Sciences, Kermanshah, Iran
Farid Najafi & Yahya Salimi

Department of Clinical Biochemistry, Kermanshah University of Medical Sciences, Kermanshah, Iran
Zohreh Rahimi

Department of Health Education & Promotion, Kermanshah University of Medical Sciences, Kermanshah, Iran
Fatemeh Rajati

Environmental Determinants of Health Research Center, Kermanshah University of Medical Sciences, Kermanshah, Iran
Satar Rezaei

Faculty of Public Health, Kermanshah University of Medical Sciences, Kermanshah, Iran
Roya Safari-Faramani

Department of Anatomical Sciences, Kermanshah University of Medical Sciences, Kermanshah, Iran
Mohammad R. Salahshoor

Taleghani Hospital, Kermanshah University of Medical Sciences, Kermanshah, Iran
Farkhonde Salehi

Department of Radiology and Nuclear Medicine, Kermanshah University of Medical Sciences, Kermanshah, Iran
Saleh Salehi Zahabi

Social Development and Health Promotion Research Center, Kermanshah University of Medical Sciences, Kermanshah, Iran
Moslem Soofi

Research Center for Environmental Determinants of Health (RCEDH), Kermanshah University of Medical Sciences, Kermanshah, Iran
Yasser Vasseghian

Social Development and Health Promotion Research Center, Kermanshah University of Medical Sciences, Kermanshah, Iran
Alireza Zangeneh

School of Public Health, Kermanshah University of Medical Sciences, Kermanshah, Iran
Neda Kianipour

Kermanshah University of Medical Sciences, Kermanshah, Iran
Masoumeh Abbasi, Soghra Bagheri, Keyghobad Ghadiri, Alireza Khatony, Neda Kianipour, Masoud Moradi, Meghdad Pirsaheb, Yahya Safari & Kiomars Sharafi

Department of Neurology, Cairo University, Cairo, Egypt
Foad Abd-Allah & Mohamed I. Hegazy

Department of Medical Parasitology, Cairo University, Cairo, Egypt
Mona M. Khater

Medical Parasitology Department, Cairo University, Cairo, Egypt
Hebat-Allah S. A. Yousof

Department of Parasitology and Mycology, Jahrom University of Medical Sciences, Jahrom, Iran
Amir Abdoli

The Institute of Pharmaceutical Sciences, Tehran University of Medical Sciences, Tehran, Iran
Mohammad Abdollahi

Department of Health Promotion and Education, Tehran University of Medical Sciences, Tehran, Iran
Zohreh Arefi

Faculty of Medicine, Tehran University of Medical Sciences, Tehran, Iran
Amir Ashraf-Ganjouei

Non-communicable Diseases Research Center, Tehran University of Medical Sciences, Tehran, Iran
Bahar Ataeinia, Shadi Rahimzadeh & Mehran Shams-Beyranvand

National Institute for Health Researchers, Tehran University of Medical Sciences, Tehran, Iran
Elham Ehsani-Chimeh

Multiple Sclerosis Research Center, Tehran University of Medical Sciences, Tehran, Iran
Amir Ashraf-Ganjouei, Ibrahim Abdollahpour & Sharareh Eskandarieh

Environmental Health Engineering, Tehran University of Medical Sciences, Tehran, Iran
Mehdi Fazlzadeh

Department of Neurology, Tehran University of Medical Sciences, Tehran, Iran
Mahsa Ghajarzadeh

Department of Pharmacology, Tehran University of Medical Sciences, Tehran, Iran
Arvin Haj-Mirzaian & Arya Haj-Mirzaian

Department of Microbiology, Tehran University of Medical Sciences, Tehran, Iran
Amir Hasanzadeh

Department of Epidemiology and Biostatistics, Tehran University of Medical Sciences, Tehran, Iran
Mostafa Hosseini

Hematologic Malignancies Research Center, Tehran University of Medical Sciences, Tehran, Iran
Amir Kasaeian

National Institute for Health Research (NIHR), Tehran University of Medical Sciences, Tehran, Iran
Roghayeh Khabiri

Digestive Diseases Research Institute, Tehran University of Medical Sciences, Tehran, Iran
Reza Malekzadeh, Gholamreza Roshandel, Hamideh Salimzadeh & Sadaf G. Sepanlou

Health Equity Research Center, Tehran University of Medical Sciences, Tehran, Iran
Efat Mohamadi

Department of Health Management and Economics, Tehran University of Medical Sciences, Tehran, Iran
Seyyed Meysam Mousavi

Department of Urology, Tehran University of Medical Sciences, Tehran, Iran
Behnam Nabavizadeh

Cancer Research Center of Cancer Institute, Tehran University of Medical Sciences, Tehran, Iran
Azin Nahvijou

Endocrinology and Metabolism Molecular-Cellular Sciences Institute, Tehran University of Medical Sciences, Tehran, Iran
Fakher Rahim

Sina Trauma and Surgery Research Center, Tehran University of Medical Sciences, Tehran, Iran
Vafa Rahimi-Movaghar, Mahdi Safdarian, Payman Salamati & Mahdi Sharif-Alhoseini

Center of Expertise in Microbiology, Tehran University of Medical Sciences, Tehran, Iran
Seyedmojtaba Seyedmousavi

Hematology-Oncology and Stem Cell Transplantation Research Center, Tehran University of Medical Sciences, Tehran, Iran
Amir Kasaeian

Department of Epidemiology, Arak University of Medical Sciences, Arak, Iran
Ibrahim Abdollahpour

Health Services Management Department, Arak University of Medical Sciences, Arak, Iran
Saeed Amini & Zohreh Anbari

Department of Public Health, Ministry of Health, Riyadh, Saudi Arabia
Rizwan S. Abdulkader

Policy and Planning Division, Ministry of Health, Riyadh, Saudi Arabia
Jayendra Sharma

Research Department, Philippine Institute for Development Studies, Quezon City, The Philippines
Michael R.M. Abrigo

Department of Preventive Medicine, Dongguk University, Gyeongju, South Korea
Dilaram Acharya

Department of Community Medicine, Kathmandu University, Devdaha, Nepal
Dilaram Acharya

College of Medicine, University College Hospital, Ibadan, Nigeria
Oladimeji M. Adebayo

School of Medicine, Cardiff University, Cardiff, UK
Victor Adekanmbi

School of Health, Ardabil University of Medical Science, Ardabil, Iran
Davoud Adham

Department of Environmental Health Engineering, Ardabil University of Medical Science, Ardabil, Iran
Mehdi Fazlzadeh

Social Determinants of Health Research Center, Ardabil University of Medical Science, Ardabil, Iran
Telma Zahirian Moghadam & Hamed Zandian

Department of Community Medicine, Ardabil University of Medical Science, Ardabil, Iran
Hamed Zandian

Department of Community Medicine, Zabol University of Medical Sciences, Zabol, Iran
Mahdi Afshari

Department of Epidemiology and Biostatistics, Qom University of Medical Sciences, Qom, Iran
Mohammad Aghaali

School of Pharmacy, University of Lincoln, Lincoln, UK
Keivan Ahmadi

Environmental Technologies Research Center, Ahvaz Jundishapur University of Medical Sciences, Ahvaz, Iran
Mehdi Ahmadi

Social Determinants of Health Research Center, Ahvaz Jundishapur University of Medical Sciences, Ahvaz, Iran
Morteza A. Khafaie

Thalassemia and Hemoglobinopathy Research Center, Ahvaz Jundishapur University of Medical Sciences, Ahvaz, Iran
Fakher Rahim

James P Grant School of Public Health, Brac University, Dhaka, Bangladesh
Rushdia Ahmed & Rajat Das Gupta

Health Systems and Population Studies Division, International Centre for Diarrhoeal Disease Research, Bangladesh, Dhaka, Bangladesh
Rushdia Ahmed & Naznin Hossain

Maternal and Child Health Division, International Centre for Diarrhoeal Disease Research, Bangladesh, Dhaka, Bangladesh
Sojib bin Zaman

Department of Medical Laboratory Science, Bahir Dar University, Bahir Dar, Ethiopia
Chalachew G. Akal

Public Health Nutrition, Bahir Dar University, Bahir Dar, Ethiopia
Hanna D. Desyibelew

Department of Public Health Nutrition, Bahir Dar University, Bahir Dar, Ethiopia
Netsanet Fentahun

Bahir Dar University, Bahir Dar, Ethiopia
Feleke M. Demeke

Epidemiology and Medical Statistics, University of Ibadan, Ibadan, Nigeria
Joshua O. Akinyemi

Department of Community Medicine, University of Ibadan, Ibadan, Nigeria
Olayinka S. Ilesanmi

Department of Health Promotion and Education, University of Ibadan, Ibadan, Nigeria
Mojisola M. Oluwasanu

Institute for Advanced Medical Research and Training, University of Ibadan, Ibadan, Nigeria
Mayowa O. Owolabi

Evidence Based Practice Center, Mayo Clinic Foundation for Medical Education and Research, Rochester, MN, USA
Fares Alahdab

Prevention Division, Queensland Health, Herston, QLD, Australia
Noore Alam

School of Health Sciences, Madda Walabu University, Bale Goba, Ethiopia
Genet Melak Alamene

Institute of Public Health, University of Gondar, Gondar, Ethiopia
Kefyalew A. Alene

Department of Obstetrics and Gynaecology, University of Gondar, Gondar, Ethiopia
Wagaye F. Chanie

Department of Epidemiology and Biostatistics, University of Gondar, Gondar, Ethiopia
Zemenu T. Tessema, Malede M. Sisay & Atalay G. Muluneh

Research School of Population Health, Australian National University, Canberra, ACT, Australia
Kefyalew A. Alene

Social Determinants of Health Research Center, Qazvin University of Medical Sciences, Qazvin, Iran
Rohollah Kalhor

Qazvin University of Medical Sciences, Qazvin, Iran
Mehran Alijanzadeh

Department of Health Care Management and Economics, Urmia University of Medical Science, Urmia, Iran
Cyrus Alinia

Health Economics Department, Iran University of Medical Sciences, Tehran, Iran
Vahid Alipour

Health Management and Economics Research Center, Iran University of Medical Sciences, Tehran, Iran
Jalal Arabloo, Samad Azari, Mehdi Hosseinzadeh, Aziz Rezapour, Telma Zahirian Moghadam & Vahid Alipour

Preventive Medicine and Public Health Research Center, Iran University of Medical Sciences, Tehran, Iran
Mehran Asadi-Aliabadi, Ebrahim Babaee, Maziar Moradi-Lakeh & Marzieh Nojomi

School of Nursing and Midwifery, Iran University of Medical Sciences, Tehran, Iran
Abbas Balouchi

Department of Health Services Management, Iran University of Medical Sciences, Tehran, Iran
Ahmad Ghashghaee

Minimally Invasive Surgery Research Center, Iran University of Medical Sciences, Tehran, Iran
Ali Kabir

Ophthalmology Department, Iran University of Medical Sciences, Tehran, Iran
Navid Manafi

Department of Health Education and Health Promotion, Iran University of Medical Sciences, Tehran, Iran
Morteza Mansourian, Javad Yoosefi Lebni & Arash Ziapour

Department of Epidemiology, Iran University of Medical Sciences, Tehran, Iran
Yousef Moradi

Department of Community and Family Medicine, Iran University of Medical Sciences, Tehran, Iran
Marzieh Nojomi

School of Behavioral Sciences and Mental Health, Iran University of Medical Sciences, Tehran, Iran
Vahid Rashedi

Department of Neuroscience, Iran University of Medical Sciences, Tehran, Iran
Mahdi Safdarian

Department of Health Policy and Management, Kuwait University, Safat, Kuwait
Syed M. Aljunid

International Centre for Casemix and Clinical Coding, National University of Malaysia, Bandar Tun Razak, Malaysia
Syed M. Aljunid

Medical Research Center, Jazan University, Jazan, Saudi Arabia
Hesham M. Al-Mekhlafi

Faculty of Public Health and Tropical Medicine, Jazan University, Jazan, Saudi Arabia
Mohammed J. Almalki

Jazan University, Jazan, Saudi Arabia
Mohammed J. Almalki & Neeraj Bedi

Department of Medical Parasitology, Sana'a University, Sana'a, Yemen
Hesham M. Al-Mekhlafi

Department of Pediatrics, King Saud University, Riyadh, Saudi Arabia
Mohamad-Hani Temsah

King Saud University, Riyadh, Saudi Arabia
Khalid Altirkawi

Research Group in Health Economics, Universidad de Cartagena, Cartagena, Colombia
Nelson Alvis-Guzman

Research Group in Hospital Management and Health Policies, Universidad de la Costa, Barranquilla, Colombia
Nelson Alvis-Guzman

Biomedical Science, University of Cape Coast, Cape Coast, Ghana
Adeladza K. Amegah

Department of Epidemiology and Biostatistics, University of the Philippines Manila, Manila, The Philippines
Arianna Maever L. Amit & Jaifred Christian F. Lopez

Department of Health Policy and Administration, University of the Philippines Manila, Manila, The Philippines
Carl Abelardo T. Antonio

Online Programs for Applied Learning, Johns Hopkins University, Baltimore, Maryland, USA
Arianna Maever L. Amit

Center for Clinical Global Health Education, Johns Hopkins University, Baltimore, Maryland, USA
Sachin R. Atre

Department of Radiology, Johns Hopkins University, Baltimore, Maryland, USA
Arya Haj-Mirzaian

Department of Medicine, University of Thessaly, Volos, Greece
Sofia Androudi

Social Determinants of Health Research Center, Rafsanjan University of Medical Sciences, Rafsanjan, Iran
Mina Anjomshoa

Department of Applied Social Sciences, Hong Kong Polytechnic University, Hong Kong, China
Carl Abelardo T. Antonio

School of Nursing, Hong Kong Polytechnic University, Hong Kong, China
Paul H. Lee

School of Health Sciences, Birmingham City University, Birmingham, UK
Olatunde Aremu

School of Nursing and Midwifery, Saveh University of Medical Sciences, Saveh, Iran
Bahram Armoon

Social Determinants of Health Research Center, Saveh University of Medical Sciences, Saveh, Iran
Bahram Armoon

School of Science and Health, Western Sydney University, Penrith, NSW, Australia
Amit Arora & Lal Rawal

Translational Health Research Institute, Western Sydney University, Penrith, NSW, Australia
Felix A. Ogbo

School of Social Sciences and Psychology, Western Sydney University, Penrith, NSW, Australia
Andre M.N. Renzaho

Social Science and Psychology, Western Sydney University, Penrith, NSW, Australia
Lal Rawal

Oral Health Services, Sydney Local Health District, Sydney, NSW, Australia
Amit Arora

Department of Community Health Sciences, University of Manitoba, Winnipeg, MB, Canada
Al Artaman

Department Ophthalmology, University of Manitoba, Winnipeg, MB, Canada
Navid Manafi

Education Development Center, Mashhad University of Medical Sciences, Mashhad, Iran
Reza Assadi

Neurogenic Inflammation Research Center, Mashhad University of Medical Sciences, Mashhad, Iran
Amirhossein Sahebkar

Biotechnology Research Center, Mashhad University of Medical Sciences, Mashhad, Iran
Amirhossein Sahebkar

Dr. D.Y. Patil Medical College, Pune, India
Sachin R. Atre

The Judith Lumley Centre, La Trobe University, Melbourne, VIC, Australia
Beatriz Paulina Ayala Quintanilla

General Office for Research and Technological Transfer, Peruvian National Institute of Health, Lima, Peru
Beatriz Paulina Ayala Quintanilla

Department of Family and Community Health, University of Health and Allied Sciences, Ho, Ghana
Martin A. Ayanore

Family and Community Health, University of Health and Allied Sciences, Ho, Ghana
Nuworza Kugbey

Center for Infectious Diseases Research, Babol, Iran
Arefeh Babazadeh & Soheil Ebrahimpour

Public Health Risk Sciences Division, Public Health Agency of Canada, Toronto, ON, Canada
Alaa Badawi

Department of Nutritional Sciences, University of Toronto, Toronto, ON, Canada
Alaa Badawi

The Centre for Global Child Health, Hospital for Sick Children, University of Toronto, Toronto, ON, Canada
Zulfiqar A. Bhutta

Department of Chemistry, Sharif University of Technology, Tehran, Iran
Mojtaba Bagherzadeh & Navid Rabiee

Department of Electrical Engineering, Sharif University of Technology, Tehran, Iran
Erfan Zabeh

Tissue Engineering and Applied Cell Sciences Division, Tarbiat Modares University, Tehran, Iran
Nafiseh Baheiraei

Department of Clinical Biochemistry, Tarbiat Modares University, Tehran, Iran
Abbas Mosapour

Parasitology and Entomology Department, Tarbiat Modares University, Tehran, Iran
Majid Pirestani

Division of Diseases, Advanced Technologies Research Group, Tehran, Iran
Nafiseh Baheiraei

Clinic for Infectious and Tropical Diseases, Clinical Center of Serbia, Belgrade, Serbia
Aleksandra Barac

Faculty of Medicine, University of Belgrade, Belgrade, Serbia
Aleksandra Barac & Isidora S. Vujcic

Centre School of Public Health and Health Management, University of Belgrade, Belgrade, Serbia
Milena M. Santric Milicevic

Barcelona Institute for Global Health, University of Barcelona, Barcelona, Spain
Quique Bassat

Catalan Institution for Research and Advanced Studies (ICREA), Barcelona, Spain
Quique Bassat & Ai Koyanagi

Department of Psychiatry, Melbourne Medical School, Melbourne, VIC, Australia
Bernhard T. Baune

Health Human Resources Research Center, Shiraz University of Medical Sciences, Shiraz, Iran
Mohsen Bayati

Non-communicable Diseases Research Center, Shiraz University of Medical Sciences, Shiraz, Iran
Reza Malekzadeh & Sadaf G. Sepanlou

Department of Community Medicine, Gandhi Medical College Bhopal, Bhopal, India
Neeraj Bedi

Department of Neuroscience, Mario Negri Institute for Pharmacological Research, Milan, Italy
Ettore Beghi

Department of Environmental Health Science, Mario Negri Institute for Pharmacological Research, Milan, Italy
Silvano Gallus

Social Determinants of Health Research Center, Lorestan University of Medical Sciences, Khorramabad, Iran
Masoud Behzadifar

Hepatitis Research Center, Lorestan University of Medical Sciences, Khorramabad, Iran
Meysam Behzadifar

Department of Public Health, Lorestan University of Medical Sciences, Khorramabad, Iran
Mohammad Hasan Imani-Nasab & Soraya Nouraei Motlagh

Pharmacoepidemiology and Social Pharmacy, Mekelle University, Mekelle, Ethiopia
Yared Belete Belay

School of Nursing, Mekelle University, Mekelle, Ethiopia
Gebreamlak G. Gebremeskel

Department of Nutrition and Dietetics, Mekelle University, Mekelle, Ethiopia
Amaha Kahsay

Clinical Pharmacy Unit, Mekelle University, Mekelle, Ethiopia
Yirga L. Nirayo & Kidu G. Weldegwergs

Department of Pharmacology and Toxicology, Mekelle University, Mekelle, Ethiopia
Dawit D.Z. Wondafrash

Mekelle University, Mekelle, Ethiopia
Destallem G. Gebre

School of Forestry and Environmental Studies, Yale University, New Haven, CT, USA
Michelle L. Bell

Department of Public Health, Arba Minch University, Arba Minch, Ethiopia
Dessalegn A. Berbada & Gebrekiros G. Meles

Hubert Department of Global Health, Emory University, Atlanta, GA, USA
Robert S. Bernstein

Department of Global Health, University of South Florida, Tampa, FL, USA
Robert S. Bernstein

Department of Health Services Research and Policy, London School of Hygiene & Tropical Medicine, London, UK
Martin McKee

Faculty of Infectious and Tropical Diseases, London School of Hygiene & Tropical Medicine, London, UK
Benn Sartorius

London School of Hygiene & Tropical Medicine, London, UK
Suraj Bhattarai

Nepal Academy of Science & Technology, Patan, Nepal
Suraj Bhattarai

Center of Excellence in Women and Child Health, Aga Khan University, Karachi, Pakistan
Zulfiqar A. Bhutta

Social Determinants of Health Research Center, Babol University of Medical Sciences, Babol, Iran
Ali Bijani

Health Research Institute, Babol University of Medical Sciences, Babol, Iran
Reza Ghadimi

Department of Clinical Biochemistry, Babol University of Medical Sciences, Babol, Iran
Seyed Mostafa Mir, Abbas Mosapour, Nahid Neamati & Hadi Parsian

Infectious Diseases and Tropical Medicine Research Center, Babol University of Medical Sciences, Babol, Iran
Ali Rostami

Student Research Committee, Babol University of Medical Sciences, Babol, Iran
Mohammad Zamani

Babol University of Medical Sciences, Babol, Iran
Mahbobeh Faramarzi

Department of Veterinary Medicine, Karaj Islamic Azad University, Kermanshah, Iran
Somayeh Bohlouli

Department of Laboratory Sciences, Karaj Islamic Azad University, Kermanshah, Iran
Mehdi Sharif

Department of Basic Sciences, Karaj Islamic Azad University, Kermanshah, Iran
Mehdi Sharif

Department of Psychology, Ohio State University, Columbus, OH, USA
Nicholas J.K. Breitborde

Psychiatry and Behavioral Health Department, Ohio State University, Columbus, OH, USA
Nicholas J.K. Breitborde

Neuroscience Department, Institute for Scientific Research and High Technology Services, City of Knowledge, Panama
Gabrielle Britton

Gorgas Memorial Institute for Health Studies, Panama, Panama
Gabrielle Britton & Hedley Quintana

Big Data Institute, Li Ka Shing Centre for Health Information and Discovery, University of Oxford, Oxford, UK
Annie J. Browne & Peter W. Gething

Department of Rheumatology, University of Oxford, Oxford, UK
Cyrus Cooper

Department of Zoology, University of Oxford, Oxford, UK
Moritz U.G. Kraemer

Nuffield Department of Population Health, University of Oxford, Oxford, UK
Ben Lacey

Martin School, University of Oxford, Oxford, UK
Max Roser

Department of Community Medicine, Employees' State Insurance Model Hospital, Bangalore, India
Sharath Burugina Nagaraja

Department for Health Care Management, Technical University of Berlin, Berlin, Germany
Reinhard Busse

School of Population and Public Health, University of British Columbia, Vancouver, BC, Canada
Zahid A. Butt

Al Shifa School of Public Health, Al Shifa Trust Eye Hospital, Rawalpindi, Pakistan
Zahid A. Butt

Centre for Population Health Sciences, Nanyang Technological University, Singapore, Singapore
Josip Car

Nanyang Technological University, Singapore, Singapore
Sathish Thirunavukkarasu

Global Ehealth Unit, Imperial College London, London, UK
Josip Car

Department of Primary Care and Public Health, Imperial College London, London, UK
Felix Greaves & Azeem Majeed

School of Public Health, Imperial College London, London, UK
Sonia Saxena

Department of Population and Health, Metropolitan Autonomous University, Mexico City, Mexico
Rosario Cárdenas

Colombian National Health Observatory, National Institute of Health, Bogota, Colombia
Carlos A. Castañeda-Orjuela

Epidemiology and Public Health Evaluation Group, National University of Colombia, Bogota, Colombia
Carlos A. Castañeda-Orjuela

Mary Mackillop Institute for Health Research, Australian Catholic University, Melbourne, VIC, Australia
Ester Cerin

School of Public Health, University of Hong Kong, Hong Kong, China
Ester Cerin

Centre for Suicide Research and Prevention, University of Hong Kong, Hong Kong, China
Paul Yip

Department of Social Work and Social Administration, University of Hong Kong, Hong Kong, China
Paul Yip

Division of Epidemiology, National Institute of Cholera and Enteric Diseases, Kolkata, India
Pranab Chatterjee

Faculty of Biology, Hanoi National University of Education, Hanoi, Vietnam
Dinh-Toi Chu

Medical Research Council Lifecourse Epidemiology Unit, University of Southampton, Southampton, UK
Cyrus Cooper

Applied Molecular Biosciences Unit (UCIBIO), University of Porto, Porto, Portugal
Vera M. Costa & João P. Silva

Department of Chemistry, University of Porto, Porto, Portugal
Nelson G.M. Gomes

EPIUnit, University of Porto, Porto, Portugal
Ana Isabel Ribeiro

Institute of Public Health Kalyani, Kalyani, India
Koustuv Dalal

School of Health Science, Orebro University, Orebro, Sweden
Koustuv Dalal

Indian Institute of Public Health, Public Health Foundation of India, Gurugram, India
Sanjay Zodpey

Public Health Foundation of India, Gurugram, India
Lalit Dandona, Rakhi Dandona, G Anil Kumar & Animika Pandey

Toxoplasmosis Research Center, Mazandaran University of Medical Sciences, Sari, Iran
Ahmad Daryani & Abdol Sattar Pagheh

Department of Parasitology, Mazandaran University of Medical Sciences, Sari, Iran
Roghiyeh Faridnia

Department of Medical Mycology, Mazandaran University of Medical Sciences, Sari, Iran
Javad Javidnia

Department of Public Health, Mazandaran University of Medical Sciences, Sari, Iran
Maryam Khazaei-Pool

Health Sciences Research Center, Mazandaran University of Medical Sciences, Sari, Iran
Mahmood Moosazadeh

Molecular and Cell Biology Research Center, Mazandaran University of Medical Sciences, Sari, Iran
Alireza Rafiei

Invasive Fungi Research Center, Mazandaran University of Medical Sciences, Sari, Iran
Seyedmojtaba Seyedmousavi

Medical Laboratory Sciences, Mazandaran University of Medical Sciences, Sari, Iran
Amir Shamshirian

Department of Immunology, Mazandaran University of Medical Sciences, Sari, Iran
Alireza Rafiei

Mazandaran University of Medical Sciences, Sari, Iran
Hamed Kalani

Department of General Surgery, Carol Davila University of Medicine and Pharmacy, Bucharest, Romania
Dragos V. Davitoiu & Mihaela Hostiuc

Faculty of Dentistry, Carol Davila University of Medicine and Pharmacy, Bucharest, Romania
Sorin Hostiuc

Emergency Hospital of Bucharest, Carol Davila University of Medicine and Pharmacy, Bucharest, Romania
Ionut Negoi

Anatomy and Embryology Department, Carol Davila University of Medicine and Pharmacy, Bucharest, Romania
Ruxandra I. Negoi

General Surgery Department, Carol Davila University of Medicine and Pharmacy, Bucharest, Romania
Ionut Negoi

Department of Surgery, Clinical Emergency Hospital Sf. Pantelimon, Bucharest, Romania
Dragos V. Davitoiu

Heidelberg Institute of Global Health (HIGH), Heidelberg University, Heidelberg, Germany
Jan-Walter de Neve

Department of Ophthalmology, Heidelberg University, Heidelberg, Germany
Jost B. Jonas

Institute of Public Health, Heidelberg University, Heidelberg, Germany
Shafiu Mohammed

Heidelberg University, Heidelberg, Germany
Songhomitra Panda-Jonas

School of Pharmacy, Aksum University, Aksum, Ethiopia
Gebre T. Demoz

Nursing Department, Aksum University, Aksum, Ethiopia
Gebreamlak G. Gebremeskel

School of Nursing, Aksum University, Aksum, Ethiopia
Girmay T. Weldesamuel

School of Public Health, Addis Ababa University, Addis Ababa, Ethiopia
Kebede Deribe

Public Health, Addis Ababa University, Addis Ababa, Ethiopia
Elias E.M. Roro

Department of Pharmacology, Addis Ababa University, Addis Ababa, Ethiopia
Dawit D.Z. Wondafrash

Addis Ababa University, Addis Ababa, Ethiopia
Gebre T. Demoz

Department of Global Health and Infection, Brighton and Sussex Medical School, Brighton, UK
Kebede Deribe

Division of Cardiology, Atlanta Veterans Affairs Medical Center, Decatur, GA, USA
Rupak Desai

Centre for Atmospheric Sciences, Indian Institute of Technology Delhi, New Delhi, India
Sagnik Dey

Department of Community Medicine, University of Peradeniya, Peradeniya, Sri Lanka
Samath D. Dharmaratne

Health Research Section, Nepal Health Research Council, Kathmandu, Nepal
Meghnath Dhimal

Center of Complexity Sciences, National Autonomous University of Mexico, Mexico City, Mexico
Daniel Diaz

Facultad de Medicina Veterinaria y Zootecnia, Autonomous University of Sinaloa, Culiacan Rosales, Mexico
Daniel Diaz

School of Medicine, Federal University of Bahia, Salvador, Brazil
Andre R. Duraes

Institute of Public Health, Federal University of Bahia, Salvador, Brazil
Davide Rasella

Diretoria Médica, Roberto Santos General Hospital, Salvador, Brazil
Andre R. Duraes

Environmental Health Research Center, Kurdistan University of Medical Sciences, Sanandaj, Iran
Roya Ebrahimi, Afshin Maleki & Tahereh Pashaei

Department of Epidemiology and Biostatistics, Kurdistan University of Medical Sciences, Sanandaj, Iran
Ghobad Moradi

Social Determinants of Health Research Center, Kurdistan University of Medical Sciences, Sanandaj, Iran
Bakhtiar Piroozi & Ghobad Moradi

Epidemiology and Biostatistics, Kurdistan University of Medical Sciences, Sanandaj, Iran
Daem Roshani

Clinical Epidemiology and Biostatistics, University of Newcastle, Newcastle, NSW, Australia
Andem Effiong

Department of Basic Sciences, Maragheh University of Medical Sciences, Maragheh, Iran
Aziz Eftekhari

Healthcare Management, Maragheh University of Medical Sciences, Maragheh, Iran
Edris Hasanpoor

Department of Microbiology, Maragheh University of Medical Sciences, Maragheh, Iran
Amir Hasanzadeh

Department of Public Health, and Department of Nutrition and Food Sciences, Maragheh University of Medical Sciences, Maragheh, Iran
Hadi Pourjafar

Medical Research Institute, Alexandria University, Alexandria, Egypt
Iman El Sayed

Pediatric Dentistry and Dental Public Health, Alexandria University, Alexandria, Egypt
Maha El Tantawi

Department of Clinical Pathology, Mansoura University, Mansoura, Egypt
Maysaa El Sayed Zaki

Preventive Dental Sciences, Imam Abdulrahman Bin Faisal University, Dammam, Saudi Arabia
Maha El Tantawi

Forensic Medicine Division, Imam Abdulrahman Bin Faisal University, Dammam, Saudi Arabia
Ritesh G. Menezes

Department of Public Health Sciences, Karolinska Institutet, Stockholm, Sweden
Ziad El-Khatib

Department of Neurobiology, Karolinska Institutet, Stockholm, Sweden
Seyed-Mohammad Fereshtehnejad

Department of Medicine Huddinge, Karolinska Institutet, Stockholm, Sweden
Dara K. Mohammad

Ophthalmic Epidemiology Research Center, Shahroud University of Medical Sciences, Shahroud, Iran
Mohammad Hassan Emamian

Department of Microbiology and Immunology, Suez Canal University, Ismailia, Egypt
Shymaa Enany

Physiology Department, Suez Canal University, Ismailia, Egypt
Sameh Magdeldin

Proteomics and Metabolomics Unit, Suez Canal University, Ismailia, Egypt
Sameh Magdeldin

Epidemiology and Population Health, British Columbia Centre for Excellence in HIV/AIDS, Vancouver, BC, Canada
Oghenowede Eyawo

Faculty of Health Sciences, Simon Fraser University, Burnaby, BC, Canada
Oghenowede Eyawo

College of Medicine, Imam Muhammad Ibn Saud Islamic University, Riyadh, Saudi Arabia
Mohammad Fareed

Department of Psychology, Federal University of Sergipe, Sao Cristovao, Brazil
Andre Faro

Social Determinants of Health Research Center, Hamadan University of Medical Sciences, Hamadan, Iran
Ali Akbar Fazaeli

Department of Epidemiology, Hamadan University of Medical Sciences, Hamadan, Iran
Manoochehr Karami & Salman Khazaei

Department of Environmental Health Engineering, Hamadan University of Medical Sciences, Hamadan, Iran
Mohammad Khazaei & Mostafa Leili

Department of Biostatistics, Hamadan University of Medical Sciences, Hamadan, Iran
Naser Mohammad Gholi Mezerji

Chronic Diseases (Home Care) Research Center, Hamadan University of Medical Sciences, Hamadan, Iran
Morteza Shamsizadeh

Division of Neurology, University of Ottawa, Ottawa, ON, Canada
Seyed-Mohammad Fereshtehnejad

Center for Biotechnology and Fine Chemistry, Catholic University of Portugal, Porto, Portugal
João C. Fernandes

Psychiatry Department, Kaiser Permanente, Fontana, CA, USA
Irina Filip

Department of Health Sciences, A.T. Still University, Mesa, Arizona, USA
Irina Filip

School of Midwifery, A.T. Still University, Mesa, Arizona, USA
Ensiyeh Jenabi

College of Graduate Health Sciences, A.T. Still University, Mesa, Arizona, USA
Amir Radfar

Department of Public Health Medicine, Bielefeld University, Bielefeld, Germany
Florian Fischer

Institute of Gerontology, National Academy of Medical Sciences of Ukraine, Kyiv, Ukraine
Nataliya A. Foigt

Abadan School of Medical Sciences, Abadan, Iran
Masoud Foroutan

Clinical Medicine and Wits Reproductive Health and HIV Insititute, University of the Witwatersrand, Johannesburg, South Africa
Joel M. Francis

Gene Expression & Regulation Program, Cancer Institute (W.I.A), Philadelphia, PA, USA
Takeshi Fukumoto

Department of Dermatology, Kobe University, Kobe, Japan
Takeshi Fukumoto

Dr. Tewelde Legesse Health Sciences College, Mekelle, Ethiopia
Destallem G. Gebre

Department of Epidemiology, Jimma University, Jimma, Ethiopia
Tsegaye T. Gebrehiwot

Vaccines Department, Pfizer Inc., Collegeville, PA, USA
Bradford D. Gessner

Agency of Preventive Medicine, Paris, France
Bradford D. Gessner

Department of Pharmacy, Wollo University, Dessie, Ethiopia
Birhanu Geta

Unit of Academic Primary Care, University of Warwick, Coventry, UK
Paramjit S. Gill

Division of Health Sciences, University of Warwick, Coventry, UK
Olalekan A. Uthman

Adelaide Medical School, University of Adelaide, Adelaide, SA, Australia
Tiffany K. Gill

REQUIMTE/LAQV, Porto, Portugal
Nelson G. M. Gomes

Nursing and Health Sciences Department, University of Massachusetts Boston, Boston, MA, USA
Philimon N. Gona

Department of Biostatistics and Epidemiology, University of Oklahoma, Oklahoma City, OK, USA
Sameer V. Gopalani

Department of Health and Social Affairs, Government of the Federated States of Micronesia, Palikir, Federated States of Micronesia
Sameer V. Gopalani

Occupational and Environmental Epidemiology Section, Cancer Prevention and Research Institute, Florence, Italy
Giuseppe Gorini

Postgraduate Program in Epidemiology, Federal University of Rio Grande do Sul, Porto Alegre, Brazil
Bárbara N. G. Goulart

Health Improvement Directorate, Public Health England, London, UK
Felix Greaves

School of Public Health, University of Haifa, Haifa, Israel
Manfred S. Green & Kerem Shuval

School of Public Health and Preventive Medicine, Monash University, Melbourne, VIC, Australia
Yuming Guo, Thomas R. Hird & Shanshan Li

Centre of Cardiovascular Research and Education in Therapeutics, Monash University, Melbourne, VIC, Australia
Richard Ofori-Asenso

Department of Medicine, Monash University, Melbourne, VIC, Australia
Sojib bin Zaman

Department of Epidemiology and Biostatistics, Zhengzhou University, Zhengzhou, China
Yuming Guo

Obesity Research Center, Research Institute for Endocrine Sciences, Shahid Beheshti University of Medical Sciences, Tehran, Iran
Arvin Haj-Mirzaian

Research Institute for Endocrine Sciences, Shahid Beheshti University of Medical Sciences, Tehran, Iran
Seyed Sina N. Irvani

Foodborne and Waterborne Diseases Research Center, Shahid Beheshti University of Medical Sciences, Tehran, Iran
Hamed Mirjalali & Abbas Yadegar

Prevention of Metabolic Disorders Research Center, Shahid Beheshti University of Medical Sciences, Tehran, Iran
Azra Ramezankhani

Department of Epidemiology, Shahid Beheshti University of Medical Sciences, Tehran, Iran
Seyed Mohammad Riahi & Siamak Sabour

Prevention of Cardiovascular Disease Research Center, Shahid Beheshti University of Medical Sciences, Tehran, Iran
Mahmoud Yousefifard

Department of Health, Safety, and Environment (HSE), Shahid Beheshti University of Medical Sciences, Tehran, Iran
Reza Saeedi

Critical Care Quality Improvement Research Center, Shahid Beheshti University of Medical Sciences, Tehran, Iran
Azra Ramezankhani

Global and Community Mental Health Research Group, University of Macau, Macao, China
Brian J. Hall

School of Health and Environmental Studies, Hamdan Bin Mohammed Smart University, Dubai, United Arab Emirates
Samer Hamidi

Carlos III Health Institute, Biomedical Research Networking Center for Mental Health Network (CIBERSAM), Madrid, Spain
Rafael Tabarés-Seisdedos

Biomedical Research Networking Center for Mental Health Network (CIBERSAM), Madrid, Spain
Josep M. Haro

Research and Development Unit, San Juan de Dios Sanitary Park, Sant Boi De Llobregat, Spain
Josep M. Haro

CIBERSAM, San Juan de Dios Sanitary Park, Sant Boi De Llobregat, Spain
Ai Koyanagi

Independent Consultant, Tabriz, Iran
Hadi Hassankhani

Public Health Department, Mizan-Tepi University, Teppi, Ethiopia
Hamid Y. Hassen

Unit of Epidemiology and Social Medicine, University Hospital Antwerp, Wilrijk, Belgium
Hamid Y. Hassen

School of Public Health, Curtin University, Bentley, Western Australia, Australia
Delia Hendrie & Ted R. Miller

Population Health, Baker Heart and Diabetes Institute, Melbourne, VIC, Australia
Thomas R. Hird

Center of Excellence in Behavioral Medicine, Nguyen Tat Thanh University, Ho Chi Minh, Vietnam
Chi L. Hoang & Giang T. Vu

Center for Excellence in Behavioral Health, Nguyen Tat Thanh University, Ho Chi Minh, Vietnam
Long H. Nguyen & Son H. Nguyen

Social Determinants of Health Research Center, Guilan Road Trauma Research Center, Guilan University of Medical Sciences, Rasht, Iran
Enayatollah Homaie Rad

Guilan Road Trauma Research Center, Guilan University of Medical Sciences, Rasht, Iran
Enayatollah Homaie Rad

Department of Pharmacology and Therapeutics, University of Dhaka, Dhaka, Bangladesh
Naznin Hossain

Computer Science Department, University of Human Development, Sulaimaniyah, Iraq
Mehdi Hosseinzadeh

Information Technology Department, University of Human Development, Sulaimaniyah, Iraq
Aso Mohammad Darwesh

Department of Internal Medicine, Bucharest Emergency Hospital, Bucharest, Romania
Mihaela Hostiuc

Clinical Legal Medicine, National Institute of Legal Medicine Mina Minovici, Bucharest, Romania
Sorin Hostiuc

Division of Information and Computing Technology, Hamad Bin Khalifa University, Doha, Qatar
Mowafa Househ

Qatar Foundation for Education, Science, and Community Development, Doha, Qatar
Mowafa Househ

Faculty of Medicine Tunis, Medicine School of Tunis, Baab Saadoun, Tunisia
Mohamed Hsairi

Global Health and Development Department, Taipei Medical University, Taipei City, Taiwan,
Usman Iqbal

MRC Epidemiology Unit, University of Cambridge, Cambridge, UK
Nazrul Islam

Medical School, Harvard University, Boston, MA, USA
Moritz U.G. Kraemer

Department of Genetics, Harvard University, Boston, MA, USA
Alexandre Pereira

Division of General Internal Medicine and Primary Care, Harvard University, Boston, MA, USA
Aziz Sheikh

Harvard University, Boston, MA, USA
Nazrul Islam

Institute for Physical Activity and Nutrition, Deakin University, Burwood, VIC, Australia
Sheikh Mohammed Shariful Islam

Sydney Medical School, University of Sydney, Sydney, NSW, Australia
Sheikh Mohammed Shariful Islam

Institute of Family Medicine and Public Health, University of Tartu, Tartu, Estonia
Mikk Jürisson

Psychosis Department, Babol Nushirvani University of Technology, Babol, Iran
Nader Jafari Balalami

Faculty of Graduate Studies, University of Colombo, Colombo, Sri Lanka
Achala U. Jayatilleke

Institute of Medicine, University of Colombo, Colombo, Sri Lanka
Achala U. Jayatilleke

Environmental Research Center, Duke Kunshan University, Kunshan, China
John S. Ji

Department of Medicine, University of Miami, Atlantis, FL, USA
Yash B. Jobanputra

Beijing Institute of Ophthalmology, Beijing Tongren Hospital, Beijing, China
Jost B. Jonas

Social Determinants of Health Research Center, University of Social Welfare and Rehabilitation Sciences, Tehran, Iran
Zahra Jorjoran Shushtari

University of Social Welfare and Rehabilitation Sciences, Tehran, Iran
Mehdi Noroozi

Faculty of Medicine and Health Sciences, University of Opole, Opole, Poland
Jacek J. Jozwiak

Department of Family Medicine and Public Health, University of Opole, Opole, Poland
Jacek J. Jozwiak

Isfahan University of Medical Sciences, Isfahan, Iran
Hamed Kalani

Research and Development, Australian Red Cross Blood Service, Sydney, NSW, Australia
Surendra Karki

School of Public Health and Community Medicine, University of New South Wales, Sydney, NSW, Australia
Surendra Karki

School of Medicine, University of New South Wales, Sydney, NSW, Australia
Pallab K. Maulik

Odel Campus, University of Nairobi, Nairobi, Kenya
Peter N. Keiyoro

Department of Psychiatry, University of Nairobi, Nairobi, Kenya
Manasi Kumar

Michigan State University, East Lansing, MI, USA
Grant R. Kemp

Department of Public Health and Community Medicine, Jordan University of Science and Technology, Ramtha, Jordan
Yousef S. Khader

Epidemiology and Biostatistics Department, Health Services Academy, Islamabad, Pakistan
Ejaz A. Khan

Population Studies, International Institute for Population Sciences, Mumbai, India
Junaid Khan

International Institute for Population Sciences, Mumbai, India
Pushpendra Kumar & Parul Puri

Department of Internal Medicine, John H. Stroger, Jr. Hospital of Cook County, Chicago, IL, USA
Muhammad S. Khan

Department of Internal Medicine, Dow University of Health Sciences, Karachi, Pakistan
Muhammad S. Khan, Tariq J. Siddiqi & Muhammad S. Usman

Institute of Health Policy and Management, Seoul National University, Seoul, South Korea
Young-Ho Khang

Department of Health Policy and Management, Seoul National University, Seoul, South Korea
Young-Ho Khang

Faculty of Health and Wellbeing, Sheffield Hallam University, Sheffield, UK
Khaled Khatab

Department of Arts and Sciences, Ohio University, Zanesville, OH, USA
Khaled Khatab

Internal Medicine and Gastroenterology Department, National Hepatology and Tropical Research Institute, Cairo, Egypt
Amir Khater

Department of Nutrition and Health Science, Ball State University, Muncie, IN, USA
Jagdish Khubchandani

School of Medicine, Xiamen University Malaysia, Sepang, Malaysia
Yun Jin Kim

Department of Nutrition, Simmons College, Boston, MA, USA
Ruth W. Kimokoti

Department of Health Management and Health Economics, Kristiania University College, Oslo, Norway
Adnan Kisa

Department of Health Services Policy and Management, University of South Carolina, Columbia, SC, USA
Adnan Kisa

Nursing and Health Promotion, Oslo Metropolitan University, Oslo, Norway
Sezer Kisa

Department of Public Health, Debre Berhan University, Debre Berhan, Ethiopia
Tufa Kolola

Independent Consultant, Jakarta, Indonesia
Soewarta Kosen

Department of Internal and Pulmonary Medicine, Sheri Kashmir Institute of Medical Sciences, Srinagar, India
Parvaiz A. Koul

Department of Anthropology, Panjab University, Chandigarh, India
Kewal Krishan

Psychology and Health Promotion, University of Kwazulu-Natal, Durban, South Africa
Nuworza Kugbey

Department of Public Health Medicine, University of Kwazulu-Natal, Durban, South Africa
Desmond Kuupiel, Yoshan Moodley & Kelechi E. Oladimeji

Department of Psychology, University College London, London, UK
Manasi Kumar

Institute of Child Health, University College London, London, UK
Joseph L. Ward

Nursing, St. John of God Hospital, Duayaw Nkwanta, Ghana
Desmond Kuupiel

National Institute for Health Research, Oxford Biomedical Research Centre (NIHR), Oxford, UK
Ben Lacey

Department of Pediatrics, Post Graduate Institute of Medical Education and Research, Chandigarh, India
Sheetal D. Lad

Department of Community and Family Medicine, Academy of Medical Science, Baghdad, Iraq
Faris H. Lami

Department of Medical Sciences, Uppsala University, Uppsala, Sweden
Anders O. Larsson

Department of Clinical Chemistry and Pharmacology, Uppsala University Hospital, Uppsala, Sweden
Anders O. Larsson

Department of Medicine, University of Malaya, Kuala Lumpur, Malaysia
Lee-ling Lim

Department of Medicine and Therapeutics, The Chinese University of Hong Kong, Shatin, China
Lee-ling Lim

Department of Dentistry, Radboud University, Nijmegen, The Netherlands
Stefan Listl

Section for Translational Health Economics, Heidelberg University Hospital, Heidelberg, Germany
Stefan Listl

Department of Vector Biology, Liverpool School of Tropical Medicine, Liverpool, UK
Joshua Longbottom

Alliance for Improving Health Outcomes, Inc., Quezon City, The Philippines
Jaifred Christian F. Lopez

Institute of Nutrition, Friedrich Schiller University Jena, Jena, Germany
Stefan Lorkowski

Competence Cluster for Nutrition and Cardiovascular Health (NUTRICARD), Jena, Germany
Stefan Lorkowski

Department of Cardiology, Damietta University, Damietta, Egypt
Hassan Magdy Abd El Razek

Ophthalmology Department, Aswan Faculty of Medicine, Aswan, Egypt
Muhammed Magdy Abd El Razek

Department of Maternal and Child Nursing and Public Health, Federal University of Minas Gerais, Belo Horizonte, Brazil
Deborah C. Malta

Post-graduate Program in Infectious Diseases and Tropical Medicine, Federal University of Minas Gerais, Belo Horizonte, Brazil
Bruno P. Sao Jose

Institute for Social Science Research, The University of Queensland, Brisbane, QLD, Australia
Abdullah A. Mamun

Surgery Department, Emergency University Hospital Bucharest, Bucharest, Romania
Ana-Laura Manda

Campus Caucaia, Federal Institute of Education, Science and Technology of Ceará, Caucaia, Brazil
Francisco R. Martins-Melo

Faculty of Health and Education, Botho University-Botswana, Gaborone, Botswana
Anthony A. Masaka

Research Department, The George Institute for Global Health, New Delhi, India
Pallab K. Maulik

Department of Biology and Biological Engineering, Chalmers University of Technology, Gothenburg, Sweden
Mohsen Mazidi

Preventive Oncology Department, National Institute of Cancer Prevention and Research, Noida, India
Ravi Mehrotra

Department of Epidemiology and Biostatistics, University of California San Francisco, San Francisco, CA, USA
Kala M. Mehta

Peru Country Office, United Nations Population Fund (UNFPA), Lima, Peru
Walter Mendoza

Neurocenter, Helsinki University Hospital, Helsinki, Finland
Atte Meretoja

Breast Surgery Unit, Helsinki University Hospital, Helsinki, Finland
Tuomo J. Meretoja

School of Health Sciences, University of Melbourne, Parkville, VIC, Australia
Atte Meretoja

University of Helsinki, Helsinki, Finland
Tuomo J. Meretoja

Clinical Microbiology and Parasitology Unit, Dr. Zora Profozic Polyclinic, Zagreb, Croatia
Tomislav Mestrovic

University Centre Varazdin, University North, Varazdin, Croatia
Tomislav Mestrovic

Pacific Institute for Research & Evaluation, Calverton, MD, USA
Ted R. Miller

Health, Evidence, and Impact, McMaster University, Hamilton, ON, Canada
Edward J. Mills

Department of Psychiatry and Behavioural Neurosciences, McMaster University, Hamilton, ON, Canada
Andrew T. Olagunju

Department of Computer Science and Software Engineering, University of Western Australia, Perth, WA, Australia
George J. Milne

Department of Public Health, Amrita Institute of Medical Sciences, Kochi, India
GK Mini

Golestan Research Center of Gastroenterology and Hepatology, Golestan University of Medical Sciences, Gorgan, Iran
Gholamreza Roshandel

Golestan University of Medical Sciences, Gorgan, Iran
Seyed Mostafa Mir

Faculty of General Medicine, Kyrgyz State Medical Academy, Bishkek, Kyrgyzstan
Erkin M. Mirrakhimov

Department of Atherosclerosis and Coronary Heart Disease, National Center of Cardiology and Internal Disease, Bishkek, Kyrgyzstan
Erkin M. Mirrakhimov

Department of Food Technology, College of Agriculture, Salahaddin University-Erbil, Erbil, Iraq
Dara K. Mohammad

School of Pharmacy, Haramaya University, Harar, Ethiopia
Ammas S. Mohammed

Health Systems and Policy Research Unit, Ahmadu Bello University, Zaria, Nigeria
Shafiu Mohammed

Department of Community Medicine, Ahmadu Bello University, Zaria, Nigeria
Mu'awiyyah B. Sufiyan

Faculty of Life Sciences and Medicine, King's College London, London, UK
Mariam Molokhia

Clinical Epidemiology and Public Health Research Unit, Burlo Garofolo Institute for Maternal and Child Health, Trieste, Italy
Lorenzo Monasta

Department of Mathematical Sciences, University of Bath, Bath, UK
Paula Moraga

International Laboratory for Air Quality and Health, Queensland University of Technology, Brisbane, QLD, Australia
Lidia Morawska

School of Exercise and Nutrition Sciences, Queensland University of Technology, Brisbane, QLD, Australia
Quyen G. To

Competence Center of Mortality-Follow-Up, German National Cohort
Ronny Westerman

Federal Institute for Population Research, Wiesbaden, Germany
Ulrich O. Mueller

Center for Population and Health, Wiesbaden, Germany
Ulrich O. Mueller

Department of Pediatric Medicine, Nishtar Medical University, Multan, Pakistan
Ghulam Mustafa

Department of Pediatrics & Pediatric Pulmonology, Institute of Mother & Child Care, Multan, Pakistan
Ghulam Mustafa

Research and Analytics, Initiative for Financing Health and Human Development, Chennai, India
Ahamarshan J. Nagarajan

Founder, Research and Analytics, Bioinsilico Technologies, Chennai, India
Ahamarshan J. Nagarajan

Suraj Eye Institute, Nagpur, India
Vinay Nangia

Cochrane South Africa, South African Medical Research Council, Cape Town, South Africa
Duduzile E. Ndwandwe, Chukwudi A. Nnaji & Evanson Z. Sambala

Department of Cardiology, Cardio-aid, Bucharest, Romania
Ruxandra I. Negoi

Department of Biological Sciences, University of Embu, Embu, Kenya
Josephine W. Ngunjiri

Institute for Global Health Innovations, Duy Tan University, Hanoi, Vietnam
Huong L.T. Nguyen

Public Health Science DepartmentSchool of Public Health and Family Medicine, University of Cape Town, Cape Town, South Africa
Chukwudi A. Nnaji

Department of Medicine, University of Cape Town, Cape Town, South Africa
Jean Jacques Noubiap, Karen Sliwa

Independent Consultant, Accra, Ghana
Richard Ofori-Asenso

Center for the Aid Program of Research in South Africa (CAPRISA) TB and HIV Pathogenesis Unit, United Nations Programme on HIV/AIDS (UNAIDS), Durban, South Africa
Kelechi E. Oladimeji

Department of Psychiatry, University of Lagos, Lagos, Nigeria
Andrew T. Olagunju

Gastroenterology and Liver Disease Research Center, A.C.S. Medical College and Hospital, Tehran, Iran
Meysam Olfatifar

Department of Health Management and Economics, A.C.S. Medical College and Hospital, Tehran, Iran
Hasan Yusefzadeh

Department of Food Science and Postharvest Technology, Gulu University, Gulu, Uganda
Solomon Olum

Ghent University, Ghent, Belgium
Solomon Olum

Centre for Healthy Start Initiative, Lagos, Nigeria
Bolajoko O. Olusanya

Department of Pharmacology and Therapeutics, University of Nigeria Nsukka, Enugu, Nigeria
Obinna E. Onwujekwe

Graduate School of Public Health, San Diego State University, San Diego, CA, USA
Eyal Oren

Center for Health Systems Research, National Institute of Public Health, Cuernavaca, Mexico
Doris D. V. Ortega-Altamirano

School of Medicine, Autonomous University of Madrid, Madrid, Spain
Alberto Ortiz

Hospital Universitario de la Princesa, Autonomous University of Madrid, Madrid, Spain
Joan B. Soriano

Department of Nephrology and Hypertension, The Institute for Health Research Foundation Jiménez Díaz University Hospital, Madrid, Spain
Alberto Ortiz

Environmental Mangement and Toxicology, University of Benin, Benin City, Nigeria
Osayomwanbo Osarenotor

Faculty of Geoinformation Science and Earth Observation, University of Twente, Enschede, The Netherlands
Frank B. Osei

Department of Mathematics and Statistics, University of Energy and Natural Resources, Sunyani, Ghana
Frank B. Osei

Analytical Center, Moscow Institute of Physics and Technology, Dolgoprudny, Russia
Stanislav S. Otstavnov

Department of Information Technologies and Management, Moscow Institute of Physics and Technology, Dolgoprudny, Russia
Sergey K. Vladimirov

Committee for the Comprehensive Assessment of Medical Devices and Information Technology, Health Technology Assessment Association, Moscow, Russia
Stanislav S. Otstavnov

Department of Tb & Respiratory Medicine, Jagadguru Sri Shivarathreeswara University, Mysore, India
Mahesh P A

Department of Medicine, Ottawa Hospital Research Institute, Ottawa, ON, Canada
Smita Pakhale

Department of Medical Humanities and Social Medicine, Kosin University, Busan, South Korea
Eun-Kee Park

Research and Evaluation, Population Council, New Delhi, India
Sangram K. Patel

Indian Institute of Health Management Research University, Jaipur, India
Sangram K. Patel

Center for Research and Innovation, Ateneo De Manila University, Pasig City, The Philippines
Veincent Christian F. Pepito

Laboratory of Genetics and Molecular Cardiology, University of São Paulo, Sao Paulo, Brazil
Alexandre Pereira

Department of Pathology and Legal Medicine, University of São Paulo, Sao Paulo, Brazil
Marcos R. Tovani-Palone

Department of Psychiatry, University of São Paulo, Sao Paulo, Brazil
Yuan-Pang Wang

Department of Cardiology, University of Bern, Bern, Switzerland
Thomas Pilgrim

National Centre for Register-Based Research, Aarhus University, Aarhus, Denmark
Oleguer Plana-Ripoll

Non-communicable Diseases Research Center, Alborz University of Medical Sciences, Karaj, Iran
Mostafa Qorbani

Department of Health Promotion and Education, Alborz University of Medical Sciences, Karaj, Iran
Faramarz Shaahmadi

Biomedical Engineering, Amirkabir University of Technology, Tehran, Iran
Mohammad Rabiee

Medichem, Barcelona, Spain
Amir Radfar

Department of Nephrology, Nizam's Institute of Medical Sciences, Hyderabad, India
Sree Bhushan Raju

Policy Research Institute, Kathmandu, Nepal
Chhabi L. Ranabhat

Institute for Poverty Alleviation and International Development, Yonsei University, Wonju, South Korea
Chhabi L. Ranabhat

Gonçalo Moniz Institute, Oswaldo Cruz Foundation, Salvador, Brazil
Davide Rasella

Department of Epidemiology, Birjand University of Medical Sciences, Birjand, Iran
Seyed Mohammad Riahi

Department of Clinical Research, Federal University of Uberlândia, Uberlândia, Brazil
Leonardo Roever

Department of Public Health, Wollega University, Nekemte, Ethiopia
Elias E.M. Roro

School of Biotechnology, Ikiam Amazon Regional University, Ciudad De Tena, Ecuador
Enrico Rubagotti

Department of Ocean Science and Engineering, Southern University of Science and Technology, Shenzhen, China
Enrico Rubagotti

Department of Biomedical Sciences, University of Sassari, Sassari, Italy
Salvatore Rubino

Department of Pathology Al-Imam Mohammad Ibn Saud Islamic University, Riyadh, Saudi Arabia
Nasir Salam

School of Health and Policy Management, Faculty of Health, York University, Toronto, ON, Canada
Payman Salamati

Research Deputy, Taleghani Hospital, Kermanshah, Iran
Saleh Salehi Zahabi

Center for Health Policy & Center for Primary Care and Outcomes Research, Stanford University, Stanford, CA, USA
Joshua A. Salomon

Department of Entomology, Ain Shams University, Cairo, Egypt
Abdallah M. Samy

Department of Community Medicine, PSG Institute of Medical Sciences and Research, Coimbatore, India
Sivan Y. Saraswathy

PSG-FAIMER South Asia Regional Institute, Coimbatore, India
Sivan Y. Saraswathy

Department of Health and Society, Faculty of Medicine, University of Applied and Environmental Sciences, Bogotá, Colombia
Rodrigo Sarmiento-Suárez

Surgery Department, Hamad Medical Corporation, Doha, Qatar
Brijesh Sathian

Faculty of Health & Social Sciences, Bournemouth University, Bournemouth, UK
Brijesh Sathian

Department of Psychology, University of Alabama at Birmingham, Birmingham, AL, USA
David C. Schwebel

Department of Epidemiology, University of Alabama at Birmingham, Birmingham, AL, USA
Jasvinder A. Singh

Department of Medicine, University of Alabama at Birmingham, Birmingham, AL, USA
Jasvinder A. Singh

Independent Consultant, Karachi, Pakistan
Masood Ali Shaikh

School of Medicine, Dezful University of Medical Sciences, Dezful, Iran
Mehran Shams-Beyranvand

HIV/STI Surveillance Research Center, Kerman University of Medical Sciences, Kerman, Iran
Hamid Sharifi

Department of Health Management, Policy and Economics, Kerman University of Medical Sciences, Kerman, Iran
Vahid Yazdi-Feyzabadi

Health Services Management Research Center, Kerman University of Medical Sciences, Kerman, Iran
Vahid Yazdi-Feyzabadi

University School of Management and Entrepreneurship, Delhi Technological University, New Delhi, India
Rajesh Sharma

Usher Institute of Population Health Sciences and Informatics, University of Edinburgh, Edinburgh, UK
Aziz Sheikh

National Institute of Infectious Diseases, Tokyo, Japan
Mika Shigematsu

Finnish Institute of Occupational Health, Helsinki, Finland
Rahman Shiri

Institute of Medical Epidemiology, Martin Luther University Halle-Wittenberg, Halle, Germany
Ivy Shiue

Department of Epidemiology, School of Preventive Oncology, Patna, India
Dhirendra N. Sinha

Department of Epidemiology, Healis Sekhsaria Institute for Public Health, Mumbai, India
Dhirendra N. Sinha

Department of Physiotherapy and Occupational Therapy, Næstved-Slagelse-Ringsted Hospitals, Slagelse, Denmark
Malede M. Sisay

Medical Division, German Leprosy and TB Relief Association Ethiopia, Addis Ababa, Ethiopia
Solomon Sisay

Department of Medicine, University of Calgary, Calgary, AB, Canada
Ranjani Somayaji

Centro de Investigación en Red de Enfermedades Respiratorias (CIBERES), Institute of Health Carlos Iii, Madrid, Spain
Joan B. Soriano

Division of Community Medicine, International Medical University, Kuala Lumpur, Malaysia
Chandrashekhar T. Sreeramareddy

Department of Nursing, Muhammadiyah University of Surakarta, Kartasura, Indonesia
Agus Sudaryanto

Department of Criminology, Law, and Society, University of California Irvine, Irvine, CA, USA
Bryan L. Sykes

Neurology Department, Sree Chitra Tirunal Institute for Medical Sciences and Technology, Trivandrum, India
PN Sylaja

Department of Medicine, University of Valencia, Valencia, Spain
Rafael Tabarés-Seisdedos

School of Social Work, University of Illinois, Urbana, IL, USA
Karen M. Tabb

Cancer Control Center, Osaka International Cancer Institute, Osaka, Japan
Takahiro Tabuchi

University Institute "Egas Moniz", Monte Da Caparica, Portugal
Nuno Taveira

Research Institute for Medicines, Faculty of Pharmacy of Lisbon, University of Lisbon, Lisbon, Portugal
Nuno Taveira

College of Medicine, Alfaisal University, Riyadh, Saudi Arabia
Mohamad-Hani Temsah

Anesthesiology Department, University of Virginia, Charlottesville, VA, USA
Abdullah S. Terkawi

Syrian Expatriate Medical Association (SEMA), Charlottesville, VA, USA
Abdullah S. Terkawi

Department of Public Health and Community Medicine, Central University Kerala, Kasaragod, India
Kavumpurathu R. Thankappan

Department of Health Economics, Hanoi Medical University, Hanoi, Vietnam
Bach Xuan Tran

Department of Molecular Medicine and Pathology, University of Auckland, Auckland, New Zealand
Khanh B. Tran

Clinical Hematology and Toxicology, Military Medical University, Hanoi, Vietnam
Khanh B. Tran

Gomal Center of Biochemistry and Biotechnology, Gomal University, Dera Ismail Khan, Pakistan
Irfan Ullah

TB Culture Laboratory, Mufti Mehmood Memorial Teaching Hospital, Dera Ismail Khan, Pakistan
Irfan Ullah

Department of Education and Health, Trauma Research Center, Tehran, Iran
Amir Vahedian-Azimi

Critical and Intensive Care Department, Trauma Research Center, Tehran, Iran
Amir Vahedian-Azimi

Argentine Society of Medicine, Ciudad De Buenos Aires, Argentina
Pascual R. Valdez

Velez Sarsfield Hospital, Buenos Aires, Argentina
Pascual R. Valdez

University Medical Center Groningen, University of Groningen, Groningen, The Netherlands
Job F. M. Van Boven

Department of General Practice, University Medical Center Groningen, Groningen, The Netherlands
Job F. M. Van Boven

Ukk Institute, Tampere, Finland
Tommi J. Vasankari

Psychosocial Injuries Research Center, Ilam University of Medical Sciences, Ilam, Iran
Yousef Veisani

Raffles Neuroscience Centre, Raffles Hospital, Singapore, Singapore
Narayanaswamy Venketasubramanian

Yong Loo Lin School of Medicine, National University of Singapore, Singapore, Singapore
Narayanaswamy Venketasubramanian

Department of Medical and Surgical Sciences, University of Bologna, Bologna, Italy
Francesco S. Violante

Occupational Health Unit, Sant'orsola Malpighi Hospital, Bologna, Italy
Francesco S. Violante

Department of Information and Internet Technologies, I.m. Sechenov First Moscow State Medical University, Moscow, Russia
Sergey K. Vladimirov

Department of Health Care Administration and Economy, National Research University Higher School of Economics, Moscow, Russia
Vasily Vlassov

Foundation University Medical College, Foundation University, Rawalpindi, Pakistan
Yasir Waheed

Department of Epidemiology and Biostatistics, Wuhan University, Wuhan, China
Yafeng Wang & Chuanhua Yu

Global Health Institute, Wuhan University, Wuhan, China
Chuanhua Yu

Department of Preventative Medicine, Wuhan University, Wuhan, China
Yunquan Zhang

Cardiology Department, Royal Children's Hospital, Melbourne, VIC, Australia
Robert G. Weintraub

Murdoch Childrens Research Institute, Melbourne, VIC, Australia
Robert G. Weintraub

Cochrane South Africa, Medical Research Council South Africa, Cape Town, South Africa
Charles S. Wiysonge

Department of Global Health, Stellenbosch University, Cape Town, South Africa
Charles S. Wiysonge

Zhejiang Spine Research Center, Wenzhou Medical University, Wenzhou, China
Ai-Min Wu

School of Medicine, Nanjing University, Nanjing, China
Gelin Xu

Department of Diabetes and Metabolic Diseases, University of Tokyo, Tokyo, Japan
Tomohide Yamada

Department of Pediatrics, University of Jos, Jos, Nigeria
Christopher S. Yilgwan

Department of Pediatrics, Jos University Teaching Hospital, Jos, Nigeria
Christopher S. Yilgwan

Department of Psychopharmacology, National Center of Neurology and Psychiatry, Tokyo, Japan
Naohiro Yonemoto

Health Economics & Finance, Global Health, Jackson State University, Jackson, Mississppi, USA
Mustafa Z. Younis

Research Center for Public Health, Tsinghua University, Peking, China
Mustafa Z. Younis

Electrical Engineering, Institute for Research in Fundamental Sciences, Tehran, Iran
Erfan Zabeh

Maternal and Child Wellbeing Unit, African Population Health Research Centre, Nairobi, Kenya
Taddese A. Zerfu

Public Health Department, Dilla University, Dilla, Ethiopia
Taddese A. Zerfu

School of Public Health, Wuhan University of Science and Technology, Wuhan, China
Yunquan Zhang
